# Analysis of associations between emotions and activities of drug users and their addiction recovery tendencies from social media posts using structural equation modeling

**DOI:** 10.1186/s12859-020-03893-9

**Published:** 2020-12-30

**Authors:** Deeptanshu Jha, Rahul Singh

**Affiliations:** Department of Computer Science, San Francisco State University, 1600 Holloway Ave., San Francisco, CA 94132 USA

**Keywords:** Structural equation modeling, Social media, Text mining, Opioid epidemic, Personalized interventions, Substance misuse disorder, Addiction recovery, Reddit, Online communities

## Abstract

**Background:**

Addiction to drugs and alcohol constitutes one of the significant factors underlying the decline in life expectancy in the US. Several context-specific reasons influence drug use and recovery. In particular emotional distress, physical pain, relationships, and self-development efforts are known to be some of the factors associated with addiction recovery. Unfortunately, many of these factors are not directly observable and quantifying, and assessing their impact can be difficult. Based on social media posts of users engaged in substance use and recovery on the forum Reddit, we employed two psycholinguistic tools, Linguistic Inquiry and Word Count and Empath and activities of substance users on various Reddit sub-forums to analyze behavior underlining addiction recovery and relapse. We then employed a statistical analysis technique called structural equation modeling to assess the effects of these latent factors on recovery and relapse.

**Results:**

We found that both emotional distress and physical pain significantly influence addiction recovery behavior. Self-development activities and social relationships of the substance users were also found to enable recovery. Furthermore, within the context of self-development activities, those that were related to influencing the mental and physical well-being of substance users were found to be positively associated with addiction recovery. We also determined that lack of social activities and physical exercise can enable a relapse. Moreover, geography, especially life in rural areas, appears to have a greater correlation with addiction relapse.

**Conclusions:**

The paper describes how observable variables can be extracted from social media and then be used to model important latent constructs that impact addiction recovery and relapse. We also report factors that impact self-induced addiction recovery and relapse. To the best of our knowledge, this paper represents the first use of structural equation modeling of social media data with the goal of analyzing factors influencing addiction recovery.

## Background

### Introduction

Substance use constitutes a major contemporary health epidemic. There were 70,237 substance use overdose deaths in 2017, which was a 9.6% increase from 2016 [1]. In the US, abuse of alcohol and other illicit drugs is estimated to lead to a monetary impact of over $740 billion annually because of increased expenses related to loss of work productivity, health care, and crime [2]. Substance use can also increase the risk for liver [3], or lung diseases [4], and especially infectious diseases such as Hepatitis B, or C, and HIV/AIDS [5].

Drug addiction was usually considered a moral or character flaw. This view has undergone a significant change and addiction is now considered a chronic illness characterized by health deterioration, poor social functioning, and loss of control over substance use [6]. Substance use has also been established to change the brain function and makes a user crave drugs. The substance use journey typically begins with experimentation and because of the perceived positive effects, a person gets addicted. After an individual decides to break the addiction cycle, they typically experience physical and emotional withdrawals that are manifested through sadness, restlessness, anxiety, nausea, vomiting, sweating, and cramping. Depending on factors such as the substances used as well as the amount and duration of use, such symptoms typically last for 3–5 days and can be managed by medications, vitamins, and exercise [2]. The notion of “recovery” is polysemous in that it may be considered as an ongoing process or as a granular event [7]. Regardless, recovery is a long-term process requiring continuous effort and diligence [2]. Substance withdrawal management regimes that can lead to recovery from addiction involve managing both physical and emotional symptoms experienced by individuals as they give up drugs. To manage these symptoms, individuals are typically recommended to focus on self-development [8, 9] with the help of their families, and friends [2]. Many individuals however, relapse into drug use because they fail to follow substance use disorder treatment regimens [10].

Though managing emotional and physical symptoms during drug withdrawals is manifestly important, these constructs are multifarious, latent (i.e. not directly observable), and difficult or impossible to directly measure. In this paper, we have proposed the use of structural equation modeling (SEM)—a multivariate latent variable modeling technique to estimate critical latent constructs (italicized hereafter) such as *emotional distress*, *physical pain*, *self-development*, and *relationships* by analyzing social media activities of substance users. Social media has generated recent interest as a novel source of information in drug abuse epidemiology [11–25]. Being semi-anonymous, social media consists of unfiltered and self-reported conversations and activities of an individual. Of the different social media platforms, we used drug use and recovery data available on Reddit. This social media forum is the fifth most visited website in the USA and has over 330 million active users [26]. Reddit is a community-based social media forum where the communities (called subreddits) are created based on common interest. Members of the subreddit can post, vote, and comment in the subreddit. Each subreddit has moderators who ensure that the content posted by the members of the subreddit are topically focused. At the time of writing, there are more than 138,000 subreddits on Reddit [26], with a number of subreddits focusing on recreational drug use (RDU) and drug addiction recovery (DAR).

### Problem formulation and overview of proposed approach

Our aim was to determine the effect of emotional distress, physical pain, self-development efforts, relationships (of the drug user), and geographic disparities on drug addiction recovery and relapse, using SEM as a rigorous modeling methodology. Solving this problem required addressing the following sub-problems: first, we needed to identify and determine the instances of emotional distress, physical pain, self-development efforts, relationships, and geographic disparities in the social media posts and activity of the drug users. Then, we had to come up with a model to infer the relationships between the unobserved constructs (emotional distress, physical pain, self-development efforts, and relationships) and the observable construct drug addiction recovery (determined by observing if a user posted in a drug addiction recovery forum). Our approach consisted of the following steps: (1) we used two psychometrically validated dictionaries, namely, Linguistic Inquiry and Word Count (LIWC) and Empath, to identify instances of emotional distress, physical pain, relationships, self-development efforts, and geographic disparities present in the posts of the drug user. (2) We also utilized the forum activity of the users on Reddit to identify the instances of self-development efforts and relationships. (3) We applied SEM to identify and quantify the relationship between emotional distress, physical pain, self-development, relationships, and geographic disparities on one hand and drug addiction recovery and relapse on the other.

### Prior work

A number of recent works have utilized data from social media in conjunction with methods from machine learning and natural language processing to study and understand patterns associated with a diverse set of health-related issues, such as influenza [27], mental health [28], and suicidal ideation [29]. In terms of studying substance abuse, early works focused on manual identification of themes and tonality of the drug use posts on social media [12, 13]. The image-based social media platform Instagram was analyzed to conduct content analysis for codeine misuse in [14]. Studies have also investigated the use of social media for examining geographic differences in opioid-related discussions [15] and identified topics related to substance delivery methods, drug types, and other factors associated with recreational drug use [16]. In [17] transductive classification was applied to identify opioid addicts on Twitter. Other works have identified opioid use related tweets [18] and studied information sharing amongst drug users on Reddit [19]. Drug addiction recovery has been the focus of far fewer works. Among the latter, in our previous work Eshleman et al. [20], random forests were used with subreddit activity as features to identify users open to addiction recovery interventions in a predictive setting. The Gini impurity criterion, which measures how often a random element from a set would be labeled incorrectly if labeled according to the distribution of labels in the set, was used to rank the different subreddits on the basis of their importance. This analysis found correlations amongst subreddit categories, such as, mental health, spirituality, and relationships with addiction recovery behavior. The SEM model in the current work was developed using two latent variables—“relationships” and “mental and physical well-being”, both of which were directly inspired by findings reported in [20]. In particular, we used user activity in the following subreddits: “relationships”, “relationship_advice”, “parenting”, and “childfree” to reflect the latent variable “*relationships*”. Similarly, we used subreddits, such as, “meditation”, “yoga”, “gainit”, “bodyweightfitness”, and “running” to estimate the latent variable “*mental and physical well-being*”. In other works, MacLean et al. [21], used a trans-theoretical model of behavior change to predict the stages of addiction recovery and relapse. Lu et al. [22], used the cox regression model to identify transitions to addiction recovery subreddits. Chancellor et al. [23], studied recovery-related posts on Reddit to identify clinically unverified treatments for drug withdrawal popular amongst drug users on Reddit. Rubya et al. [24]., investigated how users in online recovery communities enact anonymity Finally, Tamersoy et al. [25], studied Reddit forums to characterize smoking and drinking abstinence and were able to predict long-term and short-term abstinence.

The current work addresses two outstanding issues in this problem domain at the state-of-the-art: *first*, drug addiction recovery and relapse involves (latent) variables that cannot be directly measured and have to be inferred from observable variables. *Second*, the addiction and recovery processes involve complex interplay of relationships between the observed and latent variables, which needs to be characterized. Current methods in the area involve variables that have to be explicitly measured and consequently are incapable of addressing these two issues. We demonstrate how SEM can be a powerful framework to test, evaluate, and characterize multivariate causal relationships in addiction recovery and relapse where both observable and latent factors are involved.

## Results

### The withdrawal management model obtained using LIWC variables

#### Summary statistics

**Fig. 1:**
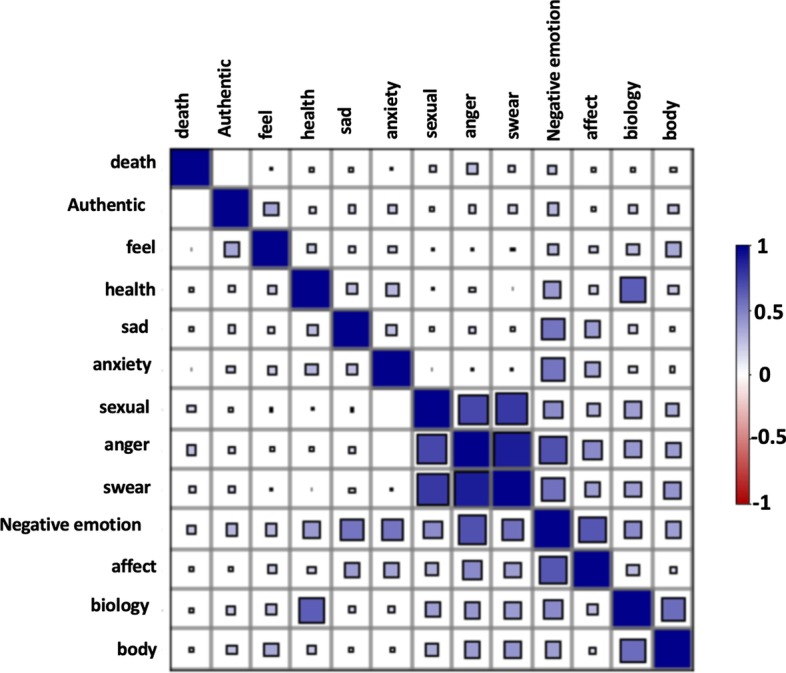
Correlation diagram of the LIWC variables present in the withdrawal management model (see also Table 1). Positive correlations are color-coded in blue and negative correlations in red. The size of each square represents the magnitude of the correlations. As this visualization indicates, every variable-pair in the model was positively correlated. The two highest correlations values were observed for the variable pairs “anger” and “swear” followed by “anger” and “sexual”

In Table 1 and Fig. 1 we present the correlations between the LIWC indicators in the withdrawal management model. From this data we observe that the majority of the LIWC variables are positively correlated with each other. We also observe some correlations that are not so obvious. For example, we see that the second (0.78) and third highest (0.72) correlations were for the categories “*swear*” and “*sexual*”, and “*anger*” and “*sexual*”. As displayed in Table 2, the high correlation was due to common expletives in these categories. We also see that the LIWC category “*health*” had high correlation values with categories, such as “*negative emotion*” (0.39), “*sad*” (0.25), and “*anxiety*” (0.28). This indicates that users in our dataset usually talked about health (physical symptoms) in the context of negative emotions- as may be expected for users experiencing withdrawals.

**Table 1:** Correlation matrix of the LIWC variables present in the withdrawal management model

	Negative emotion	Health	Authentic	Bio	Sad	Anger	Affect	Anxiety	Swear	Sexual	Feel	Death	Body
Negative emotion	1	0.39	0.28	0.45	0.54	0.67	0.66	0.54	0.55	0.45	0.28	0.22	0.38
Health		1	0.15	0.62	0.25	0.11	0.19	0.28	0.04	0.05	0.20	0.12	0.22
Authentic			1	0.21	0.17	0.16	0.09	0.19	0.17	0.11	0.34	-0.00	0.24
Bio				1	0.18	0.40	0.27	0.17	0.38	0.37	0.26	0.11	0.57
Sad					1	0.15	0.38	0.24	0.10	0.09	0.14	0.10	0.10
Anger						1	0.45	0.01	0.89	0.72	0.08	0.24	0.38
Affect							1	0.35	0.37	0.31	0.20	0.10	0.15
Anxiety								1	− 0.05	− 0.02	0.2	0.04	0.11
Swear									1	0.78	0.06	0.15	0.41
Sexual										1	0.07	0.15	0.32
Feel											1	0.04	0.34
Death												1	0.11
Body													1

**Table 2:** LIWC variables in our model for users who display or do not display addiction recovery tendencies

LIWC category	Example posts	Individuals displaying signs of addiction recovery		Individuals not displaying signs of addiction recovery		
		Mean	SD	Mean	SD	*p* <
Feel	I feel really proud to get off roxies, but I feel awful mentally. I have so much anxiety, I feel it building in my chest. Another user posted they felt the same way I feel. How do you get rid of this feeling!?	0.33	0.12	0.27	0.13	0.005
Anger	Argh. I might aswell f***ing cold turkey it. Goddamnit	0.18	0.12	0.14	0.12	0.005
Authentic		0.78	0.16	0.71	0.20	0.005
Sexual	God f***ing damn it! F*** today! Today is the shittiest f***ing damn day! These withdrawals have me sick as f***! I feel like I am screwed forever	0.08	0.09	0.07	0.09	0.005
Negative emotion	So, I'm at 7 days clean. I was abusing opiates and now I am suffering wds. The horrible physical pain has gone, but anxiety has set it in. As the muscle pain eased up, my brain opened the door and let horrible, panic attack level anxiety in instead. Can anyone relate? Don't know. Confused. Scared	0.33	0.12	0.27	0.13	0.005
Sad	I'm tired of losing jobs and missing opportunities. I'm tired of being broke. I feel empty all the time	0.18	0.10	0.14	0.11	0.005
Affect	Clean for 54 days. Things are good. I no longer feel like shit all the time. I'm having trouble accepting and fixing the mistakes I made while in active addiction.Drug dreams are crazy. My finances are completely f***ed, which is terrible	0.41	0.08	0.38	0.10	0.005
Anxiety	So, I'm at 7 days clean. I was abusing opiates and now I am suffering wds. The horrible physical pain has gone, but anxiety has set it in. As the muscle pain eased up, my brain opened the door and let horrible, panic attack level anxiety in instead. Can anyone relate? Don't know. Confused. Scared	0.13	0.09	0.10	0.10	0.005
Swear	God f***ing damn it! F*** today! Today is the shittiest f***ing damn day! These withdrawals have me sick as f***! I feel like I am screwed forever	0.13	0.12	0.11	0.11	0.005
Health	I dosed fentanyl everyday. Now, I'm 1.5–2 days into withdrawal. I am experiencing emotional instability, some stomach ache, and mostly bad flu symptoms. Not vomiting or diarrhea so far. My addiction, like many people’s, is a secret one. I have no one to turn to for help except an anonymous, online forum. Thank you",	0.19	0.10	0.14	0.10	0.005
Biology	I know that I'm not gonna sleep well, but my feet, specifically my heels, are in so much pain right now. Is there anything I can do for this other than Tylenol?	0.32	0.10	0.28	0.12	0.005
Death	These withdrawals are killing me. I feel being dead with no feeling would be much better than this pain	0.04	0.06	0.03	0.06	0.005
Body	Does anyone else get weird eye twitches and spasms in withdrawals One of my first signs, as always, is that one side of my face starts scrunching up around the eye/ear area. Just these weird muscle jerk things. Happens every time. Anyone else ever experience this or know why it happens? I'm really curious	0.19	0.10	0.18	0.12	0.005

Example terms in each category are underlined

In Table 2 we compare the values of the indicators for “*emotional distress*”, and “*physical pain*” between the users who posted or did not post in DAR subreddits. The corresponding table for Empath variables is presented in Additional file 1: Table S1. We used LIWC to determine the value of each indicator for the posts of drug users in our dataset. Then the distributions of the values of indicators for the set of users who posted in a DAR subreddit was compared with the set of users who did not post in a DAR subreddit with the null hypothesis being that there was no difference between the distributions. The Mann–Whitney *U*-test [30], a non- parametric test, was used to compare the distributions and we observe statistically significant differences between the two set of users for each observable variable.

The values of the indicators of the latent variable “*emotional distress”* were found to be higher for the users who displayed addiction recovery behavior. Posts corresponding to addiction recovery behavior typically consisted of higher values for the LIWC categories: “feel” (20%, *p* < 0.005), “anger” (22.2%, *p* < 0.005), “authentic” (9%, *p* < 0.005), “sexual” (13.3%, *p* < 0.005), “negative emotion” (20%, *p* < 0.005), “sad” (25%, *p* < 0.005), “affect” (7.5%, *p* < 0.005), “anxiety” (26.0%, *p* < 0.005), and “swear” (16.6%, *p* < 0.005) as compared to the other LIWC categories used by us (Table 2).

Similarly, the values for the indicators of the latent variable “*physical pain”* were higher for the users who displayed addiction recovery behavior. Accordingly, our data shows that drug users complained about their health and physical discomforts during the withdrawal phase. Correspondingly, these posts were found to have higher values for the relevant LIWC categories: “body” (5.4%, *p* < 0.005), “health” (30.3%, *p* < 0.005), “biology” (13.3%, *p* < 0.005), and “death” (28.5%, *p* < 0.005) (Table 2).

#### Path analysis

**Fig. 2:**
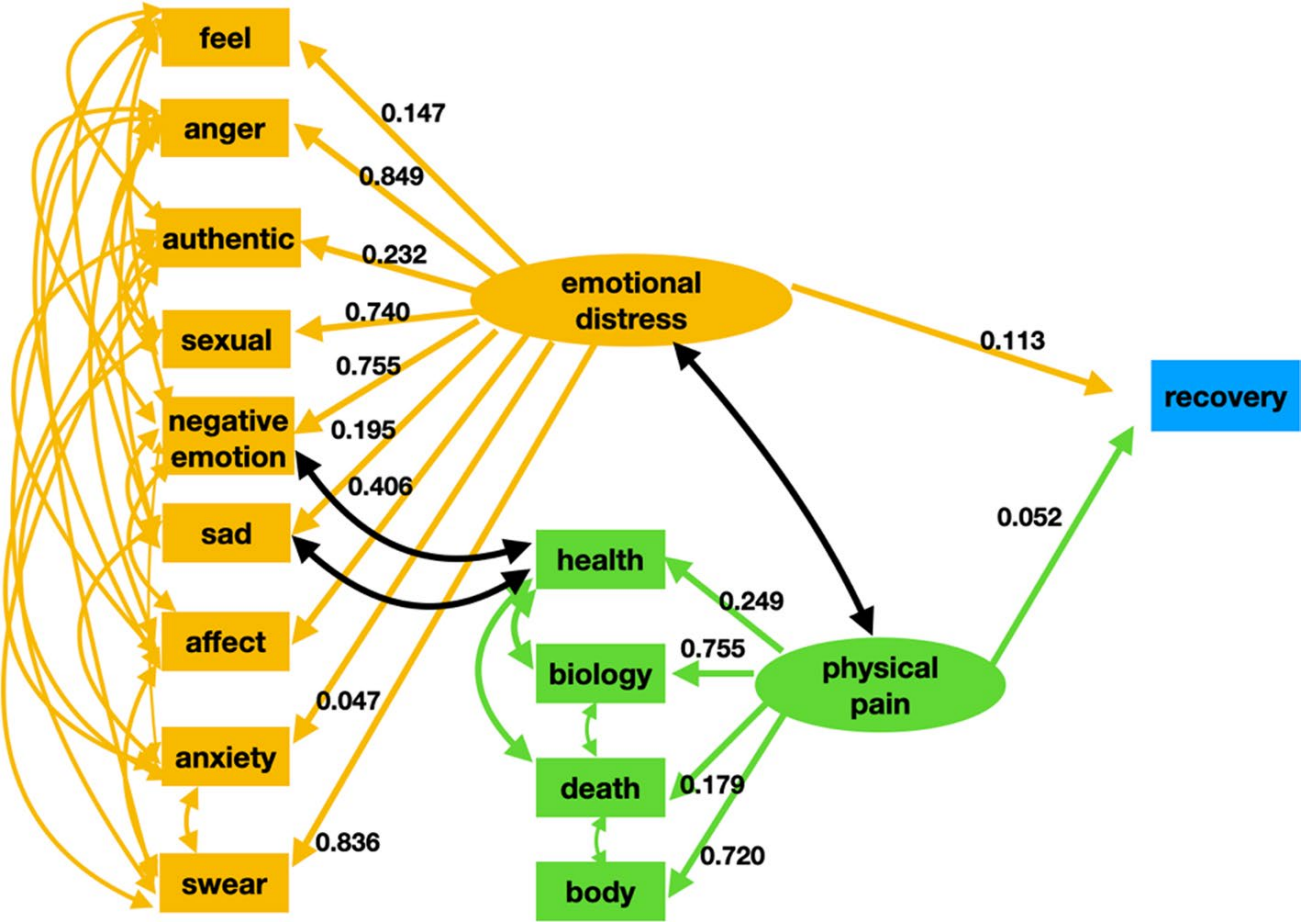
The LIWC withdrawal management model. Ellipses indicate latent variables, rectangles represent observed variables, straight line with one arrowhead represents a direct effect, and a curved line represents covariance. As indicated by this model emotional and physical pain positively affects the recovery propensity of a drug user. However, for the LIWC indicators emotional factors were found to be more important than physical factors

Figure 2 displays the final LIWC withdrawal management model with factor loadings (the value for correlations are not displayed in the figure to maintain clarity). In Fig. 2, the effect of the variables “*emotional distress*” and “*physical pain*” on drug addiction recovery behavior is studied. We estimated the latent variable “*emotional distress*” with nine LIWC categories: “negative emotion”, “sad”, “anger”, “anxiety”, “feel”, “affect”, “swear”, “sexual”, and “authentic”. The latent variable “physical pain” was estimated using four indicators “biology”, “death”, “health”, and “body”. All of the paths in the model were found to be statistically significant. Both “*emotional distress”* and “*physical pain”* were found to influence addiction recovery behavior. However, “*emotional distress”* was found to be more evident in withdrawal as compared to “*physical pain”*; all of the indicator variables for “*emotional pain”* were found to have a strong effect on withdrawal, with the LIWC categories “anger” and “swear” being the two most significant indicators.

RMSEA, SRMR, CFI, and TLI were used to assess the model fit. The results based on the hypothesized model indicated a decent fit with RMSEA = 0.08, TLI = 0.90, CFI = 0.95, and SRMR = 0.07. The relatively higher value observed for the RMSEA was due to the covariance between the LIWC categories. These covariances increased the number of paths that had to be estimated in the model, reduced the degrees of freedom of the model, and led to relatively higher RMSEA values. The values for the TLI, CFI, and SRMR indices all indicate high-quality model fit. Table 3 summarizes the results of the final SEM model.

**Table 3:** Latent variable structure, direct effects, and covariances the final LIWC withdrawal management SEM model

Relationships between variables		Estimate	Standardized estimate	Standard error	Z value	*P* value
Latent variables						
Emotional distress—>	Negative emotion	1.000	0.755	–	–	–
Emotional distress—>	Feel	0.197	0.147	0.017	11.2	0.000
Emotional distress—>	Anger	1.136	0.849	0.019	60.7	0.000
Emotional distress—>	Authentic	0.313	0.232	0.018	17.2	0.000
Emotional distress—>	Sexual	0.998	0.740	0.024	41.6	0.000
Emotional distress—>	Sad	0.262	0.195	0.016	16.3	0.000
Emotional distress—>	Affect	0.547	0.406	0.016	33.9	0.000
Emotional distress—>	Anxiety	0.063	0.047	0.017	3.6	0.000
Emotional distress—>	Swear	1.127	0.836	0.021	52.9	0.000
Physical distress—>	Health	1.000	0.249	–	–	–
Physical distress—>	Bio	3.079	0.755	0.152	20.2	0.000
Physical distress—>	Death	0.731	0.179	0.068	10.7	0.000
Physical distress—>	Body	2.899	0.720	0.171	16.9	0.000
Regressions						
Emotional distress—>	Recovery	0.153	0.113	0.027	5.6	0.000
Physical pain- >	Recovery	0.213	0.052	0.088	2.4	0.000
Correlations						
Negative emotion	Anxiety	0.474	0.744	0.010	47.6	0.000
Negative emotion	Sad	0.366	0.583	0.009	38.8	0.000
Negative emotion	Affect	0.335	0.570	0.011	31.6	0.000
Negative emotion	Anger	0.060	0.179	0.005	11.5	0.000
Negative emotion	Feel	0.107	0.168	0.006	16.5	0.000
Negative emotion	Sexual	− 0.059	− 0.136	0.007	− 8.8	0.000
Negative emotion	Authentic	− 005	− 0.008	0.005	− 1.133	0.257
Sexual	Swear	0.160	0.434	0.012	13.3	0.000
Affect	Anxiety	0.317	0.351	0.011	29.0	0.000
Sad	Affect	0.277	0.311	0.011	25.8	0.000
Health	Bio	0.418	0.669	0.011	37.7	0.00
Authentic	Feel	0.284	0.297	0.011	18.6	0.000
Sad	Anxiety	0.218	0.226	0.012	18.6	0.000
Affect	Feel	0.145	0.162	0.009	15.7	0.000
Affect	Anger	0.114	0.237	0.008	14.7	0.000
Anger	Swear	0.179	0.622	0.008	21.5	0.000
Anxiety	Feel	0.104	0.107	0.010	10.0	0.000
Anger	Sexual	0.090	0.254	0.011	8.0	0.000
Affect	Swear	0.065	0.130	0.007	9.665	0.000
Authentic	Swear	0.017	0.032	0.005	3.682	0.000
Negative emotion	Health	0.182	0.296	0.006	31.6	0.000
Anxiety	Health	0.153	0.162	0.008	18.1	0.000
Sad	Health	0.139	0.49	0.008	16.4	0.000
Feel	Body	0.181	0.270	0.009	20.6	0.000
Anger	Death	0.072	0.139	0.004	16.5	0.000
Emotional distress	Physical pain	0.123	0.675	0.008	15.2	0.000

The symbol ‘—> ’ is used to represent a path or direct effect in our SEM model. Both emotional distress and physical pain positively impacted adiction recovery behavior

### The withdrawal management model obtained using Empath variables

#### Summary statistics

**Fig. 3:**
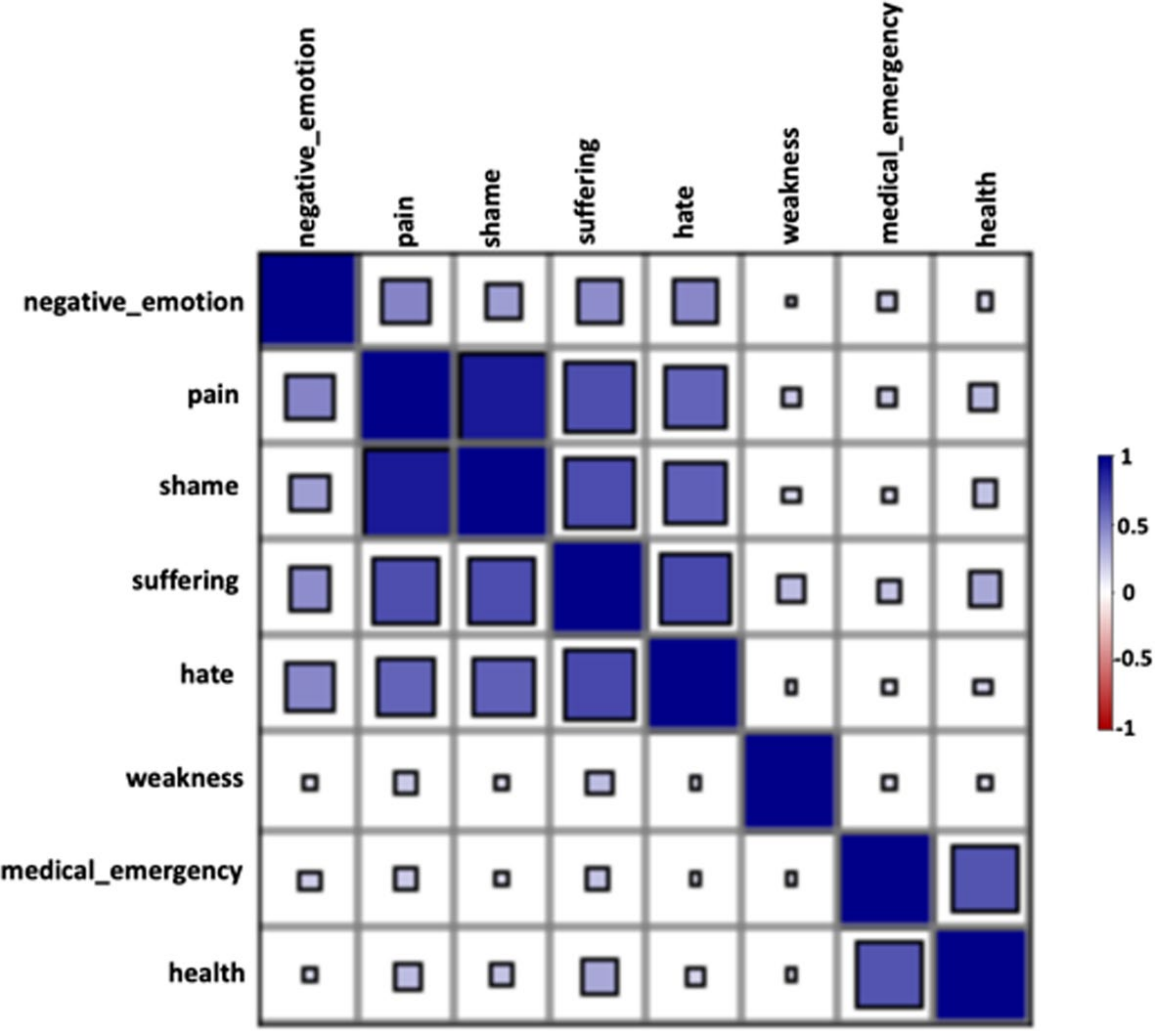
Correlation diagram of the Empath variables present in the withdrawal management model (see also Table 4). Positive correlations are color-coded in blue and negative correlations in red. The size of each square represents the magnitude of the correlations. As this visualization indicates, every variable-pair in the model is positively correlated. The two highest correlation values were observed for the variable-pairs “pain” and “shame” followed by “suffering” and “hate”

In Table 4 and Fig. 3 we present the correlations between the Empath indicators for the withdrawal management model. Similar to the LIWC variables, all of the Empath variables in the model were also found to be positively correlated with each other with the categories “pain” and “shame” (0.89) followed by “suffering” and “hate” (0.71) having the highest correlation values. The Empath category “suffering” was also found to be correlated with “medical_emergency” (0.22), “weakness” (0.25), “health” (0.34), and “pain” (0.69) indicating that users in the withdrawal phase discussed physical symptoms in the context of distress. In Additional file 1: Table S1 we compare the values of the Empath based indicators for “*emotional distress*”, and “*physical pain*” between the users who post and do not post in DAR subreddits.

**Table 4:** Correlation matrix of the Empath variables present in the withdrawal management model

	Medical_emergency	Weakness	Health	Pain	Negative_emotion	Shame	Suffering	Hate
Medical_emergency	1	0.12	0.67	0.19	0.19	0.13	0.22	0.11
Weakness		1	0.11	0.20	0.11	0.14	0.25	0.11
Health			1	0.26	0.14	0.24	0.34	0.16
Pain				1	0.47	0.89	0.69	0.60
negative_emotion					1	0.38	0.43	0.46
Shame						1	0.69	0.62
Suffering							1	0.71
Hate								1

#### Path analysis

**Fig. 4:**
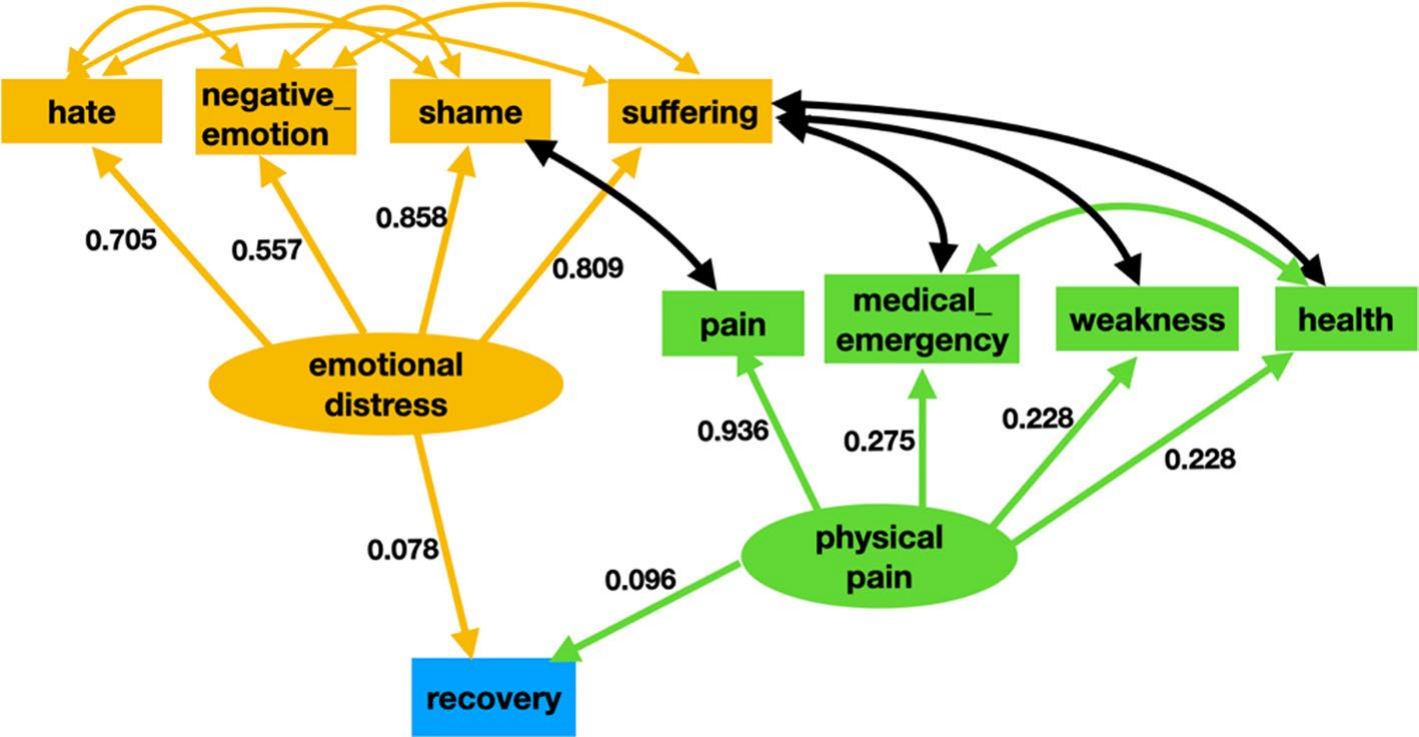
The Empath indicators-based withdrawal management model. Ellipses indicate latent variables, rectangles represent observed variables, straight line with one arrowhead represents a direct effect, and a curved line represents covariance. As indicated by this model, emotional and physical pain were found to positively influence the propensity of a drug user to recover. Unlike the model built using LIWC indicators, for the Empath indicators physical factors were found to be more important than emotional factors in recovery

Figure 4 displays the Empath indicator-based withdrawal management model with factor loadings (the value for correlations are not displayed in the figure to maintain clarity). In this figure, the effect of “*emotional distress*” and “*physical pain*” on drug addiction recovery behavior is studied. We estimated the latent variable “*emotional distress*” with four Empath categories: “negative_emotion”, “hate”, “shame”, and “suffering” The latent variable “physical pain” was estimated using four indicators ““pain”, “medical_emergency”, “weakness”, and “health”. All of the paths in the model were found to be statistically significant. As was the case for the model built using LIWC indicators, both “*emotional distress”* and “*physical pain”* were found to influence addiction recovery behavior. All of the indicators for “*emotional distress*” had a strong positive effect, with “shame” and “suffering” being the most contributory. Similarly, all of the indicators for the “physical pain” had a strong positive effect, with “pain” having the highest effect. As opposed to the LIWC model, however, “*physical pain”* was found to be more evident in withdrawal as compared to “*emotional distress”*. The model quality was determined using RMSEA, SRMR, CFI, and TLI. The hypothesized model indicated a good fit with RMSEA = 0.07, TLI = 0.96, CFI = 0.98, and SRMR = 0.03. Similar to the LIWC model, the relatively higher value observed for the RMSEA was due to the covariance between the Empath categories. The values for the TLI, CFI, and SRMR indices all indicate high-quality model fit. Table 5 summarizes this SEM model.

**Table 5:** Latent variable structure, direct effects, and covariances of the Empath withdrawal management SEM model

Relationships between variables		Estimate	Standardized estimate	Standard error	Z value	*P* value
Latent variables						
Emotional distress—>	Negative_ emotion	1.000	0.557	–	–	–
Emotional distress—>	Weakness	1.539	0.858	0.061	25.04	0.000
Emotional distress—>	Health	1.453	0.809	0.034	42.9	0.000
Emotional distress—>	Pain	1.265	0.705	0.030	42.4	0.000
Physical distress—>	medical_emergency	1.000	0.228	–	–	–
Physical distress—>	Weakness	.996	0.228	0.075	13.2	0.000
Physical distress—>	Health	0.1.206	0.275	0.050	24.0	0.000
Physical distress—>	Pain	4.100	0.936	0.250	16.4	0.000
Regressions						
Emotional distress—>	Recovery	0.140	0.078	0.146	2.2	0.022
Physical pain- >	Recovery	0.422	0.096	0.061	2.9	0.004
Correlations						
Negative_emotion	Suffering	− 0.011	− 0.023	0.016	− 0.702	0.483
Negative_emotion	Hate	0.073	0.123	0.015	4.7	0.000
Medical_emergency	Health	0.608	0.650	0.014	44.9	0.000
Health	Suffering	0.128	0.227	0.008	16.3	0.000
Weakness	Suffering	0.105	0.184	0.008	14.0	0.000
Shame	Hate	0.013	0.036	0.004	3.3	0.257
Suffering	Hate	0.152	0.365	0.018	8.2	0.000
Negative_emotion	Shame	− 0.092	− 0.217	0.005	− 17.7	0.000
Medical_emergency	Suffering	0.070	0.122	0.008	9.2	0.000
Pain	Shame	0.161	0.897	0.024	6.6	0.00
Emotional distress	Physical pain	0.116	0.913	0.008	15.0	0.000

The symbol ‘—>’ is used to represent a path or direct effect in our model. Both emotional distress and physical pain positively impacted adiction recovery behavior

### The recovery efforts model obtained using subreddit activities

#### Analysis of subreddit activities

**Fig. 5:**
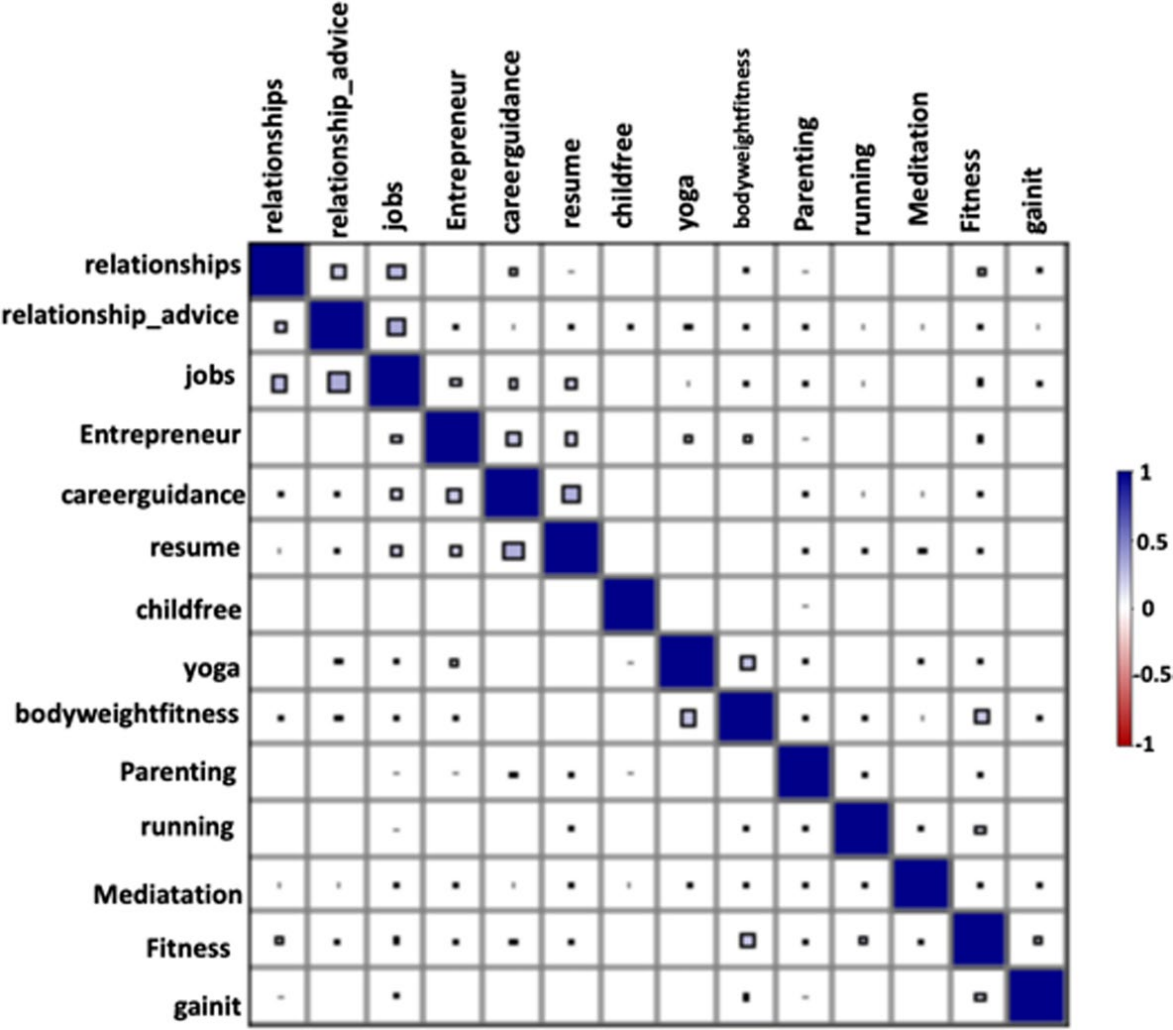
Correlation diagram of the subreddit activities variables present in the recovery model (see also Additional file 1: Table S4). Positive correlations are color-coded in blue and negative correlations in red. The size of each square represents the magnitude of the correlations. As this visualization indicates, every variable-pair in the model is positively correlated. The two highest correlation values were observed for the variable-pairs “career-guidance” and “resume” followed by “career-guidance” and “Entrepreneur”

In Fig. 5 and Additional file 1: Table S4 we present the correlations between the forum activity used in the SEM model for recovery efforts. From the figure and table, we observed that unlike the LIWC variables the correlation values between the forum activity displayed across different subreddits was low. The highest correlation was between the forums “careerguidance” and “resumes” (0.3), followed by “entrepreneur” and “careerguidance” (0.2).

The comparison of the forum activity for the users who posted and did not post in a DAR subreddit was conducted in a manner similar to that described in the withdrawal management model (Table 6). The values of the subreddit activities corresponding to the latent variable “*mental and physical well-being”* were higher for users who displayed addiction recovery behavior. Some of these subreddits were: “fitness” (66.6%, *p* < 0.005), “meditation” (85.7%, *p* < 0.005), “yoga” (85.7%, *p* < 0.005), “gainit” (66.6%, *p* < 0.005), “bodyweightfitness” (100%, *p* < 0.005), and “running” (75.8%, *p* < 0.005) (Table 6). Similarly, the values for the subreddit activities corresponding to the latent variable “*career”* were higher for users who displayed addiction recovery behavior. Some of these subreddits were: “jobs” (96.2%, *p* < 0.005), “entrepreneur” (66.6%, *p* < 0.005), “careerguidance” (66.6%, *p* < 0.005), and “resumes” (66.6%, *p* < 0.005). Finally, the values of the subreddit activities corresponding to the latent variable “*relationships”* were also found to be higher for users who displayed addiction recovery behavior. Examples of subreddits for which enhanced activity was observed included: “relationships” (66.6%, *p* < 0.005), “relationship_advice” (50%, *p* < 0.005), “parenting” (50%, *p* < 0.005), and “childfree” (66.6%, *p* < 0.005) (Table 6).

**Table 6:** Comparison of normalized values for different variables in our model using subreddit activities for people who show and do not show addiction recovery behavior

Subreddit	Description	Individuals displaying signs of addiction recovery		Individuals not displaying signs of addiction recovery		*p* <
		Mean	SD	Mean	SD	
Fitness	Discussion of physical fitness/exercise goals and how they can be achieved	0.006	0.03	0.003	0.02	0.05
Meditation	Experiences, stories, and instructions relating to the practice of meditation	0.005	0.03	0.002	0.02	0.05
Yoga	A place to discuss yoga	0.001	0.02	0.0004	0.006	0.05
Gainit	Fitness subreddit for information and discussion for people looking toput on weight and muscle	0.002	0.03	0.001	0.01	0.05
GetMotivated	This is the subreddit that will help you get up and do what you *know* you need to do. It’s the subreddit to give and receive motivation theorugh pictures videos text, music, and anything that you find motivating	0.003	0.02	0.001	0.02	0.05
Bodyweightfitness	Bodyweightfitness is for redditors who like to use their own body to train	0.003	0.03	0.001	0.02	0.05
Running	All runners welcome	0.002	0.02	0.0009	0.01	0.05
Getdisciplined	A subreddit for people who have problems with procrastination, and discipline. It is a great place to gather and meet others with a similar interest and meet your goals	0.0007	0.01	0.0001	0.00	0.05
Relationship_advice	Need help with you relationship? Whether it’s romance, friendship, family, coworkers, or basic human interaction: we’re here to help	0.004	0.03	0.001	0.01	0.05
Relationships	/r/Relationships is a community built around helping people, and the goal of providing a platform for interpersonal relationship advice between redditors. We seek posts from users who have specific and personal relationship quandaries that other redditors can help them try to solve	0.006	0.03	0.003	0.02	0.05
Parenting	/r/Parenting is the place to discuss the ins and out as well as ups and downs of child-rearing. From the early stages of pregnancy to when your teenagers are finally ready to leave the nest (even if they don’t want to) we’re here to help you through this crazy thing called parenting. You can get advice on potty training, talk about breastfeeding, discuss how to get your baby to sleep or ask if that one weird thing your kid does is normal	0.005	0.04	0.003	0.02	0.05
Childfree	Discussion and links of interest to childfree individuals. “Childfree” refers to those who do not have and do not ever want children (whether biological, adopted, or otherwise)	0.002	0.03	0.001	0.01	0.05
Jobs	How to get work and how to leave it. Employment, recruitment, interviews, etc	0.002	0.02	0.0007	0.007	0.05
Entrepreneur	A community of individuals who seek to solve problems, network professionally, collaborate on projects and make the world a better place. Be professional, humble, and open to new ideas	0.002	0.01	0.001	0.02	0.05
Careerguidance	A place to discuss career options, to ask questions and give advice!	0.001	0.02	0.0005	0.01	0.05
Resumes	Post your résumé for critique, critique someone else’s, or look for examples of résumés in your field	0.002	0.02	0.001	0.02	0.05

Again, we observe that users displaying addiction recovery behavior have higher forum activity for the chosen forums

#### Path analysis

**Fig. 6:**
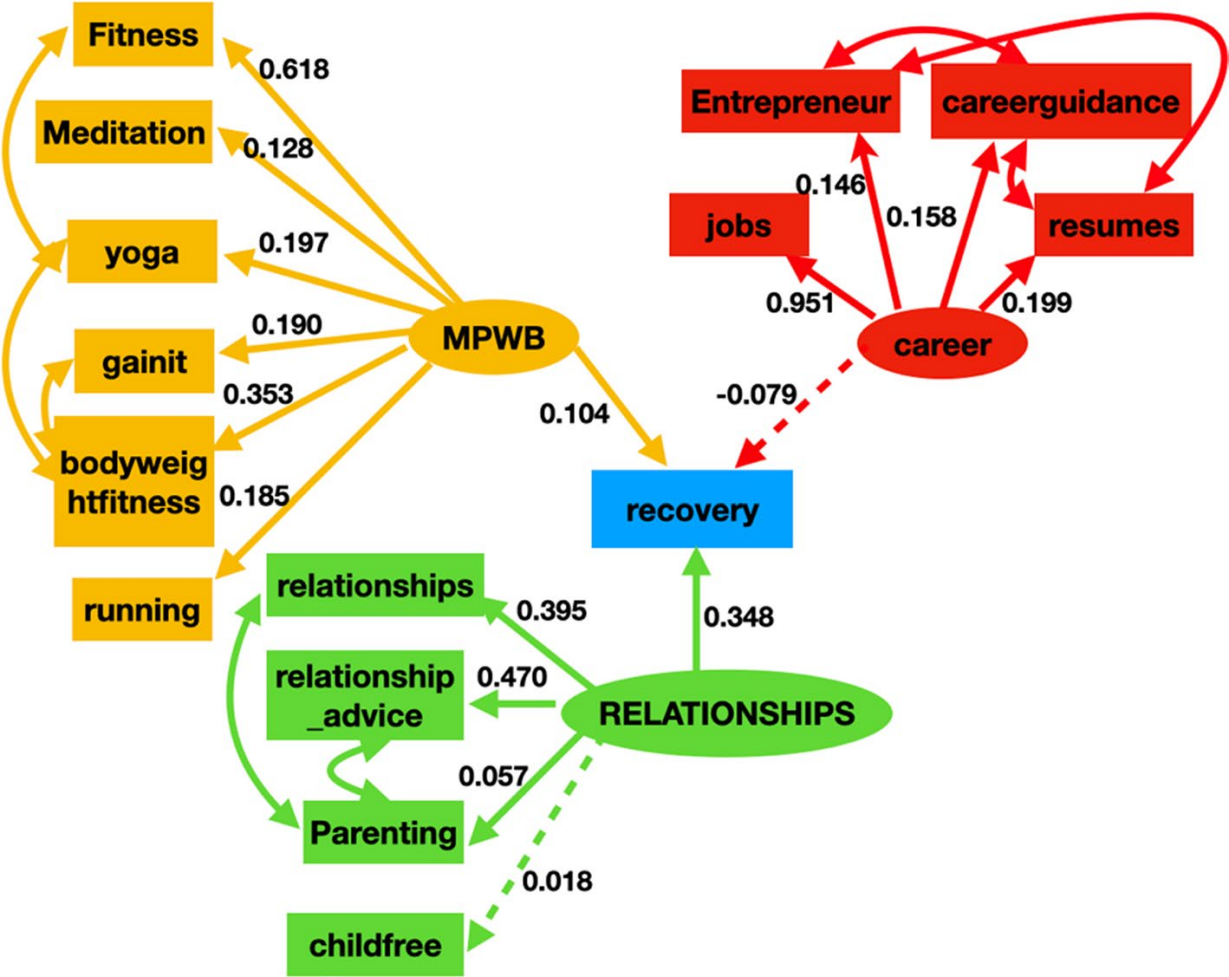
The SEM model for addiction recovery using subreddit activities. Mental and physical well-being (MPWB) and relationships were found to positively influence addiction recovery behavior. Career/job prospects negatively effects recovery behavior, however, its effect was statistically insignificant

Figure 6 shows the subreddit activity-based recovery model with factor loadings (the value for correlations are not displayed in the figure to maintain clarity). In it, the effect of “*mental and physical well-being*”, “*career*” and “*relationships*” on drug addiction recovery behavior is studied. We estimated the latent variable “*mental and physical well-being*” with six indicators: “fitness”, “meditation”, “yoga”, “gainit”, “bodyweightfitness”, and “running”. The latent variable “*career*” was estimated using four indicators “jobs”, “entrepreneur”, “careerguidance”, “resumes”. Finally, the latent variable “relationships” was estimated using the following four indicators: “relationship_advice”, “relationships”, “parenting”, and “childfree”. The effect of “*mental and physical well-being”* and “*relationships”* on addiction recovery behavior was found to be statistically significant and positive, whereas, the effect of “career” on addiction recovery behavior was negative and statistically insignificant. All of the indicator variables for “*mental and physical well-being”* had a strong positive effect*,* with “fitness” and “bodyweightfitness” being the most contributory. Similarly, the indicator variables for “*relationships”* also had a strong positive effect on “*relationships*” (except “childfree” which was statistically insignificant). “relationship_advice” had highest effect on “*relationships*” followed by the subreddit “relationships”. Between “relationships”, and “mental and physical well-being”, “relationships” was found to be more important for addiction recovery behavior. The fit indices for the final model indicated a good fit with the fit indices being: RMSEA = 0.02, TLI = 0.90, CFI = 0.92, and SRMR = 0.02. Table 7 summarizes the SEM model.

**Table 7:** Latent variable factor structure, direct effects, and covariances the final subreddit activity based recovery SEM model

Relationships between variables		Estimate	Standardized estimate	Standard error	Z value	*P* value
Latent variables						
MPWB—>	Fitness	1.000	0.618	–	–	–
MPWB—>	Meditation	0.208	0.128	0.031	6.65	0.000
MPWB—>	Bodyweightfitness	0.571	0.353	0.052	10.7	0.000
MPWB—>	Running	0.300	0.185	0.035	8.5	0.000
MPWB—>	Yoga	0.319	0.197	0.056	5.6	0.000
MPWB—>	Gainit	0.307	0.190	0.035	8.6	0.000
Career—>	Jobs	1.000	0.951	–	–	–
Career—>	Entrepreneur	0.154	0.146	0.021	7.3	0.000
Career—>	Careerguidance	0.166	0.158	0.022	7.5	0.000
Career—>	Resumes	0.209	0.199	0.026	8.1	0.000
Relationships—>	Relationship_advice	1.000	0.470	–	–	–
Relationships—>	Relationships	0.842	0.395	0.043	19.6	0.000
Relationships—>	Parenting	0.121	0.057	0.033	3.6	0.000
Relationships—>	Childfree	0.039	0.018	0.033	1.1	0.235
Regressions						
MPWB—>	Recovery	0.348	0.163	0.104	3.3	0.001
Relationships—>	Recovery	0.104	0.064	0.036	2.8	0.004
Career—>	Recovery	− 0.079	− 0.075	0.040	− 1.9	0.050
Covariance						
Careerguidance	Resumes	0.274	0.283	0.011	21.9	0.000
Yoga	Bodyweightfitness	0.170	0.185	0.016	10.3	0.000
Entrepreneur	Careerguidance	0.191	0.195	0.012	15.7	0.000
Entrepreneur	Resumes	0.150	0.154	0.012	12.3	0.000
Fitness	Yoga	− 0.095	− 0.123	0.022	− 4.4	0.000
Relationships	MPWB	0.097	0.335	0.009	10.2	0.000
Relationships	Career	0.316	0.709	0.012	25.5	0.000
MPWB	Career	0.099	0.168	0.011	8.9	0.000

‘—> ’ represents a path or direct effect in the model. “Relationships” have a positive impact on addiction recovery. “Mental and physical well-being” (MPWB) also has a positive impacton addiction recovery. But, the impact of “career” was negative and statistically insignificant

### Relapse modeling using LIWC

#### Summary statistics

**Fig. 7:**
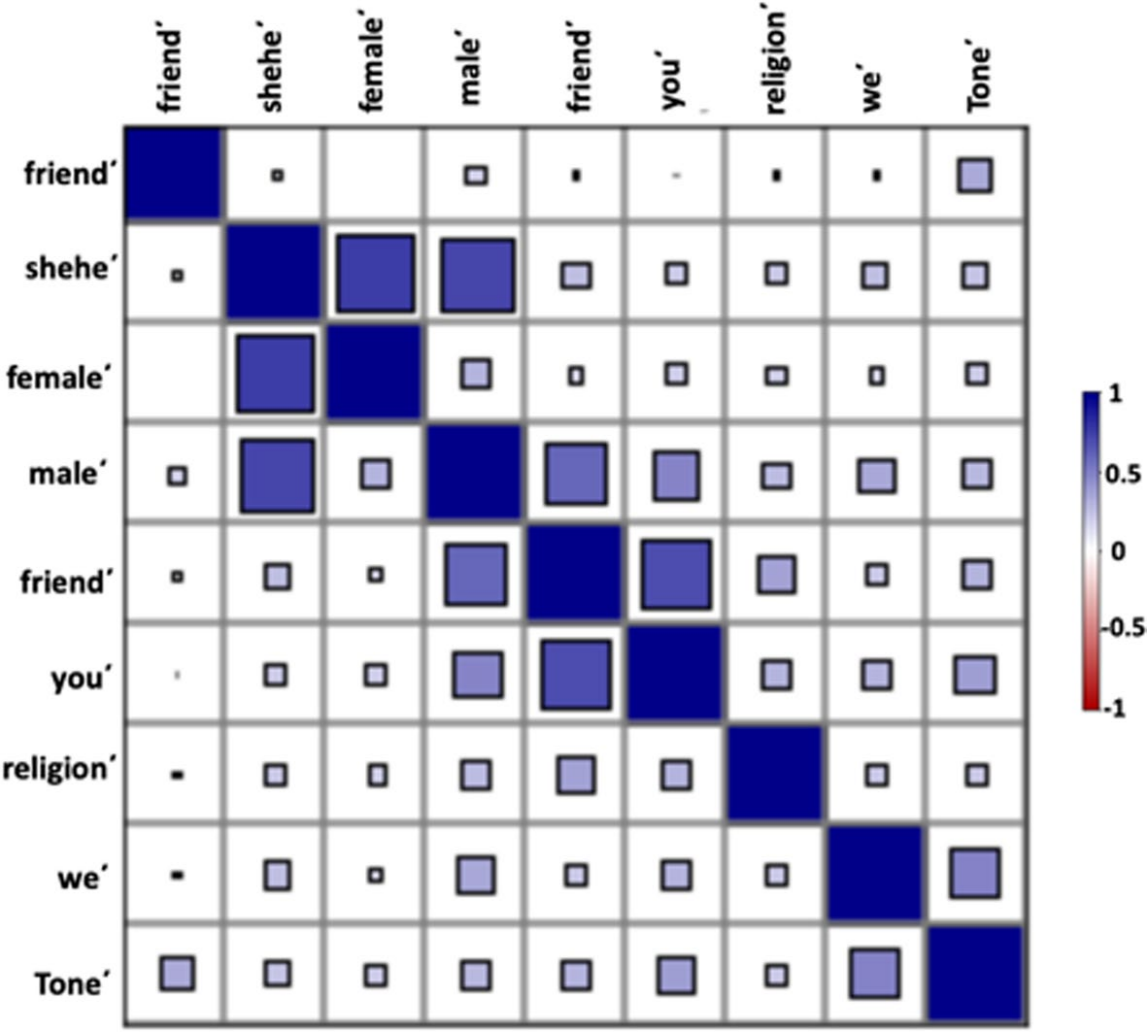
Correlation diagram of the LIWC variables present in the LIWC relapse model (see also Table 8). Positive correlations are color-coded in blue and negative correlations in red. The size of each square represents the magnitude of the correlations. As this visualization indicates, every variable-pair in the model is positively correlated. The two highest correlation values were observed for the variable-pairs “you΄” and “female΄” followed by “you΄” and “male΄”

In Table 8 and Fig. 7 we present the correlations observed between the LIWC indicators in the relapse model. All of the LIWC variables were found to be positively correlated with each other with the highest correlation observed for the categories “you΄” and “female΄” (0.76) followed by “you΄” and “male΄” (0.72). In Additional file 1: Table S3 we compare the values of the LIWC based indicators for “*anti-social*”, “*motion*΄*” (lack of physical activity), and “religion*΄” (lack of religious) between the users who relapse and who do not relapse.

**Table 8:** Correlation matrix of the LIWC variables present in the LIWC relapse model

	Friend΄	We΄	Shehe΄	You΄	Male΄	Female΄	Tone΄	Motion΄	Religion΄
Friend΄	1	0.20	0.25	0.69	0.59	0.13	0.29	0.06	0.3
We΄		1	0.25	0.29	0.34	0.13	0.47	0.05	0.20
Shehe΄			1	0.19	0.72	0.76	0.22	0.08	0.20
You΄				1	0.47	0.19	0.38	0.02	0.29
Male΄					1	0.29	0.28	0.17	0.25
Female΄						1	0.19	0.00	0.18
Tone΄							1	0.33	0.20
Motion΄								1	0.05
Religion΄									1

#### Path analysis

**Fig. 8:**
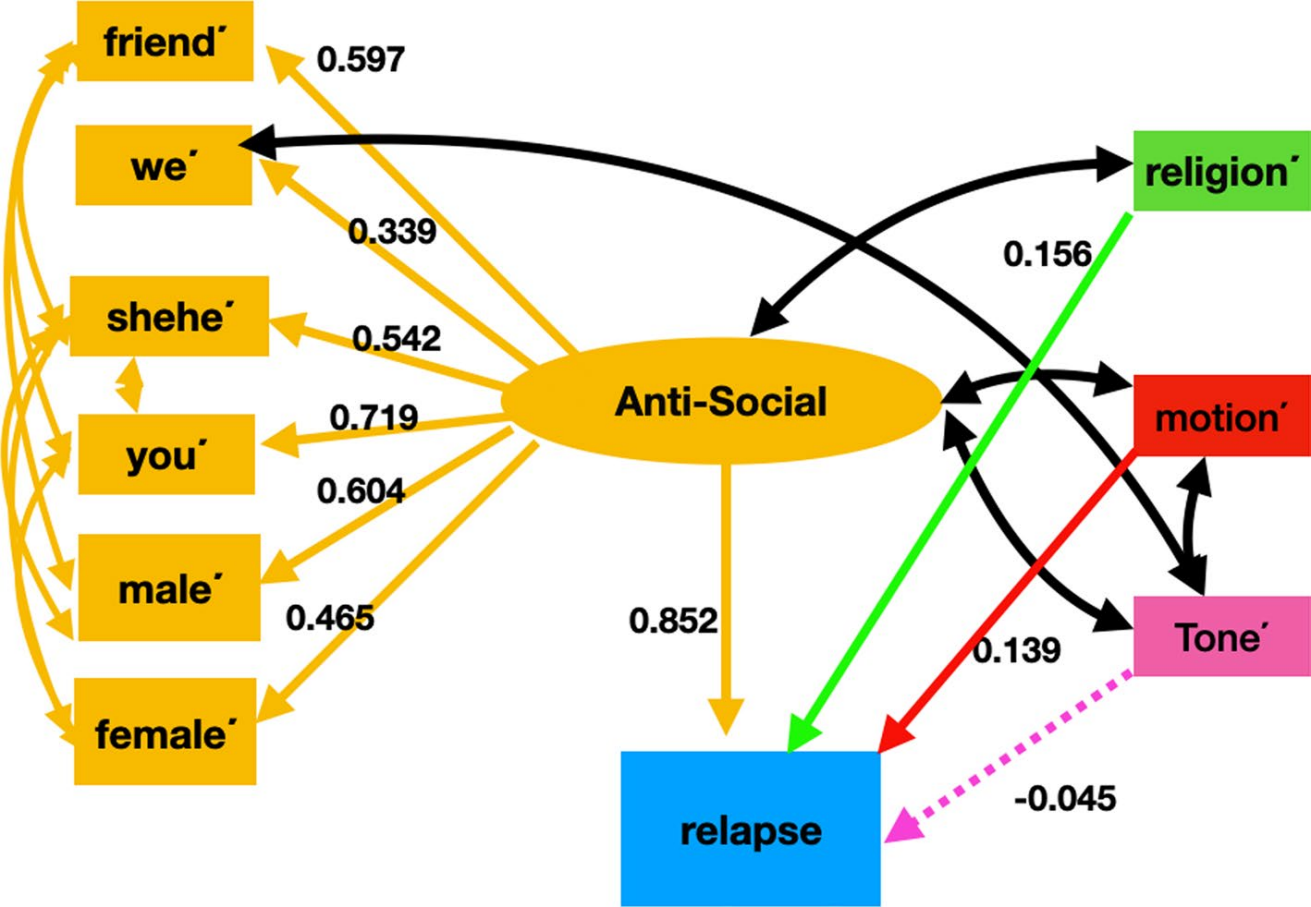
Final model of factors for the LIWC relapse model. “*Anti-Social*”, “*religion΄*”, and “*motion΄*” were found to positively influence relapse behavior. Tone΄ negatively affected relapse behavior, however, its effect was statistically insignificant

Figure 8 shows the final LIWC based relapse model with factor loadings (the value for correlations are not displayed in the figure to maintain clarity). In this figure, the effect of “*anti-social”*, “*motion*΄*” (lack of physical activity), and “religion*΄” (lack of religious) on relapse behavior is studied. We estimated the latent variable “*anti-social*” using the negation of the following six LIWC categories: “friend”, “we”, “shehe”, “you”, “male”, “female”. The effect of “*anti-social”* and the negation variables “*motion*΄*”, and “religion*΄” were found to increase relapse behavior and were statistically significant. The effect of the negation variable “tone΄” (lack of positive emotion) on recovery was negative and statistically insignificant. All of the indicator variables for “*anti-social”* had a strong positive effect*,* with “you΄” and “male΄” being the most contributory. “*Anti-social*” was found to have the highest effect on the relapse behavior. The fit indices for the final model indicated a good fit with the fit indices being: RMSEA = 0.07, TLI = 0.96, CFI = 0.98, and SRMR = 0.03. Table 9 summarizes the model.

**Table 9:** Latent variable structure, direct effects, and covariances of the LIWC-based SEM model for relapse

Relationships between variables		Estimate	Standardized estimate	Standard error	Z value	*P* value
Latent variables						
Anti-social—>	Friend΄	1.000	0.597	–	–	–
Anti-social—>	We΄	0.569	0.339	0.147	3.8	0.000
Anti-social—>	Shehe΄	0.897	0.542	0.152	5.9	0.000
Anti-social—>	You΄	1.194	0.719	0.126	9.4	0.000
Anti-social—>	Male΄	1.015	0.604	0.129	7.8	0.000
Anti-social—>	Female΄	0.785	0.465	0.151	5.1	0.000
Regressions						
Anti-social—>	Relapse	1.402	0.852	0.235	5.9	0.000
Religion΄—>	Relapse	0.151	0.156	0.063	2.3	0.016
Motion΄—>	Relapse	0.135	0.139	0.055	2.4	0.014
Tone΄—>	Relapse	− 0.044	− 0.045	0.077	− 0.573	− 0.573
Correlations						
Shehe΄	Female΄	0.467	0.652	0.057	8.1	0.000
Friend΄	You΄	0.230	0.431	0.052	4.4	0.000
You΄	Female΄	− 0.090	− 0.152	0.047	− 1.9	0.057
Friend΄	Shehe΄	0.006	0.010	0.037	0.1	0.863
Shehe΄	You΄	− 0.166	− 0.301	0.035	− 4.7	0.000
Shehe΄	Male΄	0.395	0.614	0.053	7.4	0.000
Friend΄	Male΄	0.210	0.338	0.048	4.3	0.000
Anti-social΄	Tone΄	0.257	0.440	0.060	4.2	0.000
Anti-social΄	Religion΄	0.193	0.330	0.054	3.5	0.000
Motion΄	Tone΄	0.321	0.324	0.076	4.2	0.000
Anti-social	Motion΄	0.043	0.073	0.052	0.8	0.409
We΄	Tone΄	0.300	0.325	0.069	4.3	0.000

The symbol ‘—> ’ is used to represent a path or direct effect in our SEM model. The negation of a variable is indicated by a prime. “*Anti-social*”, “*motion΄*”, and “*religion΄*” had a positive impact on relapse behavior

### Relapse modeling using Empath

#### Summary statistics

**Fig. 9:**
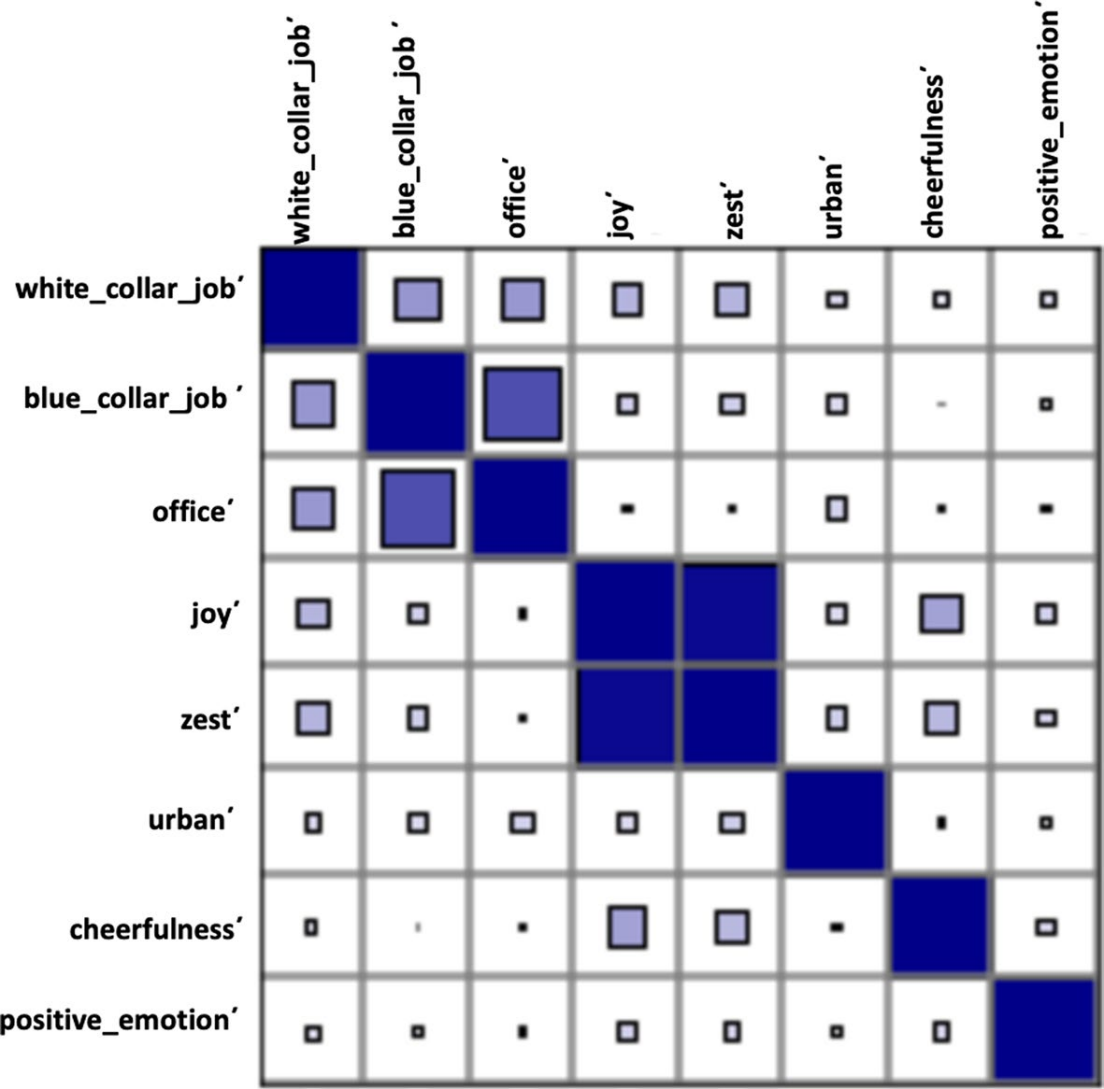
Correlation diagram of the Empath variables present in the Empath relapse model (see also Table 10). Positive correlations are color-coded in blue and negative correlations in red. The size of each square represents the magnitude of the correlations. As this visualization indicates, every variable-pair in the model was positively correlated. The two highest correlation values were observed for the variable-pairs “joy΄” and “zest΄” followed by “white_collar_job΄” and “blue_collar_job΄”

In Additional file 1: Table S2 we compare the values of the Empath based indicators for the negation variables “*positive emotion*΄” (lack of positive emotion), “*career*΄*” (lack of career interests), and “urban*΄” (lack of urban facilities) between the users who relapse and who do not. In Table 10 and Fig. 9 we present the correlations between the Empath indicators present in the relapse model. Similar to the LIWC variables, all of the Empath variables in the model were found to be positively correlated with each other with the categories “joy΄” and “zest΄” (0.95) followed by “white_collar_job΄” and “blue_collar_job΄” (0.69) having the highest correlation values.

**Table 10:** Correlation matrix of the Empath variables present in the Empath relapse model

	Joy΄	Zest΄	Cheerfulness΄	Positive_emotion΄	Office΄	White_collar_job΄	Blue_collar_job΄	Urban΄
Joy΄	1	0.95	0.36	0.17	0.29	0.18	0.07	0.14
Zest΄		1	0.27	0.16	0.29	0.18	0.06	0.18
Cheerfulness΄			1	0.16	0.08	0.02	0.04	0.06
Positive_emotion΄				1	0.12	0.10	0.07	0.09
Office΄					1	0.40	0.39	0.15
White_collar_job						1	0.69	0.14
Blue_collar_job΄							1	0.17
Urban΄								1

#### Path analysis

**Fig. 10:**
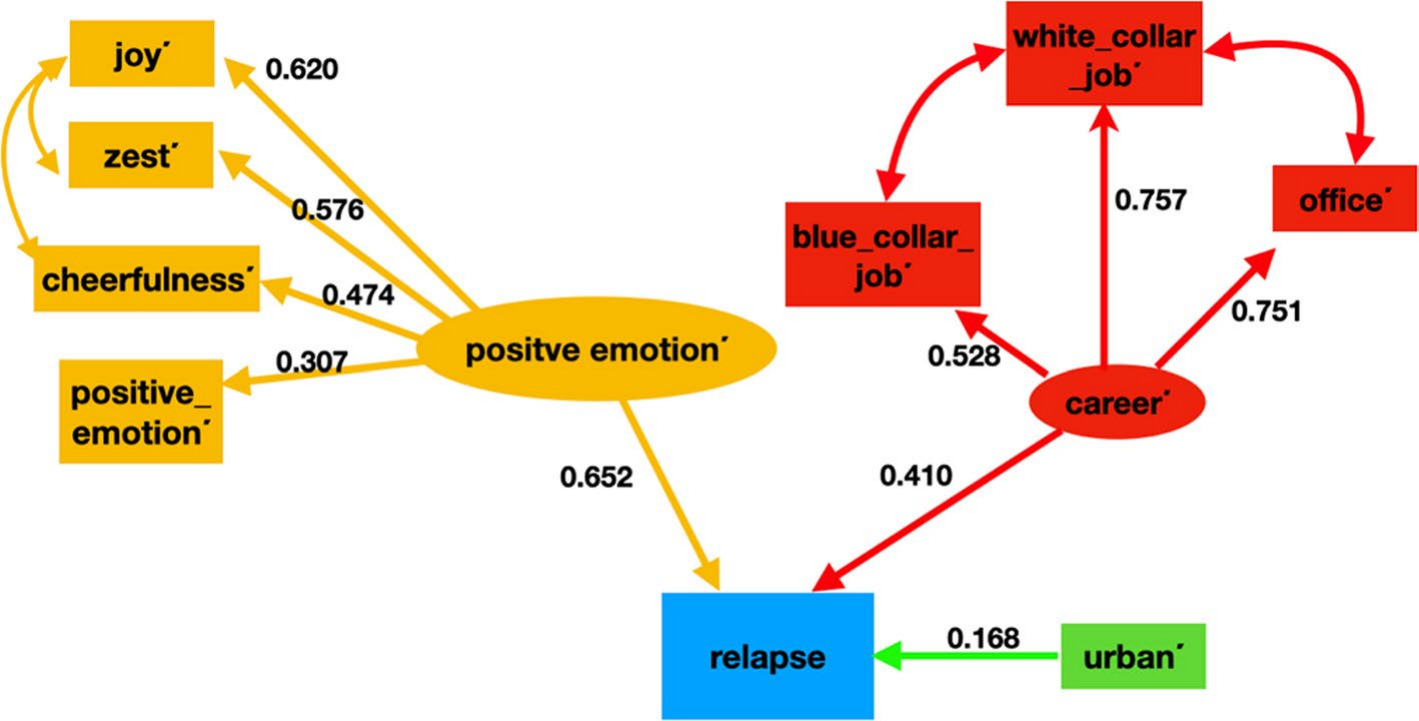
The Empath indicator-based relapse model. Ellipses indicate latent variables, rectangles represent observed variables, straight line with one arrowhead represents a direct effect, and a curved line represents covariance. As indicated by this model, “*positive emotion΄*”, “*career΄*”, and “*urban΄*” were found to positively influence the relapse behavior of a drug user

Figure 10 displays the Empath indicator-based relapse model with factor loadings (the value for correlations are not displayed in the figure to maintain clarity). In this figure, the effect of “*positive emotion*΄”, “*career*΄” and “*urban*΄” on relapse behavior is shown. We estimated the latent variable “*positive emotion*΄” with the negation of the following Empath indicators: “joy”, “zest”, “cheerfulness”, and “positive emotion”. The latent variable “career” was estimated using the negation of three Empath indicators: “blue_collar_job”, “white_collar_job”, and “office”. All of the path models were found to be statistically significant. The effect of “*positive emotion΄*”, “*career*΄”, and “*urban*΄” were found to be lead to relapse and were statistically significant. The indicator variables for “*positive emotion*΄*”* were found to have a strong effect*,* with “joy΄΄” and “zest΄΄” being the most contributory. Similarly, all of the indicators for “*career΄*” also had a strong effect, with “white_collar_job΄” and “office΄” being the most contributory. The fit indices indicated a good fit for this model: RMSEA = 0.04, TLI = 0.98, CFI = 0.99, and SRMR = 0.07. This model is summarized in Table 11.

**Table 11:** Latent variable structure, direct effects, and covariances the Empath relapse SEM model

Relationships between variables		Estimate	Standardized estimate	Standard error	Z value	*P* value
Latent variables						
Positive emotion*΄*—>	Emotional	1.000	0.527	–	–	–
Positive emotion*΄*—>	Suffering	1.196	0.630	0.260	4.5	0.000
Positive emotion*΄*—>	Swearing_terms	1.231	0.649	0.268	4.6	0.000
Career*΄*—>	White_collar_job	1.000	0.995	–	–	–
Career*΄*—>	Blue_collar_job	0.951	0.946	0.088	10.8	0.000
Career*΄*—>	office	0.134	0.133	0.077	1.7	0.082
Regressions						
Positive emotion*΄*—>	Relapse	0.694	0.372	0.204	3.3	0.001
Career*΄*—>	Relapse	0.100	0.101	0.075	1.3	0.183
Urban*΄*—>	Relapse	0.144	0.147	0.068	2.1	0.034
Covaraiances						
Joy*΄*	Zest*΄*	0.597	0.937	0.092	6.5	0.000
Joy*΄*	Cheerfulness	0.066	0.096	0.022	3.0	0.002
White_collar_job*΄*	Blue_collar_job	0.290	0.526	0.085	3.4	0.001
White_collar_job*΄*	Office*΄*	− 0.160	− 0.374	0.079	− 2.036	0.042
Positive_emotion*΄*	Career*΄*	0.198	0.424	0.062	3.2	0.001

The symbol ‘—> ’ is used to represent a path or direct effect in our SEM model. Emotional distress, career, rural, and weakness positively impacted relapse behavior, but the impact of career was statistically insignificant

## Discussions

### The role of emotional distress and physical pain in withdrawal management

We observed that both emotional distress and physical pain played a significant role for redditors who display addiction recovery and relapse related behavior. To understand the reason behind this observation we further investigated the posts from individuals discussing their withdrawals from drugs. We observed that users typically experienced both physical pain and emotional distress during withdrawal. Also, we often observed users to have employed chemical treatments such as methadone and suboxone, alternative therapies such as kratom, xanax, and loperamide, as well as other supplements known to suppress physical symptoms of withdrawal. Interventions for assuaging emotional distress were found by us to be less prevalent. In Table 12 we present example posts describing some of the measures taken by individuals to suppress physical pain and discomfort. Interestingly, many users who had successfully managed their withdrawal process and were well into recovery, were observed by us to display a sense of loss after giving up their drug of choice. Paraphrased examples of posts describing such behavior are shown in Table 13.

**Table 12:** Paraphrased posts discussing different therapies utilized by the drug users to suppress physical discomforts during withdrawals

I have experienced the withdrawals millions of time. I have a routine to get through it and I am going to share it with you. You need kratom, Xanax, restless legs tablets, vitamin C, and easy to eat food like ypgurt and bananas. Kratom is required for the first five day Take Xanax, and vitamins whenever you feel sick. Smoke pot whenever you feel like getting high	
Please review my opioid taper plan and let me know if I am missing anything. Open to suggestions. Day 1–2: 120 mg in the morning, 120 mg in the night. Day 3–4: 100 mg in the morning, 100 mg in the night. Day 5–6: 80 mg in the morning, 80 mg in the night. Day 7–8: 60 mg in the morning, 60 mg in the night. Day 9–10: 40 mg in the morning, 40 mg in the night. Day 11–12 20 mg in the morning, 20 mg in the night, and finally bring it to down to 5–10 mg a day and then call it quits

**Table 13:** Example paraphrased posts displaying drug craving and emotional distress for drug users in addiction recovery

I’ve been clean for 4 months, longest it’s ever been. I’m happy and I have my family and loving partner. We have so much fun together and I’m starting to work again, biking, seeing a number of therapists, doctors, group therapy. Life can’t be better is good. But, I’m bored because nothing in life gives me the rush and excitement that drugs did. I'm worried I will. I dont want to fail again because I know it won’t be just once. It never has been. I dont want to feel guilty after. What am I supposed to do to stop this? Is this forever? Am I never going to be 100% satisfied with life after experiencing the highs of drugs?	
The cravings go so far beyond just managing our greed. The disease is so far beyond what others can comprehend and I hate how people try to just tell me to stop covering my emotions. I have 74 days clean and crave whether I’m happy or sad	

### Mental and physical well-being

Both mental and physical well-being were found to have a positive effect of addiction recover behavior. Physical activities are known to increase the production dopamine, noradrenaline, and serotonin and can act as mechanisms for a natural high [31–39]. Many initiatives such as “lace- ‘em-up” have demonstrated the importance of physical activity for recovering addicts [40]. Our work confirms that similar conclusions can be drawn by analyzing social media data. In Table 14 we display paraphrased excerpts from posts demonstrating the positive effects of mental and physical activities on addiction recovery behavior.

**Table 14:** Example paraphrased posts displaying participation of users in differen mental and physical well being activities while inaddiction recovery

I have been clean for 2 years now. I journal my improvements and small victories and make sure I do fun things like riding my bike, and exercising. I also meditate, and do yoga. Even though occasionally I’ll get cravings, there’s no way in hell I would trade my life today to go back to addiction	
I started with a simple routine of a morning walk. That was it. Now I am into lifting weights, stretching, yoga, and meditation. If anyone wants to learn more about these things there are many videos on YouTube. Look for something low impact to start off with and don’t push yourself too hard. Remember baby steps	

### Relationships

We found that “*relationships*” had a positive effect on addiction recovery. Unsurprisingly, friends and family play an important role in the addiction recovery efforts of an individual. There are many reasons that underlie this finding. First, the stigma associated with drug use causes an individual to feel shame and fear discrimination. Consequently, they don’t feel safe to discuss their issues with co-workers, or strangers. It has been shown that addicts and recovering addicts feel comfortable in sharing their addictions and recovery journey with friends and family [41]. Research has also highlighted the willingness and positive outcomes of users undergoing addiction recovery efforts with the help and support drug-free friends, family members, and significant others [42]. Our analysis of social media data led to similar conclusions. In Table 15 we share excerpts from posts depicting the different ways friends and family affect the addiction recovery behavior.

**Table 15:** Example paraphrased posts displaying the role of family and friends in addiction recovery

I’m grateful for my relationships. My children, my husband, and my dog were like a rock to me during my struggles. I hope everyone finds such a supporting family. I owe my sobriety to them	
I felt really guilty to directly tell my father. He has already done a lot for me. So I asked my best friend to contact him and let him know that I relapsed. Both of them are coming over and taking me to an addiction specialist tomorrow	

### Jobs and career

We observed a negative, albeit statistically insignificant, effect of career/job opportunities on addiction recovery behavior. As noted in the “Research design and methods” section, the addiction literature is ambiguous on the effect of profession on addiction recovery. To highlight this point, we present example posts showing both the negative and positive aspects of profession on addiction recovery in Table 16.

**Table 16:** Example paraphrased posts discussing positive and negative impacts of focusing of career during addiction recovery

I can’t believe I relapsed again. My job as a selling cars causes me so much stress. I can feel my customers hating me when I talk to them. I have to work extremely hard to earn and it’s exhausting. I have to sell cars to earn and when I don’t I go straight back to cocaine	
Hey everyone. How do you guys handle a high pressure career in recovery, particularly early recovery. I’ve seen fellow redditors who are in the corporate grind. I work a Wall Street job, with unpredictable and stressful hours. I am 10 days clean now, but the timing and pressure keeps on triggering me to use again. If anyone has any experience they can share, would be much appreciated. It’s an extremely well paying job and I don’t want to just walk away from it. Thanks guys	
Tomorrow will be day 10 from snorting dope and honestly it’s been great! I also got a 2nd full time job at night last month so which keeps me busy and helps me sustain myself. Feels great to have some money for once! I don't know why but this feels like the time it will actually work out	
I cleaned up about 3 years ago entirely on my own will power. I found my calling—my dream job. It helped me stay busy and get over my cravings. The enjoyment I felt moving forward in my career was so much more enthralling than getting high off any other drug	

### Supporting addiction recovery and personalized addiction recovery care

Personalized addiction recovery treatments have been found to be essential for successful abstinence [43, 44]. Our results identifying the impact of family and friends, self-development efforts, emotional distress and physical pain on addiction recovery can be utilized to provide direction for a person’s recovery. For example, an individual in the initial stages of abstinence may be asked to focus on mental and physical well-being, and at least for some time stay away from high pressure situations (new jobs or returning to a previous stressful job). Their family and friends could also be made aware about their role in an individual’s recovery and how they provide a safe non-judgmental space for the afflicted individual. Additionally, efforts could be made to manage emotional pains and cravings during and after the withdrawal period.

## Conclusions

In this paper, we have described a framework that uses SEM to analyze and quantify latent constructs using SEM for modelling addiction recovery behavior using data from social media. The paper presents different SEM models to quantify the relationship between a number of observable and latent variables and their link to substance addiction.

To the best of our knowledge, this is the first study to utilize social media data and SEM to measure the latent constructs associated with substance abuse and recovery. Our results underscore the value of information present on social media platforms like Reddit to the study of substance misuse and design of interventions.

## Research design and methods

### Data source and participants

We used a set of 117 recreational drug use (RDU) subreddits, and 29 drug addiction recovery (DAR) subreddits reported in our prior works to identify users discussing drug use and recovery on Reddit [20, 45]. In [20] we had utilized the word2vec algorithm [46] to create a term embedding space. In this space related terms were grouped using an iterative set expansion technique to construct drug-use and addiction-recovery lexicons. These lexicons were subsequently employed to characterize the different subreddits following which bi-clustering was used to cluster the different RDU and DAR subreddits. These bi-clusters were further manually curated to arrive at two RDU, and DAR subreddits sets. For this paper, we further identified 170,097 unique users discussing their drug use and recovery from these two RDU and DAR subreddit sets. For each of these users we retrieved their 1000 most recent posts (the specific number of retrieved posts was platform imposed) using the praw api [47]. Finally, we filtered out those users who had less than five nonempty posts in the RDU and DAR subreddit sets. As a consequence of this filtering, we ended up with a set of 7025 users consisting of 2679 users who posted in both RDU and DAR subreddit, and 4346 users who posted only in an RDU subreddit. In Table 17 we present example posts in different RDU and DAR subreddits.

**Table 17:** Example Reddit posts from the recreation and addiction recovery forums

Subreddit	Paraphrased example posts
Opiates (RDU)	As an addict I feel my entire life is a lie. I can never share with other people that I love to stay in motel rooms and shoot heroin and cocaine together. I am constantly fantasizing about my next shot and I don’t relate to sober people anymore
Benzodiazepines (RDU)	My mom was recently prescribed some diazepam (bullet pills) and I like popping valium once in a while. I am thinking of stealing some from her stash. I hope she doesn’t notice
Trees (weed RDU)	My favorite is the hippie speedball. I love waking up to coffee and smoking a fat blunt, eating breakfast and then smoking another not so fat blunt
OpiatesRecovery (DAR)	I can’t believe it, but I am 2 weeks clean now. Thank you to all of you for your support. I don’t have anyone else to talk about my addiction. You guys are all I have. Please continue helping me though my recovery
Leaves (Weed DAR)	I have finally decided to quit. Today is my day 0. I have smoked continuously for the last to years and I am done for good now. I am a dad and I am still doing my undergraduate. I have to focus on my graduation and being a good dad

### Overview of modeling and analysis

**Fig. 11:**
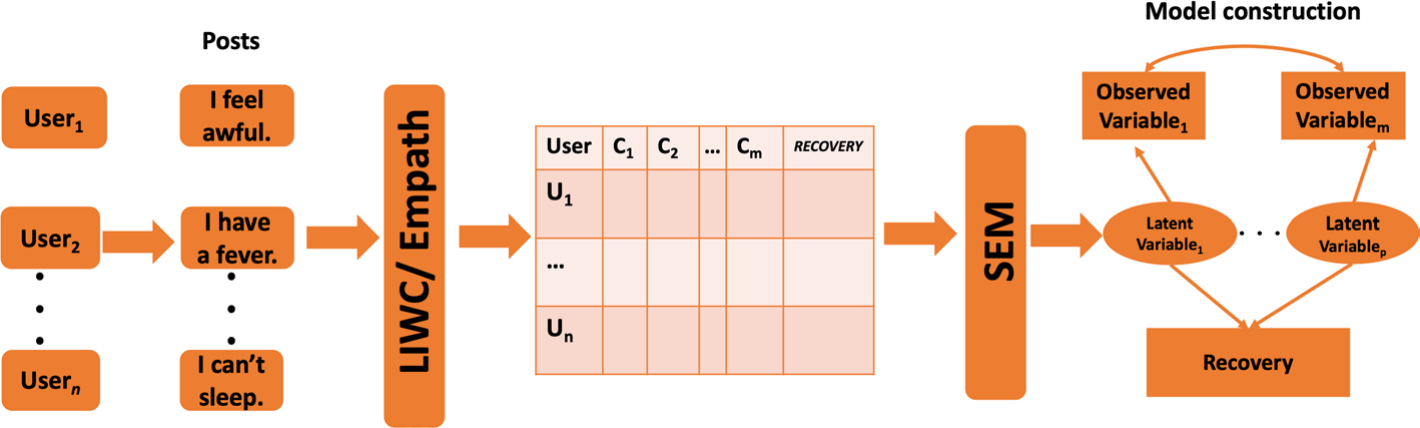
Overview of modeling and analysis process: the posts of every user were analyzed using the LIWC and Empath dictionaries to generate the matrix ${M}_{n x m+1}$, where $n$ represented the number of users and $m$ the number of LIWC or Empath categories of interest. Each cell ${M}_{ij}$ in the matrix represents LIWC and Empath generated values of category *j* for the *i*th user. The column labeled ‘*RECOVERY*’ contained a binary flag representing if the user posted in a recovery forum. The data in $M$ was subsequently analyzed using SEM

In Fig. 11 we display the key steps of our analysis process. We used LIWC or Empath to analyze the posts of the users in our dataset to extract language features, such as, negative emotions, anxiety, and pain, associated with recovery/relapse behavior of drug users. We next hypothesized certain unobserved (latent) variables for the observed features as well as the relationship between observed and latent variables. The model and its goodness of fit was iteratively analyzed and refined using SEM to obtain the final path diagram displaying the interrelationships between latent and observed variables and recovery/relapse behavior. In the following, we describe each of the modeling steps.

### Linguistic feature specification using LIWC and Empath

LIWC [48] and Empath [49] are text analysis tools developed to measure psychological, cognitive, emotional, and behavioral components in a given text sample using human-validated dictionaries. Given a piece of text, these dictionaries can be utilized to make complex determinations, such as, calculating the percentage of terms related to sadness, religion, finance, negative emotions, or physical activity. In particular, LIWC outputs the percentage of total words that belong to 90 unique categories defined therein. Empath operates similarly and uses over 200 categories. Empath can also be used to create new categories by defining appropriate seed terms. Our research used the existing categories of Empath.

### Basic concepts and definitions of structural equation modeling

In this section we describe the essential terms and concepts used in SEM. SEM is also referred to as the analysis of co-variance structure as model fitting is accomplished by utilizing the observed co-variances of the variables. For a detailed explanation of SEM, the reader is referred to [50]. SEM models are represented as a graphical representation of variable relationships and are called path diagrams. In SEM terminology *observed variables* (manifest variables) are those variables that are present in the dataset and can be measured. These variables are represented as rectangles in a path diagram. By contrast *latent variables* are not directly observable. Latent variables can be interpreted as the causes of manifest variables and are represented as ovals in the path diagram. In these diagrams, putative relationships between two variables are represented as directed edges (*paths*) weighted by path coefficients that are analogous to regression coefficients. Latent variables or error terms that co-vary are joined by curved arrows in the path diagram. SEM designates two other sets of variables: *exogenous variables* are determined to be outside of the model and have no paths pointing to them while *endogenous variables* are determined by the system of equations and have at least one path pointing to them. Both exogenous and endogenous variables can be observable or latent. Finally, for a specific model, its *degrees of freedom* (*d*), denotes the number of model parameters that are allowed to vary. Specifically, *d* is the difference between the number of possible parameters that can be estimated and number of actual parameters estimated. The number of possible parameters is quadratic in *p* -the number of observed variables while the number of estimated variables consists of all the paths (direct effects, correlations, error terms) being estimated in the model. A model is considered to be under-identified, just-identified, or over-identified if *d* < 0, *d* = 0, and *d* > 0 respectively. To estimate and evaluate the relationships in the model correctly we need to have *d* > 0.

It is important to clarify the relationship between SEM and another popular graph-based probabilistic reasoning framework, called Bayesian Networks (BN). We begin by noting that SEM does not denote a single technique; it refers to a family of related procedures. This family can be broadly characterized in terms of taking three inputs and generating three outputs [51]. The inputs being: (1) one or more qualitative causal hypotheses, (2) a set of questions about causal relations among variables of interest, and (3) a model instance. The outputs of SEM are: (1) estimates of model parameters for hypothesized effects, (2) a set of logical implications of the model that can be tested in the data, and (3) a measure of how well the testable implications of the model are supported by the data. The point of SEM is to test a theory by specifying a model that represents predictions of the aforementioned theory from among plausible constructs measured with appropriate observed variables. BN represent dependencies among sets of random variables as (causal) graphs which are traversed to update conditional probabilities of events. The ideas underlying BN have been extended to the broader problem of causal inference under a framework called the structural causal model (SCM), which is subsumed under the umbrella of SEM [52]. In our problem context, a direct application of BN entails limitations. In particular, BN cannot differentiate between causal and non-causal relationships without intervention from a domain expert [53]. Furthermore, it is non-trivial to employ BN while differentiating between latent and observed variables—a core requirement in our research. Finally, the output of BN is known not to be well suited for theoretical explanations [54].

### The process of structural equation modeling

SEM is an iterative process and involves the following steps: (1) *Model specification*: At this step a researcher hypothesizes the latent variables, the observed variables, and the relationships between them. (2) *Estimation*: The proposed model structure is estimated by using covariance analysis to solve a system of equations representing the interrelationships in the system. (3) Evaluation of *model fit*: The model fit can be evaluated using a variety of measures, such as, the comparative fit index (CFI), the Tucker Lewis index (TLI), root mean square error of approximation (RMSEA), and standardized root mean square residual (SRMR). (4) *Model re-specification*: If the initial fit is not deemed to be adequate, the model is modified and the above steps iterated.

### SEM estimation

In the estimation step the difference between the sample covariance ($C$) and the model-predicted covariance ($\tilde{C} \left( \theta \right)$) is minimized. The underlying idea is that the covariance matrix of the observed variables is a function of a set of parameters. If the parameters are correctly estimated (i.e. the model is correct) then the population covariance matrix will be exactly reproduced as shown in Eq. (1), where $\theta$ denotes the vector of model parameters.

1 $$C = \tilde{C} \left( \theta \right)$$

The standard form of the structural equation relating the endogenous and exogenous variable is:

2 $${\varvec{y}} = {\varvec{By}} + \user2{ \Gamma x} + \user2{ \zeta }$$

In Eq. (2), $\user2{y }\left( {n \times 1} \right)$ denotes the *n* dependent or endogenous variables, $\user2{x }\left( {m \times 1} \right)$ denotes the *m* exogenous variables, and ${\varvec{\zeta}} \left( {n \times 1} \right)$ denotes the specification errors. The matrix ***B ***$\left( {n \times n} \right)$ denotes the coefficients of the regression of ***y ***variables on other ***y ***variables with zeros on the diagonal which implies a variable cannot cause itself. The matrix ${\varvec{\varGamma}}$ $\left( {n \times m} \right)$ denotes the coefficients of regression of the endogenous variables on the exogenous variables. A maximum likelihood function is used to fit the structural model equations by minimizing the fitting function (*F*_*ML*_) shown in Eq. (3):

3 $$F_{ML} = \log \left| {{\varvec{C}}\left( {\varvec{\theta}} \right)} \right| + tr\left( {{\varvec{S}}{\mathbf{C}}^{ - 1} \left( {\varvec{\theta}} \right)} \right) - \log \left| {\varvec{S}} \right| - \left( {m + n} \right)$$

In Eq. (3), ***S ***is the sample covariance matrix, |.| denotes the determinant, and *tr* (.) denotes the trace of a matrix. Additionally, in SEM, it is assumed that ${\varvec{C}}\left( {\varvec{\theta}} \right)$, and ***S*** are positive-definite which means they are non-singular.

### Employing SEM for social media data modeling: an operational explanation

In this section, we explain the progression of our analysis-process from Reddit posts to a final SEM model. As the specific context, we describe the withdrawal management modeling process using LIWC indicators. To generate this model, we had used 209,804 posts from 7025 drug users. The withdrawal management model involved nine LIWC categories: “negative emotion”, “sad”, “anger”, “anxiety”, “feel”, “affect”, “swear”, “sexual”, and “authentic” which were postulated to capture the emotive underpinnings of a post. Similarly, the four LIWC categories: “biology”, “death”, “health”, and “body” were postulated to describe physical discomfort. In Table 2 we present example posts and the terms identified by LIWC for the aforementioned categories. We also present post-specific LIWC category values in the table. Also, Additional file 2: Table S1 contains the LIWC category values for a sample set of 1000 users engaged in substance use. Finally, the (binary) variable “recovery” was the outcome variable of the model; it was set to 1 if an individual posted in a DAR subreddit else it was set to 0. As explained in Fig. 11, the posts of these users were analyzed using LIWC to generate the matrix $M_{7025 x 14}$.

In SEM, variables that can be measured constitute the observable variables. In our context (Fig. 2) this role was fulfilled by the thirteen LIWC categories listed above (these variables are represented as rectangles in the path diagram shown in Fig. 2). Our hypothesis was that the latent variables (represented as ovals in Fig. 2): “*emotional distress*” could be measured using the LIWC categories: “negative emotion”, “sad”, “anger”, “anxiety”, “feel”, “affect”, “swear”, “sexual”, and “authentic”, while the latent variable “*physical pain*” could be measured via the LIWC categories: “biology”, “death”, “health”, and “body”. Finally, we hypothesized that these two latent variables had a direct effect on the recovery behavior as reflected by the Reddit posts of drug users. We measured the recovery behavior (observed variable) by using a binary variable “recovery” which was set to 1 if a user was found to have posted in drug addiction recovery forum. Alternatively, this variable was set to 0. The reader may also note that “*emotional distress*”, and “*physical pain*” were the only endogenous variables in the model; the rest of the variables being exogenous.

Next, in the SEM estimation step the difference between the population covariance ($C$), i.e., the covariance observed in LIWC variables and the “recovery” variable for the population of 7025 drug users and the hypothesized-model-predicted covariance ($\tilde{C} \left( \theta \right)$) was minimized. For our dataset, the standard form of the structural equation (Eq. (2)) relating the endogenous and exogenous variable took the following form:

4 $${\varvec{y}}_{{14\user2{ x }1}} = {\varvec{B}}_{{14\user2{ x }14}} \user2{ y}_{{14\user2{ x }1}} + \user2{ \Gamma }_{{14\user2{ x }2}} \user2{ x}_{{2\user2{ x }1}} + \user2{ \zeta }_{{14\user2{ x }1}}$$

In Eq. (4), $\user2{y }\left( {14 \times 1} \right)$ denotes the 14 exogenous variables (13—LIWC categories and 1—recovery variable), $\user2{x }\left( {2 \times 1} \right)$ denotes the 2 endogenous variables (“*emotional distress*” and “*physical pain*”), and ${\varvec{\zeta}} \left( {14 \times 1} \right)$ denotes the specification errors. The matrix ***B ***$\left( {14 \times 14} \right)$ denotes the effect of the exogenous variables on other exogenous variables while the matrix ${\varvec{\varGamma}}$ $\left( {14 \times 2} \right)$ denotes the coefficients of regression of the LIWC variables on the endogenous variables. The maximum likelihood function explained in Eq. (3) is used to fit the structural model equations by minimizing the fitting function (*F*_*ML*_) and obtain the model shown graphically in Fig. 2.

### Model evaluation

In SEM, the model fit is evaluated by examining difference between the sample covariance ($C$) and the covariance ($\tilde{C} \left( \theta \right)$) computed using the model. The goal is to minimize the difference between $C$ and $\tilde{C} \left( \theta \right)$. The simplest fitting function for SEM models is the Chi-square fit $\chi^{2}$ = $\left( {N - 1} \right)F_{ML}$. However, this function is affected by sample size; large sample sizes may increase the *χ*^2^ value even if the difference between $C$ and $\tilde{C} \left( \theta \right)$ is small and small sample sizes may lead to Type II errors [50]. The $\chi^{2}$ function however, is used as part of other fitting functions. Typically, these fitting functions are of three types: relative goodness-of-fit functions, parsimony functions, and functions that determine absolute (standalone) fit.

Examples of relative goodness-of-fit functions include the CFI (Eq. 5) and TLI (Eq. 6) measures. These measures compare the proposed model against a baseline model where all variables are allowed to have a variance, but none are allowed to co-vary. For both CFI and TLI, goodness of fit values above 0.90 denote high-quality agreement [55].

5 $$CFI = 1 - \frac{{\max \left[ {\chi_{I}^{2} - d_{I} , 0} \right]}}{{\max \left[ {\chi_{I}^{2} - df_{I} ,\chi_{B}^{2} - d_{B} ,0} \right]}}$$

6 $$TLI = \frac{{{\raise0.7ex\hbox{${\chi_{B}^{2} }$} \!\mathord{\left/ {\vphantom {{\chi_{B}^{2} } {d_{B} }}}\right.\kern-\nulldelimiterspace} \!\lower0.7ex\hbox{${d_{B} }$}} - {\raise0.7ex\hbox{${\chi_{I}^{2} }$} \!\mathord{\left/ {\vphantom {{\chi_{I}^{2} } {d_{I} }}}\right.\kern-\nulldelimiterspace} \!\lower0.7ex\hbox{${d_{I} }$}}}}{{{\raise0.7ex\hbox{${\chi_{B}^{2} }$} \!\mathord{\left/ {\vphantom {{\chi_{B}^{2} } {d_{B} }}}\right.\kern-\nulldelimiterspace} \!\lower0.7ex\hbox{${d_{B} }$}} - 1}}$$

In Eqs. (5) and (6), the baseline model is indicated by the subscript *B* while the subscript *I* denotes the proposed model. The degree of freedom is denoted by *d*.

The RMSEA [see Eq. (7)] constitutes an example of a parsimony-based fitting measure. The RMSEA takes into the account the complexity of the model by penalizing models with lower degrees of freedom since such models lead to higher values of RMSEA. RMSEA values less than 0.01, 0.05, and 0.08 are respectively considered to indicate excellent, good, or mediocre fit [55].

7 $$RMSEA = \sqrt {\frac{{\chi_{I}^{2} - d_{I} }}{{\left( {d_{I} } \right)\left( {n - 1} \right)}}}$$

In the above equation, *n* denotes the sample size.

Finally, SRMR [see Eq. (8)] is an example of an absolute fit index. SRMR is the average of standardized residuals between the observed and the model computed covariance matrices. An advantage of using SRMR over CFI, TLI, and RMSEA is that it is independent of the sample size.

8 $$SRMR = \sqrt {\frac{{\mathop \sum \nolimits_{i = 1}^{p} \mathop \sum \nolimits_{j = 1}^{i} \left[ {{\raise0.7ex\hbox{${{\text{C}}_{ij} - \tilde{C} \left( \theta \right)_{ij} }$} \!\mathord{\left/ {\vphantom {{{\text{C}}_{ij} - \tilde{C} \left( \theta \right)_{ij} } {{\text{C}}_{ii} {\text{C}}_{jj} }}}\right.\kern-\nulldelimiterspace} \!\lower0.7ex\hbox{${{\text{C}}_{ii} {\text{C}}_{jj} }$}}} \right]^{2} }}{{{\raise0.7ex\hbox{${p\left( {p + 1} \right)}$} \!\mathord{\left/ {\vphantom {{p\left( {p + 1} \right)} 2}}\right.\kern-\nulldelimiterspace} \!\lower0.7ex\hbox{$2$}}}}}$$

In the above equation ${\text{C}}_{ii}$ and ${\text{C}}_{jj}$ are the observed standard deviations and *p* is the number of observed variables. Usually, SRMR values of less than 0.08 are considered to denote models of adequate quality [55].

### Modeling withdrawal management and recovery

Withdrawal from drug addiction is accompanied by physical discomforts and negative emotions. Sedatives, opioids, and alcohol are known to cause intense physical discomforts during withdrawals, while withdrawal from substances such as marijuana, and stimulants cause emotional negativity [56]. Physical symptoms during the process of withdrawal include a variety of symptoms such as muscle aches, runny nose, dilated pupils, piloerection, insomnia, sweating, yawning, shivering, pain, cramps, weight loss, toothache, colds, and sometimes even mortality [57–59]. Emotional distress and negativity during withdrawal is characterized by aggression, anxiety, and loss of temper [60–62]. The medical approach to manage withdrawal symptoms typically involves gradually tapering doses of drug agonists to diminish the bodily discomforts and prevent a relapse. However, there are no clear methods to measure, and compare the intensity of either emotional distress or physical pain during withdrawal. In the following we describe the development of SEM models to determine the effect and importance of “*emotional distress*”, and “*physical pain*” in withdrawal management using linguistic features determined using both LIWC and Empath.

### Determining observed variables using LIWC

We used nine LIWC categories: “negative emotion”, “sad”, “anger”, “anxiety”, “feel”, “affect”, “swear”, “sexual”, and “authentic” to measure the latent variable *“emotional distress”*. Examples of terms in each of the categories are presented in Table 18. The categories “negative emotion”, “sad”, “anger”, and “anxiety” consisted of terms that had a negative connotation or valance and reflected negative thoughts. The category “feel” consisted of terms related to bodily sensations, while the category “affect” consisted of terms having both a negative and a positive connotation. We included the LIWC category “swear” as one of the indicators for *“emotional distress”* because we noticed that it was common for drug users to employ expletives to express their physical and emotional anguish. We also included the LIWC category “sexual” as one of our indicators for *“emotional distress”* because of analogous reasons. “Authentic” was a summary variable and was calculated as a single value for a given text input. The algorithm in LIWC for determining the authenticity of a text was developed based on the studies on deceptive and truthful communications [48, 63]; it determines the openness, honesty, and disclosure of a given body of text. Consequently, there are no example terms for “authentic” in Table 18. To reflect the latent variable “*physical pain*”, we used the following four LIWC categories: “biology”, “death”, “health”, and “body”. Example terms in each of these categories are presented in Table 18. The category “biology” contained terms related to human biology and biological activities. Terms representing death were present in the category “death” (bury, coffin, kill). The category “health” consisted of a number of terms related to medicine and health of an individual. The category “body” consisted of terms related to body parts and bodily functions. Additional file 2: Table S1 contains the LIWC category values for a sample set of users engaged in substance use. Finally, the (binary) variable “recovery” was the outcome variable of the model; it was set to 1 if an individual posted in a DAR subreddit else it was set to 0.

**Table 18:** Example terms present in different LIWC and Empath categories

Term category	Lexicon	Meaning	Example terms
Negative emotion	LIWC	Terms reflecting negative emotion	Hurt, ugly, nasty,):, uncomfortable, shame
Sad	LIWC	Terms depicting sadness	Crying, grief, sad, low, useless, depressive
Anger	LIWC	Terms related to anger	Hate, kill, annoyed, damn, battle, destroy
Anxiety	LIWC	TERMS related to anxiety	Fearful, unsure, afraid, panic, paranoia, misery
Feel	LIWC	Terms related to sensations	Pain, painful, hurt, feels, touch
Affect	LIWC	Terms related to affect (feeling or emotion)	Cried, unsure, worst, depress, painful, killing
Swear	LIWC	Swear terms	Hell, crap, screw, pissed, shitstorm, dumb
Sexual	LIWC	Terms related to sex and sexual orientations	f***, stds, screwed, screw, aids, unplanned
Biology	LIWC	Terms reflecting biological processes	Brain, body, sleep, mouth, dosing, live
Death	LIWC	Terms related to death	Slay, dead, die, bury, od, hard,
Health	LIWC	Terms related to health	Dose, nauseas, drug, druggie, pain, addiction
Body	LIWC	Terms associated with body and body parts	Sleep, mouth, hand, sweating, blood, urinary
Hate	Empath	Terms depicting hatred	Hate, disgust, dislike, worse, awful, nasty
Negative_emotion	Empath	Terms reflecting negative emotion	Crying, stop, crushed, worried, scared, hard
Shame	Empath	Terms depicting shame	Uneasiness, suffer, terror, pitiful, shameful, sorrowful
Suffering	Empath	Terms related to suffering	Suffering, painful, tears, torture, excruciating, regret
Pain	Empath	Terms associated with pain	Pain, kill, kick, bad, headache, sick
Medical_emergency	Empath	Terms related to a medical emergency	Epilepsy, trauma, flu, lifeless, seizure, fever
Weakness	Empath	Terms depicting weakness	Shaky, weariness, emaciated, weakening, fatigue, frail
Health	Empath	Terms related to health	Health, clinic, pill, cramp, chronic, diarrhea
Emotional	Empath	Terms depicting an individual’s emotions	Suicidal, unhappy, rant, miserable, angry, mad
Swearing_terms	Empath	Swear terms	Hell, curse, swear, damn, shit, retard
Rural	Empath	Terms depicting a rural setting	Barren, cornfield, plantation, meadow, farmhouse, village
White_collar_job	Empath	Terms associated with white collar jobs	Manager, lawyer, nurse, job, engineer, analyst, salary
Blue_collar_job	Empath	Terms associated with blue collar jobs	Serving, maid, pizzeria, clerk, waiter, bartender
Office	Empath	Terms used in an office setting	Laptop, manager, fax, reception, printing, workplace

### Determining observed variables using Empath

We used four Empath categories: “negative_emotion”, “hate”, “shame”, and “suffering” to measure the latent variable *“emotional distress”*. Examples of terms in each of the categories are presented in Table 18. The categories “negative_emotion”, “hate”, “shame”, and “suffering” all consisted of terms that had a negative undertone and reflected negative feelings. To reflect the latent variable “*physical pain*”, we used the following four Empath categories: “pain”, “medical_emergency”, “health”, and “weakness” (see Table 18 for examples). The category “pain” contained terms related to physical discomfort. Terms representing a medical emergency were present in the category “medical_emergency”. The category “health” consisted of a number of terms related to the health of an individual and the category “weakness” consisted of terms related to lack of strength of an individual. Again, the (binary) variable “recovery” was the outcome variable of the model; it was set to 1 if an individual posted in a DAR subreddit else it was set to 0.

### The SEM model for withdrawal management

The SEM modeling was conducted using the lavaan package [64]. Here, we estimate the effect of “emotional distress” and “physical pain” on drug addiction recovery behavior using LIWC and Empath. As mentioned before, drug addiction recovery behavior was measured using an observed variable (“*recovery*”). The reader may note that the LIWC model was based on our estimation of the latent variable “*emotional distress*”, using nine indicators [(1)“negative emotion”, (2) “authentic”, (3) “sad”, (4) “affect”, (5) “anger”, (6) “anxiety”, (7) “sexual”, (8) “feel”, and (9) “swear”]. Similarly, the latent variable and “*physical pain*”, was estimated with four indicators [(1) “health”, (2) “biology”, (3) “death”, and (4) “body”]. The Empath model estimated the latent variable “*emotional distress*”, using four indicators [(1)“negative_emotion”, (2) “hate”, (3) “suffering”, and (4) “shame”]. Similarly, the latent variable “*physical pain*”, was estimated with four indicators [(1) “health”, (2) “weakness”, (3) “pain”, and (4) “medical_emergency”]. It may be noted that the LIWC and Empath categories were not exclusive in that terms could simultaneously belong to different categories. We also observed that terms of certain categories frequently co-occurred. For example, in posts describing effects of withdrawal, expression of negative emotions or terms describing sadness would usually co-occur with terms associated with health. Consequently, such variables were allowed to co-vary in our models. The specific models obtained using the LIWC and Empath variables are described in the "Results" section.

### The SEM model for recovery

Self-development efforts and relationships have been found to be indispensable for drug addiction recovery [65]. Family support, especially for adolescents in long term residential programs has been proven to be necessary for successful recovery from addiction [66]. Studies have also showed that having a strong social and family resource improves the chances of addiction recovery [67–70].

Self-development efforts encompassing activities that lead to mental and physical well-being, such as regular exercise, meditation, and yoga have been observed to help heal the body and mind [71, 72]. Such activities have also been shown to address psychological and physiological needs of a recovering addict by reducing negative feelings and preventing weight gain following abstinence. Additionally, regular exercise is known to alleviate physical and mental stress. It is also known to positively alter the brain chemistry as it releases endorphins and creates a natural high, similar to ones released when an individual uses drugs. Studies have shown that addition of exercise as a lifestyle change leads to abstinence or reduction in drug use [31–34]. Mediation and yoga has also been proved to help individuals in their withdrawals and addiction by acting a calming effect during their period of struggles [35–37]. Professional activities constitute another aspect of self-development. However, the literature on the importance of jobs, and career on addiction recovery is ambiguous: some sources suggest that a stable job helps provide the recovering addicts with income and health benefits, improved mental health, and a purpose in their life. For example, Flynn et al. [72], found job/career to be one of the fundamental personal motivations for a recovering addict to stay sober. The importance of vocational rehabilitation and job search as one of the services in the social model of recovery has also been noted [73]. Other works have found that employed individuals undergoing recovery are more engaged in recovery activities and are more likely to abstain from substance use [74–77]. However, studies also have found that returning to old jobs, or stress experienced at work can lead to drug use and relapse [76]. Amongst these, Buczkowski et al., identified smoking environment at work as one of the triggers for relapse of smoking [77]. The stress associated with changing jobs has been cited to lead to substance use relapse [78–82]. Furthermore, the social stigma associated with drug addiction has been found to play a major role in the unwillingness of working individuals to opt for recovery interventions [83]. Finally, since employers are prejudiced against recovering addicts applying for jobs, such situations can also lead to a relapse or unwillingness to come out as an addict [83].

Because of the aforementioned reasons self-development efforts and relationships play a pivotal role in withdrawal management and drug addiction recovery. We therefore construct a SEM model to determine the effect and importance of the latent variables “*mental and physical well-being*”, “*career*”, and “*relationships*” in drug addiction recovery. To estimate these latent variables, we utilized forum activity of the drug users in multiple subreddits related to self-development efforts and relationships. We used the number of times an individual posted in the following eight subreddits: “fitness”, “meditation”, “yoga”, “gainit”, “bodyweightfitness”, and “running” to estimate the latent variable *“mental and physical well-being”*. Similarly, we used the posts in the subreddits: “jobs”, “entrepreneur”, “careerguidance”, and “resumes” to estimate latent variable “*career*”. As indicator variable for “*relationships*” we used the posts in the four subreddits: “relationship_advice”, “relationships”, “parenting”, and “childfree”. Finally, our outcome variable for the model was “recovery”. The SEM model captures the effect of these variables on addiction recovery.

### Modeling addiction relapse

As described above, the variables “*emotional distress*”, “*physical pain*”, “*relationships*”, and “*self-development*” were found to play a critical role in addiction recovery. In addition to these factors, religion and geographic disparities were also found by us to influence the process of recovery. These results are supported by previous work in the field of relapse where it was found that recovering individuals display higher levels of religious faith [84–87]. Similarly, researchers have observed that addicts living in a rural setting have a higher chance for relapse as compared to their urban counterparts [88–91] because of limited access to relapse prevention facilities and preventive medications. In the following, we describe models that study the effect of the aforementioned latent variables along with demographic setting for drug users who undergo relapse. We defined relapse as the event of an individual posting in an RDU subreddit after posting in a DAR subreddit. Individuals who never posted in an RDU subreddit after posting in a DAR subreddit were defined to be in (continued) recovery. Based on these definitions 2363 individuals in our dataset were found to have relapsed, while 1355 users displayed continued recovery. To study users who relapsed while minimizing the impact of stray postings, we investigated only those users who had at least five posts in succession in a DAR subreddit before they were defined to have relapsed. Similarly, to study users who displayed signs of continued recovery we investigated only who had at least five posts in DAR subreddits before they stopped posting. As a consequence of this filtering, we ended up with a total of 174 users of whom 108 were identified to have relapsed while 66 users were identified to have continued their recovery journey till our observations concluded. Also, to extract relapse specific information, we scaled the values for LIWC and Empath categories by dividing them by the number of days between the post under investigation and the day when the user was defined to have relapsed.

### Determining observed variables using LIWC for modeling relapse

While modeling users who relapsed we observed a limitation of using psycholinguistic dictionaries such as LIWC and Empath. Anti-social behavior, lack of religious expression, physical exercise, and positive emotion increases the chances of a relapse. However, using these dictionaries we could only obtain a value for the presence of such categories, i.e., the absence of such psycholinguistic information was not represented via any appropriate categories. To overcome this weakness and to build a model for relapse using LIWC, we generated values for such (absent, in LIWC or Empath) variables by subtracting the numeric weight of the corresponding LIWC/Empath categories from 1. For example, if a post had a value of 0.2 for the category “friends”, we calculated the value of “friends΄” (i.e. the negation of the category “friends”) to be 0.8 (hereafter, such variables are referred to as negated variables and denoted by a prime). We used negation of the following six LIWC categories “friend”, “we”, “shehe”, “you”, “male”, and “female” to represent and study the latent variable *“anti-social”*. To model lack of physical exercise and religious expression we used the negation of LIWC categories “motion” and “religion”. The (binary) variable “relapse” was the outcome variable in our model; it was set to 1 if an individual relapsed else it was set to 0.

### Determining observed variables using Empath

We used Empath to model the relapse behavior as a consequence of lack of positive emotion, career interests, and urban facilities. Similar to obtaining the values of LIWC categories for modeling relapse, we used negation of the following four Empath categories “joy”, “zest”, “cheerfulness”, and “positive emotion” to study the latent variable *“positive emotion*΄*”* (lack of positive emotion). To model “*career΄*” (and lack of career development), we used the negation of the following three Empath categories: “blue_collar_job”, “white_collar_job”, and “office”. Finally, to model “*urban*΄” (i.e.*,* the lack of an urban setting and facilities) we used the negation of LIWC category “urban”. The (binary) variable “relapse” was the outcome variable in our model; it was set to 1 if an individual relapsed else it was set to 0.

### The SEM model for relapse of addiction

In this model we estimated the effect of factors including the social and physical activities of a drug user, their positive or negative emotions, recourse to religion, career-related activities, and location (urban or rural) on relapse by employing linguistic characteristics determined using LIWC and Empath. The relapse behavior was itself measured using the observed variable “relapse”. The latent variable *“anti-social”* was estimated using six negated LIWC categories (“friend΄”, “we΄”, “shehe΄”, “you΄”, “male΄”, and “female΄”) and two observed negated variables “motion΄” and “religion΄”. The Empath model estimated the latent negation variable “*positive emotion*΄” using four negated categories (“joy΄”, “zest΄”, “cheerfulness΄”, and “positive emotion΄”). Similarly, the latent negated variable “*career΄*” was estimated using three negated categories (“blue_collar_job΄”, “white_collar_job΄”, and “office΄”). Finally, the variable “urban΄” corresponding to the location of the user was an observed variable in the model. The models obtained using the LIWC and Empath variables are described in the "Results" section.

### User privacy

Any investigation of the type reported by us must take cognizance of user privacy concerns. In our case, the data used in this paper was publicly available (via Reddit) and the authors did not have personal interaction with any of the users. Even though this data is publicly available, to ensure user privacy, we anonymized the data and all examples presented in the paper were paraphrased.

## Supplementary information

**Additional file 1**. Supplementary Tables and Analysis.

**Additional file 2**. LIWC category values for a sample set of 1000 users.

## Data Availability

The primary data used in this paper is publicly available from Reddit. We are unable to act as a secondary source of this data because of the rules and regulations as enforced by Reddit. According to these rules, Reddit account holders own their content and data. Reddit grants the developers a non-exclusive, non-transferable, and revocable license which does not allow for further data sharing and sub-licensing. The LIWC category values for a sample set of substance users is presented in Additional file 2: Table S1.

## Abbreviations

SEM: Structural equation modeling
RDU: Recreational drug use
DAR: Drug addiction recovery
LIWC: Linguistic Inquiry and Word Count
BN: Bayesian Networks
SCM: Structural causal model
CFI: Comparative fit index
TLI: Tucker Lewis index
RMSEA: Root mean square error of approximation
SRMR: Standardized root mean square residual
