# Supplementary material for: Analysis of associations between emotions and activities of drug users and their addiction recovery tendencies from social media posts using structural equation modeling
Source: BMC Bioinformatics. 2020 Dec 30;21(Suppl 18):554. doi: 10.1186/s12859-020-03893-9 (PMC7772931; doi:10.1186/s12859-020-03893-9)
Supplement: Supplementary file 1 — Additional file 1. Supplementary Tables and Analysis. [file 12859_2020_3893_MOESM1_ESM.docx]

Table S1. Comparison of normalized values for different Empath variables in our withdrawal management model for users who display and do not display addiction recovery behavior. Example terms in each category are underlined. We observe high values for all the chosen LIWC categories for users displaying addiction recovery behavior.

|  |  | **Individuals displaying signs of addiction recovery** | | **Individuals not displaying signs of addiction recovery** | |  |
| --- | --- | --- | --- | --- | --- | --- |
|  | **Example Posts** | Mean | SD | Mean | SD | p < |
| medical_emergency | I feel really sick today. I always panic when I give up drug s and end up feeling sick. | 0.07 | 0.07 | 0.04 | 0.06 | 0.005 |
| weakness | I have become what I always feared. Also, my chest hurts. | 0.029 | 0.04 | 0.023 | 0.04 | 0.005 |
| health | These wds are worsening my depression. | 0.12 | 0.11 | 0.09 | 0.11 | 0.005 |
| pain | Can kratom help me for my chronic pain? | 0.20 | 0.11 | 0.16 | 0.12 | 0.005 |
| negative_emotion | I have to beat this addiction and get out this hell. | 0.20 | 0.08 | 0.16 | 0.12 | 0.005 |
| shame | Detoxing without kratom/subs is so miserable. | 0.17 | 0.10 | 0.13 | 0.11 | 0.005 |
| suffering | I have relapsed and now have a horrific daily intake. | 0.13 | 0.09 | 0.10 | 0.10 | 0.005 |
| hate | I am having terrible knee pain bc of wds. | 0.12 | 0.08 | 0.09 | 0.09 | 0.005 |

**Supplementary Tables and Analysis**

In Table S1 we compare the values of the Empath indicators for the latent variables “*emotional distress*”, and “*physical pain*” between the users who post and do not post in DAR subreddits. The distributions of the values of indicators for the set of users who posted in a DAR subreddit was compared with the set of users who did not post in a DAR subreddit with the null hypothesis that there was no difference between the distributions. The Mann-Whitney U test was used to compare the distributions and we observed statistically significant differences between the two set of users for each variable.

The values of the indicators for the latent variable “*emotional distress” and “physical pain”* were found to be higher for users who displayed addiction recovery behavior. Posts corresponding to addiction recovery behavior typically consisted of higher values for the Empath categories: “medical_emergency” (54.4 %, *p*<0.005), “weakness” (23.07 %, *p*<0.005), “health” (28.5%, *p*<0.005), “pain” (22.2 %, *p*<0.005), “negative_emotion” (22.2 %, *p*<0.005), “shame” (26.6 %, *p*<0.005), “suffering” (26.0 %, *p*<0.005), and “hate” (40.0 %, *p*<0.005), as compared to the other Empath categories used by us (Table S1). Accordingly, our data shows that drug users complained about their emotional and physical discomforts during the withdrawal phase.

Table S2. Comparison of normalized values for different Empath variables in our relapse model for users who display and do not display relapse behavior. Example terms in each category are underlined. We observe high values for all the chosen Empath categories for users displaying relapse behavior.

|  |  | **Individuals who relapsed** | | **Individuals who did not relapse** | |  |
| --- | --- | --- | --- | --- | --- | --- |
|  | **Example Posts** | Mean | SD | Mean | SD | p < |
| joy΄ | I have relapsed and now have a horrific daily intake. | 0.99 | 0.02 | 0.83 | 0.19 | 0.005 |
| zest΄ | I am feeling anxious, emotional and depressed. | 0.99 | 0.02 | 0.83 | 0.20 | 0.005 |
| cheerfulness΄ | These wds are killing me. I want this shit to stop rn. | 0.99 | 0.005 | 0.91 | 0.16 | 0.5 |
| positive emotion΄ |  | 0.98 | 0.09 | 0.94 | 0.03 | 0.005 |
| white_collar_job΄ | Currently, I am job searching and I also have to manage my cravings. | 0.99 | 0.01 | 0.79 | 0.25 | 0.05 |
| office΄ | My manager is suddenly asking me for my id. | 0.98 | 0.03 | 0.79 | 0.23 | 0.005 |
| blue_collar_job΄ | I want to focus on my job and become a better worker. | 0.99 | 0.02 | 0.89 | 0.19 | 0.5 |
| urban΄ | I am a farmer and have to do a lot of physical tasks and I miss my opioids. | 0.98 | 0.05 | 0.91 | 0.16 | 0.5 |

Table S2 presents a comparison of the values for the following empath indicators: “joy΄”, “zest΄”, “cheerfulness΄”, “positive_emotion΄”, “white_collar_job΄”, ”blue_collar_job΄”, “office΄΄”, and “urban”, between the users who relapse and do not relapse. The distributions of the values of indicators for the set of users who relapsed was compared with the set of users who did not with the null hypothesis that there is no difference between the distributions. Mannwhitney U test was used to compare the distributions.

The values of the all the indicators were found to be higher for the users who relapsed. Posts corresponding to relapse behavior typically consisted of higher values for the categories: “joy΄” (17.5 %, *p*<0.005), “zest΄” (17.5%, *p*<0.005), “cheerfulness΄” (8.4%, *p*<0.5), “positive emotion΄” (4.1%, p <0.005 ), “white_collar_job΄” (22..4 %, *p*< 0.05), “blue_collar_job΄” (10.6 %, *p*<0.5), “office΄” (22.4 %, *p*<0.005), and “*urban*΄” (7.4%, *p* <0.5). Accordingly, our data shows that users who relapse have higher values for “*positive emotion*΄”, “career΄”, and “urban΄” facilities.

Table S3. Comparison of normalized values for different LIWC variables in our model for users who display and do not display relapse behavior. Example terms in each category are underlined. We observe high values for all the chosen LIWC categories for users displaying relapse behavior.

|  |  | **Individuals who relapsed** | | **Individuals who did not relapse** | |  |
| --- | --- | --- | --- | --- | --- | --- |
|  | **Example Posts** | Mean | SD | Mean | SD | p < |
| *friend*΄ | I just called my best friend and confessed to him that I am addicted. | 0.98 | 0.03 | 0.82 | 0.17 | 0.005 |
| *we*΄ | Here we go again. Day 1. | 0.97 | 0.12 | 0.88 | 0.15 | 0.005 |
| *shehe*΄ | She is my pillar in this detox. | 0.98 | 0.02 | 0.82 | 0.22 | 0.005 |
| *you*΄ | I want to know your experience with managing wds. | 0.98 | 0.02 | 0.77 | 0.19 | 0.005 |
| *male*΄ | Guys my life has fallen apart and I need your help. | 0.98 | 0.03 | 0.78 | 0.22 | 0.005 |
| *female*΄ | My mother has been always there for me. | 0.99 | 0.00 | 0.89 | 0.16 | 0.005 |
| *Tone*΄ |  | 0.96 | 0.12 | 0.85 | 0.05 | 0.005 |
| *motion*΄ | What do you guys think about multiweek hike in early recovery? | 0.97 | 0.11 | 0.92 | 0.14 | 0.005 |
| *religion*΄ | Jesus, so sick rn. Help me god. | 0.98 | 0.03 | 0.83 | 0.21 | 0.005 |

In Table S3 we compare the values of the LIWC of the following indicators: “Tone΄”, “motion΄”, “religion΄”, “friend΄”, ”we΄”, “shehe΄”, “you΄”, “male΄”, “female΄” between the users who relapse and do not relapse. The distributions of the values of indicators for the set of users who relapsed was compared with the set of users who did not with the null hypothesis that there is no difference between the distributions. The Mann-Whitney U test was used to compare the distributions and we observed statistically significant differences between the two set of users for each observable variable.

Posts corresponding to relapse behavior consisted of higher values for the LIWC categories: “friend΄” (17.7 %, *p*<0.005), “w΄e” (9.7 %, *p*<0.005), “shehe΄” (17.7%, *p*<0.005), “you΄” (24 %, *p*<0.005), “male΄” (22.7 %, *p*<0.005), and “female΄” (10.6 %, *p*<0.005). Values were also higher for “Tone΄ “ (11.4%, *p* <0.005), “motion΄” ( 5.2%, *p* < 0.005) , and “*religion*΄” (15.3%, *p* < 0.005). Accordingly, our data shows that users who relapse have higher values for “anti-*social*”, “religion΄”, “Tone΄”, and “motion΄”.

Table S4. Correlation matrix of all the observed variables present in the recovery efforts model. We observe lower correlation scores as compared to the LIWC categories. The highest correlation is observed for the subreddits “careerguidance” and “resume”.

|  | Meditation | yoga | Fitness | gainit | running | bodyweightfitness | relationships | relationship_advice | Parenting | childfree | jobs | Entrepreneur | careerguidance | resumes |
| --- | --- | --- | --- | --- | --- | --- | --- | --- | --- | --- | --- | --- | --- | --- |
| Meditation | 1 | 0.06 | 0.06 | 0.02 | 0.04 | 0.03 | 0.009 | 0.04 | 0.02 | 0.01 | 0.02 | 0.02 | 0.02 | 0.1 |
| yoga |  | 1 | 0.02 | 0.005 | 0.008 | 0.2 | 0.004 | 0.07 | 0.03 | 0.01 | 0.04 | 0.09 | 0.001 | -0.01 |
| Fitness |  |  | 1 | 0.12 | 0.12 | 0.22 | 0.10 | 0.05 | 0.04 | 0.001 | 0.09 | 0.06 | 0.02 | 0.04 |
| gainit |  |  |  | 1 | 0.002 | 0.06 | 0.03 | 0.02 | 0.008 | 0.0001 | 0.05 | 0.02 | 0.009 | 0.01 |
| running |  |  |  |  | 1 | 0.05 | 0.02 | 0.02 | 0.07 | -0.002 | 0.02 | 0.01 | 0.02 | 0.04 |
| bodyweightfitness |  |  |  |  |  | 1 | 0.04 | 0.07 | 0.008 | -0.004 | 0.03 | 0.08 | 0.002 | 0.001 |
| relationships |  |  |  |  |  |  | 1 | 0.18 | 0.004 | 0.009 | 0.26 | 0.01 | 0.07 | 0.02 |
| relationship_advice |  |  |  |  |  |  |  | 1 | 0.009 | 0.01 | 0.32 | 0.03 | 0.04 | 0.05 |
| Parenting |  |  |  |  |  |  |  |  | 1 | 0.01 | 0.03 | 0.03 | 0.06 | 0.04 |
| childfree |  |  |  |  |  |  |  |  |  | 1 | 0.002 | 0.006 | -0.003 | -0.005 |
| jobs |  |  |  |  |  |  |  |  |  |  | 1 | 0.13 | 0.14 | 0.18 |
| Entrepreneur |  |  |  |  |  |  |  |  |  |  |  | 1 | 0.21 | 0.17 |
| Careerguidance |  |  |  |  |  |  |  |  |  |  |  |  | 1 | 0.3 |
| resumes |  |  |  |  |  |  |  |  |  |  |  |  |  | 1 |
