# Supplementary material for: Analysis of associations between emotions and activities of drug users and their addiction recovery tendencies from social media posts using structural equation modeling
Source: BMC Bioinformatics. 2020 Dec 30;21(Suppl 18):554. doi: 10.1186/s12859-020-03893-9 (PMC7772931; doi:10.1186/s12859-020-03893-9)
Supplement: Supplementary file 2 — Additional file 2. LIWC category values for a sample set of 1000 users. [file 12859_2020_3893_MOESM2_ESM.docx]

**LIWC category values for a sample set of 1000 users**

Table S1. LIWC category values for a sample set of 1000 substance users. The ‘recovery’ column was set to one if the user displayed addiction recovery behavior.

| user_number | recovery | negemo | health | Authentic | bio | sad | affect | anger | swear |
| --- | --- | --- | --- | --- | --- | --- | --- | --- | --- |
| 1 | 1 | 0.09815951 | 0.06426735 | 0.91326531 | 0.19457014 | 0.06422018 | 0.30134814 | 0 | 0.07692308 |
| 2 | 1 | 0.37116564 | 0.37403599 | 0.58632653 | 0.4561086 | 0.37614679 | 0.4258525 | 0.2408377 | 0.13793103 |
| 3 | 1 | 0.20092025 | 0.14652956 | 0.84489796 | 0.17737557 | 0.20183486 | 0.44409199 | 0.07068063 | 0.04244032 |
| 4 | 1 | 0.34815951 | 0.14524422 | 0.59265306 | 0.28235294 | 0.12844037 | 0.3370341 | 0.07329843 | 0.07427056 |
| 5 | 1 | 0.16257669 | 0.25578406 | 0.74540816 | 0.28868778 | 0.12385321 | 0.30610626 | 0.10471204 | 0.1061008 |
| 6 | 1 | 0.27300613 | 0.12339332 | 0.85642857 | 0.35837104 | 0.16513761 | 0.37906423 | 0.28534031 | 0.28912467 |
| 7 | 1 | 0.28220859 | 0.42673522 | 0.75602041 | 0.37828054 | 0.33944954 | 0.43854084 | 0.12827225 | 0.09814324 |
| 8 | 1 | 0.28067485 | 0.19922879 | 0.46622449 | 0.25882353 | 0.29357798 | 0.34575734 | 0.14659686 | 0.10079576 |
| 9 | 1 | 0.4493865 | 0.13496144 | 0.94459184 | 0.38823529 | 0.16055046 | 0.45757335 | 0.44764398 | 0.47480106 |
| 10 | 1 | 0.39723926 | 0.23521851 | 1 | 0.34479638 | 0 | 0.55511499 | 0.2382199 | 0.24137931 |
| 11 | 1 | 0.26687117 | 0.17609254 | 0.79040816 | 0.28506787 | 0.2293578 | 0.34179223 | 0.10732984 | 0.066313 |
| 12 | 1 | 0.46165644 | 0.3251928 | 0.61336735 | 0.44977376 | 0.17431193 | 0.46391753 | 0.28795812 | 0.18832891 |
| 13 | 1 | 0.22546012 | 0.21079692 | 0.92071429 | 0.23710407 | 0.0733945 | 0.23394132 | 0.21465969 | 0.25994695 |
| 14 | 1 | 0.43251534 | 0.14267352 | 0.64102041 | 0.24524887 | 0.12844037 | 0.35527359 | 0.2460733 | 0.27851459 |
| 15 | 1 | 0.32208589 | 0.13496144 | 0.71897959 | 0.22986425 | 0.13761468 | 0.28469469 | 0.23560209 | 0.15915119 |
| 16 | 1 | 0.31595092 | 0.20822622 | 0.90765306 | 0.31945701 | 0.2706422 | 0.52498017 | 0.23036649 | 0.11671088 |
| 17 | 1 | 0.40644172 | 0.37660668 | 0.43040816 | 0.38099548 | 0.17431193 | 0.51784298 | 0.17277487 | 0.11405836 |
| 18 | 1 | 0.24386503 | 0.15809769 | 0.6805102 | 0.40723982 | 0.09174312 | 0.31562252 | 0.11518325 | 0.09549072 |
| 19 | 1 | 0.21932515 | 0.29048843 | 0.84244898 | 0.32217195 | 0.10091743 | 0.30531324 | 0.05235602 | 0.09018568 |
| 20 | 1 | 0.40490798 | 0.26092545 | 0.96265306 | 0.32307692 | 0.12844037 | 0.36161776 | 0.22513089 | 0.19363395 |
| 21 | 1 | 0.41717791 | 0.13624679 | 0.76683673 | 0.34208145 | 0.32568807 | 0.53370341 | 0.37172775 | 0.40583554 |
| 22 | 1 | 0.35736196 | 0.30719794 | 0.86734694 | 0.3800905 | 0.18807339 | 0.35130849 | 0.18324607 | 0.14058355 |
| 23 | 1 | 0.23619632 | 0.21979434 | 0.86887755 | 0.27873303 | 0.07798165 | 0.36637589 | 0.08900524 | 0.09018568 |
| 24 | 1 | 0.3696319 | 0.27377892 | 0.53642857 | 0.49049774 | 0.05504587 | 0.36003172 | 0.2617801 | 0.23342175 |
| 25 | 1 | 0.37269939 | 0.13367609 | 0.92295918 | 0.27511312 | 0.39908257 | 0.53132435 | 0.2513089 | 0.20689655 |
| 26 | 1 | 0.46932515 | 0.15809769 | 0.67663265 | 0.40180995 | 0.16055046 | 0.44647105 | 0.42670157 | 0.30238727 |
| 27 | 1 | 0.36042945 | 0.25578406 | 0.84979592 | 0.45791855 | 0.20642202 | 0.40523394 | 0.27748691 | 0.23076923 |
| 28 | 1 | 0.32668712 | 0.22107969 | 0.83755102 | 0.28687783 | 0.16972477 | 0.45519429 | 0.15706806 | 0.0397878 |
| 29 | 1 | 0.51226994 | 0.30077121 | 0.50357143 | 0.41538462 | 0.42201835 | 0.49008723 | 0.13089005 | 0.066313 |
| 30 | 1 | 0.26993865 | 0.21079692 | 0.6722449 | 0.26696833 | 0.19266055 | 0.36161776 | 0.10994764 | 0.11140584 |

| 31 | 1 | 0.44631902 | 0.08354756 | 0.8605102 | 0.39457014 | 0.14678899 | 0.58921491 | 0.33769634 | 0.34217507 |
| --- | --- | --- | --- | --- | --- | --- | --- | --- | --- |
| 32 | 1 | 0.21625767 | 0.11953728 | 0.80357143 | 0.15384615 | 0.20183486 | 0.370341 | 0.10209424 | 0.06366048 |
| 33 | 1 | 0.2208589 | 0.69537275 | 0.95806122 | 0.57104072 | 0.16513761 | 0.24266455 | 0.04712042 | 0 |
| 34 | 1 | 0.37576687 | 0.10796915 | 0.80255102 | 0.2361991 | 0.06880734 | 0.50436162 | 0.2617801 | 0.24403183 |
| 35 | 1 | 0.33128834 | 0.21079692 | 0.93693878 | 0.22533937 | 0.23853211 | 0.44647105 | 0.12041885 | 0.06896552 |
| 36 | 1 | 0.32822086 | 0.32904884 | 0.81377551 | 0.40542986 | 0.05045872 | 0.49088025 | 0.13874346 | 0.08488064 |
| 37 | 1 | 0.35736196 | 0.14781491 | 0.65357143 | 0.31312217 | 0.12385321 | 0.41316416 | 0.28272251 | 0.24137931 |
| 38 | 1 | 0.47699387 | 0.3059126 | 0.91612245 | 0.37013575 | 0.30275229 | 0.51070579 | 0.15183246 | 0.09814324 |
| 39 | 1 | 0.5690184 | 0.35347044 | 0.84132653 | 0.51493213 | 0.24770642 | 0.4369548 | 0.21989529 | 0.17506631 |
| 40 | 1 | 0.29754601 | 0.33161954 | 0.9394898 | 0.39457014 | 0.14678899 | 0.38461538 | 0.17015707 | 0.08488064 |
| 41 | 1 | 0.49693252 | 0.24807198 | 0.89438776 | 0.26968326 | 0.24311927 | 0.45836638 | 0.2513089 | 0.22015915 |
| 42 | 1 | 0.51380368 | 0.2840617 | 0.99540816 | 0.31312217 | 0.35779817 | 0.44567803 | 0.20418848 | 0.19098143 |
| 43 | 1 | 0.24079755 | 0.61182519 | 0.62479592 | 0.58823529 | 0.05504587 | 0.36399683 | 0.06020942 | 0.01591512 |
| 44 | 1 | 0.22699387 | 0.30976864 | 0.17244898 | 0.27420814 | 0.19724771 | 0.27597145 | 0.07329843 | 0.0132626 |
| 45 | 1 | 0.30828221 | 0.31876607 | 0.81459184 | 0.36832579 | 0.19724771 | 0.41871531 | 0.06806283 | 0.02122016 |
| 46 | 1 | 0.36042945 | 0.2622108 | 0.72030612 | 0.27782805 | 0.2293578 | 0.4147502 | 0.14921466 | 0.10344828 |
| 47 | 1 | 0.23619632 | 0.20437018 | 0.82530612 | 0.25067873 | 0.18348624 | 0.36637589 | 0.04712042 | 0.02387268 |
| 48 | 1 | 0.35122699 | 0.28277635 | 0.96091837 | 0.5719457 | 0.16972477 | 0.38540841 | 0.28795812 | 0.24403183 |
| 49 | 1 | 0.32822086 | 0.14652956 | 0.80244898 | 0.31493213 | 0.08715596 | 0.48374306 | 0.2486911 | 0.23607427 |
| 50 | 1 | 0.25460123 | 0.23521851 | 0.78112245 | 0.30045249 | 0.09633028 | 0.38858049 | 0.12303665 | 0.11405836 |
| 51 | 1 | 0.47392638 | 0.15938303 | 0.83 | 0.33574661 | 0.33027523 | 0.58842189 | 0.21465969 | 0.08222812 |
| 52 | 1 | 0.59969325 | 0.36632391 | 0.82071429 | 0.44162896 | 0.55963303 | 0.61300555 | 0.42670157 | 0.47480106 |
| 53 | 1 | 0.28374233 | 0.42673522 | 0.96908163 | 0.55656109 | 0.22477064 | 0.30214116 | 0 | 0 |
| 54 | 1 | 0.17791411 | 0.16966581 | 0.84081633 | 0.34479638 | 0 | 0.34099921 | 0.21727749 | 0.22015915 |
| 55 | 1 | 0.33128834 | 0.21208226 | 0.89234694 | 0.22262443 | 0.17431193 | 0.33624108 | 0.10732984 | 0.05835544 |
| 56 | 1 | 0.26380368 | 0.05912596 | 0.84102041 | 0.17466063 | 0.13761468 | 0.28945282 | 0.12041885 | 0.0530504 |
| 57 | 1 | 0.34509202 | 0.20308483 | 0.94653061 | 0.20361991 | 0.10550459 | 0.35765266 | 0.20680628 | 0.20954907 |
| 58 | 1 | 0.49233129 | 0.35732648 | 0.82826531 | 0.36832579 | 0.21559633 | 0.4036479 | 0.06806283 | 0.01061008 |
| 59 | 1 | 0.4202454 | 0.4151671 | 0.85346939 | 0.39457014 | 0.25688073 | 0.45122918 | 0.12827225 | 0.07427056 |
| 60 | 1 | 0.23312883 | 0.20694087 | 0.94632653 | 0.27420814 | 0 | 0.48057098 | 0.02356021 | 0.02387268 |
| 61 | 1 | 0.78220859 | 0.39974293 | 0.96142857 | 0.52307692 | 0.2293578 | 0.52735924 | 0.68324607 | 0.51193634 |

| 62 | 1 | 0.34662577 | 0.1940874 | 0.76653061 | 0.27330317 | 0.30275229 | 0.51546392 | 0.14921466 | 0.15119363 |
| --- | --- | --- | --- | --- | --- | --- | --- | --- | --- |
| 63 | 1 | 0.23006135 | 0.30848329 | 0.86142857 | 0.29864253 | 0.13761468 | 0.30927835 | 0.07853403 | 0.15915119 |
| 64 | 1 | 0.3404908 | 0.24678663 | 0.85887755 | 0.23529412 | 0.1559633 | 0.38540841 | 0.12303665 | 0.06896552 |
| 65 | 1 | 0.46165644 | 0.46401028 | 0.89030612 | 0.41538462 | 0.25688073 | 0.35289453 | 0.10994764 | 0.02387268 |
| 66 | 1 | 0.43558282 | 0.18251928 | 0.53295918 | 0.30045249 | 0.57798165 | 0.52656622 | 0.20680628 | 0.20954907 |
| 67 | 1 | 0.59815951 | 0.60282776 | 0.89428571 | 0.67511312 | 0.13761468 | 0.45995242 | 0.22774869 | 0.0795756 |
| 68 | 1 | 0.41411043 | 0.20179949 | 0.95571429 | 0.34208145 | 0.26146789 | 0.38302934 | 0.33769634 | 0.25729443 |
| 69 | 1 | 0.23006135 | 0.08354756 | 0.50091837 | 0.22352941 | 0.08715596 | 0.34179223 | 0.19371728 | 0.16445623 |
| 70 | 1 | 0.21472393 | 0.09897172 | 0.70816327 | 0.18099548 | 0.1559633 | 0.39492466 | 0.09947644 | 0.0795756 |
| 71 | 1 | 0.29141104 | 0.33547558 | 0.80520408 | 0.52217195 | 0.09633028 | 0.43457573 | 0.10994764 | 0.04509284 |
| 72 | 1 | 0.23619632 | 0.20565553 | 0.89255102 | 0.27963801 | 0.1146789 | 0.37272006 | 0.08638743 | 0.04509284 |
| 73 | 1 | 0.14110429 | 0.13624679 | 0.91122449 | 0.20361991 | 0.05963303 | 0.28310864 | 0.06806283 | 0.03448276 |
| 74 | 1 | 0.31288344 | 0.11053985 | 0.85928571 | 0.17918552 | 0.33027523 | 0.42823156 | 0.15445026 | 0.08753316 |
| 75 | 1 | 0.4202454 | 0.31233933 | 0.83010204 | 0.48687783 | 0.18807339 | 0.40206186 | 0.15968586 | 0.10875332 |
| 76 | 1 | 0.28374233 | 0.20051414 | 0.81153061 | 0.30769231 | 0.04587156 | 0.37827121 | 0.2539267 | 0.18037135 |
| 77 | 1 | 0.20705521 | 0.17352185 | 0.95336735 | 0.35022624 | 0 | 0.41395718 | 0.12565445 | 0.05039788 |
| 78 | 1 | 0.17331288 | 0.2159383 | 0.76357143 | 0.23348416 | 0.16055046 | 0.36241079 | 0.06544503 | 0.0530504 |
| 79 | 1 | 0.39110429 | 0.24421594 | 0.88285714 | 0.32850679 | 0.21559633 | 0.52101507 | 0.22513089 | 0.20954907 |
| 80 | 1 | 0.33588957 | 0.14395887 | 0.90438776 | 0.27149321 | 0.21559633 | 0.41316416 | 0.21727749 | 0.14588859 |
| 81 | 1 | 0.38957055 | 0.16966581 | 0.87561224 | 0.32307692 | 0.11926606 | 0.43219667 | 0.30890052 | 0.23342175 |
| 82 | 1 | 0.22546012 | 0.30719794 | 0.78153061 | 0.3158371 | 0.16972477 | 0.29183188 | 0 | 0 |
| 83 | 1 | 0.62883436 | 0.32647815 | 0.8627551 | 0.46244344 | 0.37614679 | 0.59159397 | 0.43979058 | 0.4137931 |
| 84 | 1 | 0.29447853 | 0.20951157 | 0.87969388 | 0.23076923 | 0.20183486 | 0.40206186 | 0.14136126 | 0.0928382 |
| 85 | 1 | 0.26840491 | 0.07712082 | 0.98561224 | 0.19909502 | 0.18348624 | 0.33782712 | 0.15706806 | 0.1061008 |
| 86 | 1 | 0.24539877 | 0.28663239 | 0.83438776 | 0.35927602 | 0.19266055 | 0.3148295 | 0.07329843 | 0.05570292 |
| 87 | 1 | 0.3696319 | 0.23650386 | 0.82857143 | 0.32036199 | 0.16055046 | 0.38382236 | 0.17277487 | 0.11140584 |
| 88 | 1 | 0.4601227 | 0.35732648 | 0.95020408 | 0.43800905 | 0.23394495 | 0.4964314 | 0.34031414 | 0.28912467 |
| 89 | 1 | 0.38343558 | 0.19023136 | 0.92591837 | 0.29502262 | 0.34862385 | 0.42268041 | 0.19895288 | 0.12201592 |
| 90 | 1 | 0.39263804 | 0.28791774 | 0.85816327 | 0.46425339 | 0.29357798 | 0.43219667 | 0.08376963 | 0.04244032 |
| 91 | 1 | 0.12576687 | 0.39460154 | 0.6777551 | 0.39276018 | 0.06880734 | 0.37351308 | 0.01832461 | 0.05835544 |
| 92 | 1 | 0.29754601 | 0.3496144 | 0.56377551 | 0.33393665 | 0.14678899 | 0.36716891 | 0.05759162 | 0.03713528 |

| 93 | 1 | 0.42484663 | 0.1311054 | 0.93234694 | 0.20723982 | 0.21100917 | 0.5368755 | 0.28534031 | 0.23342175 |
| --- | --- | --- | --- | --- | --- | --- | --- | --- | --- |
| 94 | 1 | 0.34509202 | 0.21979434 | 0.83 | 0.29773756 | 0.22477064 | 0.4591594 | 0.27225131 | 0.25994695 |
| 95 | 1 | 0.2208589 | 0.19794344 | 0.84571429 | 0.25701357 | 0.04587156 | 0.38540841 | 0.13874346 | 0.12732095 |
| 96 | 1 | 0.23773006 | 0.27763496 | 0.86346939 | 0.3321267 | 0.14220183 | 0.27517843 | 0.07068063 | 0.0397878 |
| 97 | 1 | 0.1993865 | 0.28920308 | 0.67102041 | 0.34298643 | 0.09174312 | 0.26804124 | 0.11780105 | 0.10875332 |
| 98 | 1 | 0.30368098 | 0.29820051 | 0.75663265 | 0.37556561 | 0.08256881 | 0.36082474 | 0.13874346 | 0.08753316 |
| 99 | 1 | 0.25613497 | 0.34447301 | 0.96632653 | 0.4081448 | 0.15137615 | 0.43536875 | 0.10994764 | 0.06100796 |
| 100 | 1 | 0.29907975 | 0.41131105 | 0.83714286 | 0.42352941 | 0.14220183 | 0.28469469 | 0.14397906 | 0.10344828 |
| 101 | 1 | 0.32055215 | 0.16838046 | 0.88877551 | 0.34298643 | 0.26605505 | 0.33068993 | 0.12041885 | 0.10344828 |
| 102 | 1 | 0.30521472 | 0.30205656 | 0.86510204 | 0.31764706 | 0.12844037 | 0.41157811 | 0.07329843 | 0.04774536 |
| 103 | 1 | 0.52147239 | 0.16195373 | 0.93663265 | 0.23800905 | 0.66972477 | 0.50118953 | 0.2539267 | 0.20689655 |
| 104 | 1 | 0.2208589 | 0.16966581 | 0.7877551 | 0.31402715 | 0.02752294 | 0.31800159 | 0.09424084 | 0.09549072 |
| 105 | 1 | 0.48773006 | 0.1311054 | 0.75173469 | 0.22714932 | 0.40366972 | 0.66058684 | 0.2486911 | 0.16180371 |
| 106 | 1 | 0.32208589 | 0.13624679 | 0.92010204 | 0.22352941 | 0.27522936 | 0.47977795 | 0.22513089 | 0.15915119 |
| 107 | 1 | 0.35276074 | 0.12339332 | 0.98673469 | 0.33031674 | 0.35321101 | 0.25852498 | 0.2513089 | 0.25464191 |
| 108 | 1 | 0.38650307 | 0.21208226 | 0.80714286 | 0.28778281 | 0.18807339 | 0.49484536 | 0.30366492 | 0.27320955 |
| 109 | 1 | 0.52147239 | 0.09640103 | 0.85663265 | 0.33122172 | 0.19724771 | 0.5519429 | 0.58638743 | 0.55172414 |
| 110 | 1 | 0.23006135 | 0.10539846 | 0.83142857 | 0.20995475 | 0.06422018 | 0.31403648 | 0.14397906 | 0.18037135 |
| 111 | 1 | 0.28374233 | 0.06683805 | 0.88969388 | 0.21719457 | 0.23853211 | 0.39968279 | 0.09162304 | 0.066313 |
| 112 | 1 | 0.2101227 | 0.17609254 | 0.73714286 | 0.39366516 | 0.05504587 | 0.53211737 | 0.16230366 | 0.16445623 |
| 113 | 1 | 0.31595092 | 0.15681234 | 0.92479592 | 0.20361991 | 0.29357798 | 0.3259318 | 0.15183246 | 0.10344828 |
| 114 | 1 | 0.32515337 | 0.12339332 | 0.84612245 | 0.19819005 | 0.11926606 | 0.40285488 | 0.2513089 | 0.22281167 |
| 115 | 1 | 0.41104294 | 0.22365039 | 0.73806122 | 0.2959276 | 0.16055046 | 0.40602696 | 0.18062827 | 0.0928382 |
| 116 | 1 | 0.40337423 | 0.31105398 | 0.63816327 | 0.52488688 | 0.19266055 | 0.46788263 | 0.35863874 | 0.36339523 |
| 117 | 1 | 0.63803681 | 0.23007712 | 0.90285714 | 0.4081448 | 0.55504587 | 0.55670103 | 0.38743455 | 0.30769231 |
| 118 | 1 | 0.42944785 | 0.20565553 | 0.93989796 | 0.37466063 | 0.12385321 | 0.370341 | 0.2434555 | 0.1061008 |
| 119 | 1 | 0.41564417 | 0.27249357 | 0.80234694 | 0.32126697 | 0.21100917 | 0.41633624 | 0.27225131 | 0.23607427 |
| 120 | 1 | 0.33895706 | 0.37403599 | 0.8027551 | 0.44524887 | 0.13761468 | 0.41871531 | 0.18324607 | 0.14588859 |
| 121 | 1 | 0.30981595 | 0.24678663 | 0.91244898 | 0.25701357 | 0.25229358 | 0.45440127 | 0.14136126 | 0.11405836 |
| 122 | 1 | 0.68711656 | 0.1722365 | 0.8855102 | 0.35837104 | 0.30733945 | 0.50277557 | 0.35078534 | 0.23872679 |
| 123 | 1 | 0.50920245 | 0.24293059 | 0.875 | 0.38371041 | 0.33486239 | 0.48691515 | 0.32460733 | 0.22811671 |

| 124 | 1 | 0.24386503 | 0.2596401 | 0.70714286 | 0.36289593 | 0.1559633 | 0.32275971 | 0.11256545 | 0.0795756 |
| --- | --- | --- | --- | --- | --- | --- | --- | --- | --- |
| 125 | 1 | 0.39877301 | 0.15681234 | 0.77877551 | 0.28687783 | 0.21100917 | 0.42109437 | 0.23298429 | 0.19628647 |
| 126 | 1 | 0.31441718 | 0.14910026 | 0.62785714 | 0.31131222 | 0.18348624 | 0.37827121 | 0.23560209 | 0.20424403 |
| 127 | 1 | 0.31134969 | 0.19151671 | 0.75081633 | 0.24705882 | 0.19266055 | 0.50832672 | 0.13874346 | 0.11140584 |
| 128 | 1 | 0.22546012 | 0.11696658 | 0.6944898 | 0.29140271 | 0.25688073 | 0.36082474 | 0.12827225 | 0.12997347 |
| 129 | 1 | 0.49233129 | 0.24421594 | 0.97377551 | 0.3321267 | 0.36238532 | 0.50832672 | 0.20680628 | 0.09814324 |
| 130 | 1 | 0.47546012 | 0.22365039 | 0.95040816 | 0.45339367 | 0.13761468 | 0.36003172 | 0.32198953 | 0.23607427 |
| 131 | 1 | 0.49693252 | 0.18637532 | 0.89897959 | 0.34932127 | 0.18348624 | 0.51546392 | 0.34816754 | 0.33952255 |
| 132 | 1 | 0.64263804 | 0.16838046 | 0.91112245 | 0.48597285 | 0.17889908 | 0.63283109 | 0.7539267 | 0.69496021 |
| 133 | 1 | 0.36503067 | 0.2596401 | 0.90346939 | 0.35475113 | 0.16972477 | 0.36954798 | 0.23560209 | 0.16710875 |
| 134 | 1 | 0.47699387 | 0.24293059 | 0.94244898 | 0.37285068 | 0.24770642 | 0.51308485 | 0.35863874 | 0.25729443 |
| 135 | 1 | 0.4095092 | 0.21208226 | 0.61520408 | 0.30678733 | 0.12844037 | 0.56621729 | 0.22774869 | 0.13527851 |
| 136 | 1 | 0.27760736 | 0.18894602 | 0.84387755 | 0.27963801 | 0.14678899 | 0.42505948 | 0.2382199 | 0.19893899 |
| 137 | 1 | 0.37423313 | 0.16709512 | 0.73408163 | 0.43800905 | 0.22477064 | 0.34496431 | 0.31413613 | 0.33687003 |
| 138 | 1 | 0.3696319 | 0.17994859 | 0.85571429 | 0.34479638 | 0.20642202 | 0.43378271 | 0.23560209 | 0.14854111 |
| 139 | 1 | 0.43711656 | 0.3496144 | 0.81744898 | 0.39638009 | 0.25229358 | 0.49325932 | 0.2513089 | 0.15649867 |
| 140 | 1 | 0.30828221 | 0.24421594 | 0.86102041 | 0.28597285 | 0.2293578 | 0.41871531 | 0.14921466 | 0.07692308 |
| 141 | 1 | 0.29294479 | 0.18380463 | 0.60265306 | 0.24072398 | 0.23853211 | 0.52339413 | 0.2382199 | 0.19893899 |
| 142 | 1 | 0.80981595 | 0.32133676 | 0.89479592 | 0.38823529 | 0.55963303 | 0.64869151 | 0.59162304 | 0.47745358 |
| 143 | 1 | 0.35736196 | 0.1940874 | 0.95816327 | 0.29773756 | 0.22018349 | 0.38065028 | 0.2513089 | 0.21750663 |
| 144 | 1 | 0.49233129 | 0.23907455 | 0.94397959 | 0.34570136 | 0.35321101 | 0.53291039 | 0.22774869 | 0.14323607 |
| 145 | 1 | 0.26840491 | 0.25449871 | 0.9205102 | 0.32579186 | 0.16513761 | 0.35844568 | 0.13089005 | 0.12201592 |
| 146 | 1 | 0.31748466 | 0.22750643 | 0.6622449 | 0.36108597 | 0.08256881 | 0.35210151 | 0.29842932 | 0.27851459 |
| 147 | 1 | 0.35736196 | 0.22236504 | 0.87969388 | 0.35927602 | 0.25688073 | 0.49325932 | 0.12565445 | 0.10344828 |
| 148 | 1 | 0.32668712 | 0.20051414 | 0.96540816 | 0.3638009 | 0.18807339 | 0.34496431 | 0.28010471 | 0.32625995 |
| 149 | 1 | 0.21779141 | 0.24550129 | 0.86316327 | 0.27149321 | 0.0733945 | 0.34575734 | 0.15706806 | 0.14588859 |
| 150 | 1 | 0.33435583 | 0.16452442 | 0.78612245 | 0.26063348 | 0.08715596 | 0.3814433 | 0.28534031 | 0.22015915 |
| 151 | 1 | 0.53834356 | 0.28663239 | 0.84489796 | 0.51945701 | 0.14678899 | 0.54004758 | 0.52879581 | 0.45092838 |
| 152 | 1 | 0.2791411 | 0.18508997 | 0.78357143 | 0.29230769 | 0.11009174 | 0.39571768 | 0.16230366 | 0.18037135 |
| 153 | 1 | 0.30521472 | 0.24421594 | 0.77408163 | 0.37647059 | 0.08256881 | 0.35130849 | 0.21204188 | 0.16710875 |
| 154 | 1 | 0.42331288 | 0.17737789 | 0.74806122 | 0.29140271 | 0.2293578 | 0.40444092 | 0.37958115 | 0.34482759 |

| 155 | 1 | 0.45705521 | 0.16452442 | 0.91734694 | 0.40361991 | 0.05045872 | 0.39571768 | 0.5 | 0.59151194 |
| --- | --- | --- | --- | --- | --- | --- | --- | --- | --- |
| 156 | 1 | 0.50153374 | 0.20951157 | 0.72540816 | 0.3239819 | 0.33027523 | 0.70499603 | 0.34816754 | 0.27055703 |
| 157 | 1 | 0.38343558 | 0.13496144 | 0.6972449 | 0.27963801 | 0.05963303 | 0.45281523 | 0.39528796 | 0.38196286 |
| 158 | 1 | 0.23773006 | 0.18766067 | 0.68112245 | 0.2561086 | 0.21100917 | 0.3481364 | 0.07068063 | 0 |
| 159 | 1 | 0.56748466 | 0.21208226 | 0.93571429 | 0.32488688 | 0.33486239 | 0.55828707 | 0.4921466 | 0.37665782 |
| 160 | 1 | 0.31134969 | 0.16066838 | 0.67938776 | 0.27058824 | 0.20183486 | 0.5257732 | 0.21204188 | 0.14588859 |
| 161 | 1 | 0.23773006 | 0.32647815 | 0.92255102 | 0.28325792 | 0.05045872 | 0.31641554 | 0.04188482 | 0 |
| 162 | 1 | 0.6303681 | 0.31748072 | 0.90061224 | 0.3719457 | 0.32110092 | 0.48691515 | 0.2486911 | 0.10079576 |
| 163 | 1 | 0.30981595 | 0.26735219 | 0.85826531 | 0.26063348 | 0.26605505 | 0.4036479 | 0.12041885 | 0.07427056 |
| 164 | 1 | 0.34509202 | 0.30848329 | 0.80193878 | 0.32217195 | 0.10091743 | 0.38620143 | 0.13350785 | 0.09549072 |
| 165 | 1 | 0.35122699 | 0.20437018 | 0.93561224 | 0.2678733 | 0.14220183 | 0.38223632 | 0.27486911 | 0.22546419 |
| 166 | 1 | 0.21319018 | 0.04884319 | 0.78887755 | 0.24072398 | 0 | 0.43140365 | 0.13350785 | 0.23607427 |
| 167 | 1 | 0.61656442 | 0.281491 | 0.96010204 | 0.40995475 | 0.49541284 | 0.58207772 | 0.38481675 | 0.32625995 |
| 168 | 1 | 0.43865031 | 0.49614396 | 0.91418367 | 0.48959276 | 0.24770642 | 0.39333862 | 0.22513089 | 0.0928382 |
| 169 | 1 | 0.31595092 | 0.24678663 | 0.87683673 | 0.26063348 | 0.24770642 | 0.40047581 | 0.10471204 | 0.06896552 |
| 170 | 1 | 0.43711656 | 0.31233933 | 0.84734694 | 0.3918552 | 0.13302752 | 0.4147502 | 0.33507853 | 0.25994695 |
| 171 | 1 | 0.34509202 | 0.25706941 | 0.84806122 | 0.30859729 | 0.17889908 | 0.35527359 | 0.19895288 | 0.18037135 |
| 172 | 1 | 0.37423313 | 0.31876607 | 0.77561224 | 0.42533937 | 0.18807339 | 0.38461538 | 0.19633508 | 0.14058355 |
| 173 | 1 | 0.40184049 | 0.18508997 | 0.92520408 | 0.34027149 | 0.13761468 | 0.42743854 | 0.34816754 | 0.29708223 |
| 174 | 1 | 0.3404908 | 0.33933162 | 0.93408163 | 0.48778281 | 0.19266055 | 0.33862014 | 0.14921466 | 0.1061008 |
| 175 | 1 | 0.28834356 | 0.19537275 | 0.79071429 | 0.30497738 | 0.1559633 | 0.35210151 | 0.12041885 | 0.1061008 |
| 176 | 1 | 0.41564417 | 0.24164524 | 0.93204082 | 0.4 | 0.17889908 | 0.58762887 | 0.39005236 | 0.30769231 |
| 177 | 1 | 0.33282209 | 0.24421594 | 0.88102041 | 0.41176471 | 0.2706422 | 0.5035686 | 0.23298429 | 0.24137931 |
| 178 | 1 | 0.37576687 | 0.08997429 | 0.94642857 | 0.23167421 | 0.20642202 | 0.45678033 | 0.28795812 | 0.26525199 |
| 179 | 1 | 0.28067485 | 0.26478149 | 0.88673469 | 0.33755656 | 0.17889908 | 0.4258525 | 0.14397906 | 0.15384615 |
| 180 | 1 | 0.47699387 | 0.14138817 | 0.87193878 | 0.37285068 | 0.18807339 | 0.48770817 | 0.42931937 | 0.35543767 |
| 181 | 1 | 0.54601227 | 0.19023136 | 0.93010204 | 0.37828054 | 0.21100917 | 0.62648692 | 0.53926702 | 0.50928382 |
| 182 | 1 | 0.34662577 | 0.24550129 | 0.81408163 | 0.32850679 | 0.23394495 | 0.47581285 | 0.16753927 | 0.06100796 |
| 183 | 1 | 0.74693252 | 0.10796915 | 0.76734694 | 0.44072398 | 0.16972477 | 0.5852498 | 1 | 1 |
| 184 | 1 | 0.3696319 | 0.29691517 | 0.92091837 | 0.47420814 | 0.23853211 | 0.49881047 | 0.27486911 | 0.13793103 |
| 185 | 1 | 0.39110429 | 0.20822622 | 0.93877551 | 0.23891403 | 0.29816514 | 0.44171293 | 0.12041885 | 0.08488064 |

| 186 | 1 | 0.28527607 | 0.24678663 | 0.90734694 | 0.24343891 | 0.16513761 | 0.37509913 | 0.12565445 | 0.04774536 |
| --- | --- | --- | --- | --- | --- | --- | --- | --- | --- |
| 187 | 1 | 0.46625767 | 0.26092545 | 0.91846939 | 0.33393665 | 0.2293578 | 0.45360825 | 0.22251309 | 0.23872679 |
| 188 | 1 | 0.40797546 | 0.11439589 | 0.73408163 | 0.21357466 | 0.20183486 | 0.45678033 | 0.30890052 | 0.27320955 |
| 189 | 1 | 0.35122699 | 0.23264781 | 0.86897959 | 0.25701357 | 0.21559633 | 0.48770817 | 0.08376963 | 0.02122016 |
| 190 | 1 | 0.39877301 | 0.18251928 | 0.93071429 | 0.26425339 | 0.14220183 | 0.40523394 | 0.2460733 | 0.16710875 |
| 191 | 1 | 0.57822086 | 0.31233933 | 0.80897959 | 0.4959276 | 0.21559633 | 0.45122918 | 0.39267016 | 0.31564987 |
| 192 | 1 | 0.36809816 | 0.29948586 | 0.57234694 | 0.40180995 | 0.1559633 | 0.46788263 | 0.20418848 | 0.16710875 |
| 193 | 1 | 0.40644172 | 0.32647815 | 0.81142857 | 0.48868778 | 0.19266055 | 0.39492466 | 0.30628272 | 0.36604775 |
| 194 | 1 | 0.28834356 | 0.17866324 | 0.70510204 | 0.25701357 | 0.09174312 | 0.42030135 | 0.14397906 | 0.0928382 |
| 195 | 1 | 0.36196319 | 0.14652956 | 0.72173469 | 0.34660633 | 0.15137615 | 0.51704996 | 0.34031414 | 0.41114058 |
| 196 | 1 | 0.48312883 | 0.22622108 | 0.87020408 | 0.40271493 | 0.13761468 | 0.54242665 | 0.40837696 | 0.36074271 |
| 197 | 1 | 0.32208589 | 0.1092545 | 0.89663265 | 0.21266968 | 0.18348624 | 0.34099921 | 0.23560209 | 0.21220159 |
| 198 | 1 | 0.26687117 | 0.18508997 | 0.89459184 | 0.23076923 | 0.12385321 | 0.37351308 | 0.12565445 | 0.10344828 |
| 199 | 1 | 0.3404908 | 0.2596401 | 0.88755102 | 0.30226244 | 0.2293578 | 0.44964314 | 0.13612565 | 0.06896552 |
| 200 | 1 | 0.41717791 | 0.16323907 | 0.96744898 | 0.23800905 | 0.20183486 | 0.46153846 | 0.2408377 | 0.17241379 |
| 201 | 1 | 0.27607362 | 0.29434447 | 0.46020408 | 0.37647059 | 0.14678899 | 0.44964314 | 0.15183246 | 0.0530504 |
| 202 | 1 | 0.57361963 | 0.34061697 | 0.72183673 | 0.45520362 | 0.20642202 | 0.63917526 | 0.47382199 | 0.39257294 |
| 203 | 1 | 0.44018405 | 0.17609254 | 0.97316327 | 0.32126697 | 0.25688073 | 0.47977795 | 0.11518325 | 0.08222812 |
| 204 | 1 | 0.28834356 | 0.11825193 | 0.63377551 | 0.35656109 | 0.05963303 | 0.38540841 | 0.35602094 | 0.39522546 |
| 205 | 1 | 0.34815951 | 0.15424165 | 0.76857143 | 0.20542986 | 0.14220183 | 0.35844568 | 0.18324607 | 0.15915119 |
| 206 | 1 | 0.32822086 | 0.32262211 | 0.91653061 | 0.34751131 | 0.19724771 | 0.36478985 | 0.19109948 | 0.17241379 |
| 207 | 1 | 0.3190184 | 0.3277635 | 0.82377551 | 0.46425339 | 0.21100917 | 0.39333862 | 0.09424084 | 0.17506631 |
| 208 | 1 | 0.40184049 | 0.11825193 | 0.79591837 | 0.32126697 | 0.21100917 | 0.36954798 | 0.42670157 | 0.4403183 |
| 209 | 1 | 0.50613497 | 0.1092545 | 0.77877551 | 0.32760181 | 0.18807339 | 0.51308485 | 0.47120419 | 0.52785146 |
| 210 | 1 | 0.46165644 | 0.1529563 | 0.92061224 | 0.39547511 | 0.1559633 | 0.54956384 | 0.47643979 | 0.49602122 |
| 211 | 1 | 0.36349693 | 0.32647815 | 0.8944898 | 0.37466063 | 0.26605505 | 0.42902458 | 0.20157068 | 0.10344828 |
| 212 | 1 | 0.43865031 | 0.21208226 | 0.87357143 | 0.3520362 | 0.2706422 | 0.51308485 | 0.33246073 | 0.25464191 |
| 213 | 1 | 0.48159509 | 0.32647815 | 0.91020408 | 0.38642534 | 0.18348624 | 0.47977795 | 0.28272251 | 0.27055703 |
| 214 | 1 | 0.27147239 | 0.39203085 | 0.92540816 | 0.42895928 | 0.23394495 | 0.39016653 | 0.07853403 | 0.0530504 |
| 215 | 1 | 0.3404908 | 0.1066838 | 0.54122449 | 0.17556561 | 0.06422018 | 0.39571768 | 0.39790576 | 0.29442971 |
| 216 | 1 | 0.31288344 | 0.21208226 | 0.90346939 | 0.25067873 | 0.19266055 | 0.3814433 | 0.13612565 | 0.13527851 |

| 217 | 1 | 0.53680982 | 0.16323907 | 0.96510204 | 0.31945701 | 0.33486239 | 0.49881047 | 0.40052356 | 0.36339523 |
| --- | --- | --- | --- | --- | --- | --- | --- | --- | --- |
| 218 | 1 | 0.36809816 | 0.42673522 | 0.77336735 | 0.38552036 | 0.21559633 | 0.35527359 | 0.18062827 | 0.12466844 |
| 219 | 1 | 0.29447853 | 0.19151671 | 0.8394898 | 0.2678733 | 0.20642202 | 0.44250595 | 0.19109948 | 0.12732095 |
| 220 | 1 | 0.34815951 | 0.27763496 | 0.91040816 | 0.38190045 | 0.09633028 | 0.40206186 | 0.30628272 | 0.22546419 |
| 221 | 1 | 0.48773006 | 0.16580977 | 0.60581633 | 0.41809955 | 0.17431193 | 0.56463125 | 0.45549738 | 0.50132626 |
| 222 | 1 | 0.37883436 | 0.30976864 | 0.7327551 | 0.33393665 | 0.31192661 | 0.46708961 | 0.10209424 | 0.04774536 |
| 223 | 1 | 0.40030675 | 0.17994859 | 0.82938776 | 0.28597285 | 0.2293578 | 0.45836638 | 0.2565445 | 0.18302387 |
| 224 | 1 | 0.32208589 | 0.32390746 | 0.86469388 | 0.30497738 | 0.1146789 | 0.36875496 | 0.04188482 | 0.01591512 |
| 225 | 1 | 0.28067485 | 0.20179949 | 0.90612245 | 0.31945701 | 0.17889908 | 0.47660587 | 0.27486911 | 0.32891247 |
| 226 | 1 | 0.32668712 | 0.3470437 | 0.89397959 | 0.38642534 | 0.03211009 | 0.37747819 | 0.15706806 | 0.13793103 |
| 227 | 1 | 0.1993865 | 0.21336761 | 0.86673469 | 0.25701357 | 0.20642202 | 0.33465504 | 0.07329843 | 0.08488064 |
| 228 | 1 | 0.22852761 | 0.13624679 | 0.85632653 | 0.21176471 | 0.06422018 | 0.40444092 | 0.10994764 | 0.11140584 |
| 229 | 1 | 0.38496933 | 0.1285347 | 0.5344898 | 0.34117647 | 0.3440367 | 0.73037272 | 0.2408377 | 0.17771883 |
| 230 | 1 | 0.43865031 | 0.34832905 | 0.91163265 | 0.41176471 | 0.20183486 | 0.49960349 | 0.30628272 | 0.23342175 |
| 231 | 1 | 0.48619632 | 0.27249357 | 0.9122449 | 0.39457014 | 0.24311927 | 0.50436162 | 0.31413613 | 0.27055703 |
| 232 | 1 | 0.51993865 | 0.34318766 | 0.89020408 | 0.44524887 | 0.37155963 | 0.47501983 | 0.30628272 | 0.26790451 |
| 233 | 1 | 0.41257669 | 0.17994859 | 0.59897959 | 0.21447964 | 0.24770642 | 0.4520222 | 0.19633508 | 0.08488064 |
| 234 | 1 | 0.32055215 | 0.12596401 | 0.83765306 | 0.1918552 | 0.17431193 | 0.44329897 | 0.15706806 | 0.0795756 |
| 235 | 1 | 0.29601227 | 0.13881748 | 0.92357143 | 0.22171946 | 0.13302752 | 0.34099921 | 0.08376963 | 0.05039788 |
| 236 | 1 | 0.52607362 | 0.18637532 | 0.83714286 | 0.32036199 | 0.19266055 | 0.54639175 | 0.36910995 | 0.40318302 |
| 237 | 1 | 0.37269939 | 0.17866324 | 0.82367347 | 0.2841629 | 0.33944954 | 0.36161776 | 0.2486911 | 0.28381963 |
| 238 | 1 | 0.35429448 | 0.27892031 | 0.90785714 | 0.30497738 | 0.25688073 | 0.41395718 | 0.07329843 | 0.05570292 |
| 239 | 1 | 0.34355828 | 0.36632391 | 0.57959184 | 0.39004525 | 0.27981651 | 0.42030135 | 0.20418848 | 0.10875332 |
| 240 | 1 | 0.28374233 | 0.22365039 | 0.90979592 | 0.29864253 | 0.02752294 | 0.40681998 | 0.20418848 | 0.14588859 |
| 241 | 1 | 0.36656442 | 0.26478149 | 0.86306122 | 0.49230769 | 0.22477064 | 0.51625694 | 0.34554974 | 0.30503979 |
| 242 | 1 | 0.33588957 | 0.24935733 | 0.92806122 | 0.31040724 | 0.26605505 | 0.36716891 | 0.11780105 | 0.10875332 |
| 243 | 1 | 0.37116564 | 0.21336761 | 0.8405102 | 0.38280543 | 0.17431193 | 0.46153846 | 0.29581152 | 0.25994695 |
| 244 | 1 | 0.26380368 | 0.20308483 | 0.74479592 | 0.29140271 | 0.09633028 | 0.32910389 | 0.18848168 | 0.16976127 |
| 245 | 1 | 0.49079755 | 0.29048843 | 0.69969388 | 0.3438914 | 0.25229358 | 0.53846154 | 0.28010471 | 0.16976127 |
| 246 | 1 | 0.1993865 | 0.13624679 | 0.87112245 | 0.19457014 | 0.11009174 | 0.32989691 | 0.11518325 | 0.12732095 |
| 247 | 1 | 0.39417178 | 0.10282776 | 0.69397959 | 0.23257919 | 0.22018349 | 0.56621729 | 0.23036649 | 0.19098143 |

| 248 | 1 | 0.4708589 | 0.27249357 | 0.81520408 | 0.39276018 | 0.29816514 | 0.54401269 | 0.22251309 | 0.11140584 |
| --- | --- | --- | --- | --- | --- | --- | --- | --- | --- |
| 249 | 1 | 0.26533742 | 0.07455013 | 0.90265306 | 0.28959276 | 0.15137615 | 0.39095956 | 0.23560209 | 0.26259947 |
| 250 | 1 | 0.38190184 | 0.16709512 | 0.62 | 0.27058824 | 0.12844037 | 0.48929421 | 0.19895288 | 0.16976127 |
| 251 | 1 | 0.3190184 | 0.21979434 | 0.83377551 | 0.34027149 | 0.1146789 | 0.37430611 | 0.13089005 | 0.10875332 |
| 252 | 1 | 0.37883436 | 0.13753213 | 0.96346939 | 0.2561086 | 0.16513761 | 0.42823156 | 0.20680628 | 0.21750663 |
| 253 | 1 | 0.1993865 | 0.16323907 | 0.63040816 | 0.2479638 | 0.07798165 | 0.36558287 | 0.12827225 | 0.14058355 |
| 254 | 1 | 0.39723926 | 0.19151671 | 0.80846939 | 0.30859729 | 0.10091743 | 0.42981761 | 0.33769634 | 0.36604775 |
| 255 | 1 | 0.40030675 | 0.13624679 | 0.39510204 | 0.21900452 | 0.13302752 | 0.38382236 | 0.17801047 | 0.05039788 |
| 256 | 1 | 0.4493865 | 0.20437018 | 0.88581633 | 0.31312217 | 0.1559633 | 0.42347343 | 0.26439791 | 0.29442971 |
| 257 | 1 | 0.35276074 | 0.4151671 | 1 | 0.50497738 | 0.17889908 | 0.28310864 | 0.31937173 | 0.35013263 |
| 258 | 1 | 0.24539877 | 0.27892031 | 0.64979592 | 0.39366516 | 0.11926606 | 0.42030135 | 0.15183246 | 0.10079576 |
| 259 | 1 | 0.57822086 | 0.2596401 | 0.80408163 | 0.29411765 | 0.2706422 | 0.57256146 | 0.18848168 | 0.08753316 |
| 260 | 1 | 0.41871166 | 0.23136247 | 0.97204082 | 0.31312217 | 0.26605505 | 0.42902458 | 0.20418848 | 0.14323607 |
| 261 | 1 | 0.7101227 | 0.32904884 | 0.92489796 | 0.34570136 | 0.57798165 | 0.57335448 | 0.35340314 | 0.1061008 |
| 262 | 1 | 0.30981595 | 0.12082262 | 0.61530612 | 0.25882353 | 0.17889908 | 0.40285488 | 0.19371728 | 0.16976127 |
| 263 | 1 | 0.49233129 | 0.26092545 | 0.96255102 | 0.38190045 | 0.31192661 | 0.45519429 | 0.30890052 | 0.26790451 |
| 264 | 1 | 0.32208589 | 0.20179949 | 0.97857143 | 0.21176471 | 0.32110092 | 0.40761301 | 0.12303665 | 0.11405836 |
| 265 | 1 | 0.46932515 | 0.1311054 | 0.91540816 | 0.30859729 | 0.46788991 | 0.46788263 | 0.26701571 | 0.20954907 |
| 266 | 1 | 0.45552147 | 0.23007712 | 0.97255102 | 0.31674208 | 0.17889908 | 0.40761301 | 0.28010471 | 0.28381963 |
| 267 | 1 | 0.19171779 | 0.06169666 | 0.8277551 | 0.13122172 | 0.17889908 | 0.35210151 | 0.12565445 | 0.05039788 |
| 268 | 1 | 0.4493865 | 0.23393316 | 0.90755102 | 0.3520362 | 0.34862385 | 0.49881047 | 0.19109948 | 0.15649867 |
| 269 | 1 | 0.71319018 | 0.23136247 | 0.67581633 | 0.52579186 | 0.33944954 | 0.56145916 | 0.47120419 | 0.44827586 |
| 270 | 1 | 0.34815951 | 0.23521851 | 0.9722449 | 0.27058824 | 0.25229358 | 0.35130849 | 0.13089005 | 0.13262599 |
| 271 | 1 | 0.34815951 | 0.23136247 | 0.81285714 | 0.25701357 | 0.17431193 | 0.36003172 | 0.15968586 | 0.07427056 |
| 272 | 1 | 0.29447853 | 0.13624679 | 0.90153061 | 0.31131222 | 0.21100917 | 0.33624108 | 0.19109948 | 0.19363395 |
| 273 | 1 | 0.65030675 | 0.40102828 | 0.82867347 | 0.40633484 | 0.27522936 | 0.52418715 | 0.41099476 | 0.26525199 |
| 274 | 1 | 0.4202454 | 0.1092545 | 0.6177551 | 0.38642534 | 0.3440367 | 0.370341 | 0.2434555 | 0.14058355 |
| 275 | 1 | 0.37730061 | 0.17095116 | 0.74663265 | 0.41085973 | 0.21100917 | 0.45122918 | 0.2486911 | 0.24668435 |
| 276 | 1 | 0.28374233 | 0.17737789 | 0.79540816 | 0.24615385 | 0.09174312 | 0.39333862 | 0.18586387 | 0.19893899 |
| 277 | 1 | 0.36656442 | 0.10282776 | 0.20857143 | 0.16832579 | 0.36697248 | 0.48691515 | 0.16230366 | 0.15119363 |
| 278 | 1 | 0.37423313 | 0.30976864 | 0.84326531 | 0.31312217 | 0.20183486 | 0.50911975 | 0.06806283 | 0.02917772 |

| 279 | 1 | 0.38190184 | 0.23264781 | 0.86744898 | 0.32669683 | 0.27981651 | 0.4369548 | 0.2460733 | 0.18567639 |
| --- | --- | --- | --- | --- | --- | --- | --- | --- | --- |
| 280 | 1 | 0.44631902 | 0.31748072 | 0.96489796 | 0.42895928 | 0.17889908 | 0.51229183 | 0.37434555 | 0.31299735 |
| 281 | 1 | 0.54754601 | 0.79434447 | 0.66030612 | 0.68868778 | 0.11009174 | 0.54718477 | 0.2486911 | 0.22015915 |
| 282 | 1 | 0.40337423 | 0.3470437 | 0.92602041 | 0.39638009 | 0.24770642 | 0.38540841 | 0.31675393 | 0.19628647 |
| 283 | 1 | 0.57055215 | 0.21465296 | 0.97734694 | 0.42352941 | 0.35321101 | 0.41712926 | 0.33507853 | 0.22015915 |
| 284 | 1 | 0.19171779 | 0.12339332 | 0.93489796 | 0.2561086 | 0.09633028 | 0.36637589 | 0.13089005 | 0.16445623 |
| 285 | 1 | 0.40030675 | 0.1748072 | 0.81561224 | 0.30226244 | 0.1559633 | 0.48295004 | 0.2565445 | 0.22811671 |
| 286 | 1 | 0.27300613 | 0.32647815 | 0.70387755 | 0.32850679 | 0.05963303 | 0.40206186 | 0.11518325 | 0.12201592 |
| 287 | 1 | 0.42638037 | 0.20179949 | 0.76081633 | 0.29502262 | 0.27522936 | 0.42188739 | 0.15706806 | 0.09549072 |
| 288 | 1 | 0.49693252 | 0.25449871 | 0.65816327 | 0.30950226 | 0.31192661 | 0.53211737 | 0.11780105 | 0.0530504 |
| 289 | 1 | 0.51687117 | 0.20694087 | 0.92244898 | 0.36923077 | 0.19724771 | 0.45519429 | 0.46073298 | 0.4403183 |
| 290 | 1 | 0.48159509 | 0.12724936 | 0.9627551 | 0.29230769 | 0.16513761 | 0.49722443 | 0.4921466 | 0.45092838 |
| 291 | 1 | 0.28220859 | 0.27634961 | 0.83061224 | 0.33846154 | 0.14220183 | 0.38858049 | 0.16230366 | 0.10875332 |
| 292 | 1 | 0.40184049 | 0.23521851 | 0.92091837 | 0.32579186 | 0.29816514 | 0.42030135 | 0.08638743 | 0.03448276 |
| 293 | 1 | 0.32208589 | 0.29691517 | 0.94357143 | 0.32307692 | 0.23853211 | 0.34575734 | 0.16492147 | 0.16710875 |
| 294 | 1 | 0.43251534 | 0.38817481 | 0.35071429 | 0.41085973 | 0.2293578 | 0.56780333 | 0.26439791 | 0.13262599 |
| 295 | 1 | 0.25306748 | 0.26092545 | 0.98418367 | 0.30678733 | 0.17889908 | 0.39175258 | 0.10209424 | 0.10344828 |
| 296 | 1 | 0.29447853 | 0.14395887 | 0.89265306 | 0.25339367 | 0.09174312 | 0.36161776 | 0.21989529 | 0.22281167 |
| 297 | 1 | 0.49386503 | 0.1285347 | 0.80877551 | 0.31764706 | 0.2293578 | 0.53291039 | 0.43193717 | 0.41644562 |
| 298 | 1 | 0.60429448 | 0.29177378 | 0.74428571 | 0.43348416 | 0.22018349 | 0.53053132 | 0.56806283 | 0.45888594 |
| 299 | 1 | 0.32668712 | 0.35989717 | 0.9494898 | 0.39004525 | 0.19266055 | 0.44012688 | 0.21727749 | 0.17771883 |
| 300 | 1 | 0.6702454 | 0.281491 | 0.90673469 | 0.45972851 | 0.2706422 | 0.56225218 | 0.46335079 | 0.43766578 |
| 301 | 1 | 0.26993865 | 0.17737789 | 0.88153061 | 0.20723982 | 0.08715596 | 0.33624108 | 0.13612565 | 0.07692308 |
| 302 | 1 | 0.36042945 | 0.4151671 | 0.91591837 | 0.50316742 | 0.44036697 | 0.39175258 | 0.13350785 | 0.09814324 |
| 303 | 1 | 0.29907975 | 0.16966581 | 0.90826531 | 0.2678733 | 0.1146789 | 0.32910389 | 0.06544503 | 0.05039788 |
| 304 | 1 | 0.28527607 | 0.11825193 | 0.94653061 | 0.2 | 0.16055046 | 0.39809675 | 0.09162304 | 0.03448276 |
| 305 | 1 | 0.4309816 | 0.16580977 | 0.76204082 | 0.40090498 | 0.24311927 | 0.32513878 | 0.27225131 | 0.22281167 |
| 306 | 1 | 0.44478528 | 0.20951157 | 0.97142857 | 0.34479638 | 0.08256881 | 0.45995242 | 0.45811518 | 0.30503979 |
| 307 | 1 | 0.50613497 | 0.30719794 | 0.74071429 | 0.42352941 | 0.34862385 | 0.45836638 | 0.2539267 | 0.21750663 |
| 308 | 1 | 0.44018405 | 0.41645244 | 0.82183673 | 0.49411765 | 0.06422018 | 0.3925456 | 0.15706806 | 0.14854111 |
| 309 | 1 | 0.28680982 | 0.19537275 | 0.7305102 | 0.239819 | 0.17889908 | 0.3481364 | 0.14397906 | 0.12732095 |

| 310 | 1 | 0.38957055 | 0.14010283 | 0.85530612 | 0.38190045 | 0.35779817 | 0.46153846 | 0.26701571 | 0.22811671 |
| --- | --- | --- | --- | --- | --- | --- | --- | --- | --- |
| 311 | 1 | 0.41411043 | 0.13239075 | 0.82153061 | 0.25791855 | 0.16972477 | 0.53211737 | 0.37958115 | 0.31564987 |
| 312 | 1 | 0.6702454 | 0.18251928 | 0.69540816 | 0.53031674 | 0.18807339 | 0.50118953 | 0.7408377 | 0.63660477 |
| 313 | 1 | 0.49386503 | 0.20308483 | 0.77112245 | 0.30769231 | 0.25229358 | 0.4147502 | 0.2539267 | 0.16180371 |
| 314 | 1 | 0.2392638 | 0.26735219 | 0.74193878 | 0.35294118 | 0.05963303 | 0.43219667 | 0.06806283 | 0.06896552 |
| 315 | 1 | 0.72699387 | 0.11053985 | 0.83673469 | 0.40271493 | 0.06422018 | 0.53528945 | 0.79057592 | 0.91511936 |
| 316 | 1 | 0.56288344 | 0.17609254 | 0.58663265 | 0.34027149 | 0.27522936 | 0.54163362 | 0.31413613 | 0.18037135 |
| 317 | 1 | 0.53374233 | 0.58868895 | 0.92030612 | 0.42986425 | 0.46788991 | 0.42426646 | 0.17801047 | 0.04509284 |
| 318 | 1 | 0.52453988 | 0.32262211 | 0.86132653 | 0.40271493 | 0.31651376 | 0.57969865 | 0.39528796 | 0.36604775 |
| 319 | 1 | 0.39110429 | 0.38560411 | 0.62173469 | 0.40542986 | 0.1559633 | 0.42902458 | 0.20680628 | 0.22015915 |
| 320 | 1 | 0.29447853 | 0.35989717 | 0.73204082 | 0.33393665 | 0.13302752 | 0.37430611 | 0.03926702 | 0.0397878 |
| 321 | 1 | 0.38957055 | 0.2159383 | 0.81683673 | 0.38190045 | 0.14678899 | 0.43140365 | 0.2486911 | 0.15119363 |
| 322 | 1 | 0.29141104 | 0.13753213 | 0.86683673 | 0.31221719 | 0.05504587 | 0.49008723 | 0.40575916 | 0.37931034 |
| 323 | 1 | 0.43251534 | 0.26478149 | 0.93530612 | 0.32760181 | 0.24770642 | 0.48612213 | 0.31675393 | 0.21485411 |
| 324 | 1 | 0.29754601 | 0.38303342 | 0.85785714 | 0.43257919 | 0.11009174 | 0.2926249 | 0.07329843 | 0.06366048 |
| 325 | 1 | 0.45552147 | 0.25449871 | 0.94785714 | 0.39004525 | 0.20642202 | 0.57018239 | 0.28534031 | 0.20954907 |
| 326 | 1 | 0.52453988 | 0.1722365 | 0.68438776 | 0.28959276 | 0.2706422 | 0.58049167 | 0.39005236 | 0.35543767 |
| 327 | 1 | 0.4202454 | 0.33161954 | 0.87102041 | 0.51131222 | 0.03669725 | 0.40919905 | 0.23298429 | 0.1061008 |
| 328 | 1 | 0.49079755 | 0.28534704 | 0.90765306 | 0.3918552 | 0.06880734 | 0.43774782 | 0.51308901 | 0.46419098 |
| 329 | 1 | 0.1993865 | 0.16709512 | 0.5572449 | 0.21900452 | 0.0412844 | 0.3148295 | 0.13612565 | 0.11405836 |
| 330 | 1 | 0.37730061 | 0.3251928 | 0.87418367 | 0.31674208 | 0.20183486 | 0.41950833 | 0.12041885 | 0.1061008 |
| 331 | 1 | 0.51226994 | 0.19280206 | 1 | 0.2081448 | 0.21100917 | 0.59397304 | 0.21204188 | 0.21485411 |
| 332 | 1 | 0.41871166 | 0.22107969 | 0.74663265 | 0.32850679 | 0.14220183 | 0.52894528 | 0.13089005 | 0.05570292 |
| 333 | 1 | 0.51380368 | 0.14010283 | 0.75755102 | 0.40180995 | 0.20183486 | 0.57652657 | 0.40052356 | 0.25198939 |
| 334 | 1 | 0.34355828 | 0.38046272 | 0.81602041 | 0.43438914 | 0.18348624 | 0.40602696 | 0.16753927 | 0.14854111 |
| 335 | 1 | 0.5690184 | 0.34190231 | 0.8494898 | 0.49321267 | 0.25688073 | 0.5368755 | 0.33507853 | 0.29708223 |
| 336 | 1 | 0.38650307 | 0.36246787 | 0.92295918 | 0.37285068 | 0.28899083 | 0.43536875 | 0.10732984 | 0.05039788 |
| 337 | 1 | 0.43404908 | 0.36760925 | 0.68561224 | 0.45248869 | 0.22018349 | 0.46708961 | 0.37958115 | 0.29442971 |
| 338 | 1 | 0.3404908 | 0.17866324 | 0.97091837 | 0.27692308 | 0.50917431 | 0.43536875 | 0.07329843 | 0.05570292 |
| 339 | 1 | 0.50153374 | 0.26349614 | 0.9744898 | 0.29773756 | 0.22018349 | 0.45678033 | 0.28010471 | 0.19628647 |
| 340 | 1 | 0.54601227 | 0.23264781 | 0.8644898 | 0.30859729 | 0.3440367 | 0.50277557 | 0.2539267 | 0.15384615 |

| 341 | 1 | 0.42638037 | 0.26992288 | 0.66693878 | 0.36923077 | 0.28440367 | 0.47501983 | 0.22513089 | 0.22811671 |
| --- | --- | --- | --- | --- | --- | --- | --- | --- | --- |
| 342 | 1 | 0.22852761 | 0 | 0.92938776 | 0.05429864 | 0.27522936 | 0.37747819 | 0 | 0.0795756 |
| 343 | 1 | 0.37883436 | 0.2159383 | 0.92938776 | 0.29864253 | 0.15137615 | 0.43219667 | 0.23560209 | 0.20424403 |
| 344 | 1 | 0.34355828 | 0.11953728 | 0.87418367 | 0.3040724 | 0.16972477 | 0.6518636 | 0.34293194 | 0.29708223 |
| 345 | 1 | 0.4493865 | 0.28534704 | 0.89846939 | 0.36832579 | 0.2293578 | 0.54401269 | 0.41099476 | 0.26525199 |
| 346 | 1 | 0.40030675 | 0.20694087 | 0.84326531 | 0.27873303 | 0.29816514 | 0.49088025 | 0.2539267 | 0.20159151 |
| 347 | 1 | 0.34969325 | 0.27634961 | 0.93040816 | 0.32488688 | 0.18807339 | 0.37192704 | 0.18062827 | 0.17771883 |
| 348 | 1 | 0.28220859 | 0.11825193 | 0.8227551 | 0.15113122 | 0.30733945 | 0.34417129 | 0.06544503 | 0.07692308 |
| 349 | 1 | 0.14877301 | 0.17737789 | 0.82020408 | 0.20995475 | 0.08256881 | 0.27914354 | 0.09685864 | 0.06100796 |
| 350 | 1 | 0.1809816 | 0.2403599 | 0.71173469 | 0.2760181 | 0.13302752 | 0.36637589 | 0.05235602 | 0 |
| 351 | 1 | 0.30981595 | 0.14395887 | 0.82520408 | 0.22533937 | 0.23394495 | 0.36954798 | 0.20680628 | 0.13527851 |
| 352 | 1 | 0.34509202 | 0.2159383 | 0.75765306 | 0.26696833 | 0.2293578 | 0.44250595 | 0.15968586 | 0.08753316 |
| 353 | 1 | 0.31748466 | 0.22750643 | 0.81469388 | 0.30226244 | 0.16055046 | 0.38223632 | 0.14659686 | 0.1193634 |
| 354 | 1 | 0.32055215 | 0.12210797 | 0.96081633 | 0.31493213 | 0.20183486 | 0.33624108 | 0.19895288 | 0.23607427 |
| 355 | 1 | 0.4309816 | 0.21465296 | 0.9022449 | 0.31945701 | 0.12385321 | 0.35210151 | 0.27748691 | 0.18037135 |
| 356 | 1 | 0.36196319 | 0.24807198 | 0.85438776 | 0.32217195 | 0.19724771 | 0.41950833 | 0.10732984 | 0.0265252 |
| 357 | 1 | 0.25153374 | 0.11568123 | 0.83489796 | 0.39909502 | 0.03211009 | 0.39095956 | 0.15706806 | 0.13793103 |
| 358 | 1 | 0.30981595 | 0.21722365 | 0.88867347 | 0.30497738 | 0.34862385 | 0.46153846 | 0.17539267 | 0.15649867 |
| 359 | 1 | 0.39417178 | 0.22879177 | 0.86122449 | 0.31764706 | 0.43119266 | 0.4742268 | 0.27486911 | 0.27851459 |
| 360 | 1 | 0.59355828 | 0.31105398 | 0.94561224 | 0.45520362 | 0.31192661 | 0.46788263 | 0.42931937 | 0.30769231 |
| 361 | 1 | 0.29754601 | 0.22107969 | 0.82285714 | 0.22624434 | 0.19266055 | 0.4369548 | 0.10209424 | 0.05835544 |
| 362 | 1 | 0.13803681 | 0.15809769 | 0.82122449 | 0.20271493 | 0.07798165 | 0.20935765 | 0.07329843 | 0.07427056 |
| 363 | 1 | 0.30674847 | 0.16709512 | 0.59102041 | 0.26877828 | 0.18348624 | 0.39809675 | 0.20418848 | 0.15915119 |
| 364 | 1 | 0.40184049 | 0.2159383 | 0.83938776 | 0.3438914 | 0.26146789 | 0.49405234 | 0.26963351 | 0.18302387 |
| 365 | 1 | 0.26687117 | 0.23521851 | 0.47418367 | 0.26425339 | 0.13302752 | 0.37668517 | 0.10209424 | 0.06100796 |
| 366 | 1 | 0.32361963 | 0.20694087 | 0.92030612 | 0.28868778 | 0.19724771 | 0.40126883 | 0.17539267 | 0.1193634 |
| 367 | 1 | 0.49693252 | 0.27763496 | 0.88755102 | 0.41809955 | 0.18807339 | 0.61934972 | 0.5104712 | 0.44827586 |
| 368 | 1 | 0.48773006 | 0.3470437 | 0.74316327 | 0.40361991 | 0.21559633 | 0.43457573 | 0.32722513 | 0.27055703 |
| 369 | 1 | 0.3803681 | 0.21208226 | 0.89071429 | 0.27239819 | 0.16055046 | 0.39730373 | 0.17015707 | 0.12466844 |
| 370 | 1 | 0.41411043 | 0.2377892 | 0.7644898 | 0.30678733 | 0.29816514 | 0.3592387 | 0.31413613 | 0.26525199 |
| 371 | 1 | 0.3006135 | 0.08611825 | 0.95683673 | 0.21719457 | 0.07798165 | 0.34099921 | 0.21989529 | 0.23607427 |

| 372 | 1 | 0.44171779 | 0.16066838 | 0.92877551 | 0.27511312 | 0.17889908 | 0.370341 | 0.2434555 | 0.26790451 |
| --- | --- | --- | --- | --- | --- | --- | --- | --- | --- |
| 373 | 1 | 0.26226994 | 0.16195373 | 0.80428571 | 0.26877828 | 0.0412844 | 0.43536875 | 0.11780105 | 0.02387268 |
| 374 | 1 | 0.29294479 | 0.15424165 | 0.85979592 | 0.29230769 | 0.11926606 | 0.42505948 | 0.19895288 | 0.17771883 |
| 375 | 1 | 0.37116564 | 0.34832905 | 0.92510204 | 0.35022624 | 0.22018349 | 0.36082474 | 0.22774869 | 0.15915119 |
| 376 | 1 | 0.26687117 | 0.42159383 | 0.87897959 | 0.3638009 | 0.09174312 | 0.24980174 | 0.07068063 | 0.01856764 |
| 377 | 1 | 0.3297546 | 0.27634961 | 0.95153061 | 0.35656109 | 0 | 0.28390167 | 0.18848168 | 0.19098143 |
| 378 | 1 | 0.32668712 | 0.37017995 | 0.735 | 0.40723982 | 0.24311927 | 0.40999207 | 0.12303665 | 0.07692308 |
| 379 | 1 | 0.30214724 | 0.23264781 | 0.7505102 | 0.31855204 | 0.16513761 | 0.39095956 | 0.17277487 | 0.12466844 |
| 380 | 1 | 0.35429448 | 0.29691517 | 0.94867347 | 0.34751131 | 0.31192661 | 0.39333862 | 0.14921466 | 0.11140584 |
| 381 | 1 | 0.4309816 | 0.32005141 | 0.96102041 | 0.47963801 | 0.28899083 | 0.32831086 | 0.18324607 | 0.16710875 |
| 382 | 1 | 0.32055215 | 0.17866324 | 0.85897959 | 0.30226244 | 0.09174312 | 0.36241079 | 0.21204188 | 0.17771883 |
| 383 | 1 | 0.38190184 | 0.23393316 | 0.935 | 0.35837104 | 0.23394495 | 0.45360825 | 0.29319372 | 0.33952255 |
| 384 | 1 | 0.17331288 | 0.28277635 | 0.92244898 | 0.29230769 | 0.09174312 | 0.27993656 | 0.12041885 | 0.12201592 |
| 385 | 1 | 0.43865031 | 0.2596401 | 0.96153061 | 0.43710407 | 0.16055046 | 0.38540841 | 0.34293194 | 0.25994695 |
| 386 | 1 | 0.34509202 | 0.3470437 | 0.44734694 | 0.3800905 | 0.2293578 | 0.41316416 | 0.15706806 | 0.066313 |
| 387 | 1 | 0.42177914 | 0.16838046 | 0.7777551 | 0.34751131 | 0.16972477 | 0.46946868 | 0.36910995 | 0.38196286 |
| 388 | 1 | 0.29601227 | 0.21722365 | 0.69193878 | 0.39819005 | 0.1559633 | 0.33862014 | 0.23298429 | 0.15119363 |
| 389 | 1 | 0.40797546 | 0.16709512 | 0.95826531 | 0.35565611 | 0.14220183 | 0.47898493 | 0.32722513 | 0.32625995 |
| 390 | 1 | 0.38496933 | 0.12724936 | 0.83040816 | 0.19547511 | 0.18807339 | 0.36399683 | 0.22251309 | 0.08488064 |
| 391 | 1 | 0.50920245 | 0.44215938 | 0.89561224 | 0.4561086 | 0.44954128 | 0.5368755 | 0.29057592 | 0.22811671 |
| 392 | 1 | 0.64877301 | 0.19280206 | 0.96153061 | 0.33665158 | 0.19724771 | 0.49960349 | 0.5052356 | 0.57029178 |
| 393 | 1 | 0.47392638 | 0.16966581 | 0.83183673 | 0.3438914 | 0.26605505 | 0.5630452 | 0.36125654 | 0.29442971 |
| 394 | 1 | 0.51226994 | 0.19151671 | 0.85765306 | 0.29230769 | 0.38073394 | 0.64631245 | 0.31413613 | 0.29177719 |
| 395 | 1 | 0.49386503 | 0.20565553 | 0.86530612 | 0.34117647 | 0.29816514 | 0.54956384 | 0.34816754 | 0.27851459 |
| 396 | 1 | 0.39723926 | 0.16966581 | 0.90887755 | 0.30859729 | 0.25229358 | 0.39571768 | 0.21465969 | 0.14058355 |
| 397 | 1 | 0.35276074 | 0.26735219 | 0.98489796 | 0.35565611 | 0.24770642 | 0.36478985 | 0.07068063 | 0.02387268 |
| 398 | 1 | 0.41411043 | 0.30848329 | 0.73836735 | 0.37828054 | 0.1146789 | 0.51149881 | 0.36649215 | 0.36604775 |
| 399 | 1 | 0.49386503 | 0.1940874 | 0.85581633 | 0.34479638 | 0.2706422 | 0.44409199 | 0.35078534 | 0.29442971 |
| 400 | 1 | 0.34355828 | 0.35218509 | 0.76265306 | 0.50769231 | 0.16972477 | 0.39095956 | 0.19633508 | 0.14854111 |
| 401 | 1 | 0.30674847 | 0.12339332 | 0.95306122 | 0.24705882 | 0.14678899 | 0.36478985 | 0.20942408 | 0.14854111 |
| 402 | 1 | 0.24233129 | 0.16709512 | 0.78510204 | 0.28868778 | 0.05504587 | 0.34417129 | 0.2486911 | 0.18832891 |

| 403 | 1 | 0.52607362 | 0.33033419 | 0.90673469 | 0.33574661 | 0.33486239 | 0.53846154 | 0.23560209 | 0.19098143 |
| --- | --- | --- | --- | --- | --- | --- | --- | --- | --- |
| 404 | 1 | 0.41411043 | 0.23264781 | 0.92765306 | 0.31221719 | 0.10091743 | 0.42505948 | 0.39528796 | 0.27055703 |
| 405 | 1 | 0.42944785 | 0.20822622 | 0.60704082 | 0.38371041 | 0.32568807 | 0.51942902 | 0.31675393 | 0.25464191 |
| 406 | 1 | 0.32361963 | 0.46272494 | 0.60142857 | 0.47058824 | 0.22477064 | 0.41633624 | 0.17277487 | 0.14854111 |
| 407 | 1 | 0.37883436 | 0.1285347 | 0.88255102 | 0.28868778 | 0.16972477 | 0.49563838 | 0.34554974 | 0.37135279 |
| 408 | 1 | 0.38957055 | 0.28020566 | 0.89683673 | 0.39819005 | 0.14220183 | 0.39016653 | 0.28534031 | 0.29973475 |
| 409 | 1 | 0.26533742 | 0.22236504 | 0.58265306 | 0.32307692 | 0.21100917 | 0.45360825 | 0.09685864 | 0.07427056 |
| 410 | 1 | 0.28220859 | 0.21336761 | 0.69683673 | 0.2959276 | 0.13302752 | 0.3592387 | 0.14397906 | 0.14588859 |
| 411 | 1 | 0.25153374 | 0.15424165 | 0.83765306 | 0.32307692 | 0.10550459 | 0.45678033 | 0.16492147 | 0.17241379 |
| 412 | 1 | 0.45245399 | 0.12596401 | 0.45387755 | 0.46425339 | 0.2293578 | 0.45598731 | 0.34554974 | 0.25464191 |
| 413 | 1 | 0.32055215 | 0.20179949 | 0.73234694 | 0.32850679 | 0.14678899 | 0.42902458 | 0.20680628 | 0.22015915 |
| 414 | 1 | 0.55828221 | 0.22236504 | 0.87408163 | 0.34117647 | 0.23394495 | 0.52180809 | 0.45549738 | 0.40583554 |
| 415 | 1 | 0.36656442 | 0.08868895 | 0.82653061 | 0.29049774 | 0.09174312 | 0.35844568 | 0.39528796 | 0.4137931 |
| 416 | 1 | 0.45858896 | 0.21336761 | 0.88479592 | 0.27420814 | 0.34862385 | 0.56780333 | 0.30104712 | 0.20954907 |
| 417 | 1 | 0.3297546 | 0.26863753 | 0.72632653 | 0.34479638 | 0.22018349 | 0.45519429 | 0.26701571 | 0.16976127 |
| 418 | 1 | 0.34202454 | 0.24293059 | 0.91142857 | 0.28325792 | 0.21100917 | 0.32672482 | 0.21989529 | 0.17506631 |
| 419 | 1 | 0.23006135 | 0.18508997 | 0.71377551 | 0.29321267 | 0.10091743 | 0.3592387 | 0.19371728 | 0.18832891 |
| 420 | 1 | 0.36042945 | 0.27634961 | 0.87173469 | 0.34117647 | 0.12844037 | 0.43140365 | 0.19633508 | 0.17241379 |
| 421 | 1 | 0.45552147 | 0.18380463 | 0.87091837 | 0.45701357 | 0.1559633 | 0.44488501 | 0.46858639 | 0.45888594 |
| 422 | 1 | 0.44785276 | 0.14010283 | 0.68591837 | 0.23348416 | 0.37614679 | 0.51149881 | 0.12565445 | 0.10875332 |
| 423 | 1 | 0.13803681 | 0.34832905 | 0.8305102 | 0.32760181 | 0 | 0.28707375 | 0.14136126 | 0 |
| 424 | 1 | 0.41257669 | 0.3714653 | 0.93428571 | 0.32850679 | 0.13761468 | 0.42268041 | 0.15706806 | 0.066313 |
| 425 | 1 | 0.34355828 | 0.12982005 | 0.84408163 | 0.22262443 | 0.22477064 | 0.45440127 | 0.14921466 | 0.12201592 |
| 426 | 1 | 0.30674847 | 0.21722365 | 0.81285714 | 0.23710407 | 0.21100917 | 0.32989691 | 0.12041885 | 0.08222812 |
| 427 | 1 | 0.35429448 | 0.03727506 | 0.99102041 | 0.13122172 | 0 | 0.64155432 | 0.22774869 | 0.15384615 |
| 428 | 1 | 0.34509202 | 0.03598972 | 0.89163265 | 0.17828054 | 0.12844037 | 0.51387787 | 0.07329843 | 0.07427056 |
| 429 | 1 | 0.26687117 | 0.2159383 | 0.73010204 | 0.29683258 | 0.27522936 | 0.4147502 | 0.10471204 | 0.09018568 |
| 430 | 1 | 0.53527607 | 0.45244216 | 0.87877551 | 0.52579186 | 0.18807339 | 0.56225218 | 0.13612565 | 0.11671088 |
| 431 | 1 | 0.40184049 | 0.28791774 | 0.51336735 | 0.60904977 | 0.05504587 | 0.60348929 | 0.39267016 | 0.29708223 |
| 432 | 1 | 0.36196319 | 0.14652956 | 0.64428571 | 0.25972851 | 0.23394495 | 0.48691515 | 0.2460733 | 0.20954907 |
| 433 | 1 | 0.22392638 | 0.04755784 | 0.69918367 | 0.16199095 | 0.18807339 | 0.34655036 | 0.13089005 | 0.10875332 |

| 434 | 1 | 0.28680982 | 0.15681234 | 0.52285714 | 0.2081448 | 0.36238532 | 0.3814433 | 0.09424084 | 0.03713528 |
| --- | --- | --- | --- | --- | --- | --- | --- | --- | --- |
| 435 | 1 | 0.5398773 | 0.18894602 | 1 | 0.35565611 | 0.60091743 | 0.46788263 | 0.23560209 | 0.10875332 |
| 436 | 1 | 0.34815951 | 0.16195373 | 0.83857143 | 0.36561086 | 0.1146789 | 0.34020619 | 0.39790576 | 0.46949602 |
| 437 | 1 | 0.41104294 | 0.30077121 | 0.9105102 | 0.31945701 | 0.38990826 | 0.51546392 | 0.14921466 | 0.09018568 |
| 438 | 1 | 0.3404908 | 0.39845758 | 0.45193878 | 0.43257919 | 0.30275229 | 0.47184774 | 0.10209424 | 0.03183024 |
| 439 | 1 | 0.11656442 | 0.19537275 | 0.91795918 | 0.3438914 | 0.08715596 | 0.28628073 | 0.14921466 | 0.15119363 |
| 440 | 1 | 0.20705521 | 0.14910026 | 0.95714286 | 0.20361991 | 0.08715596 | 0.28548771 | 0.16753927 | 0.13527851 |
| 441 | 1 | 0.30521472 | 0.18508997 | 0.63857143 | 0.28506787 | 0.18807339 | 0.43457573 | 0.19633508 | 0.16445623 |
| 442 | 1 | 0.36809816 | 0.09897172 | 0.59612245 | 0.2561086 | 0.11926606 | 0.39413164 | 0.08900524 | 0.06896552 |
| 443 | 1 | 0.39570552 | 0.33161954 | 0.78204082 | 0.57828054 | 0.1559633 | 0.32910389 | 0.08900524 | 0.02917772 |
| 444 | 1 | 0.15030675 | 0.1529563 | 0.98020408 | 0.17737557 | 0.04587156 | 0.3148295 | 0.05497382 | 0.0397878 |
| 445 | 1 | 0.3696319 | 0.2159383 | 0.7594898 | 0.31493213 | 0.05045872 | 0.36954798 | 0.35340314 | 0.32625995 |
| 446 | 1 | 0.25153374 | 0.25192802 | 0.52459184 | 0.3040724 | 0.17889908 | 0.36637589 | 0.12303665 | 0.14588859 |
| 447 | 1 | 0.37883436 | 0.22365039 | 0.93081633 | 0.19004525 | 0.25229358 | 0.45678033 | 0.04712042 | 0 |
| 448 | 1 | 0.33588957 | 0.30848329 | 0.68489796 | 0.37013575 | 0.27522936 | 0.51942902 | 0.14921466 | 0.07161804 |
| 449 | 1 | 0.45552147 | 0.16580977 | 0.96 | 0.26153846 | 0.21100917 | 0.36241079 | 0.13874346 | 0.0397878 |
| 450 | 1 | 0.23466258 | 0.1311054 | 0.66479592 | 0.22171946 | 0.09174312 | 0.33068993 | 0.10994764 | 0.08753316 |
| 451 | 1 | 0.63957055 | 0.24164524 | 0.95377551 | 0.38552036 | 0.38073394 | 0.51625694 | 0.39528796 | 0.32891247 |
| 452 | 1 | 0.43558282 | 0.23007712 | 0.93857143 | 0.34298643 | 0.26605505 | 0.4591594 | 0.20418848 | 0.16976127 |
| 453 | 1 | 0.56134969 | 0.36246787 | 0.9294898 | 0.47963801 | 0.45412844 | 0.55114988 | 0.35863874 | 0.28912467 |
| 454 | 1 | 0.37576687 | 0.18251928 | 0.49744898 | 0.21900452 | 0.20642202 | 0.45043616 | 0.26963351 | 0.15384615 |
| 455 | 1 | 0.37883436 | 0.15552699 | 0.75204082 | 0.29140271 | 0.10550459 | 0.47343378 | 0.29319372 | 0.31034483 |
| 456 | 1 | 0.25 | 0.33161954 | 0.70795918 | 0.37647059 | 0.14678899 | 0.3259318 | 0.09685864 | 0.04244032 |
| 457 | 1 | 0.42638037 | 0.22622108 | 0.79642857 | 0.36561086 | 0.18807339 | 0.48215702 | 0.29842932 | 0.20689655 |
| 458 | 1 | 0.37883436 | 0.31233933 | 0.92367347 | 0.30769231 | 0.16055046 | 0.46550357 | 0.19895288 | 0.11140584 |
| 459 | 1 | 0.22392638 | 0.22107969 | 0.76081633 | 0.2959276 | 0.07798165 | 0.33465504 | 0.13612565 | 0.13793103 |
| 460 | 1 | 0.3190184 | 0.12339332 | 0.75704082 | 0.14479638 | 0.16972477 | 0.44409199 | 0.20942408 | 0.09814324 |
| 461 | 1 | 0.34969325 | 0.1966581 | 0.91193878 | 0.3520362 | 0.09633028 | 0.39968279 | 0.2513089 | 0.22811671 |
| 462 | 1 | 0.24693252 | 0.29948586 | 0.96581633 | 0.31855204 | 0.23394495 | 0.36716891 | 0.08900524 | 0.09018568 |
| 463 | 1 | 0.30981595 | 0.18123393 | 0.83846939 | 0.27692308 | 0.15137615 | 0.49960349 | 0.17277487 | 0.14854111 |
| 464 | 1 | 0.19478528 | 0.24807198 | 0.89642857 | 0.29773756 | 0.06422018 | 0.32434576 | 0.08638743 | 0.08753316 |

| 465 | 1 | 0.23466258 | 0.08097686 | 0.67 | 0.18733032 | 0.08256881 | 0.5852498 | 0.18848168 | 0.19098143 |
| --- | --- | --- | --- | --- | --- | --- | --- | --- | --- |
| 466 | 1 | 0.46625767 | 0.4125964 | 0.83295918 | 0.39638009 | 0.21100917 | 0.55590801 | 0.17015707 | 0.10875332 |
| 467 | 1 | 0.1809816 | 0.10025707 | 0.60989796 | 0.34932127 | 0.12385321 | 0.35448057 | 0.09424084 | 0.07161804 |
| 468 | 1 | 0.4708589 | 0.12082262 | 0.8355102 | 0.3321267 | 0.40825688 | 0.55352895 | 0.39005236 | 0.26259947 |
| 469 | 1 | 0.33742331 | 0.27377892 | 0.76479592 | 0.35113122 | 0.16055046 | 0.40206186 | 0.20942408 | 0.15649867 |
| 470 | 1 | 0.30981595 | 0.22107969 | 0.9477551 | 0.36470588 | 0.13761468 | 0.30452022 | 0.13612565 | 0.14854111 |
| 471 | 1 | 0.3696319 | 0.20179949 | 0.91744898 | 0.29411765 | 0.33486239 | 0.44488501 | 0.23298429 | 0.26525199 |
| 472 | 1 | 0.54754601 | 0.19794344 | 0.81306122 | 0.2841629 | 0.2706422 | 0.42188739 | 0.39005236 | 0.22546419 |
| 473 | 1 | 0.2791411 | 0.07326478 | 0.86806122 | 0.11312217 | 0.10550459 | 0.37827121 | 0.20942408 | 0.21220159 |
| 474 | 1 | 0.35122699 | 0.07712082 | 0.60306122 | 0.26606335 | 0.2293578 | 0.4147502 | 0.2408377 | 0.23076923 |
| 475 | 1 | 0.35736196 | 0.16580977 | 0.63479592 | 0.23438914 | 0.16972477 | 0.35765266 | 0.23560209 | 0.18037135 |
| 476 | 1 | 0.1993865 | 0.20437018 | 0.97612245 | 0.21357466 | 0.04587156 | 0.37430611 | 0.10209424 | 0.06366048 |
| 477 | 1 | 0.2791411 | 0.39845758 | 0.50397959 | 0.38371041 | 0.11926606 | 0.33465504 | 0.12041885 | 0.04774536 |
| 478 | 1 | 0.33895706 | 0.14138817 | 0.88357143 | 0.26606335 | 0.10091743 | 0.42823156 | 0.23036649 | 0.14588859 |
| 479 | 1 | 0.5506135 | 0.34190231 | 0.85918367 | 0.43710407 | 0.12385321 | 0.50436162 | 0.61256545 | 0.63395225 |
| 480 | 1 | 0.51533742 | 0.26863753 | 0.91336735 | 0.38099548 | 0.23394495 | 0.46867565 | 0.34031414 | 0.30503979 |
| 481 | 1 | 0.2208589 | 0.10539846 | 0.72683673 | 0.16742081 | 0.09633028 | 0.56938937 | 0.14397906 | 0.12732095 |
| 482 | 1 | 0.40644172 | 0.1748072 | 0.70785714 | 0.28054299 | 0.44495413 | 0.65503569 | 0.11780105 | 0.05039788 |
| 483 | 1 | 0.26687117 | 0.30077121 | 0.76632653 | 0.39638009 | 0.17431193 | 0.35606661 | 0.07068063 | 0.03448276 |
| 484 | 1 | 0.28680982 | 0.23136247 | 0.8077551 | 0.28868778 | 0.10550459 | 0.37827121 | 0.15183246 | 0.06896552 |
| 485 | 1 | 0.25153374 | 0.25064267 | 0.67326531 | 0.30859729 | 0.18348624 | 0.42188739 | 0.03403141 | 0 |
| 486 | 1 | 0.48006135 | 0.30462725 | 0.51785714 | 0.40995475 | 0.24770642 | 0.53053132 | 0.31151832 | 0.27320955 |
| 487 | 1 | 0.26993865 | 0.11696658 | 0.78346939 | 0.21266968 | 0.15137615 | 0.45519429 | 0.12041885 | 0.08753316 |
| 488 | 1 | 0.51533742 | 0.2377892 | 0.87336735 | 0.46515837 | 0.10091743 | 0.48929421 | 0.52356021 | 0.53050398 |
| 489 | 1 | 0.56441718 | 0.1966581 | 0.92030612 | 0.34660633 | 0.14220183 | 0.52260111 | 0.40052356 | 0.28381963 |
| 490 | 1 | 0.45705521 | 0.10025707 | 0.95 | 0.17013575 | 0.17889908 | 0.50911975 | 0.28795812 | 0.14588859 |
| 491 | 1 | 0.34662577 | 0.32390746 | 0.82265306 | 0.36561086 | 0.31192661 | 0.36954798 | 0.17015707 | 0.14323607 |
| 492 | 1 | 0.40184049 | 0.19280206 | 0.90561224 | 0.25067873 | 0.26605505 | 0.44726408 | 0.27225131 | 0.23607427 |
| 493 | 1 | 0.41257669 | 0.13239075 | 0.93040816 | 0.27420814 | 0.25688073 | 0.40444092 | 0.30628272 | 0.23342175 |
| 494 | 1 | 0.24079755 | 0.13496144 | 0.91112245 | 0.21809955 | 0.23853211 | 0.35685964 | 0.10994764 | 0.11140584 |
| 495 | 1 | 0.30828221 | 0.14910026 | 0.84561224 | 0.27873303 | 0.10091743 | 0.4036479 | 0.16492147 | 0.12997347 |

| 496 | 1 | 0.21932515 | 0.23650386 | 0.83806122 | 0.28144796 | 0.07798165 | 0.35051546 | 0.12303665 | 0.12732095 |
| --- | --- | --- | --- | --- | --- | --- | --- | --- | --- |
| 497 | 1 | 0.19785276 | 0.20179949 | 0.48061224 | 0.27149321 | 0.26146789 | 0.37351308 | 0.03664921 | 0 |
| 498 | 1 | 0.46472393 | 0.16195373 | 0.54173469 | 0.27420814 | 0.1146789 | 0.46708961 | 0.17539267 | 0.15649867 |
| 499 | 1 | 0.45858896 | 0.28663239 | 0.95 | 0.32850679 | 0.17431193 | 0.49167328 | 0.17801047 | 0.09018568 |
| 500 | 1 | 0.17944785 | 0.11439589 | 0.61071429 | 0.16561086 | 0.08715596 | 0.30848533 | 0.06544503 | 0.05835544 |
| 501 | 1 | 0.21779141 | 0.1092545 | 0.91520408 | 0.23076923 | 0.06422018 | 0.33782712 | 0.14921466 | 0.11405836 |
| 502 | 1 | 0.41871166 | 0.21079692 | 0.73591837 | 0.37556561 | 0.15137615 | 0.31165741 | 0.2565445 | 0.23076923 |
| 503 | 1 | 0.42484663 | 0.19023136 | 0.74244898 | 0.35927602 | 0.10550459 | 0.53766852 | 0.41099476 | 0.4403183 |
| 504 | 1 | 0.38343558 | 0.16452442 | 0.81520408 | 0.27782805 | 0.18807339 | 0.37747819 | 0.2408377 | 0.20424403 |
| 505 | 1 | 0.37423313 | 0.34061697 | 0.79642857 | 0.39276018 | 0.19266055 | 0.42030135 | 0.16753927 | 0.16976127 |
| 506 | 1 | 0.4493865 | 0.16195373 | 0.8455102 | 0.37013575 | 0.16972477 | 0.39095956 | 0.32984293 | 0.22281167 |
| 507 | 1 | 0.27760736 | 0.16195373 | 0.9905102 | 0.23891403 | 0.17431193 | 0.42268041 | 0.23036649 | 0.21750663 |
| 508 | 1 | 0.22239264 | 0.09768638 | 0.55693878 | 0.16561086 | 0.26146789 | 0.38540841 | 0.04973822 | 0.01591512 |
| 509 | 1 | 0.13190184 | 0.09511568 | 0.87704082 | 0.23438914 | 0.05504587 | 0.28390167 | 0.12827225 | 0.16445623 |
| 510 | 1 | 0.24386503 | 0.1748072 | 0.90530612 | 0.2280543 | 0.08715596 | 0.34496431 | 0.10994764 | 0.08753316 |
| 511 | 1 | 0.16411043 | 0.20565553 | 0.78 | 0.23257919 | 0.08715596 | 0.34575734 | 0.04973822 | 0.05039788 |
| 512 | 1 | 0.34662577 | 0.16966581 | 0.86581633 | 0.24343891 | 0.19266055 | 0.4258525 | 0.13612565 | 0.10079576 |
| 513 | 1 | 0.51840491 | 0.29691517 | 0.87255102 | 0.37104072 | 0.41284404 | 0.37351308 | 0.2539267 | 0.24403183 |
| 514 | 1 | 0.22699387 | 0.18123393 | 0.84897959 | 0.239819 | 0.17431193 | 0.36796193 | 0.13350785 | 0.06896552 |
| 515 | 1 | 0.35736196 | 0.0874036 | 0.93989796 | 0.24524887 | 0.1146789 | 0.49960349 | 0.33246073 | 0.33687003 |
| 516 | 1 | 0.33742331 | 0.29562982 | 0.68091837 | 0.33303167 | 0.12385321 | 0.38461538 | 0.12827225 | 0.07692308 |
| 517 | 1 | 0.32055215 | 0.16195373 | 0.7272449 | 0.239819 | 0 | 0.44250595 | 0.14659686 | 0.11140584 |
| 518 | 1 | 0.47239264 | 0.37403599 | 0.84346939 | 0.439819 | 0.11009174 | 0.52022205 | 0.34031414 | 0.34482759 |
| 519 | 1 | 0.49079755 | 0.24293059 | 0.6722449 | 0.42714932 | 0.16513761 | 0.43219667 | 0.47643979 | 0.38461538 |
| 520 | 1 | 0.38650307 | 0.39717224 | 0.77969388 | 0.44524887 | 0.1559633 | 0.50832672 | 0.18062827 | 0.15119363 |
| 521 | 1 | 0.82515337 | 0.37532134 | 0.97918367 | 0.45882353 | 0.49541284 | 0.68199841 | 0.80366492 | 0.32625995 |
| 522 | 1 | 0.44171779 | 0.18508997 | 0.94102041 | 0.35565611 | 0.14678899 | 0.4147502 | 0.46858639 | 0.44827586 |
| 523 | 1 | 0.37730061 | 0.14010283 | 0.83642857 | 0.23257919 | 0.05045872 | 0.41316416 | 0.29842932 | 0.27320955 |
| 524 | 1 | 0.42944785 | 0.21465296 | 0.93602041 | 0.34479638 | 0.16513761 | 0.4036479 | 0.29319372 | 0.23872679 |
| 525 | 1 | 0.32208589 | 0.10796915 | 0.90346939 | 0.26063348 | 0.16055046 | 0.33941316 | 0.2382199 | 0.25994695 |
| 526 | 1 | 0.22239264 | 0.17095116 | 0.53091837 | 0.27420814 | 0.05504587 | 0.33624108 | 0.2539267 | 0.32095491 |

| 527 | 1 | 0.20398773 | 0.37532134 | 0.92091837 | 0.40904977 | 0.08256881 | 0.27359239 | 0.13874346 | 0.11671088 |
| --- | --- | --- | --- | --- | --- | --- | --- | --- | --- |
| 528 | 1 | 0.37730061 | 0.27892031 | 0.63581633 | 0.47873303 | 0.34862385 | 0.43457573 | 0.17277487 | 0.12466844 |
| 529 | 1 | 0.23159509 | 0.16838046 | 0.68887755 | 0.21357466 | 0.1146789 | 0.26328311 | 0.10471204 | 0.1061008 |
| 530 | 1 | 0.25 | 0.2622108 | 0.74336735 | 0.40542986 | 0.18807339 | 0.40919905 | 0.14136126 | 0.21485411 |
| 531 | 1 | 0.2208589 | 0.42673522 | 0.62602041 | 0.46334842 | 0.11926606 | 0.36320381 | 0.10471204 | 0.07692308 |
| 532 | 1 | 0.14417178 | 0.16966581 | 0.74357143 | 0.23076923 | 0.17431193 | 0.26249009 | 0.04973822 | 0.02387268 |
| 533 | 1 | 0.56134969 | 0.22107969 | 0.74326531 | 0.46606335 | 0.05045872 | 0.57176844 | 0.71989529 | 0.72944297 |
| 534 | 1 | 0.36656442 | 0.19280206 | 0.8322449 | 0.30678733 | 0.18348624 | 0.44250595 | 0.28795812 | 0.23872679 |
| 535 | 1 | 0.23619632 | 0.13624679 | 0.49469388 | 0.29954751 | 0.05504587 | 0.42188739 | 0.27748691 | 0.31299735 |
| 536 | 1 | 0.42944785 | 0.23521851 | 0.785 | 0.36651584 | 0.21100917 | 0.4147502 | 0.33246073 | 0.31299735 |
| 537 | 1 | 0.27760736 | 0.19151671 | 0.97887755 | 0.30135747 | 0.1146789 | 0.37509913 | 0.12565445 | 0.07161804 |
| 538 | 1 | 0.32208589 | 0.18251928 | 0.74877551 | 0.30769231 | 0.1559633 | 0.38461538 | 0.21989529 | 0.17241379 |
| 539 | 1 | 0.54294479 | 0.19922879 | 0.92877551 | 0.35746606 | 0.37155963 | 0.62648692 | 0.36649215 | 0.33687003 |
| 540 | 1 | 0.22546012 | 0.26478149 | 0.75795918 | 0.26606335 | 0.13302752 | 0.27993656 | 0.02617801 | 0 |
| 541 | 1 | 0.39570552 | 0.33933162 | 0.84 | 0.37918552 | 0.29816514 | 0.43298969 | 0.08638743 | 0.04244032 |
| 542 | 1 | 0.53220859 | 0.18766067 | 0.91744898 | 0.2479638 | 0.16513761 | 0.49167328 | 0.42931937 | 0.43501326 |
| 543 | 1 | 0.38650307 | 0.2596401 | 0.88377551 | 0.41357466 | 0.12385321 | 0.41395718 | 0.27225131 | 0.24668435 |
| 544 | 1 | 0.40337423 | 0.31105398 | 0.69418367 | 0.3638009 | 0.14220183 | 0.44964314 | 0.17539267 | 0.10875332 |
| 545 | 1 | 0.31441718 | 0.20565553 | 0.65387755 | 0.27149321 | 0.09174312 | 0.39016653 | 0.2382199 | 0.17241379 |
| 546 | 1 | 0.41104294 | 0.28020566 | 0.89418367 | 0.35746606 | 0.16513761 | 0.37113402 | 0.29842932 | 0.31299735 |
| 547 | 1 | 0.54601227 | 0.18637532 | 0.93683673 | 0.22895928 | 0.3853211 | 0.58287074 | 0.26701571 | 0.17506631 |
| 548 | 1 | 0.33895706 | 0.3496144 | 0.58326531 | 0.34479638 | 0.18807339 | 0.41554322 | 0.21204188 | 0.17506631 |
| 549 | 1 | 0.19785276 | 0.15424165 | 0.795 | 0.26515837 | 0.1559633 | 0.4369548 | 0.04450262 | 0.04509284 |
| 550 | 1 | 0.31595092 | 0.18894602 | 0.93673469 | 0.25158371 | 0.23853211 | 0.37985726 | 0.20942408 | 0.1193634 |
| 551 | 1 | 0.1702454 | 0.18637532 | 0.60214286 | 0.21176471 | 0.05045872 | 0.37113402 | 0.05759162 | 0 |
| 552 | 1 | 0.37883436 | 0.21079692 | 0.77183673 | 0.37737557 | 0.1146789 | 0.35606661 | 0.33246073 | 0.41909814 |
| 553 | 1 | 0.42791411 | 0.29691517 | 0.84081633 | 0.31855204 | 0.33486239 | 0.36558287 | 0.03141361 | 0.03183024 |
| 554 | 1 | 0.55674847 | 0.1940874 | 0.9105102 | 0.31221719 | 0.29357798 | 0.52498017 | 0.32722513 | 0.33156499 |
| 555 | 1 | 0.16411043 | 0.37403599 | 0.765 | 0.40090498 | 0 | 0.32751784 | 0.08115183 | 0 |
| 556 | 1 | 0.45552147 | 0.23264781 | 0.85295918 | 0.26877828 | 0.18807339 | 0.518636 | 0.17015707 | 0.15119363 |
| 557 | 1 | 0.28527607 | 0.21722365 | 0.86357143 | 0.32760181 | 0.11009174 | 0.38858049 | 0.19109948 | 0.19098143 |

| 558 | 1 | 0.37730061 | 0.12210797 | 0.77969388 | 0.24886878 | 0.22018349 | 0.44171293 | 0.26701571 | 0.16445623 |
| --- | --- | --- | --- | --- | --- | --- | --- | --- | --- |
| 559 | 1 | 0.54754601 | 0.34447301 | 0.925 | 0.42895928 | 0.20642202 | 0.53132435 | 0.52617801 | 0.48806366 |
| 560 | 1 | 0.34202454 | 0.08097686 | 0.8377551 | 0.2081448 | 0.0733945 | 0.44250595 | 0.29842932 | 0.24403183 |
| 561 | 1 | 0.44171779 | 0.31362468 | 0.95816327 | 0.50316742 | 0.13302752 | 0.47581285 | 0.33246073 | 0.28381963 |
| 562 | 1 | 0.54447853 | 0.34318766 | 0.80826531 | 0.47239819 | 0.07798165 | 0.55511499 | 0.37696335 | 0.38196286 |
| 563 | 1 | 0.2208589 | 0.26992288 | 0.78285714 | 0.29773756 | 0.09633028 | 0.32910389 | 0.12827225 | 0.01061008 |
| 564 | 1 | 0.27300613 | 0.26863753 | 0.80081633 | 0.35022624 | 0.16513761 | 0.42188739 | 0.10994764 | 0.06366048 |
| 565 | 1 | 0.23006135 | 0.30077121 | 0.97785714 | 0.28778281 | 0.12844037 | 0.42981761 | 0.21989529 | 0.12466844 |
| 566 | 1 | 0.38190184 | 0.13496144 | 0.92387755 | 0.2561086 | 0.2293578 | 0.45281523 | 0.29057592 | 0.20689655 |
| 567 | 1 | 0.27147239 | 0.1529563 | 0.9005102 | 0.23257919 | 0.14678899 | 0.34337827 | 0.08376963 | 0.08488064 |
| 568 | 1 | 0.36503067 | 0.36760925 | 0.67408163 | 0.38823529 | 0.11009174 | 0.33386201 | 0.2486911 | 0.23076923 |
| 569 | 1 | 0.32515337 | 0.17609254 | 0.68377551 | 0.28325792 | 0.18348624 | 0.44409199 | 0.23036649 | 0.16445623 |
| 570 | 1 | 0.42638037 | 0.17866324 | 0.87265306 | 0.31945701 | 0.39449541 | 0.50118953 | 0.33507853 | 0.31299735 |
| 571 | 1 | 0.36809816 | 0.21079692 | 0.83540816 | 0.31855204 | 0.18807339 | 0.49405234 | 0.07853403 | 0.08753316 |
| 572 | 1 | 0.28527607 | 0.21722365 | 0.90816327 | 0.34660633 | 0.14678899 | 0.43536875 | 0.14659686 | 0.12732095 |
| 573 | 1 | 0.23619632 | 0.05269923 | 0.77295918 | 0.16651584 | 0.18807339 | 0.46233148 | 0.13350785 | 0.19098143 |
| 574 | 1 | 0.50153374 | 0.27377892 | 0.95683673 | 0.43348416 | 0.55963303 | 0.44647105 | 0.35863874 | 0.32360743 |
| 575 | 1 | 0.30214724 | 0.47043702 | 0.66204082 | 0.56923077 | 0.08715596 | 0.49088025 | 0.07329843 | 0.07427056 |
| 576 | 1 | 0.34815951 | 0.1966581 | 0.92785714 | 0.3040724 | 0.18348624 | 0.43219667 | 0.22774869 | 0.16180371 |
| 577 | 1 | 0.58435583 | 0.19023136 | 0.76785714 | 0.35384615 | 0.17431193 | 0.53528945 | 0.5104712 | 0.4801061 |
| 578 | 1 | 0.36503067 | 0.23007712 | 0.73030612 | 0.29683258 | 0.19266055 | 0.46074544 | 0.28534031 | 0.24668435 |
| 579 | 1 | 0.30828221 | 0.6529563 | 0.5055102 | 0.52760181 | 0.19266055 | 0.38620143 | 0.08376963 | 0 |
| 580 | 1 | 0.57208589 | 0.26863753 | 0.91989796 | 0.37828054 | 0.27522936 | 0.43774782 | 0.31151832 | 0.19893899 |
| 581 | 1 | 0.19325153 | 0.1748072 | 0.85428571 | 0.23167421 | 0.04587156 | 0.43536875 | 0.05497382 | 0.0265252 |
| 582 | 1 | 0.2392638 | 0.34190231 | 0.93183673 | 0.35746606 | 0.12844037 | 0.44409199 | 0.14397906 | 0.07427056 |
| 583 | 1 | 0.27453988 | 0.14010283 | 0.68520408 | 0.28778281 | 0.13761468 | 0.3148295 | 0.23298429 | 0.20954907 |
| 584 | 1 | 0.38496933 | 0.09383033 | 0.9905102 | 0.19819005 | 0.04587156 | 0.38937351 | 0.32722513 | 0.27586207 |
| 585 | 1 | 0.40184049 | 0.2840617 | 0.89163265 | 0.34570136 | 0.09174312 | 0.49484536 | 0.20942408 | 0.1061008 |
| 586 | 1 | 0.18865031 | 0.28020566 | 0.99295918 | 0.32126697 | 0.18807339 | 0.34655036 | 0.07068063 | 0.07161804 |
| 587 | 1 | 0.24846626 | 0.15167095 | 0.88397959 | 0.21447964 | 0.25229358 | 0.41950833 | 0.04188482 | 0.01061008 |
| 588 | 0 | 0.50153374 | 0.30077121 | 0.74836735 | 0.64886878 | 0 | 0.888977 | 0.77486911 | 0.74270557 |

| 589 | 0 | 0.32822086 | 0.13753213 | 0.84326531 | 0.23800905 | 0.10550459 | 0.47184774 | 0.23036649 | 0.20689655 |
| --- | --- | --- | --- | --- | --- | --- | --- | --- | --- |
| 590 | 0 | 0.3696319 | 0.14524422 | 0.82030612 | 0.21085973 | 0.13761468 | 0.33465504 | 0.37434555 | 0.22015915 |
| 591 | 0 | 0.75 | 0.29820051 | 0.95357143 | 0.47963801 | 0.45412844 | 0.64393339 | 0.52094241 | 0.41644562 |
| 592 | 0 | 0.14570552 | 0.18251928 | 0.71765306 | 0.2841629 | 0.02752294 | 0.32910389 | 0.13874346 | 0.11140584 |
| 593 | 0 | 0.53527607 | 0.18508997 | 0.58969388 | 0.239819 | 0.40366972 | 0.54956384 | 0.36649215 | 0.27055703 |
| 594 | 0 | 0.45245399 | 0.45758355 | 0.75979592 | 0.43167421 | 0.41743119 | 0.48057098 | 0.09947644 | 0.06100796 |
| 595 | 0 | 0.38957055 | 0.07455013 | 0.52387755 | 0.33484163 | 0.10091743 | 0.40919905 | 0.45549738 | 0.44297082 |
| 596 | 0 | 0.53220859 | 0.08868895 | 0.78887755 | 0.37737557 | 0 | 0.60586836 | 0.54450262 | 0.55172414 |
| 597 | 0 | 0.44325153 | 0.2159383 | 0.88887755 | 0.30045249 | 0.13761468 | 0.45440127 | 0.28010471 | 0.23872679 |
| 598 | 0 | 0.3404908 | 0.19537275 | 0.7672449 | 0.38280543 | 0.16513761 | 0.44329897 | 0.18324607 | 0.1193634 |
| 599 | 0 | 0.33128834 | 0.1748072 | 0.72653061 | 0.25520362 | 0.17431193 | 0.45598731 | 0.2617801 | 0.27055703 |
| 600 | 1 | 0.38343558 | 0.15424165 | 0.73112245 | 0.22352941 | 0.25688073 | 0.39888977 | 0.27486911 | 0.17241379 |
| 601 | 0 | 0.43711656 | 0.09897172 | 0.81153061 | 0.27058824 | 0.19266055 | 0.60666138 | 0.38219895 | 0.33156499 |
| 602 | 0 | 0.38190184 | 0.17352185 | 0.82346939 | 0.22533937 | 0.12844037 | 0.37827121 | 0.2434555 | 0.18832891 |
| 603 | 0 | 0.25613497 | 0.33290488 | 0.84510204 | 0.3719457 | 0.06880734 | 0.37351308 | 0.2382199 | 0.20159151 |
| 604 | 0 | 0.11656442 | 0.20694087 | 0.76520408 | 0.2841629 | 0.1559633 | 0.27597145 | 0.02094241 | 0.02122016 |
| 605 | 0 | 0.29601227 | 0.44858612 | 0.29010204 | 0.40633484 | 0.25688073 | 0.38065028 | 0.08115183 | 0.03183024 |
| 606 | 0 | 0.25613497 | 0.10796915 | 0.78081633 | 0.25882353 | 0.05504587 | 0.3592387 | 0.12565445 | 0.06366048 |
| 607 | 0 | 0.25306748 | 0.16709512 | 0.89704082 | 0.32036199 | 0.11009174 | 0.42109437 | 0.18586387 | 0.22015915 |
| 608 | 0 | 0.19478528 | 0.15424165 | 0.70326531 | 0.22986425 | 0.15137615 | 0.38223632 | 0.0104712 | 0 |
| 609 | 1 | 0.40337423 | 0.34575835 | 0.78 | 0.42262443 | 0.28899083 | 0.44488501 | 0.18586387 | 0.12732095 |
| 610 | 1 | 0.46932515 | 0.29048843 | 0.83102041 | 0.38552036 | 0.16055046 | 0.50118953 | 0.35340314 | 0.25198939 |
| 611 | 1 | 0.23159509 | 0.13367609 | 0.80306122 | 0.28144796 | 0.11926606 | 0.33544806 | 0.12827225 | 0.0928382 |
| 612 | 0 | 0.33588957 | 0.13881748 | 0.70142857 | 0.26515837 | 0.16972477 | 0.4409199 | 0.2539267 | 0.23872679 |
| 613 | 0 | 0.26840491 | 0.37017995 | 0.29173469 | 0.32488688 | 0.11009174 | 0.31562252 | 0.10471204 | 0.04774536 |
| 614 | 1 | 0.17484663 | 0.1066838 | 0.53061224 | 0.18461538 | 0.09174312 | 0.31007137 | 0.05497382 | 0.01591512 |
| 615 | 1 | 0.2791411 | 0.19023136 | 0.99581633 | 0.3040724 | 0.11926606 | 0.4520222 | 0.12827225 | 0.06366048 |
| 616 | 0 | 0.69478528 | 0.14267352 | 0.97663265 | 0.30588235 | 0.25688073 | 0.52022205 | 0.40575916 | 0.33687003 |
| 617 | 0 | 0.3190184 | 0.20051414 | 0.51459184 | 0.53846154 | 0.23853211 | 0.33941316 | 0.2408377 | 0.24403183 |
| 618 | 0 | 0.33435583 | 0.16195373 | 0.95306122 | 0.19728507 | 0.17889908 | 0.43219667 | 0.18324607 | 0.12732095 |
| 619 | 0 | 0.14110429 | 0.40745501 | 0.68469388 | 0.34208145 | 0.18807339 | 0.29976209 | 0.05235602 | 0.08222812 |

| 620 | 0 | 0.74233129 | 0.20694087 | 0.73010204 | 0.36470588 | 0 | 0.54321967 | 0.95026178 | 0.85676393 |
| --- | --- | --- | --- | --- | --- | --- | --- | --- | --- |
| 621 | 0 | 0.23006135 | 0.04884319 | 0.92377551 | 0.30588235 | 0 | 0.38699445 | 0.2617801 | 0.33156499 |
| 622 | 1 | 0.32822086 | 0.12724936 | 0.78193878 | 0.17737557 | 0.12385321 | 0.43933386 | 0.22774869 | 0.14588859 |
| 623 | 0 | 0.57208589 | 0.21336761 | 0.8472449 | 0.33755656 | 0.38073394 | 0.36161776 | 0.27225131 | 0.10875332 |
| 624 | 0 | 0.23312883 | 0.26092545 | 0.96285714 | 0.72669683 | 0.04587156 | 0.28231562 | 0.10732984 | 0.10875332 |
| 625 | 0 | 0.27607362 | 0.29820051 | 0.71326531 | 0.35113122 | 0.19724771 | 0.39095956 | 0.18586387 | 0.13793103 |
| 626 | 0 | 0.24079755 | 0.17352185 | 0.92877551 | 0.30497738 | 0 | 0.28548771 | 0.05759162 | 0.05835544 |
| 627 | 0 | 0.31595092 | 0.15809769 | 0.64938776 | 0.26063348 | 0.18807339 | 0.45678033 | 0.13350785 | 0.08222812 |
| 628 | 0 | 0.49539877 | 0.24678663 | 0.8405102 | 0.3638009 | 0.32110092 | 0.46391753 | 0.36649215 | 0.34748011 |
| 629 | 0 | 0.26533742 | 0.17737789 | 0.51397959 | 0.24977376 | 0.12844037 | 0.28865979 | 0.22251309 | 0.1193634 |
| 630 | 1 | 0.58282209 | 0.43187661 | 1 | 0.44977376 | 0.26605505 | 0.49881047 | 0 | 0 |
| 631 | 0 | 0.29447853 | 0.36503856 | 0.88377551 | 0.36108597 | 0.14220183 | 0.40206186 | 0.18062827 | 0.14323607 |
| 632 | 0 | 0.34202454 | 0.1966581 | 0.94153061 | 0.30316742 | 0.12844037 | 0.35368755 | 0.18324607 | 0.18567639 |
| 633 | 1 | 0.34509202 | 0.23136247 | 0.82765306 | 0.34570136 | 0.10091743 | 0.481364 | 0.23560209 | 0.23872679 |
| 634 | 0 | 0.30828221 | 0.20694087 | 0.90265306 | 0.29049774 | 0.18348624 | 0.39809675 | 0.10471204 | 0.1061008 |
| 635 | 0 | 0.42484663 | 0.27249357 | 0.92591837 | 0.43710407 | 0.16513761 | 0.46074544 | 0.40052356 | 0.37135279 |
| 636 | 0 | 0.16871166 | 0.14267352 | 0.4755102 | 0.24615385 | 0.08715596 | 0.21570182 | 0.11518325 | 0.09549072 |
| 637 | 0 | 0.15030675 | 0.10282776 | 0.05602041 | 0.11674208 | 0.08715596 | 0.27597145 | 0.07591623 | 0.05039788 |
| 638 | 0 | 0.3297546 | 0.18766067 | 0.76693878 | 0.39728507 | 0.2706422 | 0.4409199 | 0.10209424 | 0.0530504 |
| 639 | 0 | 0.48466258 | 0.14010283 | 0.88020408 | 0.38552036 | 0.13302752 | 0.33782712 | 0.4973822 | 0.48806366 |
| 640 | 0 | 0.27760736 | 0.21979434 | 0.67357143 | 0.40995475 | 0.09174312 | 0.48691515 | 0.13089005 | 0.0530504 |
| 641 | 0 | 0.12730061 | 0.04498715 | 0.49061224 | 0.23076923 | 0.05963303 | 0.30769231 | 0.08638743 | 0.04774536 |
| 642 | 0 | 0.39877301 | 0.05141388 | 0.89265306 | 0.25339367 | 0.09174312 | 0.36478985 | 0.20942408 | 0.21220159 |
| 643 | 0 | 0.3404908 | 0.23136247 | 0.8494898 | 0.38280543 | 0.06422018 | 0.38461538 | 0.23560209 | 0.18302387 |
| 644 | 1 | 0.33435583 | 0.28020566 | 0.88193878 | 0.34117647 | 0.16972477 | 0.45043616 | 0.04188482 | 0 |
| 645 | 0 | 0.24539877 | 0.18766067 | 0.86377551 | 0.2678733 | 0.11009174 | 0.32038065 | 0.15183246 | 0.10875332 |
| 646 | 0 | 0.1809816 | 0.12210797 | 0.70918367 | 0.22443439 | 0 | 0.35606661 | 0.09162304 | 0.0928382 |
| 647 | 0 | 0.8297546 | 0.30848329 | 0.69683673 | 0.27149321 | 0.27522936 | 0.57176844 | 0.07853403 | 0.0795756 |
| 648 | 1 | 0.25920245 | 0.24678663 | 0.85071429 | 0.20180995 | 0.21100917 | 0.35368755 | 0.02094241 | 0 |
| 649 | 0 | 0.35429448 | 0.14781491 | 0.50591837 | 0.23891403 | 0.0733945 | 0.60111023 | 0.21465969 | 0.39257294 |
| 650 | 0 | 0.61196319 | 0.23907455 | 0.70744898 | 0.43348416 | 0.24311927 | 0.4853291 | 0.55759162 | 0.63395225 |

| 651 | 0 | 0.50920245 | 0.15552699 | 0.91132653 | 0.28597285 | 0.33486239 | 0.43616178 | 0.29581152 | 0.34217507 |
| --- | --- | --- | --- | --- | --- | --- | --- | --- | --- |
| 652 | 1 | 0.22699387 | 0.14524422 | 0.68755102 | 0.23529412 | 0.0412844 | 0.31641554 | 0.18062827 | 0.23076923 |
| 653 | 0 | 0.42944785 | 0.16452442 | 0.70469388 | 0.16923077 | 0.37614679 | 0.370341 | 0.27486911 | 0.21750663 |
| 654 | 0 | 0.23006135 | 0 | 0.60081633 | 0.22624434 | 0 | 0.31720856 | 0.2617801 | 0.13262599 |
| 655 | 0 | 0.37730061 | 0.19151671 | 0.80612245 | 0.22986425 | 0.20183486 | 0.41712926 | 0.20680628 | 0.16180371 |
| 656 | 0 | 0.3404908 | 0.24807198 | 0.86969388 | 0.34932127 | 0.24311927 | 0.40840603 | 0.2460733 | 0.26259947 |
| 657 | 0 | 0.33282209 | 0.07583548 | 0.97693878 | 0.17828054 | 0.31651376 | 0.36796193 | 0.18062827 | 0.18302387 |
| 658 | 0 | 0.12576687 | 0.14652956 | 0.75928571 | 0.13303167 | 0 | 0.27200634 | 0.12827225 | 0.08753316 |
| 659 | 0 | 0.32822086 | 0.11953728 | 0.68938776 | 0.22895928 | 0.20642202 | 0.39492466 | 0.10994764 | 0.11140584 |
| 660 | 0 | 0.40644172 | 0.37789203 | 0.42163265 | 0.3321267 | 0.16972477 | 0.38461538 | 0.05759162 | 0.01856764 |
| 661 | 0 | 0.38343558 | 0.11182519 | 0.83806122 | 0.26063348 | 0.28899083 | 0.43774782 | 0.23560209 | 0.25729443 |
| 662 | 0 | 0.51687117 | 0.07197943 | 0.83510204 | 0.45791855 | 0.64220183 | 0.66851705 | 0.36649215 | 0.29708223 |
| 663 | 0 | 0.33895706 | 0.30462725 | 0.74704082 | 0.33484163 | 0.18807339 | 0.39571768 | 0.28272251 | 0.10875332 |
| 664 | 0 | 0.37269939 | 0.18637532 | 0.77397959 | 0.20452489 | 0.07798165 | 0.43298969 | 0.14397906 | 0.01061008 |
| 665 | 0 | 0.10582822 | 0.12724936 | 0.87 | 0.17013575 | 0.09174312 | 0.49484536 | 0.05235602 | 0.0530504 |
| 666 | 0 | 0.26380368 | 0.12210797 | 0.86959184 | 0.30769231 | 0.12844037 | 0.43933386 | 0.09162304 | 0.06100796 |
| 667 | 0 | 0.42944785 | 0.13367609 | 0.73061224 | 0.37828054 | 0.2706422 | 0.46471055 | 0.28795812 | 0.15119363 |
| 668 | 1 | 0.47546012 | 0.34318766 | 0.97418367 | 0.36742081 | 0.49082569 | 0.47501983 | 0.02879581 | 0 |
| 669 | 0 | 0.21625767 | 0.04884319 | 0.72979592 | 0.25791855 | 0.21100917 | 0.41316416 | 0.08900524 | 0.07161804 |
| 670 | 0 | 0.50766871 | 0.16323907 | 0.89173469 | 0.34479638 | 0.17431193 | 0.4520222 | 0.33246073 | 0.31034483 |
| 671 | 0 | 0.52607362 | 0.14524422 | 0.76857143 | 0.43076923 | 0.19724771 | 0.47105472 | 0.51570681 | 0.47214854 |
| 672 | 0 | 0.42944785 | 0.20179949 | 0.78469388 | 0.30045249 | 0.23853211 | 0.53370341 | 0.22774869 | 0.11671088 |
| 673 | 0 | 0.40184049 | 0.33676093 | 0.7827551 | 0.38552036 | 0.44954128 | 0.54639175 | 0 | 0 |
| 674 | 1 | 0.34815951 | 0.07326478 | 0.94969388 | 0.20542986 | 0.17431193 | 0.39730373 | 0.2486911 | 0.15119363 |
| 675 | 0 | 0.15797546 | 0.17095116 | 0.60520408 | 0.21357466 | 0.06880734 | 0.35051546 | 0.07591623 | 0.07692308 |
| 676 | 0 | 0.28527607 | 0.51156812 | 0.79061224 | 0.48054299 | 0.24311927 | 0.42109437 | 0.20942408 | 0 |
| 677 | 0 | 0.45705521 | 0.22622108 | 0.52785714 | 0.35565611 | 0.24770642 | 0.49405234 | 0.28272251 | 0.18037135 |
| 678 | 1 | 0.25766871 | 0.10539846 | 0.82010204 | 0.30859729 | 0.11926606 | 0.31165741 | 0.12565445 | 0.11405836 |
| 679 | 1 | 0.14570552 | 0.27892031 | 0.14928571 | 0.25429864 | 0.05504587 | 0.35448057 | 0.06020942 | 0.01856764 |
| 680 | 0 | 0.41871166 | 0.15424165 | 0.73744898 | 0.34208145 | 0.17431193 | 0.46708961 | 0.32984293 | 0.30238727 |
| 681 | 0 | 0.46165644 | 0.23521851 | 0.72826531 | 0.37104072 | 0.13761468 | 0.46153846 | 0.27225131 | 0.20159151 |

| 682 | 0 | 0.51533742 | 0.29562982 | 0.79 | 0.46153846 | 0.09174312 | 0.53846154 | 0.42408377 | 0.32095491 |
| --- | --- | --- | --- | --- | --- | --- | --- | --- | --- |
| 683 | 0 | 0.18404908 | 0.19280206 | 0.8872449 | 0.29864253 | 0 | 0.44012688 | 0.11780105 | 0.0397878 |
| 684 | 0 | 0.28527607 | 0.31619537 | 0.65806122 | 0.2841629 | 0.1146789 | 0.53766852 | 0.13350785 | 0.066313 |
| 685 | 0 | 0.1809816 | 0.13624679 | 0.82591837 | 0.21357466 | 0.32568807 | 0.34655036 | 0.03141361 | 0.06366048 |
| 686 | 1 | 0.42177914 | 0.2596401 | 0.91836735 | 0.32669683 | 0.26605505 | 0.38937351 | 0.11256545 | 0.07692308 |
| 687 | 0 | 0.2791411 | 0.11696658 | 0.32265306 | 0.32941176 | 0.05045872 | 0.38778747 | 0.26701571 | 0.1193634 |
| 688 | 0 | 0.37269939 | 0.25192802 | 0.98071429 | 0.48144796 | 0.3853211 | 0.4369548 | 0.19633508 | 0.17241379 |
| 689 | 0 | 0.6196319 | 0.15552699 | 0.95571429 | 0.28054299 | 0.49541284 | 0.6407613 | 0.52879581 | 0.49867374 |
| 690 | 0 | 0.31134969 | 0.07069409 | 0.765 | 0.2 | 0.16972477 | 0.45281523 | 0.33769634 | 0.29177719 |
| 691 | 0 | 0.54754601 | 0.11825193 | 0.90795918 | 0.43800905 | 0.26605505 | 0.63045202 | 0.45287958 | 0.4270557 |
| 692 | 1 | 0.29141104 | 0.24421594 | 0.60112245 | 0.3158371 | 0.0733945 | 0.23869944 | 0.08376963 | 0.08488064 |
| 693 | 0 | 0.36809816 | 0.17352185 | 0.86561224 | 0.43438914 | 0 | 0.41633624 | 0.47120419 | 0.55702918 |
| 694 | 0 | 0.13650307 | 0.34447301 | 0.73612245 | 0.32307692 | 0 | 0.28310864 | 0 | 0 |
| 695 | 0 | 0.43251534 | 0.20565553 | 0.97459184 | 0.30769231 | 0.23394495 | 0.39095956 | 0.28534031 | 0.32360743 |
| 696 | 0 | 0.54294479 | 0.51928021 | 0.86255102 | 0.51040724 | 0.23394495 | 0.40047581 | 0.26439791 | 0.20159151 |
| 697 | 0 | 0.45398773 | 0.15552699 | 0.94306122 | 0.37828054 | 0.30733945 | 0.41712926 | 0.4947644 | 0.53580902 |
| 698 | 0 | 0.30214724 | 0.07840617 | 0.89204082 | 0.16470588 | 0 | 0.33624108 | 0.27748691 | 0.32095491 |
| 699 | 0 | 0.69785276 | 0.17352185 | 0.84387755 | 0.39547511 | 0.34862385 | 0.61459159 | 0.7513089 | 0.78249337 |
| 700 | 0 | 0.26380368 | 0.20951157 | 0.9422449 | 0.30316742 | 0.13302752 | 0.31086439 | 0.02617801 | 0.0265252 |
| 701 | 0 | 0.28220859 | 0.16966581 | 0.90428571 | 0.25791855 | 0.20642202 | 0.36161776 | 0.09685864 | 0.0530504 |
| 702 | 1 | 0.26840491 | 0.15552699 | 0.92061224 | 0.40180995 | 0.15137615 | 0.36399683 | 0.16230366 | 0.16445623 |
| 703 | 0 | 0.24693252 | 0.19922879 | 0.91571429 | 0.28506787 | 0.19266055 | 0.4853291 | 0.15445026 | 0.09549072 |
| 704 | 0 | 0.36809816 | 0.29434447 | 0.85520408 | 0.42714932 | 0.1559633 | 0.46550357 | 0.19109948 | 0.15649867 |
| 705 | 0 | 0.26380368 | 0.06298201 | 0.97826531 | 0.13303167 | 0.22477064 | 0.48691515 | 0.19371728 | 0.12997347 |
| 706 | 0 | 0.41717791 | 0.14010283 | 0.67357143 | 0.3438914 | 0.12385321 | 0.62490087 | 0.35602094 | 0.21750663 |
| 707 | 0 | 0.07361963 | 0.1529563 | 0.92469388 | 0.21538462 | 0 | 0.22680412 | 0.06282723 | 0.06366048 |
| 708 | 0 | 0.52607362 | 0.31748072 | 0.46408163 | 0.5239819 | 0.29357798 | 0.53528945 | 0.33769634 | 0.39787798 |
| 709 | 0 | 0.31288344 | 0.30976864 | 0.65897959 | 0.31764706 | 0.16055046 | 0.4147502 | 0.15183246 | 0.10079576 |
| 710 | 1 | 0.54294479 | 0.47557841 | 0.88520408 | 0.39004525 | 0.14220183 | 0.54956384 | 0.26963351 | 0.16445623 |
| 711 | 0 | 0.61503067 | 0.06940874 | 0.25959184 | 0.11764706 | 0.36238532 | 0.80888184 | 0.17277487 | 0.10875332 |
| 712 | 0 | 0.2898773 | 0.18251928 | 0.89306122 | 0.24615385 | 0.17431193 | 0.34655036 | 0.10732984 | 0.04774536 |

| 713 | 0 | 0.37883436 | 0.18123393 | 0.83040816 | 0.33393665 | 0.21100917 | 0.36637589 | 0.2486911 | 0.16976127 |
| --- | --- | --- | --- | --- | --- | --- | --- | --- | --- |
| 714 | 0 | 0.28220859 | 0.3251928 | 0.58142857 | 0.38190045 | 0.10550459 | 0.481364 | 0.07329843 | 0.01061008 |
| 715 | 0 | 0.21165644 | 0.26092545 | 0.73459184 | 0.27873303 | 0.18807339 | 0.31562252 | 0.08376963 | 0.06366048 |
| 716 | 0 | 0.29601227 | 0.15424165 | 0.79244898 | 0.22171946 | 0.05504587 | 0.42347343 | 0.17539267 | 0.14588859 |
| 717 | 1 | 0.52300613 | 0.08226221 | 0.68173469 | 0.35294118 | 0.03211009 | 0.38302934 | 0.5026178 | 0.43236074 |
| 718 | 0 | 0.34815951 | 0.16838046 | 0.72785714 | 0.31312217 | 0.20183486 | 0.4147502 | 0.11518325 | 0.06100796 |
| 719 | 0 | 0.47392638 | 0.11053985 | 0.83479592 | 0.38190045 | 0.12844037 | 0.41316416 | 0.5104712 | 0.50928382 |
| 720 | 0 | 0.29907975 | 0.12596401 | 0.44867347 | 0.20180995 | 0.11926606 | 0.35606661 | 0.19109948 | 0.15915119 |
| 721 | 0 | 0.44631902 | 0.34061697 | 0.87244898 | 0.44253394 | 0.25688073 | 0.48215702 | 0.23298429 | 0.20159151 |
| 722 | 0 | 0.4309816 | 0.31876607 | 0.87877551 | 0.39638009 | 0.2293578 | 0.51149881 | 0.19371728 | 0.08753316 |
| 723 | 0 | 0.11042945 | 0.04627249 | 0.64244898 | 0.17375566 | 0.05504587 | 0.42823156 | 0.03141361 | 0.03183024 |
| 724 | 1 | 0.23773006 | 0.1503856 | 0.93785714 | 0.31674208 | 0.13302752 | 0.33465504 | 0.07591623 | 0.03448276 |
| 725 | 0 | 0.21625767 | 0.16323907 | 0.88622449 | 0.35837104 | 0.12844037 | 0.4036479 | 0.18586387 | 0.11140584 |
| 726 | 1 | 0.28067485 | 0.33933162 | 0.90489796 | 0.50678733 | 0.09174312 | 0.41157811 | 0.21204188 | 0.13527851 |
| 727 | 0 | 0.1993865 | 0.08354756 | 0.85469388 | 0.08868778 | 0.29816514 | 0.38699445 | 0 | 0 |
| 728 | 1 | 0.36503067 | 0.3059126 | 0.93418367 | 0.38461538 | 0.41284404 | 0.38302934 | 0.13350785 | 0.06896552 |
| 729 | 0 | 0.21932515 | 0.23007712 | 0.76469388 | 0.22624434 | 0.08256881 | 0.4258525 | 0.04712042 | 0.04774536 |
| 730 | 0 | 0.2101227 | 0.17609254 | 0.42479592 | 0.51674208 | 0 | 0.38065028 | 0.06020942 | 0.02917772 |
| 731 | 0 | 0.14110429 | 0.32005141 | 0.67836735 | 0.33665158 | 0.10091743 | 0.30214116 | 0.0104712 | 0 |
| 732 | 0 | 0.17791411 | 0.13753213 | 0.87469388 | 0.17013575 | 0.12385321 | 0.44567803 | 0.14136126 | 0.09549072 |
| 733 | 0 | 0.34202454 | 0.05141388 | 0.88653061 | 0.18914027 | 0.24311927 | 0.50594766 | 0.22513089 | 0.23872679 |
| 734 | 0 | 0.18404908 | 0.33804627 | 0.6105102 | 0.29683258 | 0.25229358 | 0.37272006 | 0.11518325 | 0 |
| 735 | 0 | 0.56748466 | 0.16195373 | 0.86816327 | 0.41900452 | 0.2293578 | 0.48691515 | 0.61518325 | 0.66843501 |
| 736 | 0 | 0.34202454 | 0.34318766 | 0.61510204 | 0.42352941 | 0.30733945 | 0.44171293 | 0.17539267 | 0.17771883 |
| 737 | 0 | 0.28374233 | 0.11953728 | 0.94632653 | 0.25158371 | 0.05504587 | 0.38540841 | 0.12041885 | 0.06100796 |
| 738 | 0 | 0.27453988 | 0.12982005 | 0.85030612 | 0.20452489 | 0.25229358 | 0.28469469 | 0.14397906 | 0.06100796 |
| 739 | 0 | 0.31441718 | 0.10796915 | 0.55816327 | 0.35384615 | 0 | 0.43536875 | 0.14659686 | 0.12466844 |
| 740 | 0 | 0.24539877 | 0.18380463 | 0.66673469 | 0.19366516 | 0.24311927 | 0.52339413 | 0.09424084 | 0.14058355 |
| 741 | 0 | 0.16104294 | 0.15424165 | 0.51530612 | 0.21357466 | 0.09174312 | 0.51704996 | 0.03926702 | 0 |
| 742 | 0 | 0.43251534 | 0.33161954 | 0.86755102 | 0.33303167 | 0.27981651 | 0.61300555 | 0.28795812 | 0.22811671 |
| 743 | 0 | 0.38650307 | 0.38946015 | 0.65030612 | 0.46244344 | 0.2293578 | 0.44012688 | 0.2486911 | 0.23342175 |

| 744 | 0 | 0.34969325 | 0.26092545 | 0.88316327 | 0.59728507 | 0 | 0.38223632 | 0.33246073 | 0.20159151 |
| --- | --- | --- | --- | --- | --- | --- | --- | --- | --- |
| 745 | 0 | 0.39723926 | 0.12082262 | 0.52806122 | 0.38371041 | 0 | 0.63441713 | 0.43193717 | 0.31299735 |
| 746 | 0 | 0.2607362 | 0.26349614 | 0.84469388 | 0.34570136 | 0.16055046 | 0.32038065 | 0.14921466 | 0.0928382 |
| 747 | 0 | 0.27300613 | 0.16838046 | 0.94653061 | 0.26244344 | 0.15137615 | 0.40444092 | 0.17015707 | 0.14854111 |
| 748 | 0 | 0.25766871 | 0.11311054 | 0.58040816 | 0.31221719 | 0.0412844 | 0.3925456 | 0.09162304 | 0.0928382 |
| 749 | 0 | 0.25766871 | 0.17352185 | 0.85265306 | 0.36561086 | 0.07798165 | 0.36082474 | 0.13350785 | 0.13527851 |
| 750 | 0 | 0.4601227 | 0.23521851 | 0.84653061 | 0.33122172 | 0.2293578 | 0.67327518 | 0.39267016 | 0.17771883 |
| 751 | 0 | 0.21319018 | 0.20437018 | 0.57510204 | 0.32488688 | 0.09174312 | 0.34734338 | 0.15706806 | 0.1061008 |
| 752 | 0 | 0.27147239 | 0.09125964 | 0.8094898 | 0.24615385 | 0.11009174 | 0.29976209 | 0.27748691 | 0.22015915 |
| 753 | 0 | 0.41411043 | 0.24935733 | 0.93204082 | 0.27511312 | 0.21100917 | 0.52260111 | 0.2434555 | 0.18037135 |
| 754 | 0 | 0.38496933 | 0.14652956 | 0.53132653 | 0.30950226 | 0 | 0.48850119 | 0.41884817 | 0.48541114 |
| 755 | 0 | 0.24233129 | 0.44858612 | 0.90306122 | 0.43710407 | 0.25229358 | 0.36478985 | 0.08376963 | 0.12732095 |
| 756 | 0 | 0.31288344 | 0.19151671 | 0.92173469 | 0.22081448 | 0.31192661 | 0.33386201 | 0.17801047 | 0 |
| 757 | 1 | 0.31595092 | 0.46401028 | 0.8172449 | 0.44343891 | 0.11926606 | 0.32672482 | 0 | 0.06896552 |
| 758 | 0 | 0.41257669 | 0.19794344 | 0.91397959 | 0.32036199 | 0.17431193 | 0.41316416 | 0.30104712 | 0.28381963 |
| 759 | 0 | 0.28220859 | 0.20565553 | 0.81795918 | 0.22533937 | 0.11009174 | 0.37509913 | 0.18848168 | 0.12732095 |
| 760 | 0 | 0.35736196 | 0.09511568 | 0.90765306 | 0.29954751 | 0.05504587 | 0.55432197 | 0.35340314 | 0.29177719 |
| 761 | 0 | 0.17944785 | 0.10025707 | 0.23642857 | 0.24705882 | 0 | 0.18556701 | 0.20418848 | 0.10344828 |
| 762 | 0 | 0.63190184 | 0.21208226 | 0.79959184 | 0.33031674 | 0.43119266 | 0.68199841 | 0.36910995 | 0.28116711 |
| 763 | 0 | 0.41871166 | 0.06169666 | 0.6094898 | 0.14570136 | 0.22018349 | 0.50911975 | 0.20942408 | 0.16976127 |
| 764 | 0 | 0.64263804 | 0.25064267 | 0.96346939 | 0.29773756 | 0.96330275 | 0.43933386 | 0.11780105 | 0.0795756 |
| 765 | 0 | 0.15337423 | 0.04241645 | 0.76163265 | 0.15113122 | 0.15137615 | 0.29103886 | 0 | 0 |
| 766 | 0 | 0.75153374 | 0.40231362 | 0.82826531 | 0.50497738 | 0.06422018 | 0.51784298 | 0.28534031 | 0.03713528 |
| 767 | 0 | 0.36503067 | 0.21336761 | 0.83142857 | 0.2361991 | 0 | 0.35765266 | 0.12565445 | 0.25198939 |
| 768 | 0 | 0.30521472 | 0.3251928 | 0.74602041 | 0.39457014 | 0.0733945 | 0.42743854 | 0.19633508 | 0.10079576 |
| 769 | 0 | 0.26840491 | 0.1529563 | 0.77397959 | 0.26063348 | 0.25688073 | 0.33941316 | 0.12827225 | 0.11140584 |
| 770 | 0 | 0.13496933 | 0.11311054 | 0.8244898 | 0.22171946 | 0 | 0.29183188 | 0.04712042 | 0.04774536 |
| 771 | 0 | 0.20858896 | 0.24935733 | 0.79081633 | 0.3918552 | 0.05045872 | 0.3259318 | 0.11518325 | 0.07692308 |
| 772 | 0 | 0.40337423 | 0.14010283 | 0.43234694 | 0.36561086 | 0.11926606 | 0.41157811 | 0.41884817 | 0.28912467 |
| 773 | 0 | 0.13957055 | 0.0655527 | 0.9544898 | 0.21628959 | 0.16513761 | 0.2926249 | 0.03926702 | 0.02387268 |
| 774 | 0 | 0.30981595 | 0.18766067 | 0.91316327 | 0.30769231 | 0.18348624 | 0.39571768 | 0.14921466 | 0.12732095 |

| 775 | 0 | 0.41411043 | 0.21336761 | 0.71469388 | 0.18823529 | 0.09633028 | 0.42902458 | 0.21727749 | 0.16445623 |
| --- | --- | --- | --- | --- | --- | --- | --- | --- | --- |
| 776 | 1 | 0.36809816 | 0.54241645 | 0.28020408 | 0.43800905 | 0.1146789 | 0.29341792 | 0.34554974 | 0.0397878 |
| 777 | 0 | 0.35889571 | 0.40102828 | 0.94714286 | 0.52488688 | 0.10091743 | 0.40681998 | 0.20418848 | 0.1193634 |
| 778 | 0 | 0.32361963 | 0.27120823 | 0.9444898 | 0.39728507 | 0.21559633 | 0.42347343 | 0.12303665 | 0.05835544 |
| 779 | 0 | 0.22546012 | 0.17095116 | 0.8994898 | 0.28506787 | 0.32110092 | 0.42188739 | 0.05497382 | 0 |
| 780 | 0 | 0.3190184 | 0.10796915 | 0.93938776 | 0.29502262 | 0.08256881 | 0.45043616 | 0.27225131 | 0.28912467 |
| 781 | 0 | 0.09969325 | 0.29177378 | 0.4877551 | 0.38099548 | 0.10091743 | 0.25693894 | 0.05759162 | 0 |
| 782 | 1 | 0.25306748 | 0.04884319 | 0.6344898 | 0.19457014 | 0.2293578 | 0.37192704 | 0.10994764 | 0.0397878 |
| 783 | 1 | 0.41717791 | 0.30976864 | 0.825 | 0.41357466 | 0.19266055 | 0.40681998 | 0.08115183 | 0.04509284 |
| 784 | 0 | 0.3190184 | 0.0848329 | 0.92071429 | 0.17828054 | 0.15137615 | 0.38223632 | 0.17277487 | 0.05835544 |
| 785 | 1 | 0.4892638 | 0.23393316 | 0.8455102 | 0.3719457 | 0.28899083 | 0.5146709 | 0.21204188 | 0.15649867 |
| 786 | 1 | 0.18404908 | 0.24164524 | 0.15693878 | 0.20904977 | 0.0412844 | 0.20301348 | 0.08900524 | 0 |
| 787 | 0 | 0.37730061 | 0.13239075 | 0.70908163 | 0.42081448 | 0.20642202 | 0.40444092 | 0.34031414 | 0.29708223 |
| 788 | 1 | 0.35889571 | 0.2377892 | 0.74367347 | 0.31131222 | 0.27981651 | 0.45678033 | 0.14659686 | 0.1193634 |
| 789 | 0 | 0.3696319 | 0.1940874 | 0.52244898 | 0.30497738 | 0.21559633 | 0.48057098 | 0.15183246 | 0.0530504 |
| 790 | 0 | 0.26380368 | 0.04755784 | 0.88397959 | 0.08325792 | 0.22477064 | 0.34972244 | 0.06544503 | 0.04774536 |
| 791 | 0 | 0.36196319 | 0.24678663 | 0.69438776 | 0.35384615 | 0.15137615 | 0.4147502 | 0.15445026 | 0.12466844 |
| 792 | 0 | 0.0506135 | 0 | 0.28826531 | 0.06063348 | 0 | 0.63679619 | 0.08638743 | 0.17771883 |
| 793 | 0 | 0.26993865 | 0.16580977 | 0.96469388 | 0.41176471 | 0.21559633 | 0.28707375 | 0.18848168 | 0.16445623 |
| 794 | 0 | 0.34969325 | 0.11696658 | 0.86765306 | 0.33393665 | 0.18807339 | 0.41157811 | 0.2617801 | 0.24137931 |
| 795 | 0 | 0.18558282 | 0.2377892 | 0.65979592 | 0.30316742 | 0.09633028 | 0.41237113 | 0.07329843 | 0.03713528 |
| 796 | 0 | 0.24539877 | 0.22236504 | 0.89632653 | 0.35927602 | 0.13761468 | 0.32989691 | 0.10471204 | 0.07161804 |
| 797 | 0 | 0.4493865 | 0.32904884 | 0.9177551 | 0.43076923 | 0.16972477 | 0.41554322 | 0 | 0 |
| 798 | 0 | 0.38803681 | 0.16195373 | 0.79306122 | 0.21357466 | 0.19266055 | 0.48770817 | 0.19895288 | 0.20159151 |
| 799 | 1 | 0.38803681 | 0.34447301 | 0.58908163 | 0.42352941 | 0.27522936 | 0.39413164 | 0.10994764 | 0.0132626 |
| 800 | 0 | 0.20552147 | 0.08097686 | 0.58469388 | 0.23076923 | 0.1146789 | 0.41237113 | 0.08900524 | 0.07427056 |
| 801 | 0 | 0.31134969 | 0.27377892 | 0.45683673 | 0.37556561 | 0.27981651 | 0.35368755 | 0.13350785 | 0.10875332 |
| 802 | 0 | 0.38957055 | 0.30719794 | 0.56377551 | 0.33484163 | 0.16513761 | 0.38937351 | 0.09947644 | 0.05039788 |
| 803 | 0 | 0.26226994 | 0.11696658 | 0.92520408 | 0.15475113 | 0.2293578 | 0.44726408 | 0.10471204 | 0.1061008 |
| 804 | 1 | 0.25306748 | 0.1966581 | 0.83346939 | 0.30678733 | 0.10550459 | 0.39413164 | 0.11518325 | 0.05570292 |
| 805 | 1 | 0.21319018 | 0.15167095 | 0.52030612 | 0.22895928 | 0.08256881 | 0.31720856 | 0.11256545 | 0.066313 |

| 806 | 0 | 0.6595092 | 0.20308483 | 0.87183673 | 0.38914027 | 0 | 0.5741475 | 0.47382199 | 0.29973475 |
| --- | --- | --- | --- | --- | --- | --- | --- | --- | --- |
| 807 | 0 | 0.42484663 | 0.1503856 | 0.51755102 | 0.22533937 | 0.15137615 | 0.47660587 | 0.37958115 | 0.27320955 |
| 808 | 0 | 0.2898773 | 0.18508997 | 0.65755102 | 0.36651584 | 0.12385321 | 0.35685964 | 0.11780105 | 0.1193634 |
| 809 | 0 | 0.25766871 | 0.02570694 | 0.65846939 | 0.21447964 | 0.17889908 | 0.50118953 | 0.12827225 | 0.0795756 |
| 810 | 0 | 0.50306748 | 0.16838046 | 0.64265306 | 0.29683258 | 0.30275229 | 0.45043616 | 0.34293194 | 0.28912467 |
| 811 | 0 | 0.32668712 | 0.43701799 | 0.86602041 | 0.40633484 | 0.32568807 | 0.33306899 | 0.14921466 | 0.03713528 |
| 812 | 0 | 0.28374233 | 0.15809769 | 0.67887755 | 0.25067873 | 0.14220183 | 0.42743854 | 0.12041885 | 0.08222812 |
| 813 | 0 | 0.1303681 | 0.23007712 | 0.79306122 | 0.3158371 | 0.0412844 | 0.28469469 | 0.09947644 | 0.05039788 |
| 814 | 0 | 0.42177914 | 0.11568123 | 0.5672449 | 0.35294118 | 0.32110092 | 0.481364 | 0.28534031 | 0.13527851 |
| 815 | 0 | 0.25153374 | 0.08997429 | 0.69928571 | 0.23257919 | 0 | 0.5741475 | 0.18324607 | 0.12466844 |
| 816 | 0 | 0.2392638 | 0.25192802 | 0.95826531 | 0.33393665 | 0.03669725 | 0.3592387 | 0.10471204 | 0.07427056 |
| 817 | 0 | 0.36656442 | 0.2377892 | 0.62173469 | 0.39457014 | 0.13761468 | 0.43616178 | 0.09424084 | 0.0795756 |
| 818 | 1 | 0.30674847 | 0.40745501 | 0.8077551 | 0.38280543 | 0.21559633 | 0.31720856 | 0.15445026 | 0.0928382 |
| 819 | 0 | 0.61503067 | 0.40102828 | 0.80591837 | 0.38733032 | 0.12385321 | 0.52339413 | 0.16230366 | 0.14323607 |
| 820 | 0 | 0.36809816 | 0.14910026 | 0.80540816 | 0.28325792 | 0.16513761 | 0.50198255 | 0.11518325 | 0.11671088 |
| 821 | 0 | 0.23773006 | 0.19023136 | 0.84755102 | 0.25339367 | 0.10550459 | 0.41554322 | 0.15183246 | 0.12466844 |
| 822 | 1 | 0.23312883 | 0.17994859 | 0.64571429 | 0.25339367 | 0 | 0.42505948 | 0.06020942 | 0.03183024 |
| 823 | 0 | 0.49079755 | 0.26606684 | 0.64387755 | 0.34117647 | 0.25688073 | 0.61221253 | 0.19633508 | 0.10079576 |
| 824 | 0 | 0.36196319 | 0.28920308 | 0.83112245 | 0.27782805 | 0.43577982 | 0.40285488 | 0.12303665 | 0.03183024 |
| 825 | 0 | 0.41564417 | 0.20437018 | 0.88489796 | 0.35294118 | 0.25688073 | 0.41712926 | 0.39528796 | 0.4005305 |
| 826 | 0 | 0.32515337 | 0.22750643 | 0.44204082 | 0.28778281 | 0.16055046 | 0.58842189 | 0.18586387 | 0.28116711 |
| 827 | 1 | 0.36656442 | 0.27249357 | 0.75826531 | 0.33393665 | 0.20183486 | 0.43219667 | 0.27486911 | 0.22015915 |
| 828 | 0 | 0.41411043 | 0.06940874 | 0.76234694 | 0.11402715 | 0 | 0.49881047 | 0.23560209 | 0.09549072 |
| 829 | 0 | 0.33435583 | 0.18894602 | 0.59214286 | 0.27239819 | 0.11926606 | 0.33068993 | 0.31937173 | 0.20424403 |
| 830 | 0 | 0.4309816 | 0.18894602 | 0.86234694 | 0.26515837 | 0.33486239 | 0.4258525 | 0.15968586 | 0.12997347 |
| 831 | 0 | 0.35889571 | 0.28920308 | 0.84683673 | 0.41447964 | 0.16055046 | 0.35210151 | 0.31675393 | 0.28381963 |
| 832 | 0 | 0.32822086 | 0.20308483 | 0.77346939 | 0.34751131 | 0.1559633 | 0.35765266 | 0.26701571 | 0.23872679 |
| 833 | 0 | 0.33588957 | 0.09383033 | 0.54836735 | 0.28054299 | 0.08256881 | 0.50674068 | 0.28534031 | 0.28912467 |
| 834 | 0 | 0.31441718 | 0.24935733 | 0.82622449 | 0.30950226 | 0.21100917 | 0.28945282 | 0.17801047 | 0.09018568 |
| 835 | 0 | 0.23619632 | 0.15552699 | 0.50510204 | 0.31764706 | 0.25229358 | 0.37430611 | 0.17277487 | 0.14588859 |
| 836 | 0 | 0.30828221 | 0.1940874 | 0.73867347 | 0.2841629 | 0.17431193 | 0.32910389 | 0.19633508 | 0.066313 |

| 837 | 0 | 0.24386503 | 0.14138817 | 0.89571429 | 0.19909502 | 0.11009174 | 0.29976209 | 0.15968586 | 0.12997347 |
| --- | --- | --- | --- | --- | --- | --- | --- | --- | --- |
| 838 | 0 | 0.2208589 | 0.04627249 | 0.65489796 | 0.14660633 | 0.16513761 | 0.28469469 | 0.14136126 | 0.19098143 |
| 839 | 0 | 0.25460123 | 0.03856041 | 0.74857143 | 0.21900452 | 0 | 0.3592387 | 0.03926702 | 0 |
| 840 | 0 | 0.24846626 | 0.09768638 | 0.67714286 | 0.25429864 | 0.10091743 | 0.25773196 | 0.02879581 | 0.02917772 |
| 841 | 0 | 0.46319018 | 0.11953728 | 0.52346939 | 0.33574661 | 0 | 0.5519429 | 0.2434555 | 0.12201592 |
| 842 | 0 | 0.38190184 | 0.20051414 | 0.90734694 | 0.34027149 | 0.26146789 | 0.40206186 | 0.15445026 | 0.18567639 |
| 843 | 0 | 0.36349693 | 0.22236504 | 0.835 | 0.37104072 | 0.37614679 | 0.48374306 | 0.12041885 | 0.09549072 |
| 844 | 0 | 0.25460123 | 0.14652956 | 0.76204082 | 0.20542986 | 0.11926606 | 0.33306899 | 0.04450262 | 0.04509284 |
| 845 | 0 | 0.28680982 | 0.39717224 | 0.87163265 | 0.44343891 | 0.14678899 | 0.45995242 | 0.18586387 | 0.1193634 |
| 846 | 0 | 0.26380368 | 0.13239075 | 0.67530612 | 0.38823529 | 0.47247706 | 0.35368755 | 0.13350785 | 0.18302387 |
| 847 | 0 | 0.19171779 | 0.12082262 | 0.90306122 | 0.19819005 | 0.14220183 | 0.31086439 | 0.08115183 | 0.04244032 |
| 848 | 0 | 0.13957055 | 0.11696658 | 0.74377551 | 0.25882353 | 0 | 0.26804124 | 0.03403141 | 0.06896552 |
| 849 | 0 | 0.3297546 | 0.1311054 | 0.8844898 | 0.30497738 | 0.15137615 | 0.43854084 | 0.18062827 | 0.15119363 |
| 850 | 1 | 0.45705521 | 0.3033419 | 0.7872449 | 0.46968326 | 0.14678899 | 0.49088025 | 0.2460733 | 0.16445623 |
| 851 | 0 | 0.25920245 | 0.09897172 | 0.68561224 | 0.33122172 | 0.12844037 | 0.33465504 | 0.12827225 | 0.12997347 |
| 852 | 0 | 0.16411043 | 0.11311054 | 0.93846939 | 0.25158371 | 0 | 0.33544806 | 0.11518325 | 0.10079576 |
| 853 | 0 | 0.29601227 | 0.12339332 | 0.92479592 | 0.29683258 | 0 | 0.29024584 | 0.2513089 | 0.20424403 |
| 854 | 0 | 0.09355828 | 0.07840617 | 0.63520408 | 0.16470588 | 0.13761468 | 0.33624108 | 0 | 0 |
| 855 | 0 | 0.63190184 | 0.16580977 | 0.74561224 | 0.25701357 | 0.53211009 | 0.56225218 | 0.06806283 | 0.06896552 |
| 856 | 0 | 0.48006135 | 0.31105398 | 0.76285714 | 0.40361991 | 0.12844037 | 0.41395718 | 0.13612565 | 0.07427056 |
| 857 | 0 | 0.1595092 | 0.10025707 | 0.70265306 | 0.2361991 | 0 | 0.31007137 | 0.06806283 | 0.03448276 |
| 858 | 0 | 0.11503067 | 0.26478149 | 0.50959184 | 0.2959276 | 0.08715596 | 0.20063442 | 0.02356021 | 0 |
| 859 | 1 | 0.64877301 | 0.21465296 | 0.92 | 0.60995475 | 0.12844037 | 0.57256146 | 0.60471204 | 0.49602122 |
| 860 | 0 | 0.11809816 | 0.13881748 | 0.95989796 | 0.26606335 | 0.21100917 | 0.2815226 | 0 | 0 |
| 861 | 0 | 0.29907975 | 0.31362468 | 0.69857143 | 0.39819005 | 0.11009174 | 0.33862014 | 0.06282723 | 0.09814324 |
| 862 | 1 | 0.26687117 | 0.01028278 | 0.76153061 | 0.08235294 | 0.15137615 | 0.3481364 | 0.10732984 | 0.08753316 |
| 863 | 0 | 0.32055215 | 0.14910026 | 0.93346939 | 0.3438914 | 0.0733945 | 0.30769231 | 0.18324607 | 0.14323607 |
| 864 | 0 | 0.17638037 | 0.07326478 | 0.64183673 | 0.3638009 | 0 | 0.36478985 | 0.14921466 | 0.15119363 |
| 865 | 1 | 0.40490798 | 0.27120823 | 0.73612245 | 0.44253394 | 0.18348624 | 0.38778747 | 0.27748691 | 0.24403183 |
| 866 | 0 | 0.48006135 | 0.37017995 | 0.75408163 | 0.36561086 | 0.20642202 | 0.53211737 | 0.22513089 | 0.1061008 |
| 867 | 0 | 0.15184049 | 0.4151671 | 0.83989796 | 0.42624434 | 0.1146789 | 0.3148295 | 0.06544503 | 0.066313 |

| 868 | 0 | 0.50613497 | 0.09125964 | 0.62540816 | 0.21357466 | 0.21559633 | 0.52339413 | 0 | 0 |
| --- | --- | --- | --- | --- | --- | --- | --- | --- | --- |
| 869 | 1 | 0.47546012 | 0.16452442 | 0.7822449 | 0.36561086 | 0.27981651 | 0.45440127 | 0.32722513 | 0.17771883 |
| 870 | 1 | 0.48312883 | 0.14267352 | 0.83061224 | 0.45972851 | 0.14678899 | 0.41871531 | 0.45811518 | 0.47214854 |
| 871 | 0 | 0.3297546 | 0.18380463 | 0.75755102 | 0.27058824 | 0.14220183 | 0.46788263 | 0.15445026 | 0.16976127 |
| 872 | 0 | 0.19018405 | 0.07455013 | 0.81336735 | 0.13846154 | 0.13302752 | 0.30689929 | 0.05759162 | 0.01856764 |
| 873 | 0 | 0.50153374 | 0.14395887 | 0.97867347 | 0.35746606 | 0.10091743 | 0.39095956 | 0.40575916 | 0.38992042 |
| 874 | 1 | 0.3190184 | 0.11439589 | 0.38193878 | 0.12307692 | 0.18348624 | 0.50118953 | 0.16230366 | 0.04509284 |
| 875 | 1 | 0.22852761 | 0.11311054 | 0.64479592 | 0.19004525 | 0.10091743 | 0.30610626 | 0.11518325 | 0.07427056 |
| 876 | 0 | 0.51840491 | 0.21722365 | 0.89520408 | 0.46696833 | 0.29357798 | 0.44567803 | 0.40575916 | 0.31564987 |
| 877 | 0 | 0.43251534 | 0.24164524 | 0.80265306 | 0.32579186 | 0.21559633 | 0.57097542 | 0.36910995 | 0.24933687 |
| 878 | 0 | 0.21319018 | 0.05912596 | 0.73030612 | 0.32488688 | 0.08715596 | 0.32751784 | 0.14136126 | 0.12732095 |
| 879 | 0 | 0.29754601 | 0.20051414 | 0.88132653 | 0.39909502 | 0.12385321 | 0.46471055 | 0.09947644 | 0.07161804 |
| 880 | 0 | 0.37423313 | 0.20565553 | 0.85326531 | 0.32488688 | 0.06880734 | 0.49088025 | 0.28010471 | 0.26259947 |
| 881 | 0 | 0.25460123 | 0.23393316 | 0.87591837 | 0.33393665 | 0.15137615 | 0.43536875 | 0.09162304 | 0.09018568 |
| 882 | 0 | 0.15490798 | 0.12467866 | 0.87285714 | 0.27873303 | 0.14678899 | 0.26962728 | 0.06806283 | 0.04244032 |
| 883 | 0 | 0.40184049 | 0.35475578 | 0.92479592 | 0.35475113 | 0.26605505 | 0.43774782 | 0.05759162 | 0.05835544 |
| 884 | 0 | 0.4309816 | 0.20565553 | 0.84306122 | 0.27782805 | 0.18348624 | 0.40285488 | 0.31413613 | 0.38992042 |
| 885 | 0 | 0.3696319 | 0.29177378 | 0.8055102 | 0.42262443 | 0.13302752 | 0.41237113 | 0.2408377 | 0.17771883 |
| 886 | 0 | 0.32055215 | 0.19280206 | 0.75918367 | 0.34932127 | 0.09633028 | 0.41633624 | 0.18324607 | 0.18567639 |
| 887 | 0 | 0.32668712 | 0.33676093 | 0.79632653 | 0.47782805 | 0.17431193 | 0.40999207 | 0.20942408 | 0.21220159 |
| 888 | 1 | 0.23619632 | 0.14395887 | 0.94173469 | 0.37828054 | 0.18807339 | 0.35844568 | 0.09424084 | 0.04774536 |
| 889 | 0 | 0.37883436 | 0.09897172 | 0.91734694 | 0.35113122 | 0.17889908 | 0.42030135 | 0.18062827 | 0.12732095 |
| 890 | 0 | 0.29294479 | 0.2403599 | 0.81336735 | 0.37556561 | 0.11926606 | 0.42743854 | 0.07329843 | 0.03713528 |
| 891 | 1 | 0.29141104 | 0.17609254 | 0.88785714 | 0.36923077 | 0.04587156 | 0.43774782 | 0.2513089 | 0.24137931 |
| 892 | 1 | 0.23773006 | 0.22879177 | 0.87020408 | 0.21719457 | 0.12844037 | 0.44171293 | 0.02094241 | 0.02122016 |
| 893 | 1 | 0.36196319 | 0.34832905 | 0.67795918 | 0.39457014 | 0.16513761 | 0.36241079 | 0.13089005 | 0.05570292 |
| 894 | 0 | 0.18711656 | 0.12467866 | 0.68591837 | 0.26425339 | 0.11009174 | 0.28945282 | 0 | 0 |
| 895 | 0 | 0.29294479 | 0.23521851 | 0.92 | 0.33031674 | 0.14220183 | 0.37272006 | 0.13874346 | 0.1061008 |
| 896 | 0 | 0.02147239 | 0.18380463 | 0.55244898 | 0.16832579 | 0 | 0.22759715 | 0 | 0 |
| 897 | 0 | 0.46472393 | 0.44601542 | 0.70081633 | 0.33755656 | 0.23853211 | 0.481364 | 0.04450262 | 0 |
| 898 | 0 | 0.42944785 | 0.21079692 | 0.62663265 | 0.239819 | 0.28440367 | 0.63600317 | 0.12303665 | 0.06100796 |

| 899 | 1 | 0.26993865 | 0.18766067 | 0.8344898 | 0.26696833 | 0.14220183 | 0.37351308 | 0.06282723 | 0.03448276 |
| --- | --- | --- | --- | --- | --- | --- | --- | --- | --- |
| 900 | 0 | 0.34969325 | 0.1940874 | 0.92591837 | 0.33936652 | 0.17889908 | 0.37192704 | 0.10994764 | 0.07427056 |
| 901 | 0 | 0.21165644 | 0.10154242 | 0.91285714 | 0.10769231 | 0 | 0.28231562 | 0.10471204 | 0.1061008 |
| 902 | 1 | 0.26380368 | 0.08997429 | 0.95408163 | 0.27963801 | 0.17889908 | 0.33544806 | 0.04450262 | 0.066313 |
| 903 | 0 | 0.2392638 | 0.21722365 | 0.79316327 | 0.31855204 | 0.05963303 | 0.3925456 | 0.2382199 | 0.20689655 |
| 904 | 0 | 0.39723926 | 0.25449871 | 0.89765306 | 0.44162896 | 0.34862385 | 0.47184774 | 0.28010471 | 0.32360743 |
| 905 | 0 | 0.32668712 | 0.09897172 | 0.92581633 | 0.2361991 | 0.13761468 | 0.36637589 | 0.18586387 | 0.22015915 |
| 906 | 0 | 0.61042945 | 0.35861183 | 0.76744898 | 0.40452489 | 0.29357798 | 0.66217288 | 0.2513089 | 0.20954907 |
| 907 | 0 | 0.10276074 | 0.1529563 | 0.65173469 | 0.17466063 | 0.06880734 | 0.24742268 | 0.03926702 | 0.01856764 |
| 908 | 0 | 0.30521472 | 0.3033419 | 0.58632653 | 0.44343891 | 0.18807339 | 0.43219667 | 0.11518325 | 0.06896552 |
| 909 | 0 | 0.36656442 | 0.18508997 | 0.70040816 | 0.21628959 | 0.14678899 | 0.32910389 | 0.29319372 | 0.21220159 |
| 910 | 0 | 0.31595092 | 0.3470437 | 0.81265306 | 0.47239819 | 0.06422018 | 0.370341 | 0.28795812 | 0.19363395 |
| 911 | 0 | 0.27607362 | 0.12210797 | 0.52357143 | 0.30045249 | 0 | 0.40602696 | 0.14921466 | 0.15119363 |
| 912 | 0 | 0.34202454 | 0.10796915 | 0.72918367 | 0.25248869 | 0.25688073 | 0.39888977 | 0.36649215 | 0.5198939 |
| 913 | 0 | 0.36042945 | 0.28920308 | 0.89989796 | 0.42443439 | 0.3440367 | 0.42426646 | 0.07329843 | 0.07427056 |
| 914 | 0 | 0.26993865 | 0.32133676 | 0.92306122 | 0.39909502 | 0.2706422 | 0.36082474 | 0.23036649 | 0.19363395 |
| 915 | 0 | 0.39110429 | 0.08226221 | 0.40428571 | 0.15384615 | 0.29357798 | 0.54004758 | 0.22251309 | 0.16976127 |
| 916 | 0 | 0.32668712 | 0.20822622 | 0.68520408 | 0.30316742 | 0.04587156 | 0.36161776 | 0.15968586 | 0.0530504 |
| 917 | 1 | 0.71932515 | 0.23521851 | 0.86581633 | 0.42443439 | 0.44954128 | 0.63203807 | 0.43455497 | 0.31564987 |
| 918 | 1 | 0.50306748 | 0.37017995 | 0.85285714 | 0.41809955 | 0.1146789 | 0.5630452 | 0.33769634 | 0.34217507 |
| 919 | 0 | 0.93558282 | 0.44858612 | 0.75265306 | 0.52579186 | 0.53211009 | 0.89928628 | 0.53141361 | 0.38461538 |
| 920 | 1 | 0.43251534 | 0.25321337 | 0.85367347 | 0.45882353 | 0.13761468 | 0.40047581 | 0.27486911 | 0.20689655 |
| 921 | 0 | 0.32361963 | 0.14395887 | 0.94928571 | 0.23891403 | 0.14220183 | 0.37668517 | 0.19895288 | 0.15384615 |
| 922 | 0 | 0.30981595 | 0.03727506 | 0.78295918 | 0.18280543 | 0.13302752 | 0.46946868 | 0.22774869 | 0.30769231 |
| 923 | 0 | 0.3696319 | 0.18766067 | 0.89479592 | 0.25701357 | 0.23853211 | 0.40840603 | 0.22513089 | 0.18302387 |
| 924 | 0 | 0.43558282 | 0.4562982 | 0.7805102 | 0.69321267 | 0.12844037 | 0.46153846 | 0.2591623 | 0.22546419 |
| 925 | 1 | 0.38650307 | 0.20951157 | 0.96428571 | 0.36108597 | 0 | 0.41950833 | 0.2565445 | 0.21485411 |
| 926 | 0 | 0.25306748 | 0.17609254 | 0.98979592 | 0.31674208 | 0.14678899 | 0.29341792 | 0.07329843 | 0.04244032 |
| 927 | 0 | 0.36809816 | 0.18123393 | 0.94887755 | 0.25520362 | 0.1146789 | 0.40047581 | 0.39005236 | 0.32891247 |
| 928 | 0 | 0.32668712 | 0.10539846 | 0.88142857 | 0.21538462 | 0.20642202 | 0.41237113 | 0.23560209 | 0.19628647 |
| 929 | 0 | 0.23619632 | 0.07840617 | 0.97520408 | 0.15294118 | 0 | 0.2926249 | 0.03926702 | 0.0397878 |

| 930 | 0 | 0.16257669 | 0.0874036 | 0.68632653 | 0.16832579 | 0.05504587 | 0.56145916 | 0.04973822 | 0.05039788 |
| --- | --- | --- | --- | --- | --- | --- | --- | --- | --- |
| 931 | 0 | 0.37116564 | 0.26478149 | 0.55836735 | 0.34027149 | 0.20642202 | 0.46867565 | 0.28272251 | 0.21485411 |
| 932 | 0 | 0.33895706 | 0.14781491 | 0.88142857 | 0.27420814 | 0.15137615 | 0.370341 | 0.23560209 | 0.28381963 |
| 933 | 0 | 0.05981595 | 0.25192802 | 0.81663265 | 0.31945701 | 0 | 0.37351308 | 0 | 0 |
| 934 | 0 | 0.34202454 | 0.16580977 | 0.94571429 | 0.32760181 | 0.14220183 | 0.36875496 | 0.19895288 | 0.15649867 |
| 935 | 0 | 0.17638037 | 0.29562982 | 0.55663265 | 0.34660633 | 0 | 0.27359239 | 0.09947644 | 0.10079576 |
| 936 | 0 | 0.61503067 | 0.1722365 | 0.92102041 | 0.19366516 | 0.36697248 | 0.55114988 | 0.35078534 | 0.49602122 |
| 937 | 0 | 0.44018405 | 0.48843188 | 0.53744898 | 0.54841629 | 0.33944954 | 0.47581285 | 0.22513089 | 0.15915119 |
| 938 | 0 | 0.2791411 | 0.21208226 | 0.86306122 | 0.3321267 | 0.12844037 | 0.36558287 | 0.16492147 | 0.09018568 |
| 939 | 0 | 0.43711656 | 0.27249357 | 0.94765306 | 0.30769231 | 0.08256881 | 0.36478985 | 0.43455497 | 0.19628647 |
| 940 | 0 | 0.38650307 | 0.18508997 | 0.67132653 | 0.26244344 | 0.14678899 | 0.4702617 | 0.26701571 | 0.20424403 |
| 941 | 0 | 0.41411043 | 0.11568123 | 0.8644898 | 0.23800905 | 0.16055046 | 0.44964314 | 0.36125654 | 0.38461538 |
| 942 | 0 | 0.41564417 | 0.27120823 | 0.72632653 | 0.36742081 | 0.06880734 | 0.46550357 | 0.29581152 | 0.25994695 |
| 943 | 0 | 0.1196319 | 0.20051414 | 0.83469388 | 0.2678733 | 0.14220183 | 0.38382236 | 0.04188482 | 0.04244032 |
| 944 | 0 | 0.32668712 | 0.16452442 | 0.56183673 | 0.28868778 | 0.19724771 | 0.37113402 | 0.27748691 | 0.39522546 |
| 945 | 0 | 0.39570552 | 0.18894602 | 0.59102041 | 0.29954751 | 0.10550459 | 0.44885012 | 0.26439791 | 0.18302387 |
| 946 | 0 | 0.30214724 | 0.30848329 | 0.71061224 | 0.48235294 | 0.24311927 | 0.47581285 | 0.15183246 | 0.11405836 |
| 947 | 0 | 0.35889571 | 0.30077121 | 1 | 0.28235294 | 0.15137615 | 0.38858049 | 0.2617801 | 0.23607427 |
| 948 | 0 | 0.25153374 | 0.23007712 | 0.89979592 | 0.35022624 | 0.13761468 | 0.27121332 | 0.19371728 | 0.15915119 |
| 949 | 1 | 0.67177914 | 0.16323907 | 0.59265306 | 0.49773756 | 0.39449541 | 0.60111023 | 0.59685864 | 0.50928382 |
| 950 | 0 | 0.09815951 | 0.10025707 | 0.04602041 | 0.2081448 | 0.05963303 | 0.23156225 | 0.06544503 | 0.01061008 |
| 951 | 0 | 0.5506135 | 0.24807198 | 0.75510204 | 0.47511312 | 0.30733945 | 0.60190325 | 0.22251309 | 0.04774536 |
| 952 | 1 | 0.16411043 | 0.07583548 | 0.93744898 | 0.16108597 | 0.05504587 | 0.30055511 | 0.04712042 | 0.01591512 |
| 953 | 1 | 0.50306748 | 0.22107969 | 0.91346939 | 0.31945701 | 0.22477064 | 0.39651071 | 0.28010471 | 0.19628647 |
| 954 | 0 | 0.25 | 0.22107969 | 0.94561224 | 0.29411765 | 0.33027523 | 0.40840603 | 0.09424084 | 0.1193634 |
| 955 | 0 | 0.27147239 | 0.13753213 | 0.67 | 0.24524887 | 0.1146789 | 0.40602696 | 0.14921466 | 0.10079576 |
| 956 | 0 | 0.24386503 | 0.13624679 | 0.31234694 | 0.18461538 | 0.13761468 | 0.38937351 | 0.09947644 | 0.02122016 |
| 957 | 0 | 0.19478528 | 0.18251928 | 0.89510204 | 0.26244344 | 0.1146789 | 0.37192704 | 0.06806283 | 0.0795756 |
| 958 | 0 | 0.18865031 | 0.1311054 | 0.69387755 | 0.46334842 | 0 | 0.2926249 | 0.21465969 | 0.27055703 |
| 959 | 0 | 0.28527607 | 0.11182519 | 0.77387755 | 0.12307692 | 0.2293578 | 0.55035686 | 0.06544503 | 0.03183024 |
| 960 | 0 | 0.24233129 | 0.21336761 | 0.72081633 | 0.38914027 | 0.13761468 | 0.39095956 | 0.09162304 | 0.06896552 |

| 961 | 0 | 0.47392638 | 0.09383033 | 0.50387755 | 0.39004525 | 0.15137615 | 0.49008723 | 0.42670157 | 0.49602122 |
| --- | --- | --- | --- | --- | --- | --- | --- | --- | --- |
| 962 | 0 | 0.44325153 | 0.1748072 | 0.65132653 | 0.41085973 | 0.24311927 | 0.50277557 | 0.38481675 | 0.31034483 |
| 963 | 0 | 0.37269939 | 0.29048843 | 0.67173469 | 0.34570136 | 0 | 0.48215702 | 0.04450262 | 0.04509284 |
| 964 | 0 | 0.40797546 | 0.28020566 | 0.85316327 | 0.28506787 | 0.11009174 | 0.38382236 | 0.37958115 | 0.25729443 |
| 965 | 0 | 0.15337423 | 0 | 0.99734694 | 0.14479638 | 0.09174312 | 0.52260111 | 0.10471204 | 0.1061008 |
| 966 | 0 | 0.35122699 | 0.20308483 | 0.87816327 | 0.31131222 | 0.18807339 | 0.37272006 | 0.12565445 | 0.09814324 |
| 967 | 0 | 0.30981595 | 0.19280206 | 0.74020408 | 0.32669683 | 0.1146789 | 0.44488501 | 0.19633508 | 0.12997347 |
| 968 | 0 | 0.32515337 | 0.06812339 | 0.8827551 | 0.24524887 | 0.16055046 | 0.31800159 | 0.09162304 | 0.07692308 |
| 969 | 0 | 0.32822086 | 0.21208226 | 0.80795918 | 0.37375566 | 0.15137615 | 0.39651071 | 0.26439791 | 0.26790451 |
| 970 | 0 | 0.46932515 | 0.2840617 | 0.83387755 | 0.36199095 | 0.3853211 | 0.48453608 | 0.2486911 | 0.11140584 |
| 971 | 0 | 0.53374233 | 0.22750643 | 0.71489796 | 0.46877828 | 0.13761468 | 0.57097542 | 0.47905759 | 0.50132626 |
| 972 | 1 | 0.17331288 | 0.18380463 | 0.52857143 | 0.239819 | 0.07798165 | 0.26724822 | 0.07591623 | 0.0397878 |
| 973 | 1 | 0.48006135 | 0.54884319 | 0.56510204 | 0.54479638 | 0.16972477 | 0.481364 | 0.15183246 | 0.07161804 |
| 974 | 1 | 0.42177914 | 0.42287918 | 0.58193878 | 0.4280543 | 0.20642202 | 0.46471055 | 0.09424084 | 0.02387268 |
| 975 | 0 | 0.31134969 | 0.26092545 | 0.99316327 | 0.26515837 | 0.20642202 | 0.35765266 | 0 | 0 |
| 976 | 0 | 0.20858896 | 0.11696658 | 0.93673469 | 0.26696833 | 0.20642202 | 0.25218081 | 0.17801047 | 0.18037135 |
| 977 | 1 | 0.38803681 | 0.20308483 | 0.82142857 | 0.24977376 | 0.38990826 | 0.37272006 | 0.03403141 | 0.03448276 |
| 978 | 1 | 0.47392638 | 0.17866324 | 0.80683673 | 0.46696833 | 0.16972477 | 0.49405234 | 0.43717277 | 0.40848806 |
| 979 | 0 | 0.26380368 | 0.27763496 | 0.42030612 | 0.29321267 | 0.13302752 | 0.5741475 | 0.02094241 | 0.01061008 |
| 980 | 1 | 0.66257669 | 0.1092545 | 0.46204082 | 0.56742081 | 0.24770642 | 0.58287074 | 0.78534031 | 0.77984085 |
| 981 | 1 | 0.22392638 | 0.20179949 | 0.88244898 | 0.29502262 | 0.17889908 | 0.36558287 | 0.04450262 | 0.07427056 |
| 982 | 0 | 0.30828221 | 0.24550129 | 0.42989796 | 0.42081448 | 0.08256881 | 0.41237113 | 0.12041885 | 0.12201592 |
| 983 | 0 | 0.28527607 | 0.28920308 | 0.8555102 | 0.3438914 | 0.20183486 | 0.42743854 | 0.13874346 | 0.1061008 |
| 984 | 1 | 0.34355828 | 0.32390746 | 0.80295918 | 0.43529412 | 0.17431193 | 0.42109437 | 0.2434555 | 0.18832891 |
| 985 | 1 | 0.52300613 | 0.17866324 | 0.64510204 | 0.22262443 | 0.35321101 | 0.49167328 | 0.28795812 | 0.06896552 |
| 986 | 0 | 0.20245399 | 0.15552699 | 0.85897959 | 0.239819 | 0.10091743 | 0.41157811 | 0.17277487 | 0.11671088 |
| 987 | 0 | 0.15030675 | 0.14524422 | 0.43642857 | 0.18371041 | 0 | 0.35765266 | 0.07853403 | 0.06100796 |
| 988 | 0 | 0.32668712 | 0.09125964 | 0.96540816 | 0.27330317 | 0.16513761 | 0.4369548 | 0.23298429 | 0.14058355 |
| 989 | 0 | 0.34355828 | 0.07583548 | 0.7722449 | 0.2561086 | 0.09633028 | 0.45281523 | 0.22251309 | 0.31299735 |
| 990 | 1 | 0.15797546 | 0.1503856 | 0.62959184 | 0.37285068 | 0.08256881 | 0.34972244 | 0.07068063 | 0.01856764 |
| 991 | 0 | 0.21779141 | 0.3033419 | 0.59979592 | 0.29864253 | 0.14220183 | 0.34892942 | 0.08115183 | 0 |

| 992 | 0 | 0 | 0.33933162 | 0.56938776 | 0.39909502 | 0 | 0.13957177 | 0 | 0 |
| --- | --- | --- | --- | --- | --- | --- | --- | --- | --- |
| 993 | 1 | 0.44171779 | 0.14010283 | 0.64530612 | 0.239819 | 0.33027523 | 0.48374306 | 0.17277487 | 0.11405836 |
| 994 | 1 | 0.40337423 | 0.26606684 | 0.78397959 | 0.41900452 | 0.15137615 | 0.41871531 | 0.27486911 | 0.28116711 |
| 995 | 0 | 0.11809816 | 0.24807198 | 0.51326531 | 0.41809955 | 0.08715596 | 0.22918319 | 0.15183246 | 0.15384615 |
| 996 | 0 | 0.18251534 | 0.03727506 | 0.32744898 | 0.11493213 | 0.05963303 | 0.35051546 | 0.04973822 | 0.05039788 |
| 997 | 1 | 0.27147239 | 0.22750643 | 0.98255102 | 0.27782805 | 0.31192661 | 0.37272006 | 0.10732984 | 0.03713528 |
| 998 | 0 | 0.39110429 | 0.25706941 | 0.67673469 | 0.40090498 | 0.16972477 | 0.43457573 | 0.13612565 | 0.15915119 |
| 999 | 0 | 0.35736196 | 0.24550129 | 0.54938776 | 0.30769231 | 0.11926606 | 0.34179223 | 0.13612565 | 0.0795756 |
| 1000 | 0 | 0.38343558 | 0.22236504 | 0.89316327 | 0.27782805 | 0.30733945 | 0.47184774 | 0.22513089 | 0.15384615 |

1

| sexual | feel | death | body | anx |
| --- | --- | --- | --- | --- |
| 0 | 0.15768463 | 0.03167421 | 0.26806527 | 0.11356467 |
| 0.08550186 | 0.11776447 | 0.05882353 | 0.24475524 | 0.12302839 |
| 0 | 0.09780439 | 0.07239819 | 0.08857809 | 0.0851735 |
| 0 | 0.05588822 | 0 | 0.13286713 | 0.08832808 |
| 0.10037175 | 0.10578842 | 0 | 0.12354312 | 0 |
| 0.32342007 | 0.1996008 | 0.1040724 | 0.30769231 | 0.01577287 |
| 0.0929368 | 0.31936128 | 0 | 0.17249417 | 0.07886435 |
| 0.12267658 | 0.08383234 | 0.06334842 | 0.15384615 | 0.07886435 |
| 0.2267658 | 0.22754491 | 0.05882353 | 0.37762238 | 0.0126183 |
| 0.05576208 | 0.36526946 | 0 | 0.21212121 | 0.28706625 |
| 0.04460967 | 0.18163673 | 0 | 0.13519814 | 0.09148265 |
| 0.05947955 | 0.13173653 | 0.07692308 | 0.32867133 | 0.14511041 |
| 0.18215613 | 0.03193613 | 0 | 0.07692308 | 0.10410095 |
| 0.14498141 | 0.12175649 | 0.02714932 | 0.19347319 | 0.15772871 |
| 0 | 0.11976048 | 0.13574661 | 0.31468531 | 0.09463722 |
| 0.05576208 | 0.26347305 | 0 | 0.37762238 | 0.09148265 |
| 0.10408922 | 0.16966068 | 0.04072398 | 0.13286713 | 0.40378549 |
| 0.08921933 | 0.15169661 | 0.05429864 | 0.16783217 | 0.12618297 |
| 0.01486989 | 0.13373253 | 0.00904977 | 0.11421911 | 0.13564669 |
| 0.08550186 | 0.18762475 | 0.0678733 | 0.21212121 | 0.20504732 |
| 0.26394052 | 0.11776447 | 0.26696833 | 0.3030303 | 0.03785489 |
| 0.04460967 | 0.15169661 | 0 | 0.37995338 | 0.1829653 |
| 0 | 0.16966068 | 0 | 0.11888112 | 0.2681388 |
| 0.08921933 | 0.4251497 | 0.07239819 | 0.42191142 | 0.08832808 |
| 0.1598513 | 0.08582834 | 0.04072398 | 0.1002331 | 0.13564669 |
| 0.31226766 | 0.15768463 | 0.17647059 | 0.36829837 | 0.06309148 |
| 0.17472119 | 0.31936128 | 0.04072398 | 0.39160839 | 0.07886435 |
| 0.0260223 | 0.1497006 | 0.0678733 | 0.0955711 | 0.17665615 |
| 0.02973978 | 0.1996008 | 0.0361991 | 0.44755245 | 0.23659306 |
| 0.07806691 | 0.17165669 | 0 | 0.2004662 | 0.12302839 |

| 0.05947955 | 0.06387226 | 0 | 0.41491841 | 0.15141956 |
| --- | --- | --- | --- | --- |
| 0.01858736 | 0.09780439 | 0.02262443 | 0.06759907 | 0.03154574 |
| 0 | 0.21556886 | 0 | 0.16783217 | 0.05678233 |
| 0.17100372 | 0.12175649 | 0.1719457 | 0.21445221 | 0.14511041 |
| 0.0260223 | 0.16966068 | 0.03167421 | 0.09090909 | 0.02208202 |
| 0 | 0.08582834 | 0 | 0.1981352 | 0.20189274 |
| 0.133829 | 0.15169661 | 0.05882353 | 0.25174825 | 0.08832808 |
| 0.23048327 | 0.3493014 | 0.07692308 | 0.14685315 | 0.23974763 |
| 0.11152416 | 0.30738523 | 0.05882353 | 0.58041958 | 0.13880126 |
| 0.11895911 | 0.28942116 | 0 | 0.26340326 | 0 |
| 0.04832714 | 0.18363273 | 0.04072398 | 0.15384615 | 0.15141956 |
| 0.15613383 | 0.34530938 | 0.05429864 | 0.18181818 | 0.28391167 |
| 0 | 0.18562874 | 0 | 0.05361305 | 0.09148265 |
| 0.00743494 | 0.05788423 | 0.19004525 | 0.01631702 | 0.08832808 |
| 0 | 0.20758483 | 0.02262443 | 0.17948718 | 0.14826498 |
| 0.05947955 | 0.14171657 | 0.09502262 | 0.12820513 | 0.17981073 |
| 0.01486989 | 0.11377246 | 0 | 0.05128205 | 0.13880126 |
| 0.13754647 | 0.09181637 | 0.04072398 | 0.64102564 | 0.11671924 |
| 0.10408922 | 0.20558882 | 0.06334842 | 0.38927739 | 0.02523659 |
| 0.04832714 | 0.20359281 | 0.01809955 | 0.17948718 | 0.09463722 |
| 0.26765799 | 0.20558882 | 0.18552036 | 0.21678322 | 0.12933754 |
| 0.21189591 | 0.11377246 | 0.18552036 | 0.26573427 | 0.12933754 |
| 0 | 0.49101796 | 0 | 0.22843823 | 0.27129338 |
| 0.24535316 | 0.06586826 | 0.07692308 | 0.30769231 | 0.05362776 |
| 0.02973978 | 0.19361277 | 0.09954751 | 0.13752914 | 0.11987382 |
| 0 | 0.11177645 | 0.04524887 | 0.17715618 | 0.07886435 |
| 0.16728625 | 0.15768463 | 0 | 0.02564103 | 0.21451104 |
| 0.01486989 | 0.33333333 | 0.07692308 | 0.20979021 | 0.30914826 |
| 0.07806691 | 0.08383234 | 0 | 0.06526807 | 0.28706625 |
| 0 | 0.16966068 | 0 | 0.13286713 | 0.14826498 |
| 0.55390335 | 0.1497006 | 0.14027149 | 0.18881119 | 0.17665615 |

| 0 | 0.1497006 | 0 | 0.21911422 | 0 |
| --- | --- | --- | --- | --- |
| 0.11152416 | 0.17964072 | 0 | 0.06993007 | 0.09463722 |
| 0.01486989 | 0.16966068 | 0 | 0.08857809 | 0.16088328 |
| 0 | 0.23153693 | 0.08597285 | 0.19347319 | 0.4384858 |
| 0.05947955 | 0.15768463 | 0 | 0.22144522 | 0.19873817 |
| 0.01858736 | 0.50499002 | 0.23529412 | 0.56410256 | 0.12933754 |
| 0.11152416 | 0.17165669 | 0.08144796 | 0.23076923 | 0.06624606 |
| 0.10408922 | 0.10778443 | 0.02714932 | 0.13752914 | 0.05678233 |
| 0.01486989 | 0.10978044 | 0.07692308 | 0.15850816 | 0.09463722 |
| 0.02973978 | 0.11776447 | 0 | 0.1958042 | 0.09148265 |
| 0.04832714 | 0.21357285 | 0.02262443 | 0.15384615 | 0.08832808 |
| 0.04832714 | 0.36926148 | 0.05882353 | 0.09324009 | 0.04100946 |
| 0.07434944 | 0.15768463 | 0.18099548 | 0.18414918 | 0.12618297 |
| 0.15241636 | 0.10179641 | 0.04524887 | 0.23543124 | 0.16088328 |
| 0.10780669 | 0.19361277 | 0.13122172 | 0.06759907 | 0.03154574 |
| 0.03717472 | 0.26946108 | 0 | 0.36130536 | 0.05993691 |
| 0.04089219 | 0.16566866 | 0.0361991 | 0.13519814 | 0.06309148 |
| 0.0929368 | 0.23752495 | 0.01809955 | 0.17482517 | 0.10094637 |
| 0.07806691 | 0.18163673 | 0.02262443 | 0.21212121 | 0.14826498 |
| 0.09665428 | 0.10978044 | 0.03167421 | 0.25641026 | 0.09148265 |
| 0 | 0.10978044 | 0.08144796 | 0.12820513 | 0.11671924 |
| 0.27509294 | 0.26546906 | 0.15837104 | 0.29137529 | 0.22082019 |
| 0.08921933 | 0.16566866 | 0 | 0.08158508 | 0.08201893 |
| 0.03717472 | 0.30938124 | 0.02262443 | 0.22144522 | 0.15772871 |
| 0 | 0.30538922 | 0.06334842 | 0.14685315 | 0.04416404 |
| 0.07434944 | 0.16766467 | 0.05429864 | 0.20979021 | 0.15772871 |
| 0.15241636 | 0.15568862 | 0.19004525 | 0.16317016 | 0.11041009 |
| 0.08550186 | 0.22355289 | 0.04524887 | 0.2004662 | 0.06309148 |
| 0 | 0.47904192 | 0 | 0.33566434 | 0.30283912 |
| 0.0260223 | 0.1497006 | 0 | 0.1048951 | 0.02208202 |
| 0.01486989 | 0.1996008 | 0.09954751 | 0.12587413 | 0.13564669 |

| 0.13754647 | 0.15169661 | 0.04072398 | 0.13986014 | 0.06624606 |
| --- | --- | --- | --- | --- |
| 0.0669145 | 0.12175649 | 0 | 0.22843823 | 0.01892744 |
| 0.01858736 | 0.14371257 | 0.04524887 | 0.08857809 | 0.01577287 |
| 0.04832714 | 0.17365269 | 0 | 0.16550117 | 0.07570978 |
| 0.04460967 | 0.10578842 | 0.07239819 | 0.15850816 | 0.04416404 |
| 0.0669145 | 0.14171657 | 0.15837104 | 0.19114219 | 0.0977918 |
| 0.07434944 | 0.14371257 | 0 | 0.25174825 | 0.10410095 |
| 0.05947955 | 0.32734531 | 0.07239819 | 0.34498834 | 0.12302839 |
| 0.07063197 | 0.23952096 | 0.0678733 | 0.49417249 | 0.11041009 |
| 0.01486989 | 0.13972056 | 0.06334842 | 0.18181818 | 0.21766562 |
| 0.10780669 | 0.09780439 | 0.04524887 | 0.06759907 | 0.09148265 |
| 0.04460967 | 0.22754491 | 0 | 0.13986014 | 0.07570978 |
| 0.05204461 | 0.12175649 | 0.06334842 | 0.20512821 | 0.27760252 |
| 0.08178439 | 0.17165669 | 0.04977376 | 0.17016317 | 0.06309148 |
| 0.21561338 | 0.30738523 | 0 | 0.31235431 | 0.05993691 |
| 0.0929368 | 0.23952096 | 0 | 0.21212121 | 0.06624606 |
| 0.49814126 | 0.21956088 | 0.06334842 | 0.21911422 | 0.08201893 |
| 0 | 0.08183633 | 0.06334842 | 0.31701632 | 0.0851735 |
| 0.04832714 | 0.26946108 | 0.0361991 | 0.29137529 | 0.0977918 |
| 0.13754647 | 0.19760479 | 0.05429864 | 0.31934732 | 0.07886435 |
| 0.07063197 | 0.14171657 | 0 | 0.07459207 | 0.01892744 |
| 0.13011152 | 0.14171657 | 0.07239819 | 0.17948718 | 0.08201893 |
| 0.05204461 | 0.14371257 | 0.08144796 | 0.2004662 | 0.21766562 |
| 0.11895911 | 0.23153693 | 0 | 0.29370629 | 0.13249211 |
| 0.28252788 | 0.29540918 | 0.16289593 | 0.27039627 | 0.14195584 |
| 0 | 0.10578842 | 0.05882353 | 0.21678322 | 0.29337539 |
| 0.07806691 | 0.19361277 | 0.0678733 | 0.21212121 | 0.05678233 |
| 0.11152416 | 0.11976048 | 0.0678733 | 0.27039627 | 0.04731861 |
| 0.05576208 | 0.17964072 | 0.04524887 | 0.08158508 | 0.0851735 |
| 0.13754647 | 0.28343313 | 0.0678733 | 0.27738928 | 0.21135647 |
| 0.16728625 | 0.19760479 | 0.2760181 | 0.27039627 | 0.12933754 |

| 0.02973978 | 0.11377246 | 0.04524887 | 0.2027972 | 0.0977918 |
| --- | --- | --- | --- | --- |
| 0.13754647 | 0.18363273 | 0.02714932 | 0.24941725 | 0.13249211 |
| 0.14498141 | 0.1497006 | 0.13122172 | 0.25174825 | 0.05993691 |
| 0.03717472 | 0.11576846 | 0.02262443 | 0.14219114 | 0.10094637 |
| 0.10408922 | 0.09780439 | 0 | 0.29370629 | 0.06624606 |
| 0.05576208 | 0.39520958 | 0.05429864 | 0.22843823 | 0.19242902 |
| 0.06319703 | 0.26347305 | 0.21266968 | 0.43589744 | 0.2555205 |
| 0.17472119 | 0.2255489 | 0.07239819 | 0.27039627 | 0.15772871 |
| 0.39033457 | 0.31337325 | 0.05882353 | 0.64102564 | 0 |
| 0.10037175 | 0.19161677 | 0.01357466 | 0.24475524 | 0.11041009 |
| 0.14126394 | 0.17165669 | 0.0361991 | 0.24708625 | 0.20504732 |
| 0.07063197 | 0.13572854 | 0.04977376 | 0.17249417 | 0.17981073 |
| 0.1598513 | 0.13772455 | 0.01357466 | 0.17948718 | 0.05993691 |
| 0.15613383 | 0.26546906 | 0.07239819 | 0.51515152 | 0.08201893 |
| 0.06319703 | 0.20159681 | 0.07692308 | 0.35198135 | 0.04416404 |
| 0.14498141 | 0.16566866 | 0.05429864 | 0.19114219 | 0.22712934 |
| 0.0669145 | 0.18562874 | 0.03167421 | 0.18414918 | 0.13564669 |
| 0.133829 | 0.07984032 | 0.07239819 | 0.13053613 | 0.08832808 |
| 0.34572491 | 0.16167665 | 0.05429864 | 0.2027972 | 0.31230284 |
| 0.17843866 | 0.19161677 | 0.03167421 | 0.12820513 | 0.0851735 |
| 0.0929368 | 0.23952096 | 0.12217195 | 0.21678322 | 0.16719243 |
| 0.04832714 | 0.23153693 | 0.13574661 | 0.20745921 | 0.06309148 |
| 0.21189591 | 0.16167665 | 0.04072398 | 0.23776224 | 0.05678233 |
| 0.04832714 | 0.23353293 | 0.04072398 | 0.32167832 | 0.06940063 |
| 0.12267658 | 0.13173653 | 0.0361991 | 0.30536131 | 0.07886435 |
| 0.10037175 | 0.20758483 | 0.02262443 | 0.11421911 | 0.01577287 |
| 0.14126394 | 0.08982036 | 0.11764706 | 0.19347319 | 0.05993691 |
| 0.31598513 | 0.25548902 | 0 | 0.62004662 | 0.03470032 |
| 0.08178439 | 0.18962076 | 0.02262443 | 0.27272727 | 0.13564669 |
| 0.16728625 | 0.21556886 | 0 | 0.20979021 | 0.0851735 |
| 0.16728625 | 0.1257485 | 0.21719457 | 0.26107226 | 0.11671924 |

| 0.35687732 | 0.10578842 | 0 | 0.44522145 | 0.06624606 |
| --- | --- | --- | --- | --- |
| 0.30483271 | 0.08183633 | 0.2760181 | 0.0955711 | 0.0977918 |
| 0.31598513 | 0.31536926 | 0.03167421 | 0.22843823 | 0.12302839 |
| 0 | 0.18163673 | 0 | 0.19114219 | 0.11671924 |
| 0.16356877 | 0.1996008 | 0.07692308 | 0.21911422 | 0.10410095 |
| 0.10780669 | 0.14770459 | 0.14932127 | 0.21445221 | 0.08201893 |
| 0.01115242 | 0.18762475 | 0.02262443 | 0.12587413 | 0.11041009 |
| 0.01115242 | 0.23353293 | 0.11312217 | 0.2960373 | 0.14826498 |
| 0.05576208 | 0.12175649 | 0.01357466 | 0.09324009 | 0.14511041 |
| 0.08178439 | 0.20359281 | 0.13122172 | 0.2027972 | 0 |
| 0.13011152 | 0.12974052 | 0.0678733 | 0.18881119 | 0.11671924 |
| 0.0929368 | 0.1257485 | 0 | 0.26573427 | 0.07886435 |
| 0.15613383 | 0.24550898 | 0.16289593 | 0.25874126 | 0.23659306 |
| 0.04460967 | 0.10179641 | 0.02714932 | 0.17715618 | 0.17350158 |
| 0.03717472 | 0.13572854 | 0.01809955 | 0.13053613 | 0.15141956 |
| 0.19702602 | 0.23752495 | 0.04977376 | 0.18648019 | 0.11356467 |
| 0.08550186 | 0.20758483 | 0.06334842 | 0.13752914 | 0.16088328 |
| 0.133829 | 0.14171657 | 0.00904977 | 0.34265734 | 0.16088328 |
| 0.33828996 | 0.14770459 | 0.0678733 | 0.25641026 | 0.0851735 |
| 0.0669145 | 0.31536926 | 0.01809955 | 0.43589744 | 0.10410095 |
| 0.01858736 | 0.23752495 | 0.00904977 | 0.24708625 | 0.13880126 |
| 0.2267658 | 0.20958084 | 0.09954751 | 0.25874126 | 0.12302839 |
| 0.17843866 | 0.18163673 | 0.11312217 | 0.24475524 | 0.03470032 |
| 0.07434944 | 0.1497006 | 0.02262443 | 0.20979021 | 0.01577287 |
| 0.04832714 | 0.17365269 | 0.02714932 | 0.21678322 | 0.05047319 |
| 0.28996283 | 0.13373253 | 0.09502262 | 0.24708625 | 0.10410095 |
| 0.39776952 | 0.13373253 | 0.13574661 | 0.24475524 | 0.06309148 |
| 0.11152416 | 0.14770459 | 0.1040724 | 0.17715618 | 0.08832808 |
| 0.69888476 | 0.19760479 | 0.09502262 | 0.43822844 | 0.08201893 |
| 0.15613383 | 0.22954092 | 0.04524887 | 0.31701632 | 0.0977918 |
| 0.05947955 | 0.17764471 | 0.02262443 | 0.11888112 | 0.20820189 |

| 0.08921933 | 0.13173653 | 0.10859729 | 0.0979021 | 0.11356467 |
| --- | --- | --- | --- | --- |
| 0.18587361 | 0.15968064 | 0.08144796 | 0.22144522 | 0.15141956 |
| 0.05576208 | 0.35329341 | 0 | 0.20745921 | 0.13880126 |
| 0.08921933 | 0.17365269 | 0.0361991 | 0.09090909 | 0.17350158 |
| 0.08178439 | 0.15768463 | 0.03167421 | 0.23310023 | 0.13249211 |
| 0.23048327 | 0.1756487 | 0.02262443 | 0.36130536 | 0.12933754 |
| 0.08921933 | 0.2255489 | 0.02714932 | 0.21445221 | 0.11671924 |
| 0.23791822 | 0.18962076 | 0 | 0.46853147 | 0.13249211 |
| 0.06319703 | 0.12774451 | 0.01357466 | 0.22843823 | 0.05362776 |
| 0.24163569 | 0.16167665 | 0.0361991 | 0.41724942 | 0.02523659 |
| 0.20817844 | 0.29740519 | 0.07692308 | 0.26340326 | 0.17665615 |
| 0.07434944 | 0.15968064 | 0 | 0.26806527 | 0.12618297 |
| 0.05576208 | 0.13173653 | 0.05429864 | 0.12587413 | 0.0851735 |
| 0.02230483 | 0.19361277 | 0.02714932 | 0.17948718 | 0.16719243 |
| 0.0669145 | 0.0998004 | 0.05429864 | 0.15850816 | 0.1955836 |
| 0.0669145 | 0.11776447 | 0.0361991 | 0.24009324 | 0.10410095 |
| 0.19330855 | 0.24550898 | 0.11764706 | 0.27039627 | 0.20504732 |
| 0 | 0.44710579 | 0.14027149 | 0.31934732 | 0.11671924 |
| 0.47211896 | 0.04391218 | 0.2760181 | 0.28438228 | 0.0126183 |
| 0.08550186 | 0.10379242 | 0.0361991 | 0.14685315 | 0.08201893 |
| 0.11895911 | 0.22155689 | 0.08597285 | 0.1002331 | 0.04416404 |
| 0.0260223 | 0.3493014 | 0.04524887 | 0.51748252 | 0.03154574 |
| 0.28252788 | 0.16966068 | 0.04072398 | 0.38228438 | 0.05047319 |
| 0.23420074 | 0.16966068 | 0.07692308 | 0.37995338 | 0.16088328 |
| 0.2527881 | 0.30538922 | 0 | 0.42424242 | 0.03470032 |
| 0.0929368 | 0.15369261 | 0.0361991 | 0.27039627 | 0.05993691 |
| 0.21189591 | 0.28143713 | 0.08144796 | 0.17016317 | 0.08201893 |
| 0.08178439 | 0.26347305 | 0.06334842 | 0.26573427 | 0.07886435 |
| 0.01115242 | 0.29141717 | 0.04524887 | 0.25407925 | 0.17350158 |
| 0.05204461 | 0 | 0.37556561 | 0.22610723 | 0.08832808 |
| 0.03345725 | 0.20758483 | 0.07692308 | 0.16083916 | 0.11041009 |

| 0.18959108 | 0.18363273 | 0.18552036 | 0.31934732 | 0.11987382 |
| --- | --- | --- | --- | --- |
| 0.09665428 | 0.11576846 | 0 | 0.08391608 | 0.17350158 |
| 0.0929368 | 0.15768463 | 0.0361991 | 0.17016317 | 0.05362776 |
| 0.11895911 | 0.19161677 | 0.06334842 | 0.34731935 | 0.03470032 |
| 0.33828996 | 0.16566866 | 0 | 0.40559441 | 0.11987382 |
| 0.02973978 | 0.15568862 | 0.19909502 | 0.23076923 | 0.09148265 |
| 0.08921933 | 0.16766467 | 0.14479638 | 0.17715618 | 0.07570978 |
| 0 | 0.17764471 | 0.01357466 | 0.11188811 | 0.26182965 |
| 0.17100372 | 0.20958084 | 0 | 0.33566434 | 0 |
| 0.05576208 | 0.16367265 | 0.04977376 | 0.14918415 | 0.03470032 |
| 0.01486989 | 0.09780439 | 0 | 0.16083916 | 0.02523659 |
| 0.05204461 | 0.12774451 | 0 | 0.18181818 | 0.08832808 |
| 0.12267658 | 0.21756487 | 0.11312217 | 0.27272727 | 0.13249211 |
| 0.13754647 | 0.21956088 | 0.03167421 | 0.15384615 | 0.09148265 |
| 0.21189591 | 0.25349301 | 0.07239819 | 0.28438228 | 0.08832808 |
| 0.23048327 | 0.20758483 | 0.01357466 | 0.24941725 | 0.2681388 |
| 0.04089219 | 0.1497006 | 0.04977376 | 0.07459207 | 0.20504732 |
| 0.07063197 | 0.18962076 | 0 | 0.14452214 | 0.16403785 |
| 0.01858736 | 0.19361277 | 0.01809955 | 0.12587413 | 0.21451104 |
| 0.25650558 | 0.21956088 | 0.08597285 | 0.27505828 | 0.19242902 |
| 0.133829 | 0.14171657 | 0.10859729 | 0.19347319 | 0.05678233 |
| 0.01486989 | 0.22355289 | 0.09502262 | 0.22144522 | 0.15457413 |
| 0.05204461 | 0.0738523 | 0.06334842 | 0.14219114 | 0.05362776 |
| 0.12267658 | 0.20159681 | 0.01809955 | 0.15384615 | 0.04416404 |
| 0.24535316 | 0.13173653 | 0.07239819 | 0.34498834 | 0.05047319 |
| 0.04460967 | 0.22355289 | 0.07692308 | 0.22144522 | 0.14195584 |
| 0.16728625 | 0.18163673 | 0.0361991 | 0.19347319 | 0.07255521 |
| 0.10780669 | 0.21357285 | 0.03167421 | 0.21678322 | 0.02208202 |
| 0.01486989 | 0.18762475 | 0.09502262 | 0.18881119 | 0.23028391 |
| 0.07434944 | 0.16966068 | 0.06334842 | 0.1981352 | 0.05362776 |
| 0.05947955 | 0.20758483 | 0.0361991 | 0.13053613 | 0.10094637 |

| 0.0929368 | 0.24151697 | 0.09049774 | 0.31002331 | 0.1955836 |
| --- | --- | --- | --- | --- |
| 0.02973978 | 0.19760479 | 0 | 0.28671329 | 0.07886435 |
| 0.16356877 | 0.1257485 | 0.03167421 | 0.14219114 | 0.07570978 |
| 0.03345725 | 0.20159681 | 0.02262443 | 0.36829837 | 0.03470032 |
| 0.0669145 | 0.20758483 | 0.09502262 | 0.34965035 | 0.170347 |
| 0.133829 | 0.08982036 | 0.05882353 | 0.14918415 | 0.07255521 |
| 0.12267658 | 0.13173653 | 0.0361991 | 0.27039627 | 0.06940063 |
| 0 | 0.07784431 | 0.08597285 | 0.31468531 | 0.05993691 |
| 0.23420074 | 0.17365269 | 0.04524887 | 0.17948718 | 0.11987382 |
| 0.47211896 | 0.07784431 | 0.0678733 | 0.18181818 | 0.04731861 |
| 0.02230483 | 0.16566866 | 0.08597285 | 0.31235431 | 0.04100946 |
| 0.04832714 | 0.16966068 | 0 | 0.09090909 | 0.36908517 |
| 0.05576208 | 0.23353293 | 0.04524887 | 0.24941725 | 0.23028391 |
| 0.133829 | 0.36726547 | 0.36651584 | 0.12587413 | 0.170347 |
| 0.14498141 | 0.07784431 | 0.0678733 | 0.07925408 | 0.04731861 |
| 0.12639405 | 0.43912176 | 0.15384615 | 0.35431235 | 0.21451104 |
| 0 | 0.33932136 | 0.1040724 | 0.1002331 | 0.11671924 |
| 0.16728625 | 0.16566866 | 0.1040724 | 0.29137529 | 0.14195584 |
| 0.17100372 | 0.20558882 | 0.02714932 | 0.20512821 | 0.13564669 |
| 0 | 0.07784431 | 0 | 0.22377622 | 0 |
| 0.12639405 | 0.21357285 | 0.02262443 | 0.21678322 | 0.170347 |
| 0.82527881 | 0.5489022 | 0.04977376 | 0.37062937 | 0.19873817 |
| 0.06319703 | 0.12175649 | 0.04977376 | 0.14219114 | 0.17350158 |
| 0.03345725 | 0.10379242 | 0.08597285 | 0.13286713 | 0.19242902 |
| 0.07434944 | 0.19760479 | 0 | 0.36363636 | 0.05362776 |
| 0.09665428 | 0.18762475 | 0.04072398 | 0.21911422 | 0.23343849 |
| 0.18959108 | 0.14171657 | 0.09502262 | 0.42191142 | 0.11356467 |
| 0.14869888 | 0.15369261 | 0.03167421 | 0.25874126 | 0.10410095 |
| 0.11524164 | 0.11776447 | 0.07239819 | 0.16550117 | 0.03785489 |
| 0.03345725 | 0.09780439 | 0.33936652 | 0.08158508 | 0.15457413 |
| 0 | 0.37125749 | 0.08144796 | 0.1958042 | 0.10410095 |

| 0.05576208 | 0.14371257 | 0.2081448 | 0.18881119 | 0.05362776 |
| --- | --- | --- | --- | --- |
| 0.25650558 | 0.18762475 | 0.04524887 | 0.26340326 | 0.11041009 |
| 0.04460967 | 0.42714571 | 0 | 0.22144522 | 0.15141956 |
| 0.17472119 | 0.12175649 | 0.12217195 | 0.14219114 | 0.04100946 |
| 0.09665428 | 0.35728543 | 0.02714932 | 0.5967366 | 0.16088328 |
| 0.10780669 | 0.22355289 | 0.05429864 | 0.21445221 | 0.03785489 |
| 0.0669145 | 0.16966068 | 0.04072398 | 0.21678322 | 0.09148265 |
| 0.04832714 | 0.11576846 | 0.04072398 | 0.10722611 | 0.17665615 |
| 0.04460967 | 0.21756487 | 0 | 0.22610723 | 0.07570978 |
| 0.01486989 | 0.19361277 | 0.04072398 | 0.27972028 | 0.31861199 |
| 0.18959108 | 0.12774451 | 0.0678733 | 0.35664336 | 0.05678233 |
| 0.30111524 | 0.10778443 | 0.04072398 | 0.31235431 | 0.0851735 |
| 0.07063197 | 0.27345309 | 0.01809955 | 0.1981352 | 0.06624606 |
| 0.04832714 | 0.30139721 | 0 | 0.24475524 | 0.24921136 |
| 0.05947955 | 0.14570858 | 0.07239819 | 0.24475524 | 0.06624606 |
| 0.07434944 | 0.11976048 | 0.22624434 | 0.09324009 | 0.12618297 |
| 0.07063197 | 0.17365269 | 0.04524887 | 0.11188811 | 0.05993691 |
| 0.08921933 | 0.18363273 | 0.05429864 | 0.26107226 | 0.05047319 |
| 0.39776952 | 0.0998004 | 0.09502262 | 0.2004662 | 0.11356467 |
| 0.28996283 | 0.24351297 | 0.01357466 | 0.33333333 | 0.04416404 |
| 0.09665428 | 0.1237525 | 0.04524887 | 0.18181818 | 0.14826498 |
| 0.43866171 | 0.36526946 | 0 | 0.31701632 | 0.46687697 |
| 0.03717472 | 0.20958084 | 0.02262443 | 0.17715618 | 0.07570978 |
| 0.07806691 | 0.23952096 | 0.04977376 | 0.31235431 | 0.12618297 |
| 0 | 0.30139721 | 0 | 0.29370629 | 0.17981073 |
| 0.03345725 | 0.1756487 | 0.02714932 | 0.18414918 | 0.20820189 |
| 0.14498141 | 0.33532934 | 0.02714932 | 0.55710956 | 0.10725552 |
| 0.31598513 | 0.14570858 | 0.13574661 | 0.15384615 | 0.15141956 |
| 0.14126394 | 0.25548902 | 0.13122172 | 0.27505828 | 0.17350158 |
| 0.05204461 | 0.28742515 | 0.04072398 | 0.27039627 | 0.21766562 |
| 0.05947955 | 0.15369261 | 0.05882353 | 0.13519814 | 0.12302839 |

| 0.23420074 | 0.15568862 | 0.0361991 | 0.28205128 | 0.0851735 |
| --- | --- | --- | --- | --- |
| 0.24907063 | 0.16167665 | 0.14027149 | 0.18881119 | 0.0977918 |
| 0.54275093 | 0.16167665 | 0.15384615 | 0.43589744 | 0.0977918 |
| 0.0669145 | 0.23153693 | 0 | 0.17016317 | 0.17350158 |
| 0 | 0.20758483 | 0 | 0.15151515 | 0.20504732 |
| 0.6394052 | 0.17165669 | 0 | 0.4009324 | 0.13564669 |
| 0.06319703 | 0.18762475 | 0.27149321 | 0.25874126 | 0.16088328 |
| 0 | 0.11776447 | 0.07692308 | 0 | 0.13249211 |
| 0.14498141 | 0.23353293 | 0.04072398 | 0.27272727 | 0.08201893 |
| 0.0260223 | 0.14371257 | 0.01357466 | 0.25641026 | 0.20504732 |
| 0 | 0.02994012 | 0.0678733 | 0.06759907 | 0.18611987 |
| 0.10037175 | 0.25948104 | 0.0361991 | 0.25874126 | 0.170347 |
| 0.30855019 | 0.04790419 | 0.16289593 | 0.19347319 | 0 |
| 0.16728625 | 0.4011976 | 0.12217195 | 0.20745921 | 0.11356467 |
| 0.03345725 | 0.20758483 | 0.06334842 | 0.10955711 | 0.25236593 |
| 0.20446097 | 0.31736527 | 0.09049774 | 0.25407925 | 0.12618297 |
| 0.16728625 | 0.1996008 | 0.13574661 | 0.16550117 | 0.19873817 |
| 0.08921933 | 0.27345309 | 0.07239819 | 0.20745921 | 0.20504732 |
| 0.15241636 | 0.16566866 | 0.0678733 | 0.3006993 | 0.08201893 |
| 0.03345725 | 0.05189621 | 0.15837104 | 0.06060606 | 0.02839117 |
| 0.04460967 | 0.15968064 | 0.05882353 | 0.07925408 | 0.30599369 |
| 0.08550186 | 0.20758483 | 0 | 0.13519814 | 0.21766562 |
| 0.04832714 | 0.21357285 | 0.02714932 | 0.24941725 | 0.3533123 |
| 0.59479554 | 0.46506986 | 0.29411765 | 0.49184149 | 0.23028391 |
| 0.08921933 | 0.14371257 | 0 | 0.11188811 | 0.12618297 |
| 0.20817844 | 0.249501 | 0.22171946 | 0.23076923 | 0.21766562 |
| 0.0260223 | 0.22155689 | 0.0678733 | 0.0955711 | 0.11671924 |
| 0.27881041 | 0.15568862 | 0.04524887 | 0.25641026 | 0.05047319 |
| 0.0260223 | 0.31936128 | 0.06334842 | 0.30769231 | 0.11041009 |
| 0.0929368 | 0.25149701 | 0.0361991 | 0.18181818 | 0.17665615 |
| 0.05947955 | 0.21956088 | 0.07239819 | 0.22144522 | 0.28706625 |

| 0.18215613 | 0.27145709 | 0.02714932 | 0.18648019 | 0.13564669 |
| --- | --- | --- | --- | --- |
| 0 | 0.23752495 | 0 | 0.06993007 | 0 |
| 0.13011152 | 0.249501 | 0.04977376 | 0.26107226 | 0.0851735 |
| 0.20817844 | 0.26147705 | 0 | 0.17482517 | 0 |
| 0.1598513 | 0.14171657 | 0 | 0.2004662 | 0.13564669 |
| 0.10780669 | 0.16966068 | 0.11764706 | 0.13752914 | 0.10094637 |
| 0.11524164 | 0.2754491 | 0.05882353 | 0.16783217 | 0.10410095 |
| 0.06319703 | 0.1756487 | 0.01809955 | 0.05827506 | 0.15772871 |
| 0.04460967 | 0.10179641 | 0.05429864 | 0.08624709 | 0.03785489 |
| 0 | 0.15768463 | 0 | 0.13752914 | 0.04731861 |
| 0.0260223 | 0.19361277 | 0.04977376 | 0.26107226 | 0.07886435 |
| 0.05576208 | 0.13173653 | 0.08597285 | 0.16083916 | 0.12618297 |
| 0.03717472 | 0.26147705 | 0.08597285 | 0.20512821 | 0.06309148 |
| 0.0929368 | 0.20159681 | 0.05882353 | 0.14685315 | 0.05993691 |
| 0.11152416 | 0.16566866 | 0.1040724 | 0.26573427 | 0.14511041 |
| 0.05576208 | 0.21756487 | 0.0678733 | 0.21911422 | 0.19242902 |
| 0.0260223 | 0.37325349 | 0 | 0.31468531 | 0.09463722 |
| 0.15613383 | 0.20159681 | 0 | 0.13752914 | 0.07886435 |
| 0.13754647 | 0.15768463 | 0.02262443 | 0.1958042 | 0.06624606 |
| 0.21561338 | 0.30938124 | 0.26244344 | 0.31468531 | 0.09148265 |
| 0.03345725 | 0.1996008 | 0.01357466 | 0.08158508 | 0.170347 |
| 0.08178439 | 0.16766467 | 0.04977376 | 0.11888112 | 0.01892744 |
| 0.12267658 | 0.13972056 | 0.14479638 | 0.24475524 | 0.06940063 |
| 0.18587361 | 0.16367265 | 0.03167421 | 0.24708625 | 0.09148265 |
| 0.08550186 | 0.09780439 | 0.19457014 | 0.13986014 | 0.09148265 |
| 0.0669145 | 0.2255489 | 0.02714932 | 0.2004662 | 0.10094637 |
| 0.28624535 | 0.14371257 | 0.09502262 | 0.26340326 | 0.12933754 |
| 0.19702602 | 0.20758483 | 0.1040724 | 0.22377622 | 0.11041009 |
| 0.0669145 | 0.12974052 | 0.05429864 | 0.21911422 | 0.12933754 |
| 0.17100372 | 0.26746507 | 0.04977376 | 0.15850816 | 0.07255521 |
| 0.04089219 | 0.28942116 | 0.02714932 | 0.32634033 | 0.08832808 |

| 0.23048327 | 0.20159681 | 0.14027149 | 0.10955711 | 0.17350158 |
| --- | --- | --- | --- | --- |
| 0.03345725 | 0.21556886 | 0.08144796 | 0.29370629 | 0.0851735 |
| 0.10037175 | 0.12974052 | 0.03167421 | 0.2004662 | 0.12618297 |
| 0.11895911 | 0.24151697 | 0.03167421 | 0.16083916 | 0.05362776 |
| 0 | 0.21357285 | 0.03167421 | 0.15617716 | 0.14826498 |
| 0 | 0.42914172 | 0 | 0.33333333 | 0 |
| 0.0929368 | 0.17365269 | 0.07239819 | 0.21445221 | 0.15457413 |
| 0.07806691 | 0.12774451 | 0.05882353 | 0.13752914 | 0.11356467 |
| 0.14126394 | 0.20758483 | 0.04072398 | 0.24242424 | 0.0977918 |
| 0.18215613 | 0.19560878 | 0.14479638 | 0.38461538 | 0.13249211 |
| 0.14869888 | 0.12974052 | 0.00904977 | 0.24475524 | 0.05678233 |
| 0.2267658 | 0.249501 | 0.02714932 | 0.23076923 | 0.11987382 |
| 0.01858736 | 0.21556886 | 0.02262443 | 0.11888112 | 0.04731861 |
| 0.19330855 | 0.16966068 | 0.01357466 | 0.28671329 | 0.11041009 |
| 0.03717472 | 0.25948104 | 0.0678733 | 0.17482517 | 0.03154574 |
| 0.32342007 | 0.13173653 | 0.06334842 | 0.25407925 | 0.07570978 |
| 0.03717472 | 0.20359281 | 0.01357466 | 0.26107226 | 0.09148265 |
| 0.27881041 | 0.23153693 | 0.04977376 | 0.31701632 | 0.10410095 |
| 0.09665428 | 0.15169661 | 0.05429864 | 0.14918415 | 0.13880126 |
| 0.18215613 | 0.34331337 | 0.05429864 | 0.17249417 | 0.38801262 |
| 0.3197026 | 0.14371257 | 0 | 0.2004662 | 0.22712934 |
| 0.1598513 | 0.29540918 | 0.05882353 | 0.2960373 | 0.10410095 |
| 0.24163569 | 0.15768463 | 0.02262443 | 0.14452214 | 0.13564669 |
| 0.19702602 | 0.249501 | 0.11764706 | 0.22843823 | 0.14511041 |
| 0.06319703 | 0.14770459 | 0.08597285 | 0.35198135 | 0.12933754 |
| 0 | 0.18962076 | 0.1040724 | 0.23076923 | 0.0851735 |
| 0.27137546 | 0.19560878 | 0.01357466 | 0.13986014 | 0.10410095 |
| 0.1598513 | 0.15568862 | 0.05429864 | 0.35431235 | 0.06624606 |
| 0.0929368 | 0.1996008 | 0.05429864 | 0.48018648 | 0.07886435 |
| 0.13754647 | 0.20359281 | 0.01357466 | 0.17948718 | 0.05993691 |
| 0.17472119 | 0.08582834 | 0 | 0.17482517 | 0.02523659 |

| 0.10408922 | 0.26746507 | 0.02714932 | 0.13986014 | 0.20820189 |
| --- | --- | --- | --- | --- |
| 0.24535316 | 0.24750499 | 0.19909502 | 0.14452214 | 0.0126183 |
| 0.21561338 | 0.09580838 | 0.07692308 | 0.23310023 | 0.11671924 |
| 0.04460967 | 0.05788423 | 0.19909502 | 0.19347319 | 0.07570978 |
| 0.21561338 | 0.13173653 | 0.07239819 | 0.24009324 | 0.04100946 |
| 0.1598513 | 0.16367265 | 0.04072398 | 0.33333333 | 0.17981073 |
| 0.05947955 | 0.21956088 | 0.15384615 | 0.23543124 | 0.12933754 |
| 0.0669145 | 0.10379242 | 0 | 0.17948718 | 0.08201893 |
| 0.12639405 | 0.18163673 | 0 | 0.19347319 | 0.09148265 |
| 0.21561338 | 0.20758483 | 0.08597285 | 0.51282051 | 0.09463722 |
| 0.10408922 | 0.16566866 | 0.07692308 | 0.20745921 | 0.07255521 |
| 0.21933086 | 0.25349301 | 0.0361991 | 0.25407925 | 0.09463722 |
| 0.33457249 | 0.12974052 | 0.02262443 | 0.33566434 | 0.05362776 |
| 0.15241636 | 0.18762475 | 0.13122172 | 0.13286713 | 0.15457413 |
| 0.17843866 | 0.18163673 | 0.04977376 | 0.2004662 | 0.05047319 |
| 0.10037175 | 0.1237525 | 0.05882353 | 0.16550117 | 0.11671924 |
| 0.08178439 | 0.12774451 | 0.05429864 | 0.1981352 | 0.05993691 |
| 0.08550186 | 0.26347305 | 0.03167421 | 0.22843823 | 0.17665615 |
| 0.34572491 | 0.24351297 | 0.07239819 | 0.44755245 | 0.07255521 |
| 0.05204461 | 0.1497006 | 0.06334842 | 0.15850816 | 0.25867508 |
| 0 | 0.07185629 | 0 | 0.12587413 | 0.11356467 |
| 0.01115242 | 0.18562874 | 0 | 0.13519814 | 0.14826498 |
| 0.12267658 | 0.17365269 | 0.08597285 | 0.12121212 | 0.13880126 |
| 0.11524164 | 0.09181637 | 0 | 0.14452214 | 0.04731861 |
| 0 | 0.05788423 | 0 | 0.2027972 | 0 |
| 0 | 0.4491018 | 0 | 0.1981352 | 0.2681388 |
| 0.10037175 | 0.09381238 | 0.03167421 | 0.17249417 | 0.12618297 |
| 0.02230483 | 0.34331337 | 0.07692308 | 0.39393939 | 0.21135647 |
| 0.18587361 | 0.249501 | 0 | 0.37762238 | 0 |
| 0.08921933 | 0.18762475 | 0.0361991 | 0.21911422 | 0.11041009 |
| 0.05204461 | 0.1237525 | 0.02262443 | 0.13752914 | 0.03785489 |

| 0.0260223 | 0.17165669 | 0.06334842 | 0.1002331 | 0.04416404 |
| --- | --- | --- | --- | --- |
| 0.02973978 | 0.39321357 | 0.11312217 | 0.36363636 | 0.15457413 |
| 0.56505576 | 0.15169661 | 0.11312217 | 0.23543124 | 0.07886435 |
| 0.12639405 | 0.28343313 | 0.07692308 | 0.21212121 | 0.08832808 |
| 0.04460967 | 0.15568862 | 0.04072398 | 0.3006993 | 0.170347 |
| 0.07063197 | 0.0758483 | 0 | 0.13286713 | 0 |
| 0.07063197 | 0.07784431 | 0.02714932 | 0.1048951 | 0.04100946 |
| 0.12639405 | 0.13572854 | 0.09502262 | 0.24009324 | 0.12933754 |
| 0.03345725 | 0.15369261 | 0 | 0.03962704 | 0.13564669 |
| 0 | 0.38123752 | 0.04977376 | 0.81118881 | 0.17665615 |
| 0 | 0.13373253 | 0 | 0.0955711 | 0.04731861 |
| 0.23048327 | 0.14570858 | 0.07692308 | 0.20979021 | 0.08832808 |
| 0.05947955 | 0.10978044 | 0 | 0.14685315 | 0.12302839 |
| 0 | 0.30938124 | 0 | 0.04195804 | 0.34700315 |
| 0 | 0.28143713 | 0.17647059 | 0.2027972 | 0.11356467 |
| 0.05576208 | 0.12175649 | 0.0678733 | 0.16083916 | 0.26498423 |
| 0.03345725 | 0.14371257 | 0.0361991 | 0.0979021 | 0.07570978 |
| 0.13754647 | 0.34730539 | 0.24886878 | 0.34265734 | 0.10094637 |
| 0.08921933 | 0.20359281 | 0 | 0.28438228 | 0.06309148 |
| 0.12639405 | 0.39520958 | 0.11312217 | 0.36363636 | 0.11356467 |
| 0.04832714 | 0.11576846 | 0.14479638 | 0.14219114 | 0.09148265 |
| 0.10408922 | 0.12175649 | 0.02262443 | 0.26107226 | 0.08832808 |
| 0.01858736 | 0.20958084 | 0.02262443 | 0.10955711 | 0.06624606 |
| 0.18215613 | 0.13772455 | 0.0361991 | 0.13286713 | 0.14195584 |
| 0.05204461 | 0.33932136 | 0.15837104 | 0.12121212 | 0.18611987 |
| 0.1598513 | 0.11976048 | 0.04072398 | 0.1002331 | 0.08201893 |
| 0.07806691 | 0.11776447 | 0 | 0.1002331 | 0.03470032 |
| 0.16356877 | 0.18163673 | 0.04072398 | 0.35198135 | 0.13249211 |
| 0.02973978 | 0.21157685 | 0.07692308 | 0.0979021 | 0.11987382 |
| 0.05204461 | 0.23552894 | 0.04072398 | 0.20745921 | 0.05993691 |
| 0.03345725 | 0.15968064 | 0.02262443 | 0.16550117 | 0.08832808 |

| 0 | 0.1257485 | 0.04072398 | 0.14685315 | 0.05678233 |
| --- | --- | --- | --- | --- |
| 0.05947955 | 0.31536926 | 0 | 0.12587413 | 0.12933754 |
| 0.02230483 | 0.13772455 | 0.02714932 | 0.07692308 | 0.01892744 |
| 0.16728625 | 0.29740519 | 0.02262443 | 0.20745921 | 0.12618297 |
| 0.08921933 | 0.19560878 | 0.06334842 | 0.27738928 | 0.04416404 |
| 0.05576208 | 0.17964072 | 0.03167421 | 0.40792541 | 0.16403785 |
| 0.17472119 | 0.26147705 | 0.07239819 | 0.21911422 | 0.03154574 |
| 0.1598513 | 0.19161677 | 0.19457014 | 0.17482517 | 0.13564669 |
| 0 | 0.31736527 | 0 | 0.05361305 | 0.07255521 |
| 0.11895911 | 0.13772455 | 0.04072398 | 0.24708625 | 0.02839117 |
| 0.07806691 | 0.17165669 | 0.02714932 | 0.18881119 | 0.08201893 |
| 0.03717472 | 0.16367265 | 0 | 0.1002331 | 0.04416404 |
| 0.02230483 | 0.10578842 | 0.02262443 | 0.15850816 | 0.09148265 |
| 0.04089219 | 0.24151697 | 0 | 0.26573427 | 0.11671924 |
| 0.32342007 | 0.10778443 | 0 | 0.29137529 | 0.05047319 |
| 0.21189591 | 0.29540918 | 0.08597285 | 0.27272727 | 0.17665615 |
| 0.05204461 | 0.1237525 | 0 | 0.06293706 | 0.04416404 |
| 0.09665428 | 0.34730539 | 0.08597285 | 0.16550117 | 0.1829653 |
| 0.05204461 | 0.25548902 | 0.05882353 | 0.20979021 | 0.15772871 |
| 0.02230483 | 0.19361277 | 0.02714932 | 0.22610723 | 0.10094637 |
| 0.03345725 | 0.1497006 | 0.04072398 | 0.20745921 | 0.22397476 |
| 0.1598513 | 0.10778443 | 0.07239819 | 0.26340326 | 0.03470032 |
| 0.0260223 | 0.14371257 | 0.05882353 | 0.1981352 | 0.04100946 |
| 0.42750929 | 0.21357285 | 0.09954751 | 0.25874126 | 0.04731861 |
| 0.05576208 | 0.2754491 | 0.0678733 | 0.32167832 | 0.21766562 |
| 0 | 0.13972056 | 0 | 0.18181818 | 0.12302839 |
| 0.06319703 | 0.10778443 | 0.04977376 | 0.24941725 | 0.03470032 |
| 0.12639405 | 0.13772455 | 0.04977376 | 0.12121212 | 0.09148265 |
| 0.14498141 | 0.18562874 | 0.0678733 | 0.28671329 | 0.10094637 |
| 0.07806691 | 0.1257485 | 0 | 0.12121212 | 0.03154574 |
| 0.08178439 | 0.2754491 | 0.04072398 | 0.24941725 | 0.14195584 |

| 0.06319703 | 0.13772455 | 0.00904977 | 0.16550117 | 0.09148265 |
| --- | --- | --- | --- | --- |
| 0 | 0.3992016 | 0.13122172 | 0.16550117 | 0 |
| 0.02973978 | 0.30139721 | 0.22624434 | 0.31468531 | 0.10725552 |
| 0 | 0.23353293 | 0.04977376 | 0.14918415 | 0.33438486 |
| 0.0929368 | 0.15768463 | 0 | 0.08857809 | 0.11041009 |
| 0.05204461 | 0.2255489 | 0.06334842 | 0.3962704 | 0.04416404 |
| 0.16356877 | 0.15369261 | 0 | 0.38228438 | 0.13880126 |
| 0.27509294 | 0.11976048 | 0.04072398 | 0.19347319 | 0.16088328 |
| 0.07806691 | 0.14570858 | 0.01809955 | 0.23310023 | 0.16088328 |
| 0.07806691 | 0.42315369 | 0 | 0.0979021 | 0.10094637 |
| 0.07063197 | 0.1756487 | 0.06334842 | 0.22843823 | 0.10410095 |
| 0.14126394 | 0.14171657 | 0 | 0.13986014 | 0.11987382 |
| 0 | 0.15169661 | 0 | 0.04428904 | 0.10094637 |
| 0.04460967 | 0.1237525 | 0 | 0.08624709 | 0.03785489 |
| 0.03345725 | 0.15768463 | 0.04072398 | 0.06526807 | 0.07255521 |
| 0 | 0.10578842 | 0.02262443 | 0.1025641 | 0.03154574 |
| 0.03345725 | 0.09381238 | 0 | 0.14219114 | 0.10410095 |
| 0.16356877 | 0.19361277 | 0.0678733 | 0.14918415 | 0.11356467 |
| 0.07063197 | 0.19760479 | 0.1040724 | 0.13519814 | 0.07255521 |
| 0.15613383 | 0.10179641 | 0.0361991 | 0.26573427 | 0.06624606 |
| 0.04089219 | 0.17964072 | 0.04072398 | 0.20512821 | 0.11987382 |
| 0.10408922 | 0.08383234 | 0 | 0.1958042 | 0.22082019 |
| 0.14869888 | 0.2255489 | 0.0361991 | 0.26340326 | 0.02523659 |
| 0.40520446 | 0.24750499 | 0.19909502 | 0.32167832 | 0.04731861 |
| 0.04089219 | 0.22754491 | 0 | 0.31934732 | 0 |
| 0.2267658 | 0.30738523 | 0.2760181 | 0.21445221 | 0.19242902 |
| 0.40520446 | 0.13373253 | 0.08144796 | 0.13986014 | 0.02208202 |
| 0.14869888 | 0.18363273 | 0.07692308 | 0.2004662 | 0.07255521 |
| 0.11152416 | 0.24550898 | 0.03167421 | 0.27972028 | 0.16719243 |
| 0.33828996 | 0.13972056 | 0.06334842 | 0.0979021 | 0.04416404 |
| 0.133829 | 0.12175649 | 0.10859729 | 0.31002331 | 0.03785489 |

| 0.10037175 | 0.30139721 | 0.04072398 | 0.18648019 | 0.02839117 |
| --- | --- | --- | --- | --- |
| 0.17472119 | 0.15169661 | 0.1719457 | 0.3962704 | 0.05993691 |
| 0.01858736 | 0.0998004 | 0.04524887 | 0.05827506 | 0.11041009 |
| 0.10037175 | 0.16167665 | 0 | 0.22144522 | 0.0851735 |
| 0.05204461 | 0.1756487 | 0.0361991 | 0.14452214 | 0.14511041 |
| 0 | 0.09381238 | 0 | 0.1981352 | 0.05993691 |
| 0.46840149 | 0.18363273 | 0.04977376 | 0.4009324 | 0 |
| 0.22304833 | 0.11976048 | 0 | 0.11655012 | 0.15772871 |
| 0.08921933 | 0.11776447 | 0.05429864 | 0.41258741 | 0.03785489 |
| 0.14498141 | 0.23153693 | 0.09502262 | 0.3006993 | 0.07255521 |
| 0.05204461 | 0.26746507 | 0.04072398 | 0.22144522 | 0.10094637 |
| 0.10780669 | 0.18163673 | 0.0361991 | 0.32167832 | 0.11987382 |
| 0.18587361 | 0.32335329 | 0.08597285 | 0.27505828 | 0.15772871 |
| 0 | 0.09780439 | 0.04524887 | 0.20512821 | 0.18611987 |
| 0.02973978 | 0.30938124 | 0.01357466 | 0.28438228 | 0.17981073 |
| 0.133829 | 0.18163673 | 0.08144796 | 0.21212121 | 0.17350158 |
| 0.10780669 | 0.25149701 | 0.01809955 | 0.39160839 | 0.11041009 |
| 0.07806691 | 0.08183633 | 0 | 0.21678322 | 0.22712934 |
| 0.12267658 | 0.10379242 | 0.05882353 | 0.15151515 | 0.06309148 |
| 0.21933086 | 0.1996008 | 0 | 0.22144522 | 0.12933754 |
| 0.02230483 | 0.30139721 | 0.05429864 | 0.11188811 | 0.32176656 |
| 0.15241636 | 0.11576846 | 0.04524887 | 0.1002331 | 0.07255521 |
| 0.22304833 | 0.18962076 | 0 | 0.17948718 | 0.08201893 |
| 0.06319703 | 0.16966068 | 0.10859729 | 0.20745921 | 0.06309148 |
| 0 | 0.06586826 | 0 | 0.25874126 | 0.06940063 |
| 0.21189591 | 0.18962076 | 0.05882353 | 0.25174825 | 0.07886435 |
| 0 | 0.09780439 | 0.05429864 | 0.11421911 | 0.23028391 |
| 0.14126394 | 0.1996008 | 0.01357466 | 0.2983683 | 0.13564669 |
| 0 | 0.18363273 | 0 | 0.14219114 | 0 |
| 0.05947955 | 0.14570858 | 0 | 0.21445221 | 0.1955836 |
| 0.14126394 | 0.11976048 | 0.04072398 | 0.23076923 | 0.12302839 |

| 0.11524164 | 0.16966068 | 0.08597285 | 0.26806527 | 0.14195584 |
| --- | --- | --- | --- | --- |
| 0.2267658 | 0.32335329 | 0.04977376 | 0.24708625 | 0.05362776 |
| 0.18959108 | 0.17964072 | 0.02262443 | 0.14219114 | 0.06624606 |
| 0.21933086 | 0.35129741 | 0.04524887 | 0.44289044 | 0.18611987 |
| 0.2267658 | 0.32135729 | 0.02714932 | 0.28438228 | 0.19242902 |
| 0.02973978 | 0.19760479 | 0.01809955 | 0.14452214 | 0.03785489 |
| 0.08178439 | 0.19161677 | 0 | 0.1981352 | 0.0977918 |
| 0.13754647 | 0.09381238 | 0.04072398 | 0.06526807 | 0.05993691 |
| 0.08178439 | 0.24351297 | 0.02714932 | 0.20745921 | 0.12302839 |
| 0.07063197 | 0.30139721 | 0.01357466 | 0.15617716 | 0.11041009 |
| 0.11895911 | 0.11177645 | 0.14479638 | 0.25874126 | 0.02523659 |
| 0.14869888 | 0.23752495 | 0.05882353 | 0.11421911 | 0.11041009 |
| 0.20074349 | 0.10778443 | 0 | 0.2983683 | 0 |
| 0.05204461 | 0.11377246 | 0.04977376 | 0.17249417 | 0.17350158 |
| 0.02973978 | 0.2255489 | 0.10859729 | 0.32867133 | 0.12618297 |
| 0.07434944 | 0.10179641 | 0 | 0.26340326 | 0 |
| 0.31226766 | 0.27345309 | 0.1040724 | 0.35431235 | 0.04731861 |
| 0.10408922 | 0.35528942 | 0 | 0.21911422 | 0.02839117 |
| 0.11152416 | 0.18562874 | 0.09954751 | 0.17249417 | 0.14826498 |
| 0.26022305 | 0.1257485 | 0.09049774 | 0.32167832 | 0.11356467 |
| 0.08550186 | 0.23952096 | 0.04977376 | 0.17715618 | 0.09148265 |
| 0 | 0.2754491 | 0 | 0 | 0 |
| 0.22304833 | 0.17964072 | 0 | 0.31235431 | 0.23659306 |
| 0 | 0.19760479 | 0.04524887 | 0.17016317 | 0.06624606 |
| 0.03345725 | 0.12774451 | 0 | 0.10722611 | 0.08832808 |
| 0.11152416 | 0.13972056 | 0.13574661 | 0.11655012 | 0 |
| 0.15613383 | 0.249501 | 0.04524887 | 0.17016317 | 0.03154574 |
| 0 | 0.48103792 | 0 | 0.18648019 | 0.25236593 |
| 0.05204461 | 0.57085828 | 0 | 0.19114219 | 0 |
| 0 | 0.19760479 | 0.01809955 | 0.07459207 | 0.14826498 |
| 0.34572491 | 0.18562874 | 0 | 0.54545455 | 0 |

| 0.05204461 | 0.12774451 | 0.04524887 | 0.22144522 | 0.07886435 |
| --- | --- | --- | --- | --- |
| 0.08550186 | 0.13572854 | 0.1719457 | 0.15850816 | 0.07255521 |
| 0.30855019 | 0.31337325 | 0.0361991 | 0.25174825 | 0.23659306 |
| 0.133829 | 0.12974052 | 0 | 0.16550117 | 0.07570978 |
| 0.05204461 | 0.13972056 | 0.12669683 | 0.1958042 | 0.08832808 |
| 0.02973978 | 0.10578842 | 0.0361991 | 0.15850816 | 0.14195584 |
| 0.16356877 | 0.10179641 | 0.03167421 | 0.25407925 | 0.06940063 |
| 0.25650558 | 0.13772455 | 0 | 0.16083916 | 0.21766562 |
| 0.26022305 | 0.18762475 | 0.01357466 | 0.13519814 | 0.2681388 |
| 0.09665428 | 0.22155689 | 0.08144796 | 0.33100233 | 0.09148265 |
| 0.12639405 | 0.11576846 | 0.02714932 | 0.2004662 | 0.06940063 |
| 0.0929368 | 0.1237525 | 0.05429864 | 0.17948718 | 0.05993691 |
| 0.20817844 | 0.0758483 | 0.01357466 | 0.20979021 | 0.07570978 |
| 0.0260223 | 0.0998004 | 0.06334842 | 0.13286713 | 0.08832808 |
| 0.08550186 | 0.13572854 | 0.0361991 | 0.23076923 | 0.04731861 |
| 0 | 0.11776447 | 0 | 0.13752914 | 0 |
| 0.02230483 | 0.1497006 | 0.19909502 | 0.07226107 | 0.13880126 |
| 0 | 0.16766467 | 0 | 0.44522145 | 0.11356467 |
| 0.17472119 | 0.16566866 | 0.21266968 | 0.27505828 | 0.07570978 |
| 0.0260223 | 0.17365269 | 0 | 0.16783217 | 0.09148265 |
| 0.02230483 | 0.32135729 | 0.0678733 | 0.27039627 | 0.10725552 |
| 0.18587361 | 0.11976048 | 0.04524887 | 0.23310023 | 0.18927445 |
| 0.08550186 | 0.15169661 | 0.01357466 | 0.12820513 | 0.0977918 |
| 0.16356877 | 0.08383234 | 0.00904977 | 0.16083916 | 0.12302839 |
| 0.01486989 | 0.06187625 | 0.04977376 | 0.08158508 | 0.06309148 |
| 0.05947955 | 0.14371257 | 0.02262443 | 0.13752914 | 0.12302839 |
| 0.06319703 | 0.21956088 | 0.12669683 | 0.1025641 | 0.08201893 |
| 0.20817844 | 0.26946108 | 0.01809955 | 0.35198135 | 0.21451104 |
| 0.19330855 | 0.12774451 | 0.07692308 | 0.21445221 | 0.07255521 |
| 0.03345725 | 0.23552894 | 0.07692308 | 0.19347319 | 0.0977918 |
| 0.03717472 | 0.12175649 | 0 | 0.07226107 | 0.06309148 |

| 0.30111524 | 0.07984032 | 0.36651584 | 0.37529138 | 0 |
| --- | --- | --- | --- | --- |
| 0.14126394 | 0.1756487 | 0.11312217 | 0.23310023 | 0.03785489 |
| 0.0260223 | 0.19760479 | 0.12217195 | 0.15617716 | 0.11041009 |
| 0 | 0.33133733 | 0.09502262 | 0.24242424 | 0.12933754 |
| 0 | 0.56886228 | 0 | 0.66433566 | 0.06309148 |
| 0.1598513 | 0.13173653 | 0.02262443 | 0.13286713 | 0.05993691 |
| 0.08178439 | 0.26946108 | 0 | 0.15617716 | 0.06940063 |
| 0.03717472 | 0.1237525 | 0.04524887 | 0.11888112 | 0.12933754 |
| 0.09665428 | 0.22754491 | 0.11764706 | 0.34498834 | 0.11041009 |
| 0.08550186 | 0.0758483 | 0.0361991 | 0.17016317 | 0.07886435 |
| 0.16356877 | 0.32135729 | 0 | 0.13519814 | 0.32176656 |
| 0.05576208 | 0.13772455 | 0.0678733 | 0.16083916 | 0.07255521 |
| 0.10408922 | 0.33333333 | 0.06334842 | 0.22843823 | 0.13249211 |
| 0.24907063 | 0.08982036 | 0.20361991 | 0 | 0.21135647 |
| 0 | 0.32135729 | 0 | 0.23310023 | 0.12618297 |
| 0.23048327 | 0.18962076 | 0.07239819 | 0.20512821 | 0.07255521 |
| 0.08550186 | 0.0998004 | 0.04977376 | 0.15384615 | 0.03785489 |
| 0.01115242 | 0.10179641 | 0.1040724 | 0.03962704 | 0.03785489 |
| 0.07434944 | 0.09780439 | 0 | 0.43123543 | 0.03154574 |
| 0.36431227 | 0.18363273 | 0.05429864 | 0.36130536 | 0.05362776 |
| 0.03717472 | 0.26147705 | 0 | 0.28205128 | 0.18927445 |
| 0.02973978 | 0.06586826 | 0.01357466 | 0.08857809 | 0.00946372 |
| 0 | 0.1996008 | 0 | 0.23310023 | 0 |
| 0.05204461 | 0.06986028 | 0.06334842 | 0.20979021 | 0.15457413 |
| 0.01858736 | 0.39121756 | 0.07239819 | 0.13519814 | 0.16719243 |
| 0.11524164 | 0.12974052 | 0.09049774 | 0.17482517 | 0.06309148 |
| 0.17472119 | 0.28343313 | 0 | 0.08158508 | 0.11041009 |
| 0 | 0.53892216 | 0 | 0.13986014 | 0.56782334 |
| 0.02973978 | 0.06187625 | 0 | 0.05361305 | 0.19242902 |
| 0.30483271 | 0.06586826 | 0.07239819 | 0.11421911 | 0.05047319 |
| 0.39405204 | 0.26546906 | 0.239819 | 0.31002331 | 0.0851735 |

| 0.14869888 | 0.09780439 | 0 | 0.24475524 | 0.10094637 |
| --- | --- | --- | --- | --- |
| 0.1598513 | 0.08582834 | 0.04072398 | 0.16083916 | 0.05362776 |
| 0.13011152 | 0.11576846 | 0.21266968 | 0.05361305 | 0.1829653 |
| 0 | 0.0998004 | 0 | 0.46620047 | 0.15772871 |
| 0.03345725 | 0 | 0.08144796 | 0.14219114 | 0.19242902 |
| 0.15241636 | 0.1756487 | 0 | 0.17715618 | 0.11041009 |
| 0.03717472 | 0.2754491 | 0.09049774 | 0.25407925 | 0.06309148 |
| 0.05947955 | 0.16367265 | 0 | 0.03729604 | 0 |
| 0.05204461 | 0.22155689 | 0.01357466 | 0.12820513 | 0.20820189 |
| 0 | 0.10179641 | 0.0678733 | 0.03496503 | 0.27760252 |
| 0.13011152 | 0.11177645 | 0.23529412 | 0.15384615 | 0.11987382 |
| 0.20817844 | 0.16766467 | 0 | 0.32634033 | 0.08832808 |
| 0.05576208 | 0.2255489 | 0.11764706 | 0.13286713 | 0.03154574 |
| 0 | 0.23752495 | 0 | 0.08857809 | 0.29652997 |
| 0.03717472 | 0.03992016 | 0 | 0.06993007 | 0.03154574 |
| 0.04089219 | 0.20558882 | 0.01357466 | 0.22610723 | 0.170347 |
| 0.11524164 | 0.25349301 | 0.16742081 | 0.43589744 | 0.17981073 |
| 0 | 0.2994012 | 0.04977376 | 0.1002331 | 0.13564669 |
| 0.05576208 | 0.08383234 | 0.01809955 | 0.12354312 | 0.02523659 |
| 0.18959108 | 0.15369261 | 0.05429864 | 0.25407925 | 0.15141956 |
| 0.33828996 | 0.15169661 | 0.05429864 | 0.41958042 | 0.11987382 |
| 0.03345725 | 0.19161677 | 0.19909502 | 0.2027972 | 0.30283912 |
| 0 | 0.13173653 | 0 | 0.30536131 | 0.41324921 |
| 0.03345725 | 0.20758483 | 0 | 0.28671329 | 0.11987382 |
| 0 | 0.11776447 | 0.0678733 | 0 | 0 |
| 0 | 0.15968064 | 0 | 0.31002331 | 0 |
| 0.10037175 | 0.05389222 | 0 | 0.18881119 | 0.170347 |
| 0.03345725 | 0.13772455 | 0.05882353 | 0.16083916 | 0.05362776 |
| 0.00371747 | 0.06586826 | 0.00452489 | 0.10955711 | 0.05047319 |
| 0.19702602 | 0.13572854 | 0.0678733 | 0.22610723 | 0.170347 |
| 0.11152416 | 0.29141717 | 0.02262443 | 0.36130536 | 0.05047319 |

| 0.25650558 | 0.26546906 | 0.09049774 | 0.35897436 | 0.19242902 |
| --- | --- | --- | --- | --- |
| 0.11152416 | 0.20958084 | 0.0678733 | 0.20979021 | 0 |
| 0.02973978 | 0.21956088 | 0.0361991 | 0.07925408 | 0.07886435 |
| 0 | 0.0239521 | 0.05429864 | 0.24708625 | 0.03785489 |
| 0.10780669 | 0.24550898 | 0 | 0.16783217 | 0.41009464 |
| 0.21189591 | 0.20359281 | 0.1040724 | 0.05361305 | 0.07255521 |
| 0.07063197 | 0.22355289 | 0.08597285 | 0.1958042 | 0.05993691 |
| 0.34944238 | 0.21556886 | 0 | 0.18881119 | 0.0851735 |
| 0.13754647 | 0.33133733 | 0 | 0.21445221 | 0.05678233 |
| 0.30111524 | 0.11576846 | 0.05429864 | 0.37529138 | 0.1829653 |
| 0 | 0 | 0 | 0.07459207 | 0.15141956 |
| 0.33457249 | 0.35928144 | 0 | 0.38461538 | 0 |
| 0 | 0.29740519 | 0 | 0.20745921 | 0 |
| 0.09665428 | 0.13972056 | 0 | 0.25407925 | 0.11987382 |
| 0.06319703 | 0.25149701 | 0 | 0.27505828 | 0.18611987 |
| 0 | 0.21556886 | 0.05882353 | 0.5967366 | 0.04100946 |
| 0.05576208 | 0.05988024 | 0 | 0.24708625 | 0.09463722 |
| 0.63568773 | 0.09381238 | 0.11312217 | 0.23776224 | 0.05678233 |
| 0.10780669 | 0.15169661 | 0.04524887 | 0.11188811 | 0.11987382 |
| 0.02230483 | 0.20958084 | 0.05882353 | 0.20512821 | 0.06940063 |
| 0.03717472 | 0.23552894 | 0 | 0.27039627 | 0.12933754 |
| 0.04460967 | 0.34331337 | 0 | 0.16550117 | 0.05678233 |
| 0.05204461 | 0.14570858 | 0.0361991 | 0.25641026 | 0.18611987 |
| 0.0929368 | 0.14770459 | 0 | 0.05827506 | 0.07886435 |
| 0.10037175 | 0.27145709 | 0 | 0.19114219 | 0.0851735 |
| 0 | 0.18962076 | 0 | 0.11188811 | 0 |
| 0.11895911 | 0.17165669 | 0 | 0.24941725 | 0.170347 |
| 0.03717472 | 0.15968064 | 0.05429864 | 0.13986014 | 0.13249211 |
| 0.05576208 | 0.21556886 | 0.32579186 | 0.10722611 | 0.34069401 |
| 0.05576208 | 0.04191617 | 0.01809955 | 0.06060606 | 0.08201893 |
| 0.02230483 | 0.13572854 | 0.05429864 | 0.18648019 | 0.13880126 |

| 0.10780669 | 0.25948104 | 0.01809955 | 0.17016317 | 0.11041009 |
| --- | --- | --- | --- | --- |
| 0.10408922 | 0.2255489 | 0.08144796 | 0.22377622 | 0.11671924 |
| 0 | 0.19361277 | 0.0361991 | 0.05594406 | 0.05047319 |
| 0.03345725 | 0.20159681 | 0.02714932 | 0.16550117 | 0.13564669 |
| 0.21189591 | 0.0998004 | 0.06334842 | 0.31468531 | 0.06624606 |
| 0.04460967 | 0.09181637 | 0.11764706 | 0.17016317 | 0.21135647 |
| 0.30111524 | 0.15568862 | 0.05882353 | 0.3986014 | 0.10725552 |
| 0.12267658 | 0.05988024 | 0.0361991 | 0.18881119 | 0.12302839 |
| 0.16728625 | 0.36726547 | 0.06334842 | 0.32634033 | 0.10725552 |
| 0.18587361 | 0.31337325 | 0.0361991 | 0.28904429 | 0.07886435 |
| 0.08921933 | 0.04790419 | 0.10859729 | 0.1958042 | 0.15141956 |
| 0.01115242 | 0.34530938 | 0.00904977 | 0.29370629 | 0.09463722 |
| 0.05204461 | 0.28143713 | 0.06334842 | 0.2960373 | 0.04416404 |
| 0.11524164 | 0.24351297 | 0 | 0.21445221 | 0.03154574 |
| 0 | 0.09780439 | 0 | 0.03729604 | 0.2555205 |
| 0.07063197 | 0.26946108 | 0 | 0.24009324 | 0.16088328 |
| 0 | 0 | 0 | 0.04195804 | 0.22712934 |
| 0.17100372 | 0.13772455 | 0 | 0.10722611 | 0 |
| 0 | 0.18363273 | 0.04072398 | 0.24475524 | 0.08201893 |
| 0.0669145 | 0.17764471 | 0 | 0.1048951 | 0.02839117 |
| 0.13754647 | 0.1257485 | 0.07692308 | 0.22377622 | 0.07255521 |
| 0 | 0.21756487 | 0 | 0.05128205 | 0.03470032 |
| 0.43866171 | 0.0998004 | 0.0361991 | 0.41258741 | 0.07886435 |
| 0.08178439 | 0.31137725 | 0.20361991 | 0.31235431 | 0.14195584 |
| 0.08550186 | 0.13772455 | 0 | 0.21678322 | 0.1829653 |
| 0.02973978 | 0.10978044 | 0.07239819 | 0.10955711 | 0 |
| 0.10408922 | 0.11177645 | 0.04072398 | 0.1958042 | 0.05993691 |
| 0.0669145 | 0.21357285 | 0.08144796 | 0.04195804 | 0 |
| 0.03717472 | 0.15968064 | 0.02262443 | 0.16317016 | 0.09463722 |
| 0.04460967 | 0.14770459 | 0 | 0.14219114 | 0.19242902 |
| 0.07063197 | 0.21357285 | 0.02714932 | 0.19114219 | 0.13880126 |

| 0 | 0.35528942 | 0 | 0.53146853 | 0.07886435 |
| --- | --- | --- | --- | --- |
| 0.26394052 | 0.18762475 | 0 | 0.10955711 | 0 |
| 0.05204461 | 0.18363273 | 0.12669683 | 0.14918415 | 0.11041009 |
| 0.17472119 | 0.16766467 | 0 | 0.15151515 | 0.13249211 |
| 0.03345725 | 0.15968064 | 0 | 0.22610723 | 0.1955836 |
| 0.06319703 | 0.16766467 | 0 | 0.23543124 | 0.21135647 |
| 0.30855019 | 0.1996008 | 0.07692308 | 0.11655012 | 0.05362776 |
| 0.07434944 | 0.11976048 | 0 | 0.32400932 | 0.12618297 |
| 0.17472119 | 0.11776447 | 0 | 0.13752914 | 0.03785489 |
| 0.0929368 | 0.20159681 | 0.07692308 | 0.0979021 | 0.10725552 |
| 0.2527881 | 0.18163673 | 0 | 0.37296037 | 0 |
| 0.11895911 | 0.15768463 | 0.14479638 | 0.18414918 | 0.02523659 |
| 0.10037175 | 0.08183633 | 0.06334842 | 0.0955711 | 0.04416404 |
| 0 | 0.10379242 | 0 | 0 | 0.16403785 |
| 0.18959108 | 0.16167665 | 0.01809955 | 0.18881119 | 0.05362776 |
| 0.11895911 | 0.22355289 | 0.0361991 | 0.07459207 | 0.17665615 |
| 0.18215613 | 0.09780439 | 0.05429864 | 0.2004662 | 0 |
| 0.14498141 | 0.23353293 | 0 | 0 | 0 |
| 0.08921933 | 0.30538922 | 0.21266968 | 0.38461538 | 0.22397476 |
| 0 | 0.19161677 | 0.14479638 | 0.18648019 | 0.15141956 |
| 0 | 0.32934132 | 0.13574661 | 0.31468531 | 0.14195584 |
| 0 | 0.1996008 | 0 | 0.15617716 | 0.05362776 |
| 0 | 0.35329341 | 0 | 0.44289044 | 0.72870662 |
| 0 | 0 | 0 | 0.11188811 | 0.22397476 |
| 0.05947955 | 0.17165669 | 0 | 0.1002331 | 0.13564669 |
| 0.05204461 | 0.19560878 | 0.03167421 | 0.0979021 | 0.06624606 |
| 0.0669145 | 0.24550898 | 0 | 0 | 0.11041009 |
| 0.01486989 | 0.24151697 | 0.01809955 | 0.21445221 | 0.11671924 |
| 0.14126394 | 0.08982036 | 0.14479638 | 0.35897436 | 0.01892744 |
| 0.01115242 | 0.13972056 | 0.02714932 | 0.16317016 | 0.07570978 |
| 0.08550186 | 0.21357285 | 0.00904977 | 0.15384615 | 0.13249211 |

| 0 | 0.08383234 | 0.09502262 | 0.04895105 | 0.13249211 |
| --- | --- | --- | --- | --- |
| 0.03345725 | 0.11776447 | 0.40271493 | 0.07226107 | 0.06940063 |
| 0.08178439 | 0.28942116 | 0.04977376 | 0.49417249 | 0.10410095 |
| 0.03345725 | 0.31736527 | 0.05882353 | 0.15151515 | 0.06940063 |
| 0.0260223 | 0.32135729 | 0 | 0.24475524 | 0.04416404 |
| 0.18587361 | 0.1756487 | 0.0361991 | 0.31002331 | 0.10725552 |
| 0.11895911 | 0.12974052 | 0.04977376 | 0.2004662 | 0 |
| 0.07063197 | 0.10778443 | 0.0361991 | 0.06293706 | 0.0851735 |
| 0.06319703 | 0.15369261 | 0.03167421 | 0.27738928 | 0.22082019 |
| 0 | 0.08782435 | 0.04977376 | 0.27972028 | 0.20820189 |
| 0.11895911 | 0.25548902 | 0.02262443 | 0.18881119 | 0.23343849 |
| 0 | 0.03393214 | 0.38461538 | 0.06060606 | 0.13564669 |
| 0.133829 | 0.14371257 | 0.08144796 | 0.33333333 | 0.12618297 |
| 0.05204461 | 0.16566866 | 0.0361991 | 0.23776224 | 0.170347 |
| 0.04460967 | 0.17365269 | 0.12217195 | 0.28205128 | 0.16403785 |
| 0.02230483 | 0.28143713 | 0 | 0.07226107 | 0.11671924 |
| 0.06319703 | 0.20558882 | 0.08144796 | 0.32167832 | 0.15141956 |
| 0.24907063 | 0.06586826 | 0 | 0.07692308 | 0 |
| 0.05947955 | 0.35129741 | 0.09502262 | 0.59207459 | 0.01577287 |
| 0.1598513 | 0.20359281 | 0.04977376 | 0.33333333 | 0.05047319 |
| 0 | 0.18562874 | 0 | 0.04895105 | 0.08832808 |
| 0.02230483 | 0.18962076 | 0.0361991 | 0.26806527 | 0.10094637 |
| 0 | 0.19560878 | 0 | 0.17016317 | 0.19242902 |
| 0.06319703 | 0.13373253 | 0 | 0.13752914 | 0.16088328 |
| 0.02230483 | 0.12774451 | 0.11312217 | 0.35897436 | 0.23974763 |
| 0.0260223 | 0.08383234 | 0.02714932 | 0.0979021 | 0.08201893 |
| 0.07434944 | 0.12175649 | 0.09049774 | 0.18881119 | 0.03154574 |
| 0.03345725 | 0.11377246 | 0.07692308 | 0.19347319 | 0.30599369 |
| 0.03717472 | 0.07984032 | 0 | 0.06993007 | 0.09463722 |
| 0.00743494 | 0.30139721 | 0.08597285 | 0.2983683 | 0.16088328 |
| 0.10780669 | 0.11377246 | 0.08144796 | 0.10722611 | 0.07886435 |

| 0.2527881 | 0 | 0.1040724 | 0.1048951 | 0.42902208 |
| --- | --- | --- | --- | --- |
| 0.14126394 | 0.16766467 | 0.21266968 | 0.17482517 | 0.13249211 |
| 0.133829 | 0.16167665 | 0 | 0.16783217 | 0.14195584 |
| 0.11152416 | 0.09780439 | 0.04524887 | 0.02331002 | 0.09463722 |
| 0.08178439 | 0.43512974 | 0 | 0.25407925 | 0.13880126 |
| 0 | 0.24550898 | 0.04072398 | 0.10955711 | 0.08832808 |
| 0 | 0.06187625 | 0 | 0.17948718 | 0.1955836 |
| 0.03345725 | 0.11377246 | 0 | 0.08857809 | 0 |
| 0.02230483 | 0.28143713 | 0.31674208 | 0.56643357 | 0.05993691 |
| 0.08550186 | 0.23353293 | 0.1040724 | 0.27272727 | 0.14826498 |
| 0.01486989 | 0.20758483 | 0.07239819 | 0.15850816 | 0.05047319 |
| 0.04460967 | 0.20359281 | 0 | 0.22377622 | 0.28391167 |
| 0.04460967 | 0.16367265 | 0.10859729 | 0.13752914 | 0.03785489 |
| 0.03345725 | 0.30339321 | 0.16289593 | 0.20745921 | 0.42271293 |
| 0.08178439 | 0.31936128 | 0.13122172 | 0.22144522 | 0.1829653 |
| 0.05947955 | 0.27944112 | 0 | 0.10955711 | 0.03785489 |
| 0 | 0.18562874 | 0.05429864 | 0.16317016 | 0.25867508 |
| 0.07063197 | 0.48902196 | 0.1719457 | 0.17482517 | 0 |
| 0.04460967 | 0.16566866 | 0 | 0.05594406 | 0.22397476 |
| 0.08921933 | 0.23952096 | 0.14479638 | 0.37062937 | 0.05047319 |
| 0.26394052 | 0.14171657 | 0 | 0.16550117 | 0 |
| 0.10780669 | 0.13572854 | 0.09954751 | 0.18881119 | 0.10094637 |
| 0 | 0.10778443 | 0 | 0.04195804 | 0.11356467 |
| 0.14126394 | 0.08982036 | 0.02714932 | 0.19347319 | 0.08201893 |
| 0 | 0.29341317 | 0 | 0.1981352 | 0.07570978 |
| 0.08550186 | 0.26546906 | 0.06334842 | 0.32867133 | 0.09148265 |
| 0.04089219 | 0.24750499 | 0 | 0.26340326 | 0 |
| 0.133829 | 0.21756487 | 0 | 0.17016317 | 0.05678233 |
| 0.04089219 | 0.25149701 | 0 | 0.15850816 | 0.14511041 |
| 0.04089219 | 0.15369261 | 0.04977376 | 0.28205128 | 0 |
| 0.04832714 | 0.0758483 | 0 | 0.29370629 | 0.07886435 |

| 0.13754647 | 0.14570858 | 0 | 0.08624709 | 0.07570978 |
| --- | --- | --- | --- | --- |
| 0.0669145 | 0.03592814 | 0 | 0.16783217 | 0.05678233 |
| 0 | 0.24151697 | 0.0678733 | 0.24708625 | 0.09463722 |
| 0.04089219 | 0.06387226 | 0 | 0.22610723 | 0.13564669 |
| 0.17100372 | 0.09181637 | 0.1040724 | 0.54079254 | 0.29337539 |
| 0.08921933 | 0.23752495 | 0.09954751 | 0.39160839 | 0.17665615 |
| 0.10037175 | 0.12774451 | 0 | 0.04195804 | 0.20189274 |
| 0.06319703 | 0.13972056 | 0 | 0.14219114 | 0.27444795 |
| 0.11895911 | 0.29540918 | 0.02714932 | 0.13519814 | 0.01892744 |
| 0.18959108 | 0.20558882 | 0 | 0.03962704 | 0 |
| 0 | 0.1257485 | 0 | 0.18181818 | 0 |
| 0.09665428 | 0.07784431 | 0 | 0.21212121 | 0.08201893 |
| 0.07434944 | 0.33333333 | 0.09049774 | 0.34032634 | 0.06309148 |
| 0.15241636 | 0.1756487 | 0.08144796 | 0.17948718 | 0.16719243 |
| 0.05204461 | 0.26746507 | 0 | 0.37762238 | 0.13249211 |
| 0.04832714 | 0.18962076 | 0.05882353 | 0.24941725 | 0.05993691 |
| 0.14498141 | 0.03792415 | 0 | 0.17948718 | 0.05993691 |
| 0 | 0.05988024 | 0 | 0.06993007 | 0 |
| 0 | 0.23153693 | 0 | 0.33100233 | 0.3659306 |
| 0.05204461 | 0.13173653 | 0.04072398 | 0.32167832 | 0.4637224 |
| 0 | 0.07784431 | 0 | 0.03030303 | 0.08201893 |
| 0 | 0.1497006 | 0.04072398 | 0.17482517 | 0.02839117 |
| 0.41635688 | 0.21556886 | 0.01809955 | 0.33566434 | 0.25236593 |
| 0 | 0.09181637 | 0 | 0.07226107 | 0.04731861 |
| 0 | 0.12175649 | 0.05429864 | 0.1981352 | 0.2681388 |
| 0.0929368 | 0.13173653 | 0.11312217 | 0.07692308 | 0.05362776 |
| 0.17472119 | 0.35528942 | 0.0361991 | 0.43356643 | 0.12302839 |
| 0.10780669 | 0.11377246 | 0.06334842 | 0.16783217 | 0.04416404 |
| 0.09665428 | 0.23752495 | 0 | 0.21445221 | 0.08201893 |
| 0.03717472 | 0.23153693 | 0.0678733 | 0.16550117 | 0.09463722 |
| 0.0929368 | 0.0998004 | 0 | 0.17249417 | 0 |

| 0.08921933 | 0.18762475 | 0 | 0.05594406 | 0.29652997 |
| --- | --- | --- | --- | --- |
| 0.10037175 | 0.16167665 | 0.09049774 | 0.20512821 | 0.09463722 |
| 0.2936803 | 0.11576846 | 0.08144796 | 0.27272727 | 0.11987382 |
| 0.10408922 | 0.21956088 | 0.01357466 | 0.17249417 | 0.23343849 |
| 0.08178439 | 0.04391218 | 0.03167421 | 0.13519814 | 0.1829653 |
| 0.31226766 | 0.18562874 | 0.0361991 | 0.26573427 | 0.18927445 |
| 0.03717472 | 0.0499002 | 0.03167421 | 0.07459207 | 0.17350158 |
| 0.02230483 | 0.13173653 | 0 | 0.1025641 | 0.10410095 |
| 0.18587361 | 0.25548902 | 0.08144796 | 0.2983683 | 0.10094637 |
| 0.11524164 | 0.18762475 | 0 | 0.14685315 | 0.05047319 |
| 0.07063197 | 0.21956088 | 0 | 0.32867133 | 0.09148265 |
| 0 | 0.2255489 | 0.04977376 | 0.25174825 | 0.15141956 |
| 0.19702602 | 0.16766467 | 0 | 0.05361305 | 0.07255521 |
| 0.05947955 | 0.25349301 | 0.02262443 | 0.24475524 | 0.15141956 |
| 0.01486989 | 0.30139721 | 0.04524887 | 0.24475524 | 0.05047319 |
| 0 | 0.28942116 | 0 | 0.15151515 | 0.06940063 |
| 0.14869888 | 0.18762475 | 0.18099548 | 0.18648019 | 0.0851735 |
| 0.13754647 | 0.17964072 | 0.08144796 | 0.25174825 | 0.12933754 |
| 0.11895911 | 0.30938124 | 0 | 0.28671329 | 0.20189274 |
| 0.19702602 | 0.21157685 | 0.04977376 | 0.24708625 | 0.07255521 |
| 0.01486989 | 0.26546906 | 0.02714932 | 0.19114219 | 0.04731861 |
| 0.11895911 | 0.32135729 | 0.02714932 | 0.33566434 | 0.19873817 |
| 0.04460967 | 0.31736527 | 0.01809955 | 0.24242424 | 0.14195584 |
| 0.18959108 | 0.22754491 | 0.01357466 | 0.20745921 | 0.10410095 |
| 0 | 0.10179641 | 0 | 0.05361305 | 0.22397476 |
| 0.05204461 | 0.05788423 | 0.16289593 | 0.2004662 | 0.17981073 |
| 0 | 0.09780439 | 0 | 0.11421911 | 0.15457413 |
| 0.04832714 | 0.15768463 | 0.04524887 | 0.17715618 | 0.17665615 |
| 0 | 0 | 0.06334842 | 0 | 0 |
| 0 | 0.24151697 | 0.04072398 | 0.02097902 | 0.16403785 |
| 0.02973978 | 0.29540918 | 0.1040724 | 0.14452214 | 0.22082019 |

| 0.00371747 | 0.18762475 | 0.0361991 | 0.21212121 | 0.13880126 |
| --- | --- | --- | --- | --- |
| 0.0929368 | 0.26546906 | 0.12669683 | 0.27039627 | 0.19873817 |
| 0 | 0.07984032 | 0 | 0.04662005 | 0 |
| 0 | 0.20558882 | 0.01357466 | 0.18181818 | 0.17665615 |
| 0.14498141 | 0.07784431 | 0 | 0.15151515 | 0.04100946 |
| 0.17100372 | 0.15169661 | 0 | 0.28438228 | 0.09463722 |
| 0.19702602 | 0.16566866 | 0.16289593 | 0.27505828 | 0.14826498 |
| 0.18215613 | 0.1237525 | 0.0678733 | 0.10955711 | 0.35015773 |
| 0 | 0.04391218 | 0 | 0.1048951 | 0.06940063 |
| 0 | 0.23552894 | 0.03167421 | 0.21445221 | 0.13880126 |
| 0.05947955 | 0.12774451 | 0.93665158 | 0.03729604 | 0.05047319 |
| 0.22304833 | 0.20958084 | 0.02262443 | 0.2983683 | 0.07255521 |
| 0.07063197 | 0.13173653 | 0.04072398 | 0.10955711 | 0.02839117 |
| 0.20817844 | 0.05588822 | 0 | 0.32634033 | 0 |
| 0 | 0.18762475 | 0 | 0.39393939 | 0.14826498 |
| 0.05576208 | 0.46906188 | 0.0678733 | 0.1025641 | 0.09148265 |
| 0.07806691 | 0.25548902 | 0.09502262 | 0.1002331 | 0.06624606 |
| 0.07434944 | 0.22355289 | 0.09049774 | 0.16550117 | 0.22397476 |
| 0.17472119 | 0.33133733 | 0.1719457 | 0.17948718 | 0.33753943 |
| 0.20446097 | 0.24750499 | 0.15837104 | 0.20745921 | 0.09463722 |
| 0.32342007 | 0.40518962 | 0 | 0.13519814 | 0.64037855 |
| 0.14498141 | 0.17764471 | 0.08597285 | 0.36596737 | 0.1955836 |
| 0.133829 | 0.17964072 | 0.01809955 | 0.14685315 | 0.14195584 |
| 0.21561338 | 0.08582834 | 0 | 0.23543124 | 0.04416404 |
| 0.09665428 | 0.15369261 | 0 | 0.17948718 | 0.16403785 |
| 0.21189591 | 0.16966068 | 0 | 0.56177156 | 0.35646688 |
| 0.08921933 | 0.37325349 | 0.22171946 | 0.26573427 | 0.15457413 |
| 0.02973978 | 0.23353293 | 0.0361991 | 0.44055944 | 0.05047319 |
| 0.24535316 | 0.13173653 | 0.0361991 | 0.17482517 | 0.07886435 |
| 0.15241636 | 0.22954092 | 0.01809955 | 0.18181818 | 0.02523659 |
| 0 | 0.15369261 | 0 | 0.25174825 | 0.19242902 |

| 0.04460967 | 0.08582834 | 0.08597285 | 0.08624709 | 0.05993691 |
| --- | --- | --- | --- | --- |
| 0.0669145 | 0.07185629 | 0.16289593 | 0.18881119 | 0.0851735 |
| 0.27509294 | 0.06586826 | 0 | 0.13286713 | 0.12933754 |
| 0 | 0.15568862 | 0 | 0.27505828 | 0.12302839 |
| 0.09665428 | 0.13972056 | 0.09954751 | 0.35664336 | 0.11041009 |
| 0.14126394 | 0 | 0 | 0.08857809 | 0 |
| 0.10037175 | 0.48103792 | 0 | 0.12354312 | 0.33753943 |
| 0.04460967 | 0.1237525 | 0.00904977 | 0.33566434 | 0.07255521 |
| 0.03345725 | 0.26147705 | 0.01357466 | 0.15850816 | 0.13564669 |
| 0.13754647 | 0.16566866 | 0 | 0.19347319 | 0.11671924 |
| 0.13011152 | 0.15369261 | 0.07239819 | 0.12587413 | 0.10094637 |
| 0.17843866 | 0.15169661 | 0 | 0.16083916 | 0.11041009 |
| 0.11152416 | 0.19560878 | 0 | 0.27972028 | 0.09463722 |
| 0 | 0.27944112 | 0 | 0.14452214 | 0.05047319 |
| 0 | 0.25548902 | 0 | 0.2983683 | 0 |
| 0.0669145 | 0.19361277 | 0.02262443 | 0.18181818 | 0.16088328 |
| 0.05204461 | 0.23952096 | 0 | 0.2004662 | 0.09148265 |
| 0.12267658 | 0.06586826 | 0 | 0.07692308 | 0.17665615 |
| 0.11152416 | 0.20758483 | 0.13574661 | 0.31235431 | 0 |
| 0.41263941 | 0.14371257 | 0.16742081 | 0.34032634 | 0.14511041 |
| 0.00743494 | 0.04590818 | 0.04524887 | 0.3030303 | 0.04731861 |
| 0.04832714 | 0.25149701 | 0.01809955 | 0.15617716 | 0.34069401 |
| 0 | 0.11776447 | 0.02714932 | 0.0955711 | 0.05678233 |
| 0 | 0.17964072 | 0.14932127 | 0.26806527 | 0.12933754 |
| 0.10037175 | 0.14371257 | 0 | 0.04195804 | 0 |
| 0.04832714 | 0.15169661 | 0.05882353 | 0.17016317 | 0.04416404 |
| 0 | 0.08982036 | 0 | 0.1048951 | 0.11987382 |
| 0.05947955 | 0.27944112 | 0.01357466 | 0.09090909 | 0.08832808 |
| 0 | 0.03992016 | 0.09049774 | 0.38228438 | 0 |
| 0.04460967 | 0.19760479 | 0 | 0 | 0.15772871 |
| 0.02230483 | 0.28143713 | 0.00452489 | 0.31002331 | 0.10094637 |

| 0.4535316 | 0.2754491 | 0 | 0.3986014 | 0.17981073 |
| --- | --- | --- | --- | --- |
| 0.27137546 | 0.1497006 | 0.14932127 | 0.30769231 | 0.09148265 |
| 0 | 0.03393214 | 0 | 0.12121212 | 0.32807571 |
| 0.27137546 | 0.28942116 | 0.10859729 | 0.05594406 | 0.23028391 |
| 0.14869888 | 0 | 0.09049774 | 0.04662005 | 0 |
| 0.10037175 | 0.35129741 | 0.03167421 | 0.15384615 | 0.3533123 |
| 0.11524164 | 0.21556886 | 0.1719457 | 0.26107226 | 0.13880126 |
| 0.0669145 | 0.10578842 | 0 | 0.13752914 | 0.18611987 |
| 0.15241636 | 0.14371257 | 0.04072398 | 0.31468531 | 0.11041009 |
| 0.04089219 | 0.27345309 | 0.09502262 | 0.12354312 | 0.33123028 |
| 0.2936803 | 0.14570858 | 0.05429864 | 0.3986014 | 0.05678233 |
| 0.02973978 | 0.05788423 | 0.00904977 | 0.17715618 | 0.07570978 |
| 0.11895911 | 0.25948104 | 0.02262443 | 0.1981352 | 0.17665615 |
| 0 | 0.21556886 | 0.06334842 | 0.18881119 | 0.19873817 |
| 0 | 0.13572854 | 0.1040724 | 0.05361305 | 0.28391167 |
| 0.08550186 | 0.08982036 | 0 | 0.37062937 | 0.07255521 |
| 0.0260223 | 0.1237525 | 0.03167421 | 0.12354312 | 0.12302839 |
| 0.24163569 | 0.22954092 | 0.0361991 | 0.35431235 | 0.07570978 |
| 0.01486989 | 0.26147705 | 0.00904977 | 0.14219114 | 0.21451104 |
| 0.81040892 | 0.0998004 | 0.24886878 | 0.44289044 | 0.08201893 |
| 0.02230483 | 0.08982036 | 0 | 0.11888112 | 0.17665615 |
| 0.10037175 | 0.12774451 | 0.08144796 | 0.44522145 | 0.14511041 |
| 0.08178439 | 0.24750499 | 0.04072398 | 0.14452214 | 0.11041009 |
| 0.18215613 | 0.14171657 | 0.12217195 | 0.22843823 | 0.0851735 |
| 0.0669145 | 0.18962076 | 0.01809955 | 0.06759907 | 0.1955836 |
| 0.08178439 | 0.15369261 | 0.04977376 | 0.02564103 | 0.13880126 |
| 0.05576208 | 0.25548902 | 0 | 0.08857809 | 0.02523659 |
| 0.133829 | 0.17764471 | 0 | 0.12354312 | 0.11356467 |
| 0.14126394 | 0.1257485 | 0.01809955 | 0.17715618 | 0.16088328 |
| 0.01486989 | 0.17165669 | 0.00904977 | 0.14685315 | 0.10725552 |
| 0 | 0.15768463 | 0.07239819 | 0.07226107 | 0.0977918 |

| 0 | 0 | 0.19909502 | 0.20512821 | 0 |
| --- | --- | --- | --- | --- |
| 0.07434944 | 0.13173653 | 0.05429864 | 0.12820513 | 0.19873817 |
| 0.18959108 | 0.20758483 | 0.05429864 | 0.34498834 | 0.17350158 |
| 0.14498141 | 0.03792415 | 0 | 0.09090909 | 0 |
| 0.01115242 | 0.03792415 | 0.0361991 | 0.04895105 | 0.11671924 |
| 0 | 0.35329341 | 0.09049774 | 0.23776224 | 0.04416404 |
| 0.14126394 | 0.28942116 | 0.0678733 | 0.34265734 | 0.0977918 |
| 0.04832714 | 0.15169661 | 0.0361991 | 0.15384615 | 0.24921136 |
| 0.03717472 | 0.19161677 | 0 | 0.22377622 | 0.15141956 |
